# Antiplasmodial natural products: an update

**DOI:** 10.1186/s12936-019-3026-1

**Published:** 2019-12-05

**Authors:** Nasir Tajuddeen, Fanie R. Van Heerden

**Affiliations:** School of Chemistry and Physics, University of KwaZulu-Natal, Private Bag X01, Scottsville, Pietermaritzburg, 3209 South Africa

**Keywords:** Malaria, *Plasmodium*, Antiplasmodial, Natural products, Plant metabolites, Marine natural products

## Abstract

**Background:**

Malaria remains a significant public health challenge in regions of the world where it is endemic. An unprecedented decline in malaria incidences was recorded during the last decade due to the availability of effective control interventions, such as the deployment of artemisinin-based combination therapy and insecticide-treated nets. However, according to the World Health Organization, malaria is staging a comeback, in part due to the development of drug resistance. Therefore, there is an urgent need to discover new anti-malarial drugs. This article reviews the literature on natural products with antiplasmodial activity that was reported between 2010 and 2017.

**Methods:**

Relevant literature was sourced by searching the major scientific databases, including Web of Science, ScienceDirect, Scopus, SciFinder, Pubmed, and Google Scholar, using appropriate keyword combinations.

**Results and Discussion:**

A total of 1524 compounds from 397 relevant references, assayed against at least one strain of *Plasmodium*, were reported in the period under review. Out of these, 39% were described as new natural products, and 29% of the compounds had IC_50_ ≤ 3.0 µM against at least one strain of *Plasmodium*. Several of these compounds have the potential to be developed into viable anti-malarial drugs. Also, some of these compounds could play a role in malaria eradication by targeting gametocytes. However, the research into natural products with potential for blocking the transmission of malaria is still in its infancy stage and needs to be vigorously pursued.

## Background

**Fig. 1:**
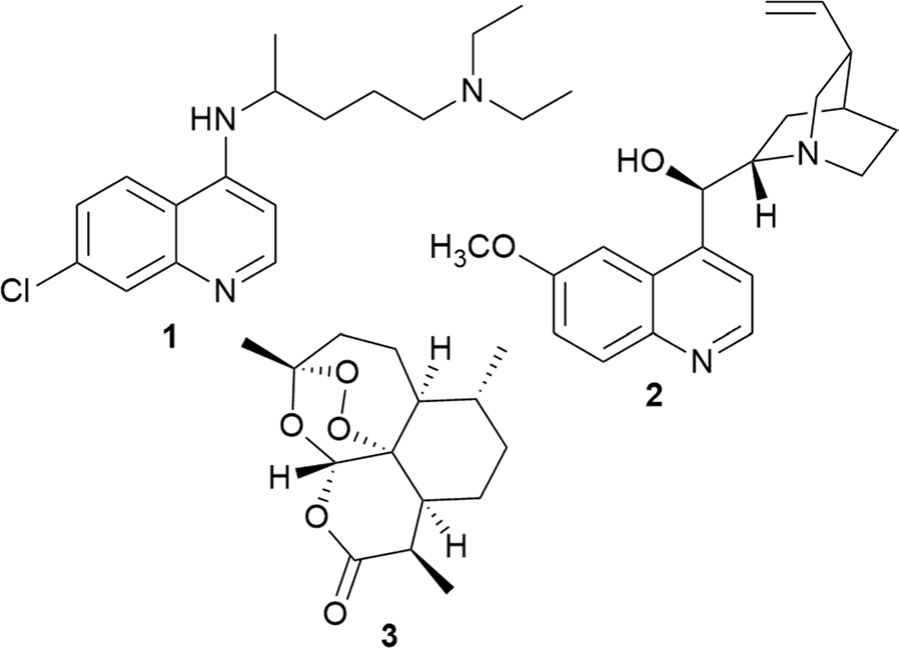
Structures of anti-malarial drugs

Malaria remains a serious parasitic disease in the world, with 219 million infections and 435,000 deaths cited for 2017 in the latest World Malaria Report [1]. An assessment of older and more recent malaria maps shows that the disease has been geographically restricted during the twentieth century, and has remained endemic in the poor regions of the world where the climate is suitable for transmission [2–5]. In Africa, where the disease burden is the highest, there has been a general decline in the trend of *Plasmodium falciparum* malaria, from a prevalence of 40% in 1910–1929 to about 24% in 2010–2015 [6]. However, in the high transmission belt covering large parts of West and Central Africa, there is little change. This shrinkage of the malaria map has been interrupted by periods of rapid increase and decline in transmission [6]. The significant decline in malaria prevalence between 1945 and 1949 and again between 2005 and 2009 correlates with deliberate intervention programs. Each of these declines was preceded by a rise in malaria prevalence. The introduction of chloroquine (**1**) (Fig. 1) and dichlorodiphenyltrichloroethane (DDT) in 1945 and widespread use of insecticide-treated bed nets and artemisinin-based combination therapy (ACT) between 2005 and 2009 are partly credited for these declines. The rapid spread of resistance to chloroquine and emerging resistance to ACT in Africa, coupled with an increase in cases of vector-borne diseases in places like the USA, poses a threat to the gains that have been achieved in malaria control [6–9]. A World Health Organization (WHO) malaria report already shows a rise in malaria incidences in 2016 compared to 2015 [1]. Also, the sustained decline in mortality due to malaria since 2010 has stalled in some regions between 2015 and 2016 and has increased in other regions [1]. Therefore, the continued search for new anti-malarial agents remains an urgent priority.

Malaria chemotherapy has a strong historical link to natural products. The most successful anti-malarial agents have their origins in plant metabolites. The first successful anti-malarial drug was quinine (**2**), isolated from the bark of the South American *Cinchona* tree. This compound served as a lead structure in the development of the successful synthetic anti-malarial chloroquine (**1**), which, in recent years, has fallen out of favour as a result of the development of drug resistance by the parasite. Likewise, artemisinin (**3**) was isolated from the leaves of a Chinese medicinal plant, *Artemisia annua*. *Cinchona* bark and *Artemisia annua* were historically used to treat fever. Sophisticated target identification strategies following the sequencing of the *P. falciparum* genome in addition to the application of combinatorial chemistry hit identification strategies, did not lead to the expected increase in the number of new successful anti-malarial agents, and it is plausible that the next anti-malarial agents will be identified from a natural source again [10]. Due to structural characteristics such as multiple stereocenters, flexible conformations, presence of heteroatoms, natural products are more likely than synthetic compounds to have multiple targets and/or new targets. Researchers investigating natural products as potential anti-malarial drugs need to incorporate the screening of the compounds for the interaction with newly identified druggable targets, such as PfATP4 and DHODH, in order to identify hits/leads. Therefore, the continued exploration of natural products as antiplasmodial agents is of great scientific interest. Equally important is the need to review the literature in the field of malaria chemotherapy to provide a perspective for future research.

Plant-derived antiplasmodial compounds organized according to plant families covering the literature from 1990 to 2000 have been reviewed [11]. Similarly, reviews categorizing antiplasmodial compounds isolated from plants according to phytochemical classes have been conducted by Bero et al. (2005–2011) [12, 13], Nogueira and Lopes (2009–2010) [14], and Wright (2000–2010) [15]. Finally, reviews covering antiplasmodial marine natural products up to 2009 have been published by Laurent and Pietra, and Fattorusso and Taglialatela-Scafati [16, 17]. However, several new antiplasmodial chemotypes have been reported in the literature since 2010, in addition to the recent increase in antiplasmodial chemical scaffolds emerging from non-vegetal sources. Against this background, this article reviews the literature on natural products with antiplasmodial activity from 2010 to the end of 2017 and is organized according to structural types of compounds.

A thorough search of the relevant scientific databases, including Web of Science, ScienceDirect, Scopus, SciFinder, Pubmed, and Google Scholar, was conducted. The keyword combinations of antiplasmodial, anti-malarial, *Plasmodium*, and malaria compounds together with plant, phytochemical, marine sponge, nudibranch, alga, cyanobacteria, mushroom, fungi, and *Streptomyces* were used in the search. Within the period under review, a total of 1524 compounds from 397 relevant references, assayed against at least one strain of *Plasmodium*, were reported. Of these compounds, 593 (39%) were described as new natural products. The number of compounds isolated from vegetal material was 1165 (76%), while 359 were from non-plant sources. Among the compounds isolated from non-plant sources, 192 (53%) were described as new, while 401 (34%) of the compounds isolated from plants were new. These numbers show that medicinal plants are still the most comprehensively explored source for antiplasmodial compounds, which may be related to ease of access. However, this review also shows the potential of non-plant material to furnish new chemotypes. Regarding the potency of these compounds, 857 (56%) had IC_50_ ≤ 10 µM, 625 (41%) had IC_50_ ≤ 5.0 µM, and 447 (29%) had IC_50_ ≤ 3.0 µM against at least one strain of *Plasmodium*. The cut-off value for potency remains an issue of ongoing debate, but the industry standard for considering a pure compound to be active is generally accepted as IC_50_ ≤ 10 µM [18].

**Fig. 2:**
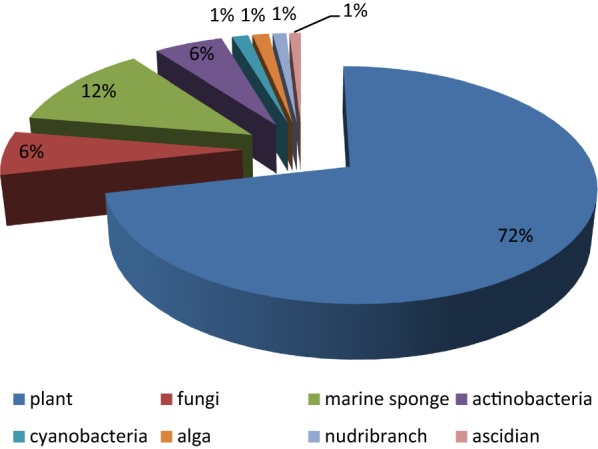
Breakdown of sources for antiplasmodial compounds discussed in this review

The in vitro antiplasmodial activities described in this review were obtained with various *P. falciparum* strains with different drug sensitivities. The chloroquine-sensitive strains were 3D7, NF54, D6, HB3, F32, D10, TM4/8.2, and MRC-pf-20. Other strains used were the chloroquine-resistant strains Dd2, FcB1, *Pf*INDO, FcM29, MRC-pf-303, and the multidrug-resistant K1, TM90-C2A, TM93-C1088, W2, TM90-C2B, TM91-C235, NHP1337, FCR3, K1CB1, and W2mef strains. Different assays were used to determine the in vitro antiplasmodial activities of the compounds. The most commonly cited methods were the radioactive hypoxanthine-incorporation assay, the colourimetric enzyme-linked immunosorbent assays (ELISA) that measure *P. falciparum* lactate dehydrogenase protein (pLDH) and histidine-rich protein 2 (HRP2), a DNA-based fluorometric method using the PicoGreen (SYBR) assay, and microscopy. These methods have different sensitivities and the advantages and disadvantages have been investigated and reviewed [19–21]. Regardless of the in vitro assay method, in this review, only compounds with IC_50_ ≤ 3.0 µM were considered to be of interest for further studies. Compounds with higher IC_50_ values are listed in Additional file 1. Among the compounds that are discussed, 317 (70.9%) were isolated from 50 different plant families, while 130 were from non-plant sources comprising of different species of marine sponges, alga, fungi, ascidians, nudibranch, cyanobacteria and actinobacteria (Fig. 2).

The antiplasmodial natural products are organized in seven classes, i.e. endoperoxides, alkaloids, terpenes, polyphenols, quinones and polyketides, macrocycles, and cyclic phosphotriesters, with subclasses where applicable. The review concludes with a summary of the cellular targets in *Plasmodium* identified for natural products and natural products with transmission-blocking potential.

## Endoperoxides

**Fig. 3:**
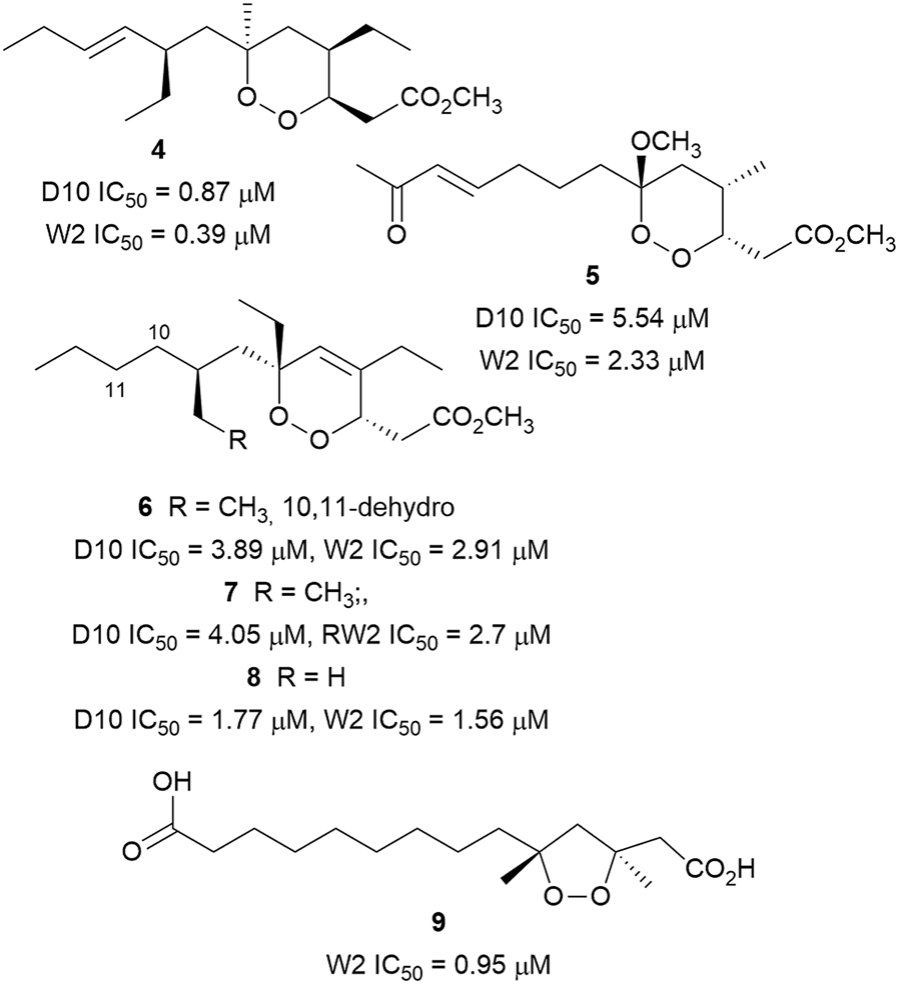
Structures of polyketide-derived endoperoxides

The profound success of artemisinin (**3**) and derivatives as drugs for the treatment of malaria prompted the selection of endoperoxides as the first class of compounds to be discussed in this review. Endoperoxide polyketides (Fig. 3) belonging to the 1,2-dioxane and 1,2-dioxolane structural class with proven antiplasmodial activity have been isolated from marine sponges. The structural variability includes different lengths of the ‘western’ side chain, different branching patterns, and a fully saturated or monounsaturated cyclohexane ring, all of which affect the bioactivity [22]. Plakortin (**4**), isolated from the marine sponge *Plakortis simplex*, is the archetype compound of this class. It demonstrated potent submicromolar antiplasmodial activity against chloroquine-sensitive and -resistant parasites [23, 24]. A plausible mechanism of action, inspired by results obtained with artemisinin and other trioxanes, was proposed for these structurally simpler molecules. It involves an initial reaction of the peroxidic bond with Fe(II) heme to form an *O*-centred radical, which is transformed into a *C*-centred radical following intramolecular rearrangement. The rearranged *C*-centred radical on the ‘western’ alkyl side-chain represents the toxic species that kills parasites. The minimum structural requirements for antiplasmodial activity of this class of compounds have been identified. The oxygen atoms of the endoperoxide bond must be accessible to Fe(II), and the molecule must adopt the appropriate conformation for the intramolecular rearrangement through a concerted intramolecular electron transfer [25]. The lower antiplasmodial activity of the peroxyketal derivative manadoperoxide C (**5**) and its analogues isolated from Indonesian-sourced *Plakortis* cfr. *simplex* was rationalized on the basis of these structural requirements. The 6-methoxy substituent of the manadoperoxides constitutes a hindrance for Fe(II) to the peroxide bond, leading to lower activity [23]. The isolation of endoperoxide analogues **6**–**8** with a 1,2-diox-4-ene ring from *Plakortis simplex* allowed an extension of the structure-activity relationship (SAR). For structurally similar compounds, the unsaturated derivatives were more active due to stereochemical influence, although they were still less active than **4**. The lower activity compared to **4** could also be explained by the relative inaccessibility of the peroxide oxygen due to steric hindrance [22]. These results indicate that structural changes affecting the conformational behaviour of this class of compounds, profoundly influence the antiplasmodial activity, and this knowledge will be beneficial for the design of optimized analogues [23]. The related 1,2-dioxolane epiplakinidioic acid (**9**) was isolated from Puerto Rican-sourced *P. halichondrioides* and inhibited *P. falciparum* W2 strain. However, it was also cytotoxic against a panel of cancerous cell lines [26].

**Fig. 4:**
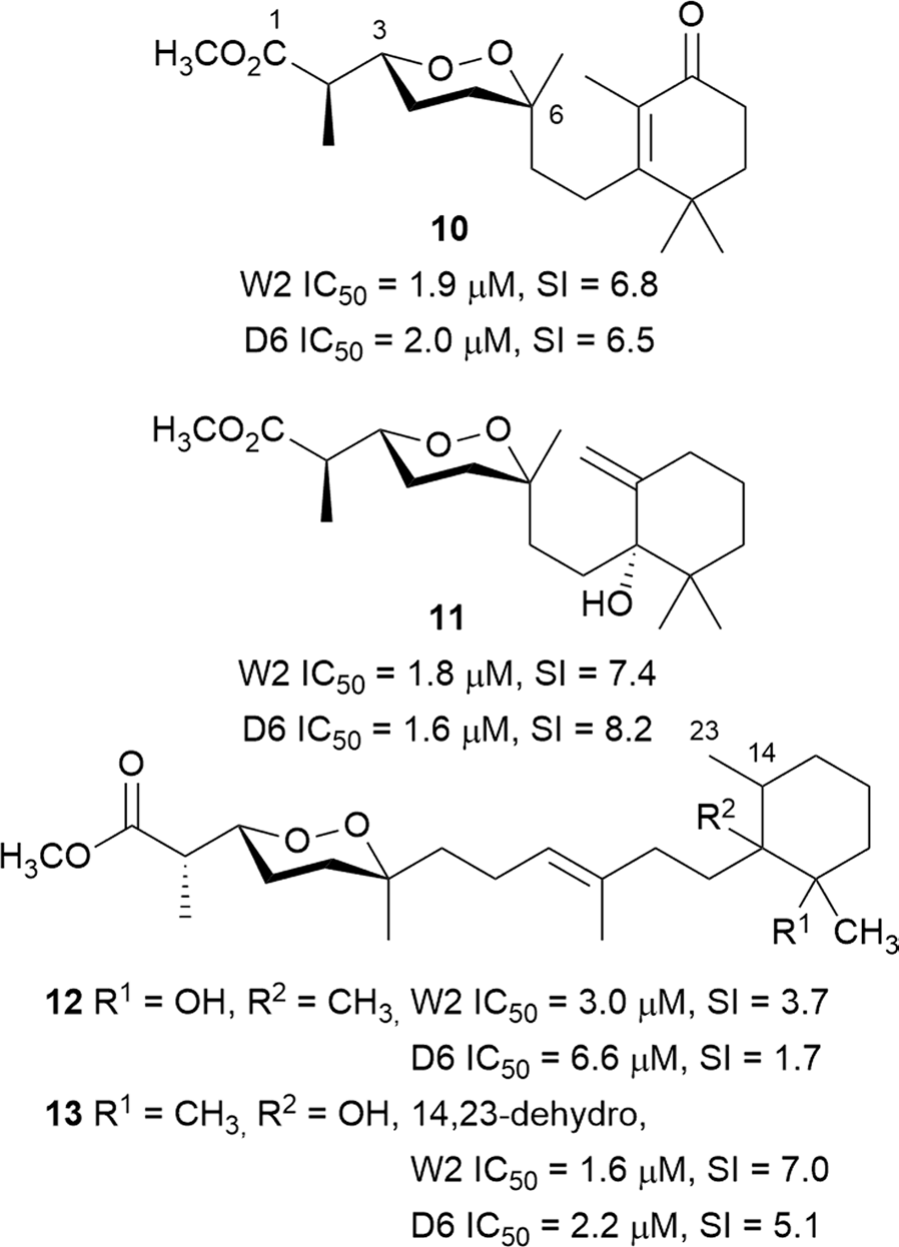
Structures of norditerpene endoperoxides

The norditerpenoid cycloperoxides, diacarperoxides A (**10**), J (**11**), diacarnuperoxide N (**12**), and 2,3,6-epihurghaperoxide (**13**) (Fig. 4) were isolated from the South China Sea sponge *Diacarnus megaspinorhabdosa* and inhibited both the W2 and D6 *P. falciparum* strains. In contrast to the polyketide-derived counterparts, the SAR of the norditerpene endoperoxides has not been studied in detail. It was suggested that variations in the configuration at C-2, C-3, and C-6, or the cyclohexane ring and the side chains do not significantly affect activity [27, 28]. However, more detailed SAR studies need to be conducted to gain a better understanding of the activity.

## Alkaloids

Among the 447 isolated natural products with IC_50_ ≤ 3.0 µM reported in this review, 31.9% are alkaloids.

### Naphthylisoquinolines

**Fig. 5:**
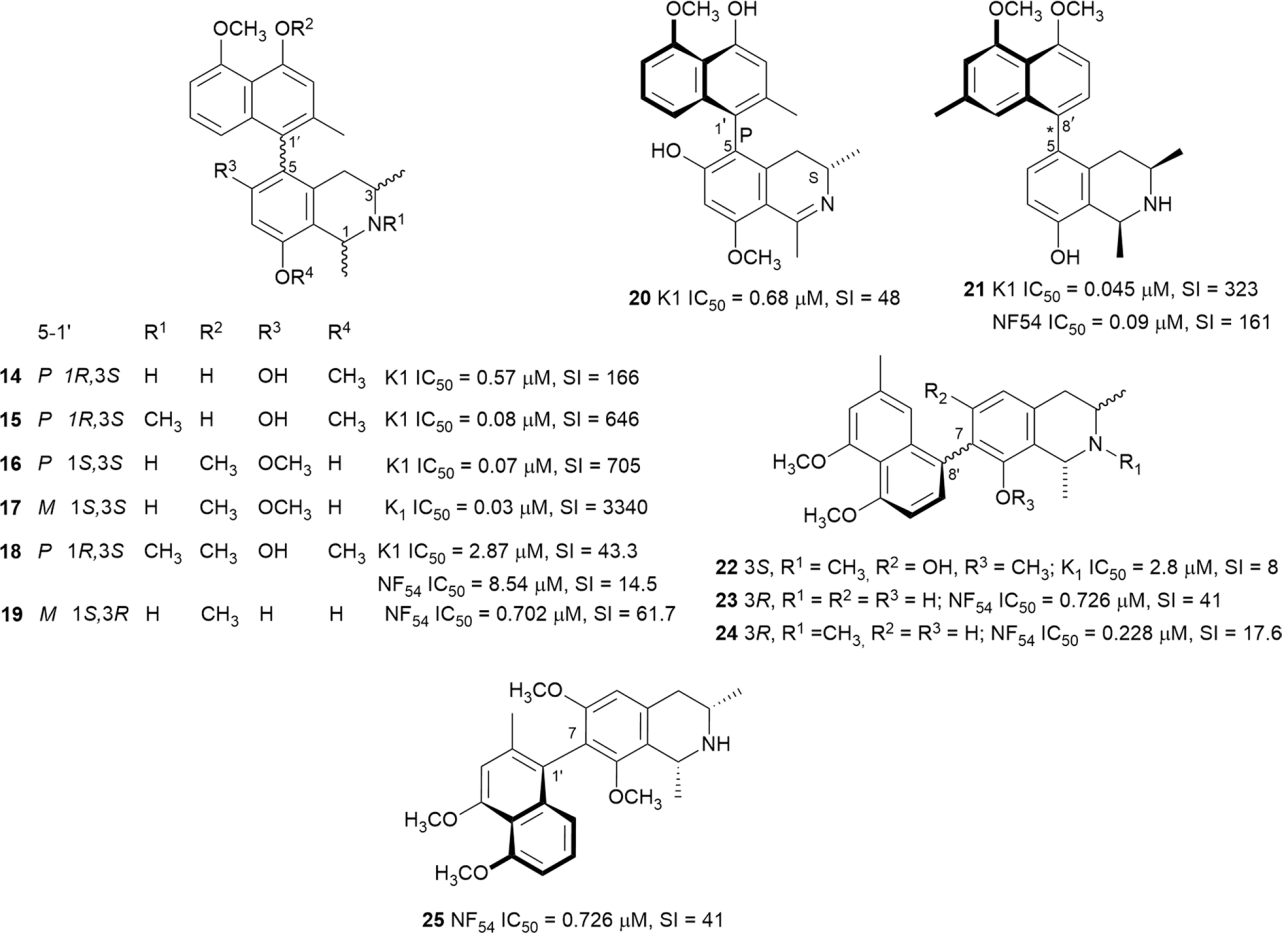
Structures of monomeric naphthylisoquinolines

**Fig. 6:**
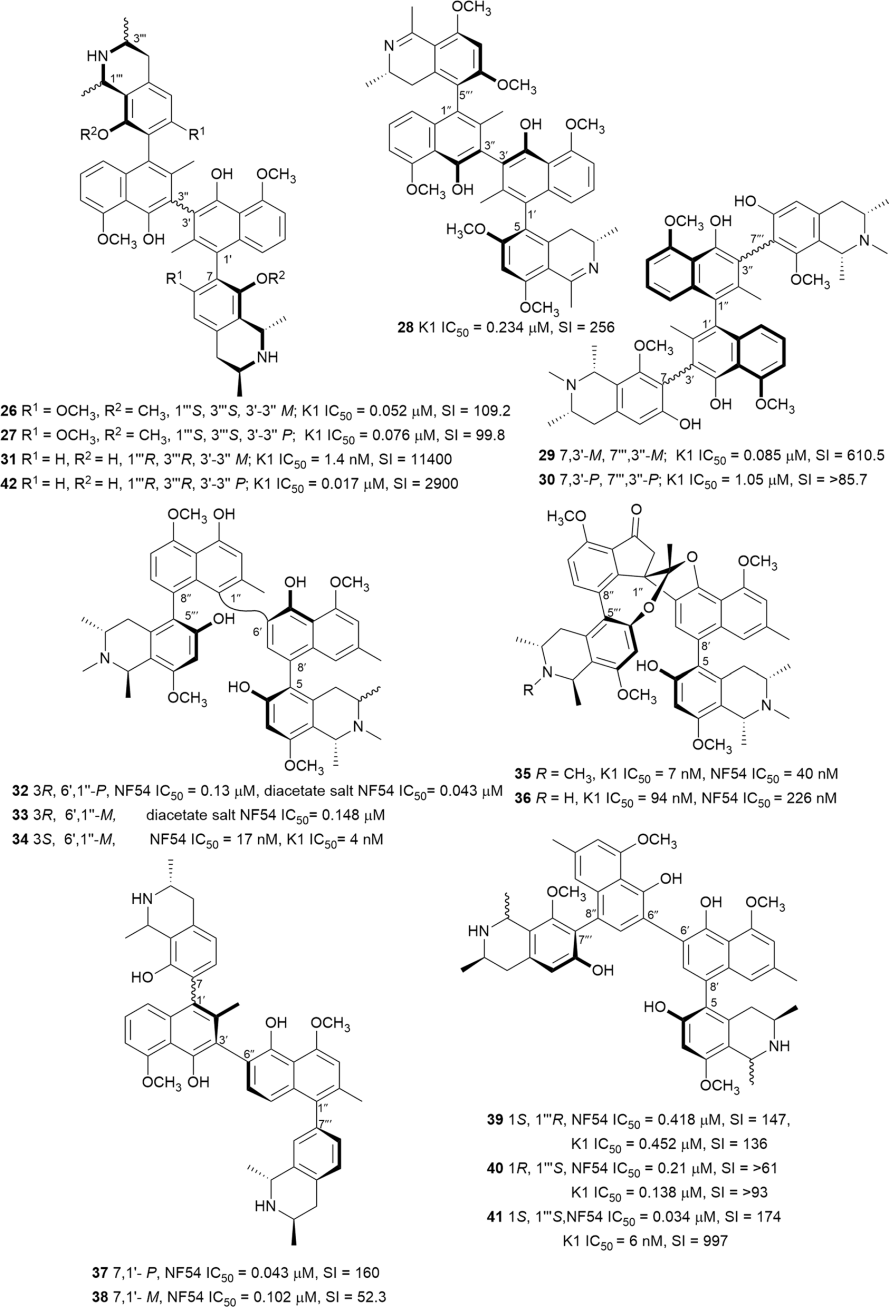
Structures of dimeric naphthylisoquinolines

The naphthylisoquinolines are a unique class of polyketide-derived biaryls of natural origin. These compounds are found exclusively in the Ancistrocladaceae from Central Africa and Southeast Asia, and Dioncophyllaceae endemic to the coast of West Africa. The compounds are composed of naphthalene and isoquinoline moieties and are biosynthetically derived from the acetate-polymalonate pathway [29, 30]. The naphthalene and isoquinoline regions are coupled through a rotationally restricted C–C or C–N axes. This rotational hindrance gives rise to axial chirality, while the isoquinoline unit can have up to three stereocentres. Twelve of the monomeric naphthylisoquinolines (**14**–**25**) (Fig. 5) are discussed in this review. Dimeric naphthylisoquinolines have been described from species of the *Ancistracladus* genus, and these dimers join four aryl units through three biaryl axes and thereby potentially doubling the number of stereocentres [31]. Seventeen of the dimeric naphthylisoquinolines (**26**–**42**) (Fig. 6) are mentioned in this report. The Ancistrocladaceae mostly produce C-6 oxygenated alkaloids with an *S*-configuration at C-3, and these are called the Ancistrocladaceae-type, while the alkaloids of Dioncophyllaceae exclusively have an *R*-configuration at C-3 and lack oxygenation at C-6 and are called the Dioncophyllaceae-type [30]. Several of these compounds displayed nanomolar selective inhibition of the *Plasmodium* parasite viability.

Ancistectorines A_1_ (**14**), *N*-methyl A_1_ (**15**), A_2_ (**16**), 5-*epi*-A_2_ (**17**), A_3_ (**20**), and C_1_ (**22**) were isolated from the twigs of Chinese *Ancistrocladus tectorius* [32]. The six alkaloids potently inhibited the K1 strain of *P. falciparum* without cytotoxicity against rat skeletal myoblast (L6) cells. The 5,1′-coupled compounds **15**, **16**, and **17** were 3–7 times more active than chloroquine with **17** having an SI > 3000 [32]. The known *N*-methylated 5,1′-coupled ancistrocline (**18**) from the same plant also showed encouraging antiplasmodial activity against the K1 and NF54 strains. In addition to low cytotoxicity against L6 cells, compound **18** was 2–3 times more active against chloroquine-resistant K1 than the chloroquine-sensitive NF54 strain [33]. However, the additional methoxy group on the naphthalene unit of **18** led to > 30 fold decrease in activity compared to **15**. The new dioncophyllines C_2_ (**19**) and F (**21**), and the known ancistrocladisine A (**25**) and 5′-*O*-methyldioncophylline D (**23**) were isolated from the root bark of Congolese *Ancistrocladus ileboensis* [30]. The total synthesis of **21**, which was the first reported natural 5,8′-coupled dioncophyllaceous alkaloid, was achieved by palladium-catalyzed Suzuki–Miyaura cross-coupling of the two aryl moieties. Furthermore, the leaves of *Ancistrocladus ileboensis* yielded the 7,8′-coupled dioncophylline D_2_ (**24**), which was also previously unreported. Compounds **19**, **21**, **23**, **24**, and **25** were found to be active against the NF54 strain, with **21** displaying double the activity against the K1 over the NF54 strain. Furthermore, the new compounds were non-toxic to L6 cells with SI values ranging from 61 to 586 [30]. It is worthy to note that axial chirality influences the antiplasmodial activity of the naphthylisoquinolines when they exist as atropo-diastereomers. The *M*-configured analogues such as **17** generally showed superior selective antiplasmodial activity compared to the *P*-configured counterparts such as **16**.

Dimerization of the naphthylisoquinoline core resulted in the rotationally-hindered 1,1′- or 3,3′-inked shuangancistrotectorines A–E (**26**–**30**) from the twigs of the Chinese *Ancistrocladus tectorius*. This was the first report of a natural product featuring three consecutive stereo axes, which in addition to the tetrahydroquinoline stereocentres, confers up to seven stereogenic units [34]. Biological assessment of these compounds revealed sub-micromolar antiplasmodial activity against the K1 strain coupled to low toxicity against L6 cells (SI = 99.8–610.6), with compounds **26**, **27**, and **30**, in particular, displaying antiplasmodial activity superior to that of chloroquine [34]. Similarly, jozimine A_2_ (**31**), the first reported dioncophyllaceae-type sterically-hindered 3,3′-coupled dimeric naphthylisoquinoline isolated from a Congolese *Ancistrocladus* sp., displayed sub-nanomolar inhibitory activity, superior to that of chloroquine against the NF54 strain coupled to low cytotoxicity against L6 cells (SI > 11,400) [35]. Mbandakamines A (**32**) and B (**33**), the first dimeric naphthylisoquinolines featuring the unsymmetrical 6′,1″-coupling of the naphthalene units were isolated from the leaf of another uncharacterized Congolese *Ancistrocladus* sp. The diacetate salts of these highly sterically-hindered compounds were more active against the NF54 strain than the free bases, possibly due to increased solubility [36]. Another unidentified Congolese *Ancistrocladus* sp. yielded the unsymmetrically 6′,1″-coupled mbandakamine B_2_ (**34**), together with two other unique dimers named spirombandakamines A_1_ (**35**) and A_2_ (**36**). Compounds **35** and **36**, which incorporate both a five-membered ketone ring alongside seven- and five-membered oxygenated heterocyclic rings into the dimeric structure, exhibited nanomolar antiplasmodial activity against both the K1 and NF54 parasite strains. The open-chain **34** was proposed as the biosynthetic precursor to the spiro-fused **35**, but the higher antiplasmodial activity of **34** as compared to **35** and **36** implies that cyclization is not beneficial to activity [37]. Jozilebomines A (**37**) and B (**38**), two unsymmetrical 3,6″-coupled dimers isolated from the root extract of the Congolese *Ancistrocladus ileboensis*, exhibited selective antiplasmodial activity against NF54 *P. falciparum* strain, with weak toxicity towards L6 cells (SI = 160 and 52.3, respectively). However, this activity was lower than the related symmetrically coupled jozimine A_2_ (**31**) [38]. Three structurally unique heterodimeric naphthylisoquinolines, ealapasamines A–C (**39**–**41**), were isolated from the leaf of *Ancistrocladus ealensis*. The ealapasamines are the first reported unsymmetrical dimers in which the constituent monomeric naphthylisoquinoline units are linked at different positions, i.e. one 5,8′-coupled monomer links to another 7,8′-coupled unit at the 6′ position of the respective naphthanyl subunits. This subsequently results in three different biaryl linkages, with the inter-naphthanyl biaryl axis being configurationally unstable. Compounds **39**–**41** were active against *P. falciparum* (K1 and NF54) with low nanomolar IC_50_ values and low toxicity to L6 cells [39].

The bioactivity of the dimeric naphthylisoquinolines, as with the monomers, is influenced by axial chirality as, for example, seen in the superior activity of jozimine A_2_ over the atropo-diastereomer, 3′-*epi*-jozimine A2 (**42**) [29, 35]. So far, jozimine A_2_ demonstrated the best antiplasmodial activity (IC_50_ = 1.4 nM) against the chloroquine-sensitive NF54 strain, while mbandakamine B_2_ is the most active dimer against the chloroquine-resistant K1 strain (IC_50_ = 4.0 nM). With potent in vitro activities and high selectivity for the parasites over mammalian cell lines, the dimeric naphthylisoquinolines can be considered as viable anti-malarial hits. It will be worthwhile to study the mechanism of action as well as in vitro and in vivo potency against a comprehensive panel of drug-sensitive and -resistant parasites. Equally important for an anti-malarial drug is the need for oral bioavailability, and hence pharmacokinetic studies are highly desirable. The synthesis of some dimeric naphthylisoquinolines has been reported, and since only small amounts of these potent compounds are present in plant material, the syntheses of these compounds will be essential for further developments [40].

### Benzylisoquinolines and other isoquinolines

**Fig. 7:**
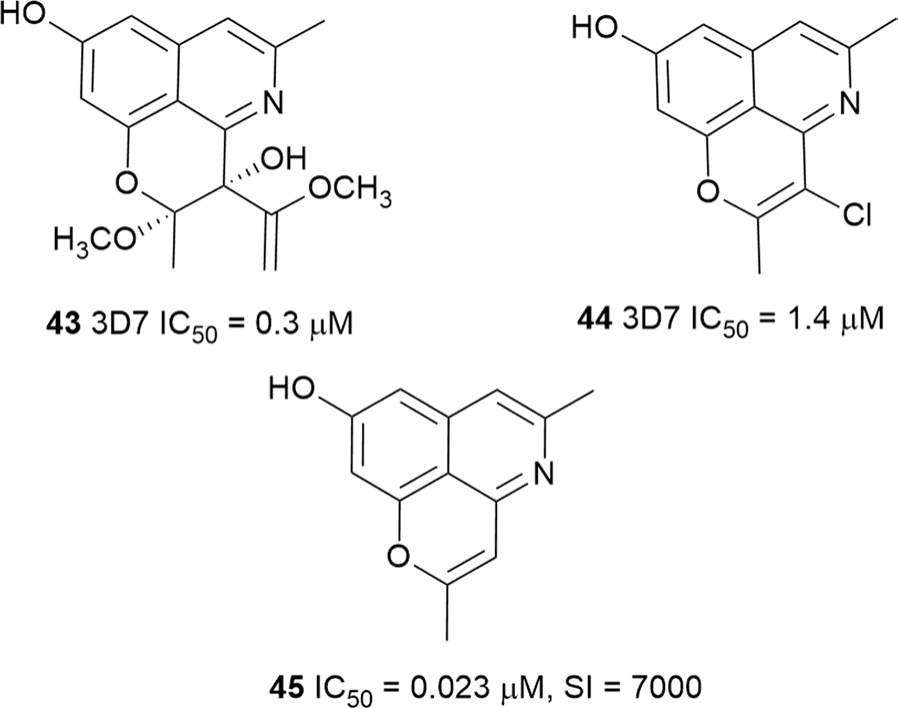
Structures of cassiarins

Three new tricyclic isoquinoline alkaloids (Fig. 7) were isolated from the leaf extract of *Cassia siamea* (Fabaceae), a plant traditionally used to treat periodic fever and malaria in Indonesia. Among the isolated compounds, cassiarin J (**43**) and the first halogenated cassiarin congener, cassiarin K (**44**), inhibited the in vitro growth of 3D7 *P. falciparum* [41]. However, both compounds **43** and **44** were less active than chemically simpler and the potent cassiarin A (**45**), suggesting that the role of the pyran ring of the cassiarins in antiplasmodial activity should be further explored in structure–activity relationship (SAR) investigations [42].

**Fig. 8:**
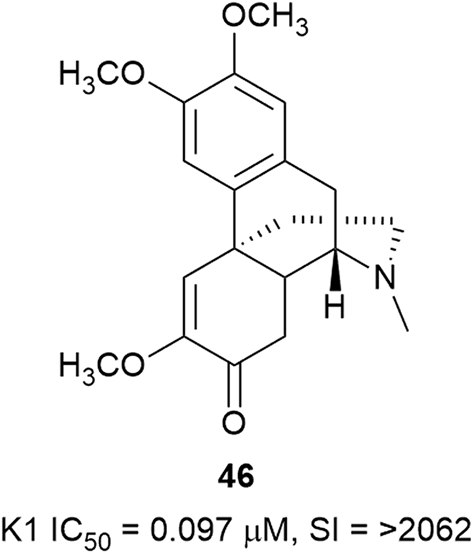
Structure of (−)-milonine

**Fig. 9:**
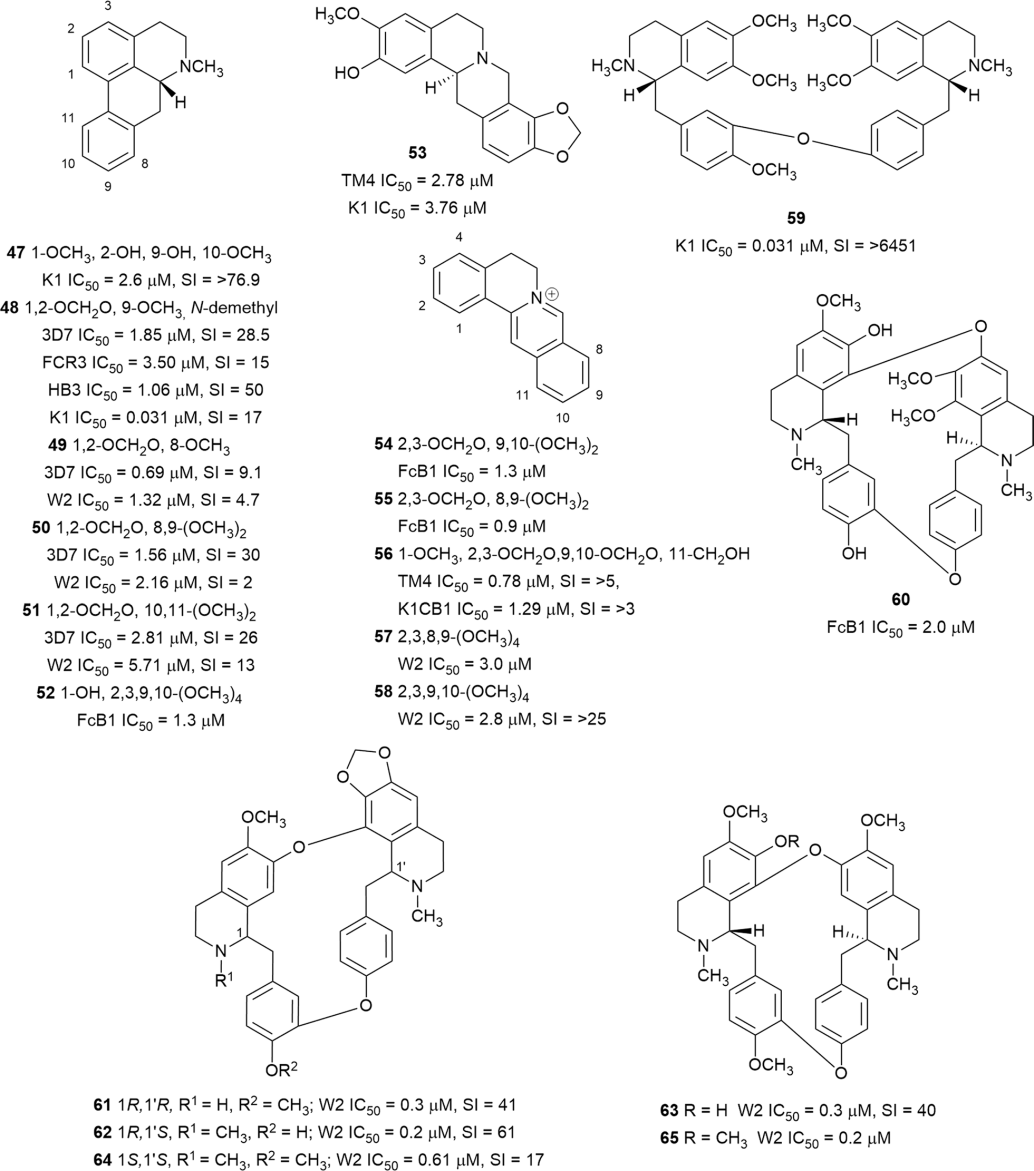
Structures of benzylisoquinoline alkaloids

The l-tyrosine-derived benzylisoquinoline alkaloids form a structurally diverse group of plant-derived compounds, many of which are associated with potent biological activities, and antiplasmodial activity is no exception. During the review period, the activity of one morphinanedienone alkaloid (**46**) (Fig. 8), six aporphines (**47**–**52**), six berberine-type compounds (**53**–**58**) and seven bisbenzyltetrahydroisoquinoline alkaloids (**59**–**65**) (Fig. 9) were reported. The morphinanedienone alkaloid, (−)-milonine (**46**) from the bark of *Dehaasia longipedicellata* (Lauraceae), exhibited sub-micromolar selective antiplasmodial activity against K1 parasites [43]. Carraz et al. reported that a derivative of a related morphinan, tazopsine, is active against the liver stages of the parasite and that this class of compounds may have potential as anti-malarial leads [44].

Following the screening of crude extracts of Malaysian medicinal plants, the extract of *Dehaasia longipedicellata* was identified as a promising antiplasmodial starting point. Chemical investigation of the bark extract afforded boldine (**47**) and (−)-*O*,*O*-dimethylgrisabine (**59**) as the most active constituents, both of which were active against the K1 strain of *P. falciparum,* with **59**, in particular, outperforming chloroquine in this assay. The compounds were not cytotoxic to a pancreatic cancer cell line (hTERT-HPNE) at 200 µM, indicating selective toxicity to the parasite [43]. Based on the antiplasmodial screening of 794 plant extracts from Papua New Guinea and Australia, four species were selected for further investigation, one of which was *Stephania zippeliana* (Menispemaceae) [45]. *Stephania zippeliana* yielded xylopine (**48**), which selectively inhibited the 3D7, FCR3, HB3, and K1, in addition to the D6 and W2 *P. falciparum* strains [46, 47]. Bioassay-guided fractionation of *Stephania venosa* tubers yielded the aporphine alkaloids stephanine (**49**), crebanine (**50**), and *O*-methylbulbocapnine (**51**) as antiplasmodial principles. Unfortunately, the most active alkaloid against 3D7 and W2 parasites, stephanine (**49**), was also the most cytotoxic to cancerous and non-cancerous cell lines [48]. Chemical interrogation of the root of *Thalictrum flavum* (Ranunculaceae) yielded the aporphine alkaloid, preocoteine (**52**), the protoberberines, pseudoberberin (**54**) and berberin (**55**), and the bisbenzylisoquinoline thaligosidine (**60**). The compounds exhibited antiplasmodial activity against the FcB1 strain. However, the activity of the quaternary protoberberines was not selective towards the parasite [49]. The tetrahydroprotoberberine alkaloid cheilanthifoline (**53**), isolated from *Corydalis calliantha* (Papaveraceae), an annual herb used in Bhutanese traditional medicine to treat malaria, also displayed activity against the TM4 strain of *P. falciparum* [50]. The aerial part of *Meconopsis simplicifolia* (Papaveraceae) is an ingredient in more than eight Bhutanese traditional medicine formulations and has displayed potent antiplasmodial activity against TM4/8.2 and K1CB1 strains of *P. falciparum* (IC_50_ = 0.4 and 6.39 µg/mL, respectively) [51]. Extraction and purification of the aerial components of *Meconopsis simplicifolia* yielded the protoberberine-type benzylisoquioline, simplicifolianine (**56**), which showed potent antiplasmodial activity against the TM4/8.2 and K1CB1 strains in the absence of significant cytotoxicity to Vero and human oral carcinoma (KB) cells [52].

Three bisbenzylisoquinoline alkaloids, 2-norcepharanthine (**61**), cepharanoline (**62**), and fangchinoline (**63**), which were isolated from *Stephania rotunda* tuber as minor constituents, all showed potent antiplasmodial activity against the W2 strain with SI ≥ 40 [53]. Encouragingly, these compounds were twice as active as the major alkaloid of *Stephania rotunda*, cepharanthine (**64**), which was previously reported to inhibit parasites in vitro and in vivo [54]. Further investigation found that the *Stephania rotunda* alkaloid **64** and the protoberberine-type benzylisoquinolines palmatine (**57**) and pseudopalmatine (**58**) also inhibited the in vitro viability of *P. falciparum* W2. However, while **64** was found to be cytotoxic against K562S cells, this was not the case for **58** at the highest tested concentration (IC_50_ > 25 µM). These findings supported the use of *Stephania rotunda* in malaria treatment by traditional healers in Cambodia [55]. Desgrouas et al. concluded that cepharanthine (**64**) affected the ring stage of the parasite [56, 57]. Furthermore, in in vitro studies, **64** had a synergistic antiplasmodial effect with the anti-malarial drugs chloroquine, atovaquone, and piperaquine, but had an antagonistic effect with dihydroartemisinin and mefloquine [56, 57]. In in vivo experiments, combinations of **64** and chloroquine, and **64** and amodiaquine were assayed in mice. Both combinations delayed parasitic growth and extended the life expectancies of the mice compared to the drugs alone [57]. Fangchinoline (**63**) and the methyl ether tetrandrin (**65**), both produced by *Stephania tetrandra*, are not only cytotoxic against cancer cell lines, but also reverse resistance of multidrug-resistant human cancer cells by inhibiting P-glycoprotein activity, thereby increasing drug concentration in the cells [58]. The resistance-reversal effect was also observed with *Plasmodium*; Ye and Van Dyke reported that **65** in combination with chloroquine resulted in a 44 fold potentiation of parasite killings [59]. These authors also reported on the structure–activity relationship of bisbenzylisoquinoline [59]. For activity, the configuration of C-1′ of the ‘right-hand’ ring should be *S*. The configuration of C-1 of the ‘left-hand’ ring has little influence on the antiplasmodial activity. Furthermore, the position of the bridges connecting the two monomeric benzylisoquinolines also plays a role and compounds with ether bridges between C-8 to C-7′, and between 11 and 12′ (head–head and tail–tail dimer) have the highest antiplasmodial activity.

### Phenanthrene derivatives: phenanthridine, phenanthroindolizidine, and phenanthrene alkaloids

**Fig. 10:**
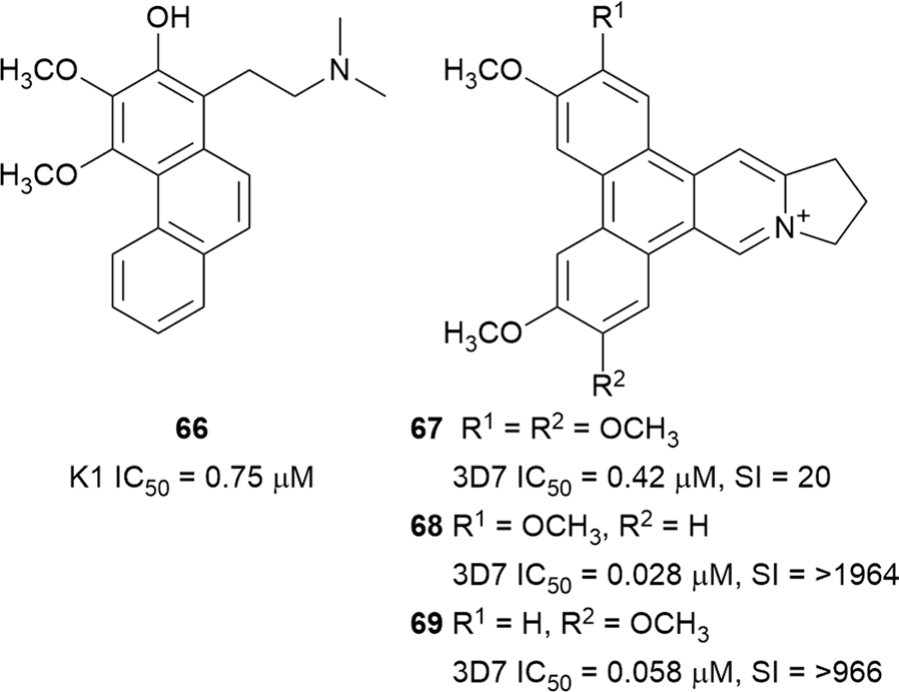
Structures of phenanthrene alkaloids

The dichloromethane bark extract of *Cryptocarya nigra* (Lauraceae) afforded the phenanthrene alkaloid 2-hydroxyatherosperminine (**66**) (Fig. 10), which was found to be active against the *P. falciparum* K1 strain. The significantly improved activity of **66** over the C-2 deoxy analogue atherosperminine indicates a possible important region of the pharmacophore [60].

The methanol extract of *Ficus septica* (Moraceae) twigs exhibited in vitro antiplasmodial activity against the 3D7 strain (IC_50_ = 2.0 µg/mL). Bioassay-guided fractionation of the chloroform fraction of the active methanol extract led to the isolation of the known compounds, dehydrotylophorine (**67**), dehydroantofine (**68**) and tylophoridicine (**69**) (Fig. 10). The three phenanthroindolizidine alkaloids inhibited the 3D7 strain with sub-micromolar IC_50_ and had low cytotoxicity. The selective antiplasmodial activity of these alkaloids indicates that other alkaloids of this class should be further explored [61].

**Fig. 11:**
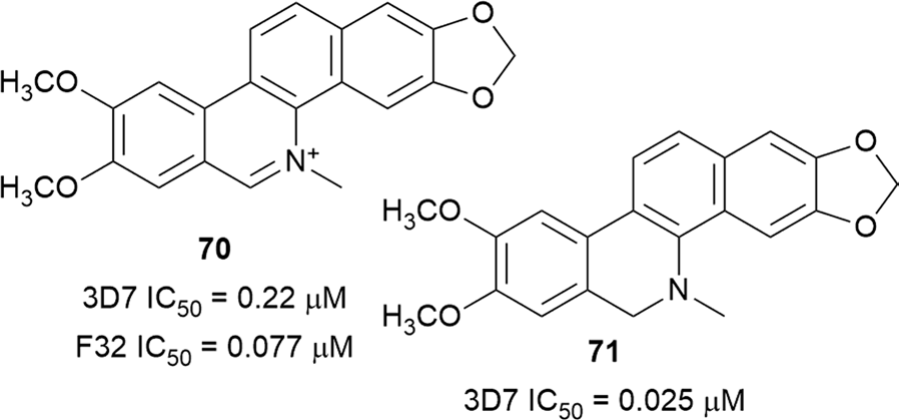
Structures of nitidine (**70**) and dihydronitidine (**71**)

The benzophenanthridine alkaloid nitidine (**70**) (Fig. 11) was isolated as the main antiplasmodial compound from *Zanthoxylum chalybeum* (Rutaceae) and *Zanthoxylum rhoifolium* [62, 63]. Compound **70** displayed rapid activity against chloroquine-sensitive and -resistant parasites with little evidence of cross-resistance, consistent with previous reports [64]. Treatment of *Plasmodium berghei*-infected mice with **70** gave an ED_50_ value of 18.9 mg/kg/day without mice mortality. Nitidine (**70**) did not interfere with parasite DNA replication and was found to localize in the parasite cytoplasm. The mechanism of action of **70** might be similar to that of chloroquine since it formed a complex with heme and inhibited the formation of β-haematin in vitro [62]. In contrast to the rapid activity of **70**, dihydronitidine (**71**), isolated from *Zanthoxylum heitzii* bark, displayed a slow-acting drug effect against 3D7 parasites. This slow-acting effect, coupled with the fact that **71** will not carry a charge at the digestive vacuole pH, which is purportedly necessary for drug accumulation, suggests that the compound might act via a different mechanism of action [65]. The poor water solubility of **71** might also limit its viability as an anti-malarial lead.

### Terpenylindoles

**Fig. 12:**
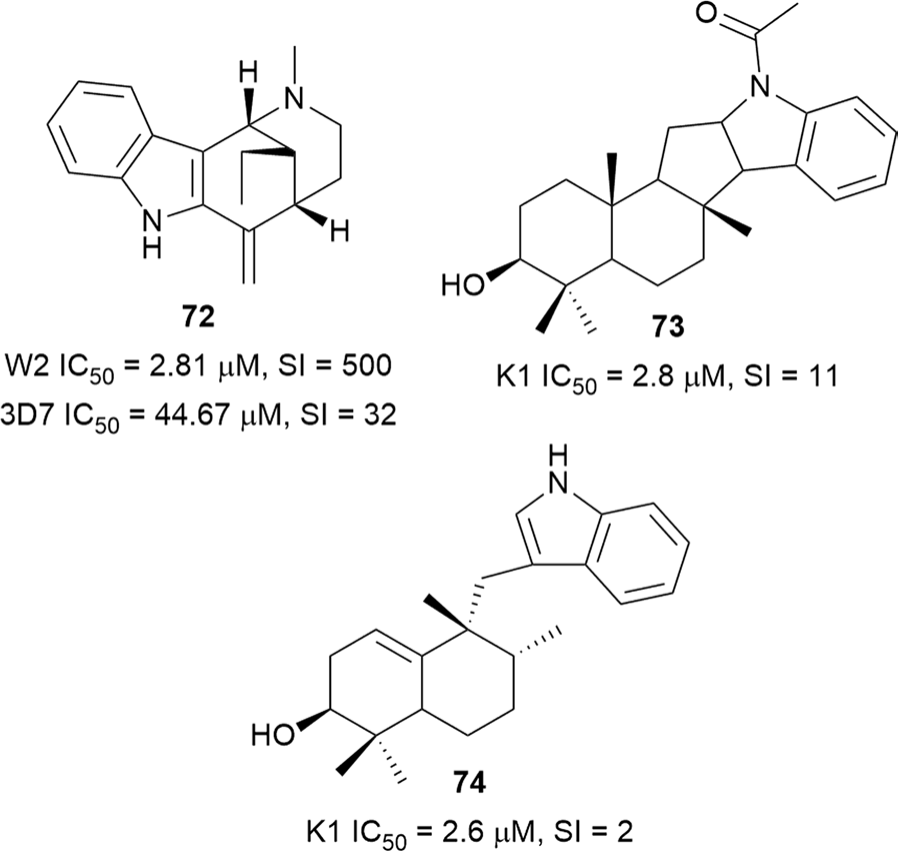
Structures of terpenyl alkaloids

The monoterpenoid indole alkaloid uleine (**72**) (Fig. 12) was isolated as the major antiplasmodial alkaloid from the trunk bark of the Brasilian tree *Aspidosperma parvifolium* (Apocynaceae) and was more active against the W2 than the 3D7 strain, with low cytotoxicity against the Hep G2A16 and Vero cell lines [66]. Compound **72** was found to localize in the parasite digestive vacuole as a result of the presence of a basic aliphatic amino group, which undergoes protonation in the acidic digestive vacuole and accumulates in suitable concentrations to inhibit heme polymerization [67]. Uleine has also been isolated from *Aspidosperma olivaceum* [68]. Traditionally, the Nkundo people in the DR Congo use various parts of *Greenwayodendron suaveolens* (Annonaceae) to treat malaria. Some species of monkeys chew the bitter leaves, presumably for zoopharmacognostic purposes [69]. Two sesquiterpenyl indole alkaloids, *N*-acetylpolyveoline (**73**) and polyalthenol (**74**) (Fig. 12), isolated from the root bark of *Greenwayodendron suaveolens*, are active against the K1 strain. While polyathenol was found to be cytotoxic against MRC-5 cells, **73** was more selective (SI = 10.6) [69].

### Bisindoles and related indoles

**Fig. 13:**
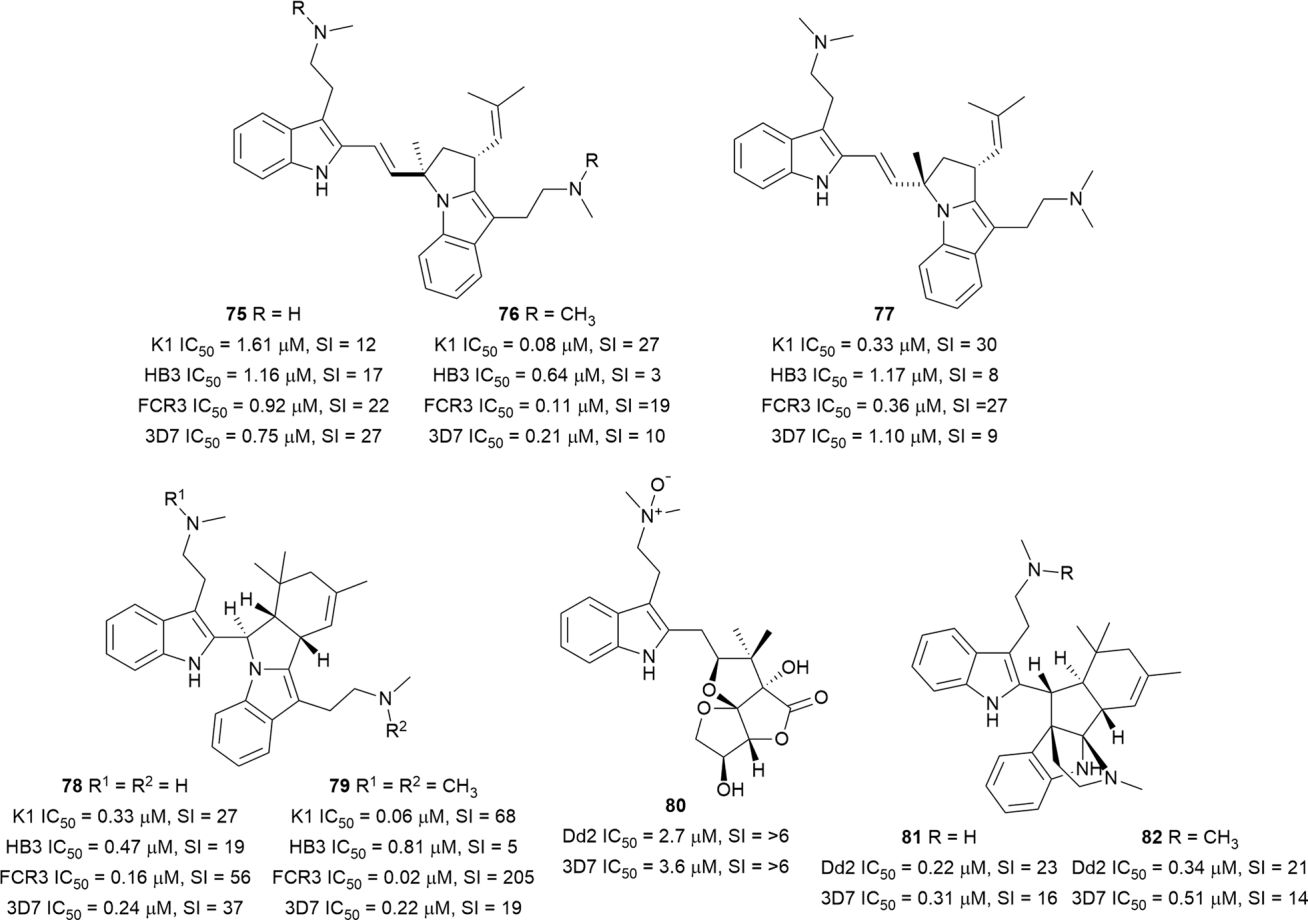
Structures of bisindole alkaloids **75**–**82**

Flinderole A (**75**) and isoborreverine (**78**) were isolated from the bark of the Australian tree *Flindersia acuminata* (Rutaceae) and flinderoles B (**76**) and C (**77**), and dimethylisoborreverine (**79**) from *Flindersia amboinensis* from Papua New Guinea [56]. These indole alkaloids (Fig. 13) exhibited selective antiplasmodial activity against a wide panel of drug-sensitive and -resistant parasites. In a further investigation to ascertain which stage of the development cycle of the parasite is affected by the most active compound **79**, it was observed that **79** was more active against *P. falciparum* trophozoites, with treated parasites showing changes in digestive vacuole morphology and a reduced formation of haemozoin [46, 70]. A different *Flindersia* species, *Flindersia pimenteliana*, was the source of the new pimentelamine C (**80**), the known borreverine (**81**), and 4-methylborreverine (**82**), which were reported to be active against *P. falciparum* 3D7 and Dd2 with low toxicity to HEK-293 cells [71]. Compound **80**, which was isolated as the trifluoroacetate salt from the plant leaves, is one of three new indole alkaloids incorporating an ascorbic acid moiety. Interestingly, the other two analogues without a polar *N*-oxide moiety on the ethylamine unit attached to the indoleskeleton, were inactive [71]. This suggests a SAR role for the ethylamine unit that could be further explored.

**Fig. 14:**
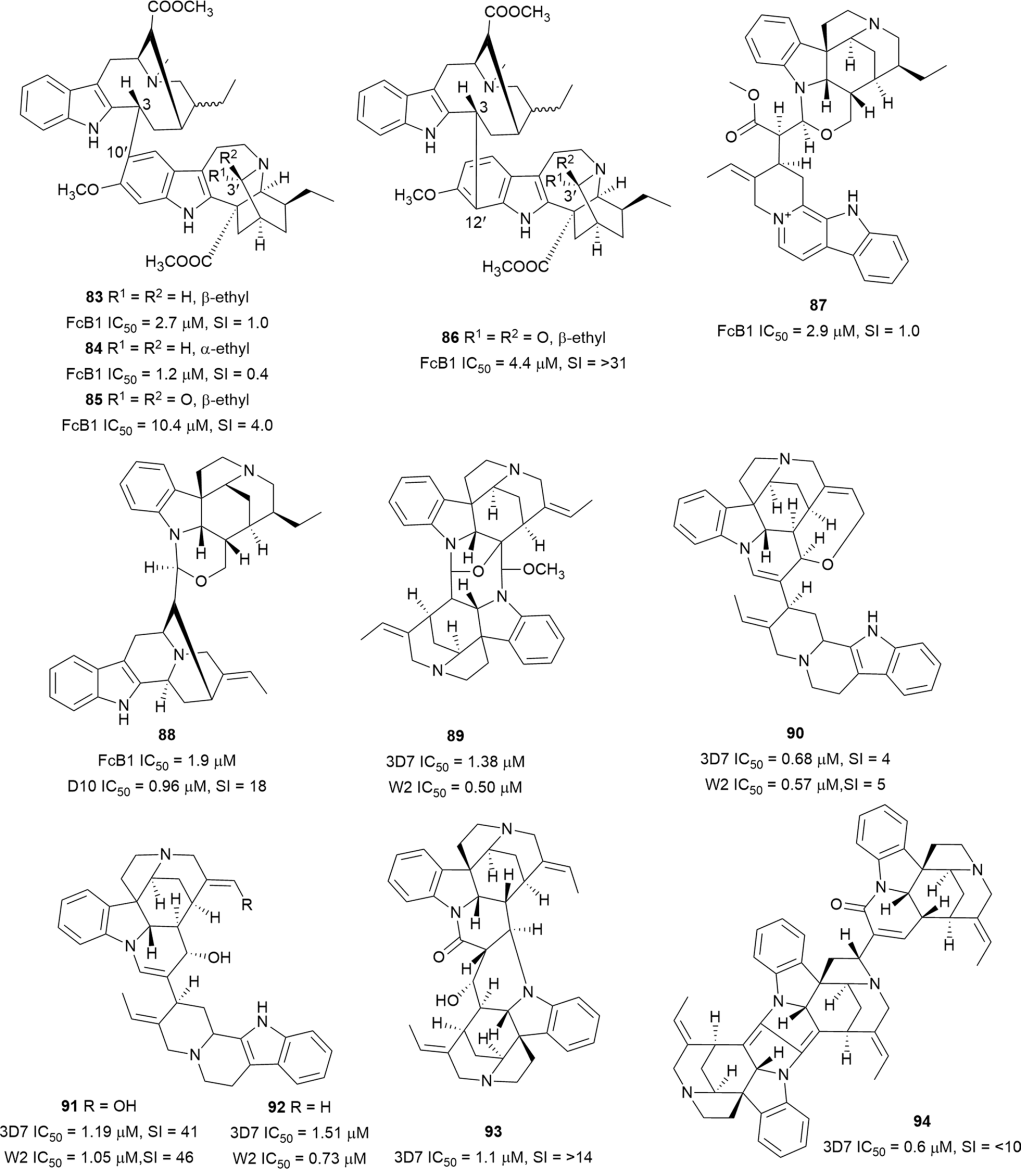
Structures of bisindole alkaloids **83**–**94**

Two bisindole alkaloids with a vobasinyl-iboga skeleton tabernaelegantine B (**83**) and D (**84**) (Fig. 14), isolated from *Muntafara sessilifolia* (Apocynaceae) stem bark, inhibited the FcB1 plasmodial strain but these compounds were also cytotoxic against L6 and MRC-5 cells [72]. However, the C-3′ oxidized analogues **85** and **86**, in particular, showed better selectivity towards the parasite suggesting the position of the linkage between the monomeric units might influence the bioactivities of these compounds [72]. This observation is supported by the nanomolar antiplasmodial activity of another vobasinyl-iboga alkaloid, voacamine, which also has a C-3/C-11′ linkage [73]. A reinvestigation of *Geissospermum laeve* (Apocynaceae) using a dereplication strategy led to the isolation of new bisindole alkaloids, 3′,4′,5′,6′-tetradehydrogeissospermine (**87**) and geissolosimine (**88**), from the bark of the tree [74]. Compound **88** has also been isolated from the bark of *Geissospermum vellosii* [75]. The compounds exhibited non-selective antiplasmodial activity against FcB1 parasites [74]. Moreover, **88** was also active against the D10 strain with low toxicity to Chinese hamster ovarian cells [75]. Divarine (**89**), longicaudatine (**90**), longicaudatine F (**91**), and longicaudatine Y (**92**) from the stem bark of *Strychnos malacoclados* (Loganiaceae) inhibited the growth of the 3D7 and W2 parasite strains. A cytotoxicity assay on WI-38 human fibroblasts with the most active **90** showed that the antiplasmodial activity is not specific. However, the structurally similar longicaudatine F (**91**), possessing an open ring in place of the six-membered oxygen heterocycle in **90**, was 40 times more selective against the parasite, despite the slightly lower antiplasmodial activity [76]. A phytochemical investigation of *Strychnos icaja* root provided a new bisindole, strychnobaillonine (**93**), and the known strychnohexamine (**94**) (Fig. 14). The alkaloids were active against the 3D7 strain of *P. falciparum*, and the trisindole **94** was also cytotoxic against WI-38 cells (SI < 10), whereas **93** was not cytotoxic at the highest tested concentration (10 µg/mL) [77]. Interestingly, while monomers of *Strychnos* alkaloids do not have antiplasmodial activity, polymerization increases the basic nature of the monomers and confers antiplasmodial potency. This suggests that some degree of basicity is essential for antiparasitic activity of the *Strychnos* alkaloids and that compounds might localize in the parasite acidic digestive vacuole [78].

**Fig. 15:**
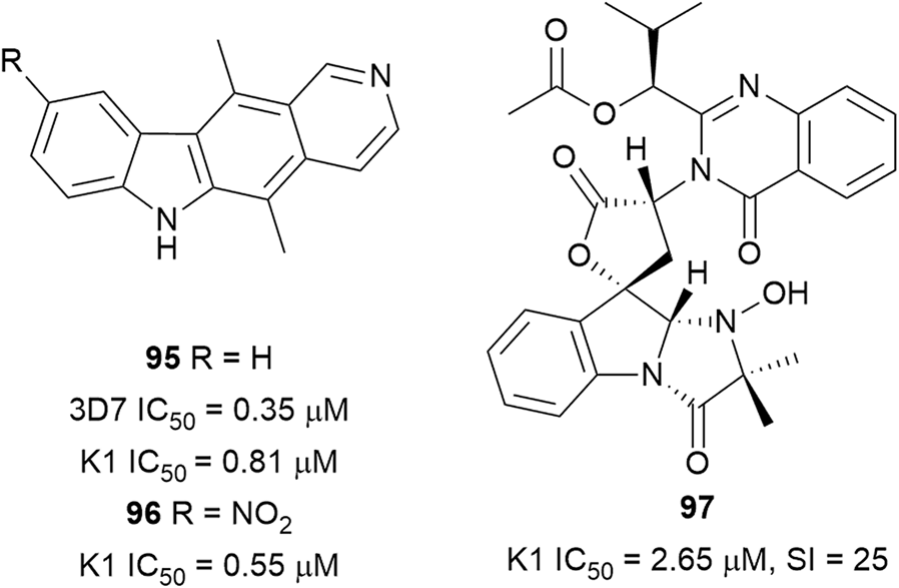
Structures of indole derivatives **95**–**97**

It was previously reported that ellipticine (**95**) (Fig. 15), isolated from *Aspidosperma vargasii* (Apocynaceae) bark, has in vitro and in vivo anti-malarial activity [79]. In search of more active analogues, semi-synthetic derivatives of **95** were prepared. 9-Nitroellipticine (**96**), in which ring A was modified, was more active than **95**, while the 7-nitro derivative was the least active [80]. The indole-quinazoline alkaloid tryptoquivaline (**97**), obtained from the culture broth of *Neosartorya spinosa* KKU-1NK1 (sexual state of *Aspergillus* fungus species), was active against K1 parasites. The compound was slightly cytotoxic to Vero cells (SI = 25) but was inactive against a panel of cancer cell lines [81].

### β-Carbolines

**Fig. 16:**
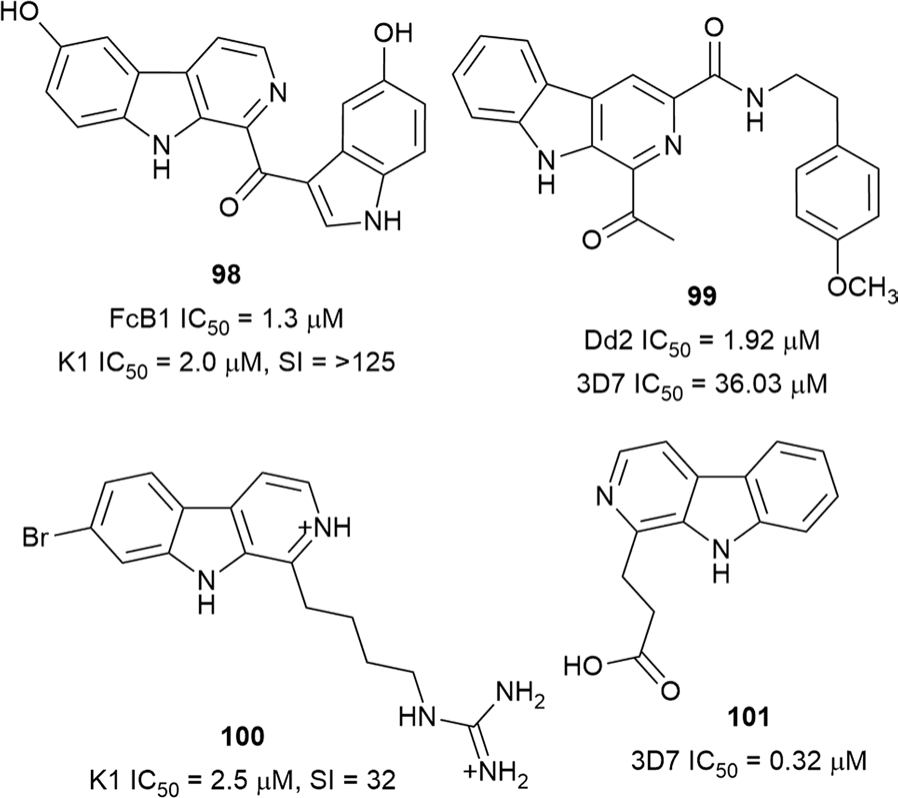
Structures of β-carbolines

The β-carboline-indole alkaloid hyrtiosulawesine (**98**) (Fig. 16), isolated from the root of *Aristolochia cordigera* (Aristolochiaceae), inhibited the in vitro viability of *P. falciparum* (3D7) without any toxicity to Hep G2 cells. Similar inhibition of the FcB1 strain was observed with synthetic **98** [82]. However, the glucoside derivative of **98** was only half as active as **98** [83]. Marinacarboline A (**99**) isolated from *Marinactinospora thermotolerans* SCSIO 00652, an actinomycetes species from South China Sea marine sediments, was 18 times more active against the multi-drug resistant Dd2 strain than against the chloroquine-sensitive 3D7. The compound was not significantly cytotoxic to a panel of tumour cell lines (IC_50_ > 50 µM) [84]. The New Zealand ascidian *Pseudodistoma opacum* was the source of a new antiplasmodial alkylguanidine-substituted β-carboline alkaloid, opacalin A (**100**). The poor cytotoxicity of **100** against L6 cells indicates a selective antiparasitic activity against the K1 strain [85]. β-Carboline-1-propionic acid (**101**) was isolated from the root of *Eurycoma longifolia* (Simaroubaceae), a popular southeast Asian medicinal plant. It exhibited antiplasmodial activity against the 3D7 strain, but the cytotoxicity was not reported [86].

### Piperidine, pyridone, and pyrimidine alkaloids

**Fig. 17:**
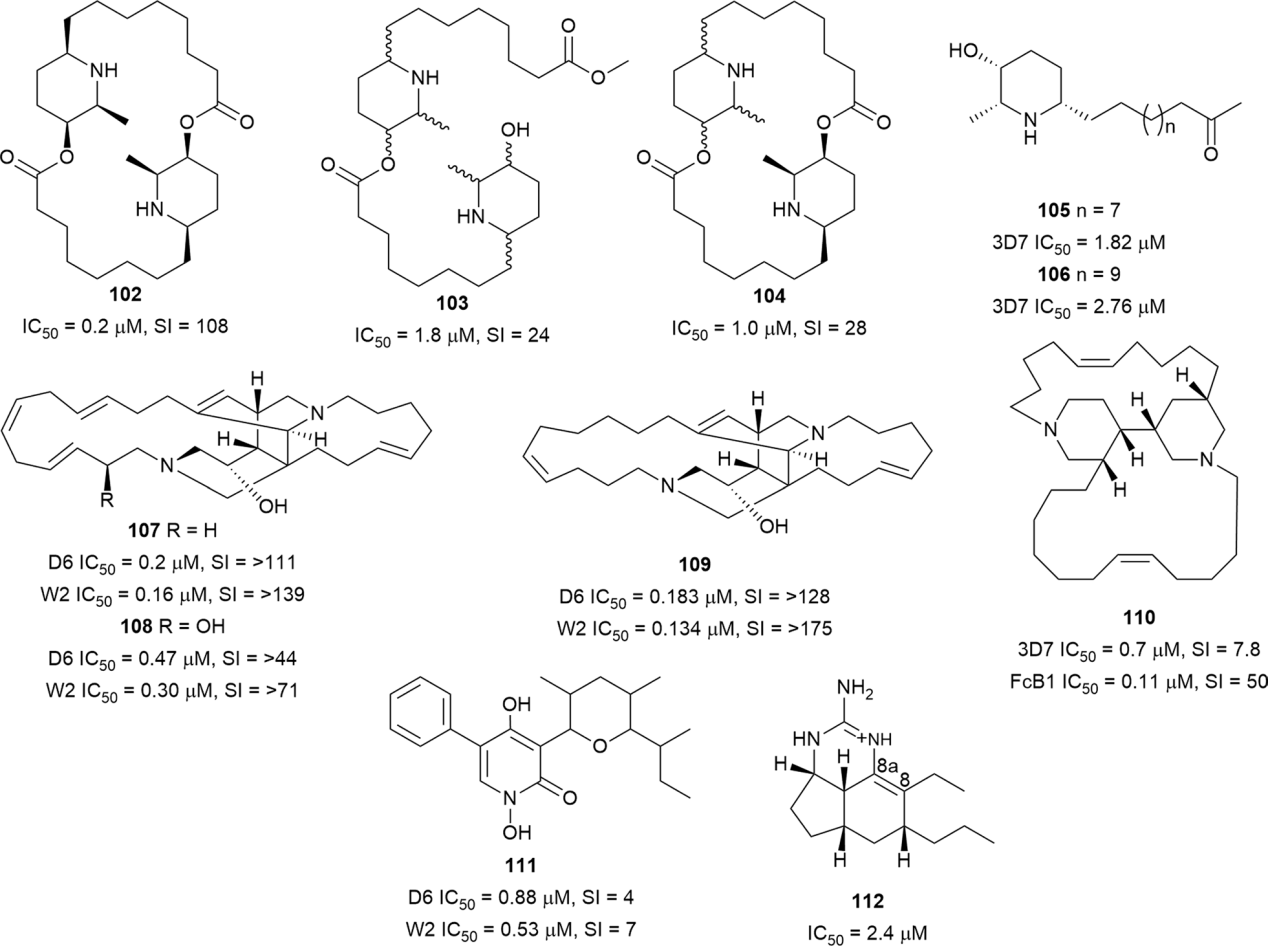
Structures of piperidines **102**–**110**, pyridone **111**, and pyrimidine alkaloid **112**

The leaf decoction of *Carica papaya* (Caricaceae), which is traditionally used to treat malaria in Indonesian Papua and Maluku islands, displayed in vitro antiplasmodial activity (51% inhibition at 4.8 µg/mL) [87]. Purification of an alkaloid-enriched fraction of the plant leaves yielded the dimeric piperidine alkaloids **102**–**104** (Fig. 17) as the antiplasmodial principles, while the monomeric carpamic acid and methyl carpamate were inactive. Carpine (**102**), with the best activity, was the major alkaloid, but it was inactive in vivo (11.9% suppression of *P. berghei* parasite at 5 mg/kg). The in vivo activity of a hydroalcoholic extract of papaya leaves suggests that other metabolites in the extracts might potentiate the antiplasmodial activity of the active compounds [87]. The observed lack of in vivo efficacy of **102** highlights the complex relationship between metabolites in natural extracts and emphasizes the need to validate in vitro potencies in animal models. Nevertheless, potent and selective activity of compounds can be exploited by medicinal chemistry methods in designing improved analogues. Cassine (**105**) and spectaline (**106**) from *Senna spectabilis* leaf were active against 3D7 *P. falciparum* in vitro. However, the 3-*O*-acetyl semi-synthetic derivatives were less active than the natural piperidine parents [88]. Ingamine A (**107**), together with two new ingamine-type piperidine alkaloids, (22*S*)-hydroxyingamine A (**108**) and dihydroingenamine D (**109**), were isolated from the marine sponge *Petrosid Ng5 Sp5*. Compounds **107**–**109** showed sub-micromolar antiplasmodial activity against D6 and W2 parasites without cytotoxicity against cancerous and noncancerous cells at 10 µg/mL [89]. An antiplasmodial high-throughput screen of the ethanolic extract of marine sponges from the Solomon Islands identified the active *Heliclona* sp. with an in vitro activity of < 1 µg/mL. Bioassay-guided fractionation of the extract from this sponge led to the isolation of haliclonacyclamine A (**110**). The bis-piperidine **110** was more active against chloroquine-resistant FcB1 than chloroquine-sensitive 3D7 parasites, with low cytotoxicity against MCF7 cancer cells. It suppressed parasitaemia in *Plasmodium vinckei petteri*-infected mice by 45% after 4 days of treatment with 10 mg/kg/day [90]. Most of the antiplasmodial piperidine alkaloids mentioned here are cyclic dimers. The potent, selective activity of these compounds makes them attractive as lead templates in anti-malarial drug design.

The new pyridone alkaloid **111** (Fig. 17), with a 1,4-dihydroxy-5-phenyl-2-pyridinone skeleton, was isolated from the Ascomycetes fungus, *Septoria pistaciarum.* Compound **111** was active against *P. falciparum* D6 and W2 strains, but it was also cytotoxic against Vero cells. Three other analogues without a free *N*-hydroxy group on the pyridone heterocycle were inactive, suggesting a SAR role for substituents on the ring nitrogen [91].

The extract of *Biemna laboutei*, a marine sponge collected at Salary Bay in Madagascar, showed antiplasmodial activity with IC_50_ of 3.2 µg/mL. Chemical investigation of the active extract yielded new tricyclic pyrimidine alkaloids named netamines. Among the isolated alkaloids, netamine K (**112**), with a Δ^8,8a^-double bond in the tricyclic skeleton, exhibited activity against *P. falciparum* without toxicity to KB cells at the highest tested concentration of 1 µM [92].

### Pyrroles

**Fig. 18:**
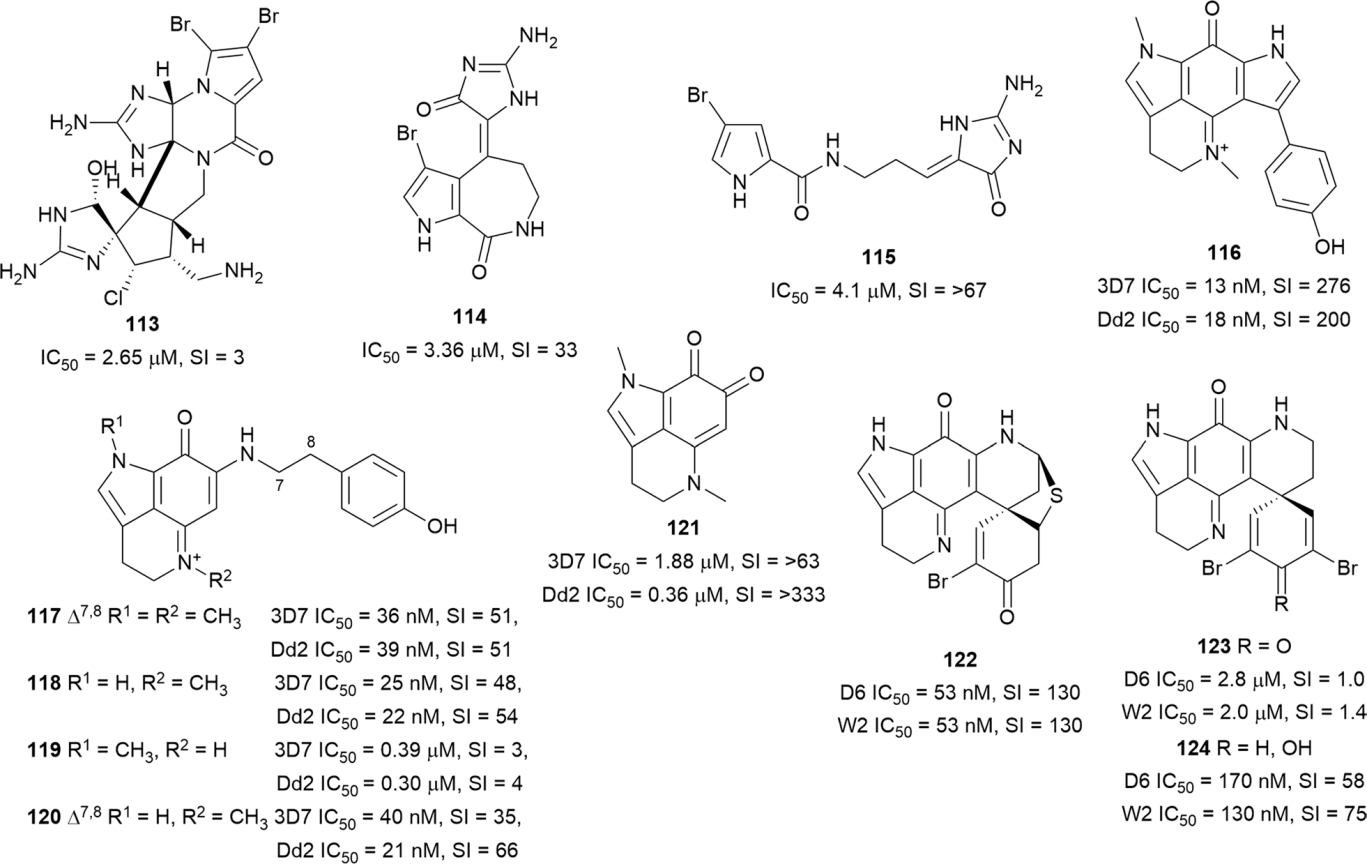
Structures of pyrrole alkaloids

A series of 14 structurally related bromopyrrole alkaloids (Fig. 18) derived from sponges of the *Agelas* and *Axinella* genera were assayed for antiplasmodial activity against the K1 strain. Dibromopalau’amine (**113**) had the highest activity against the parasite, although it was also cytotoxic against L6 cells. The slightly less potent spongiadicin B (**114**) and dispacamide B (**115**) were more selective against the parasite (SI = 32.7 and > 67.2, respectively). Preliminary SAR observations in this series indicated that the aminoimidazole ring and the level of oxidation are important for antiplasmodial activity. Analogues lacking the imidazole ring were inactive while those in which the ring is not oxidized were less active. Some bromopyrrole alkaloids inhibited *Plasmodium* type II fatty acid synthase (FAS II) enzyme, suggesting that this might be part of the mechanism of action [93]. The new bispyrroloiminoquinone tsitsikammamine C (**116**), with nanomolar antiplasmodial activity against 3D7 and Dd2 *P. falciparum* strains, together with the equally active makaluvamines G (**117**), J–L (**118**–**120**), were isolated from the marine sponge *Zyzzya* sp. [94]. Compounds **116** and **118** were equally active against both parasite schizonts and trophozoites. Subcutaneous treatment of *P. berghei* infected mice with **117** at 8 mg/kg/day for 4 days suppressed parasitaemia by 48% with no apparent toxicity to mice. Damirone A (**121**), with a benzoquinone group in place of the iminoquinone moiety of the makaluvamines, was less active, suggesting that the iminoquinone group is crucial for activity. Methylation of the iminium nitrogen led to an increase in activity [94]. Bioassay- and LC–MS-guided fractionation of an active extract from the Alaskan-sourced *Latrunculia* sp. sponge yielded discorhabdins A (**122**), and C (**123**), and dihydrodiscorhabdin C (**124**) [95]. The most potent pyrroloiminoquinones, **122** and **124**, with nanomolar antiplasmodial activity against D6 and W2 strains, were also the most selective (SI = 130 and 75, respectively). In an in vivo experiment, *P. berghei*-infected mice were treated with **122** and **124** at 10 mg/kg/day and although 50% suppression of parasitaemia was observed with **122** after 2 days of treatment, both compounds resulted in symptoms of severe toxicity [95].

### Other alkaloids

**Fig. 19:**
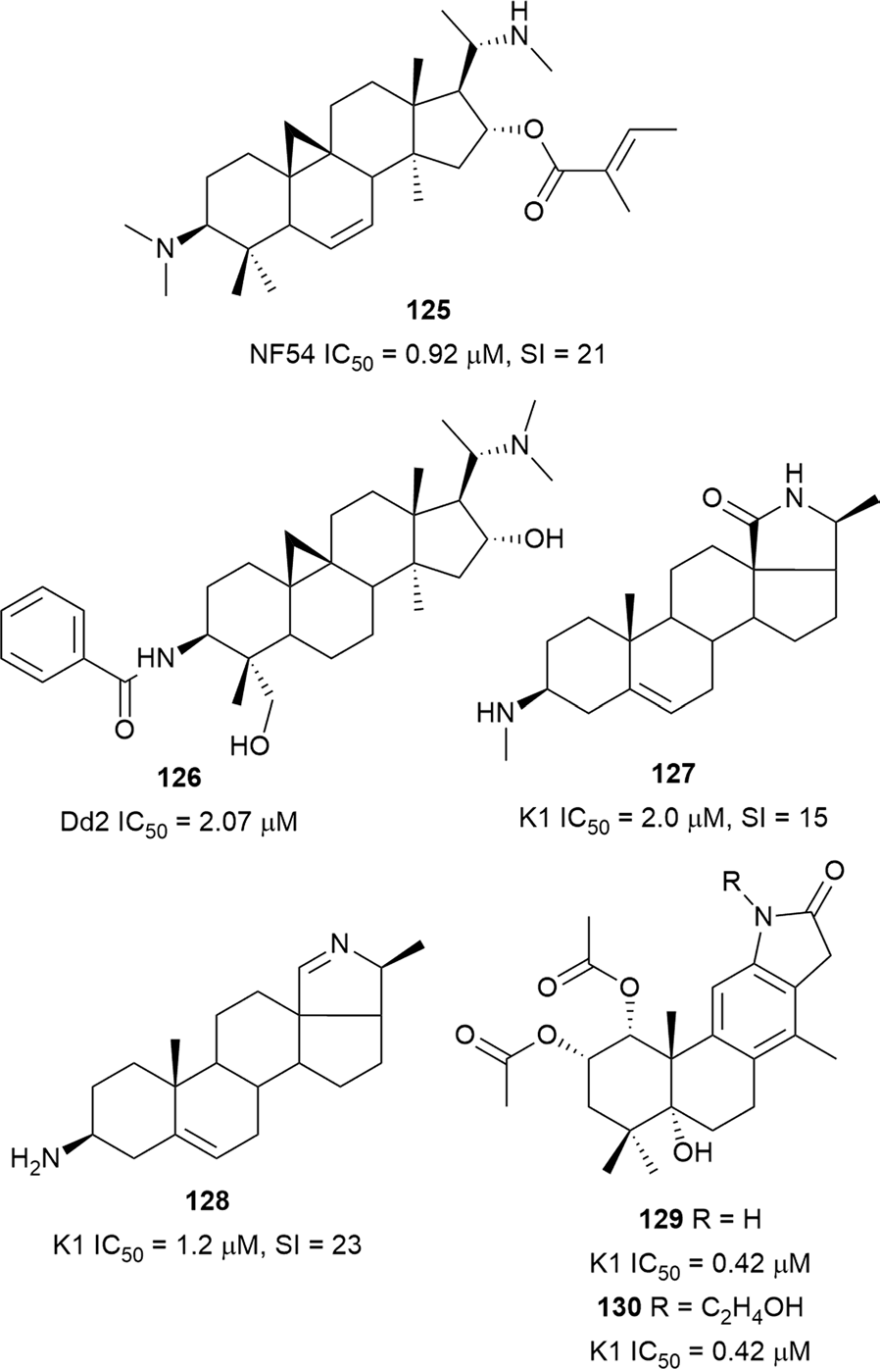
Structures of steroidal alkaloids

Concoctions prepared from *Buxus* plant species are used for the treatment of malaria in various traditional medicine systems [96, 97]. An alkaloid-enriched fraction from the leaves of *Buxus sempervirens* (Buxaceae), which exhibited selective antiplasmodial activity (IC_50_ = 0.36 µg/mL, SI = 20.3), was subjected to bioassay-guided fractionation and yielded the cycloartane alkaloid, *O*-tigloylcyclovirobuxeine B (**125**) (Fig. 19), as the major antiplasmodial compound. The antiplasmodial activity of **125** against the NF54 strain was slightly less than that of the crude alkaloid fraction, but the compound was not significantly cytotoxic against L6 cells. Compound **125** was also detected in significant quantities in a leaf decoction of *Buxus sempervirens* that was prepared in accordance with ethnobotanical protocols [98]. Purification of the chloroform fraction of an extract from the combined twigs, leaves, and fruits of *Buxus cochinchinensis* yielded a number of bioactive triterpenoids, including the cycloartane alkaloid, *N*-benzoyldihydrocyclomicrophylline F (**126**). This compound inhibited both Dd2 *P. falciparum* and HT-29 human colon cancer cells, suggesting non-selective activity [99]. Two pregnane-type steroidal alkaloids, mokluangin A (**127**) and irehline (**128**), isolated from the root of *Holarrhena pubescens* (Apocynaceae), were active against K1 *P. falciparum* with low cytotoxicity against NCI-H187 lung cancer cells (IC_50_ = 30.6 and 27.7 µM, respectively) [100]. Two new cassane diterpene alkaloids, caesalminines A (**129**) and B (**130**) (Fig. 19), possessing a tetracyclic furanoditerpenoid skeleton were isolated from the seeds of *Caesalpinia minax* (Fabaceae). The compounds, which were proposed to be biosynthetically derived from the aminolysis of the geranylgeranyl pyrophosphate precursor, inhibited K1 *P. falciparum* [101].

**Fig. 20:**
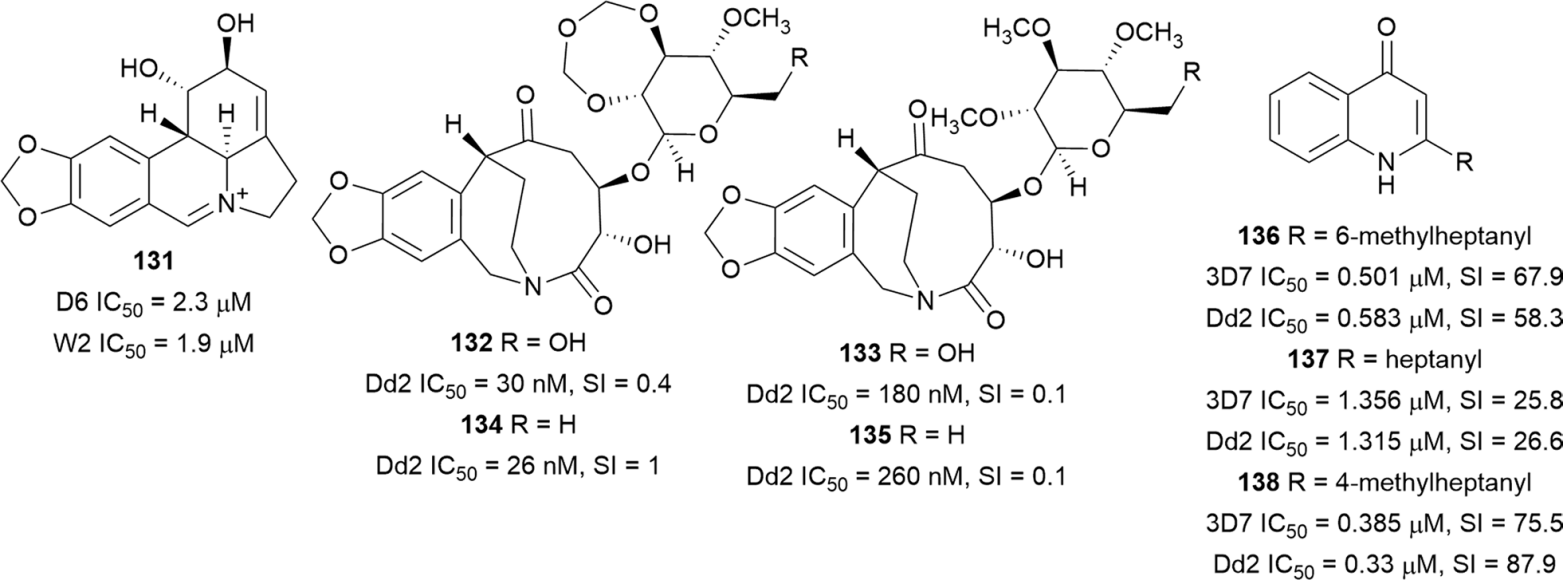
Structures of (+)-5,6-dehydrolycorine **131**, lactams **132**–**135** and quinolinone alkaloids **136**–**138**

A new Amaryllidaceae alkaloid, (+)-5,6-dehydrolycorine (**131**) (Fig. 20), isolated from the bulbs of *Lycoris radiata* (Amaryllidaceae), inhibited the in vitro viability of *P. falciparum* (D6 and W2), albeit with associated cytotoxicity against eight human tumour cell lines, indicating non-specific antiparasitic activity [102]. Similarly, the macrocyclic lactams cripowellin A–D (**132**–**135**) (Fig. 20), which were isolated from the alkaloid-enriched extract of *Crinum erubescens* (Amaryllidaceae), inhibited the Dd2 strain with nanomolar IC_50_ values but were also cytotoxic against cancerous A2780 cells. Importantly, the presence of the 1,3,5-trioxepane-ring in **132** and **134** correlated with improved activity [103]. Another bioassay-guided purification, this time of *Crinim firmifolium* leaf extract, led to the isolation of the new 2-alkylquinolinones **136** and the known **137**, which were both active against the 3D7 and Dd2 strains with mild cytotoxicity against A2780 mammalian ovarian cancer cells [104]. Incorporation of a branched alkyl into **137** to form **138** improved antiplasmodial activity, suggesting that branching of the alkyl side chain is beneficial to potency [104].

**Fig. 21:**
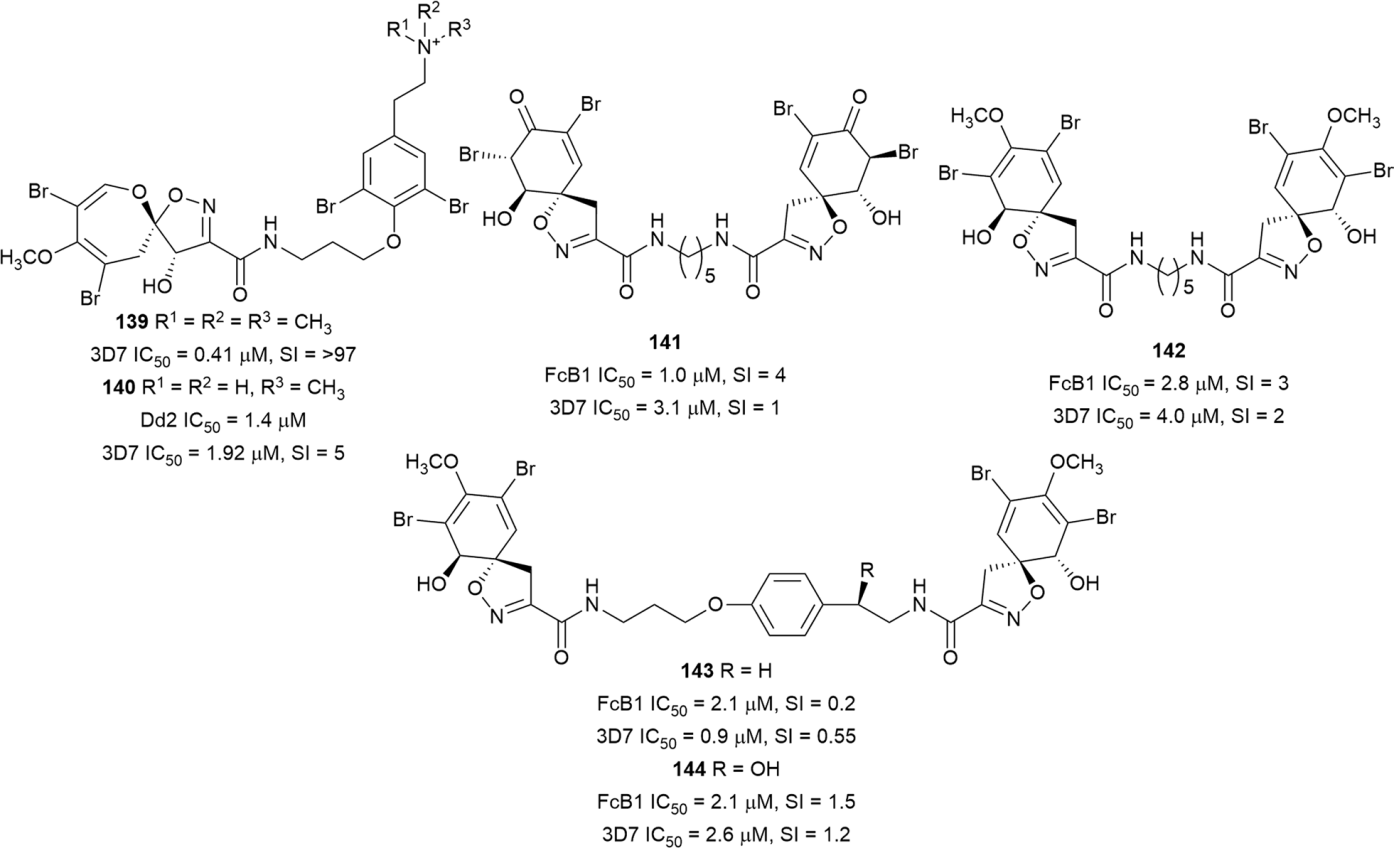
Structures of bromotyrosine alkaloids

The bromotyrosine alkaloid psammaplysin H (**139**) was isolated from a marine *Pseudoceratina* sponge together with the known psammaplysin F (**140**), previously isolated from a *Hyattella* sponge, and both exhibited antiplasmodial activity against the 3D7 strain (Fig. 21) [105]. Psammaplysin H (**139**), which has a trimethylated quaternary terminal nitrogen, was not toxic to HEK293 and HepG2 mammalian cells, while **140** suffered from reduced activity coupled with significant cytotoxicity [106]. Preliminary SAR studies indicated that substitution on the terminal nitrogen influences the selective antiplasmodial activity. Four other antiplasmodial bromotyrosine derivatives, aplysinone D (**141**), homoaerothionin (**142**), 11,19-dideoxyfistularin 3 (**143**), and 11-hydroxyfistularin (**144**), were isolated from *Suberea ianthelliformis*, a marine sponge from the Solomon Islands. Unfortunately, these compounds were also cytotoxic against Vero cells [107].

**Fig. 22:**
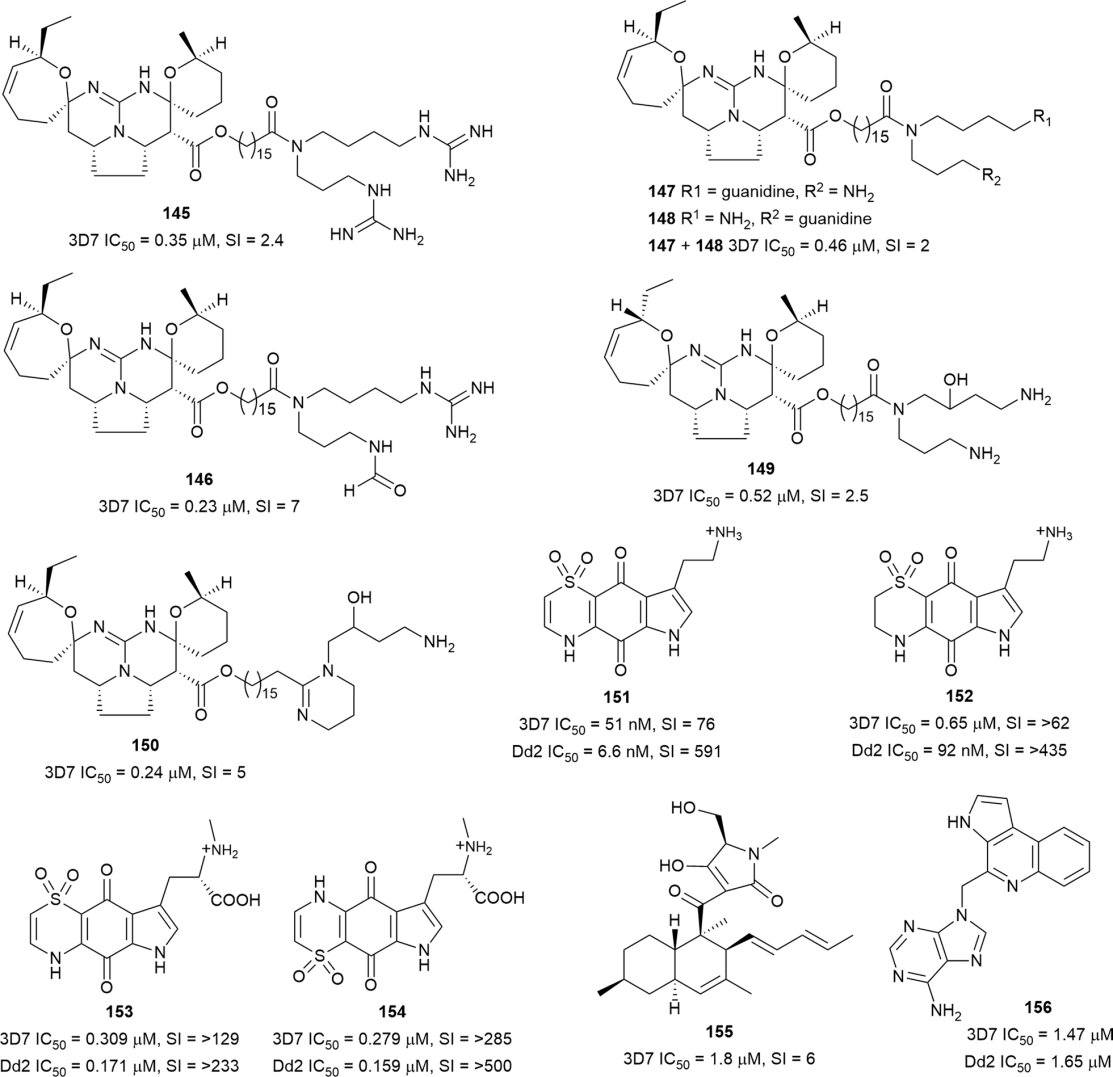
Structures of guanidines **145**–**150**, thiazines **151**–**154**, phomasetin **155** and aplidiopsamine A **156**

Interrogation of the marine sponge *Monanchora unguiculata*, collected at the Mitsio islands of Madagascar, yielded four new guanidine alkaloids ptilomycalins E–H (**145**–**148**), along with the known crambescidin 800 (**149**) and fromiamycalin (**150**) (Fig. 22). The compounds exhibited sub-micromolar antiplasmodial activity against the 3D7 strain but were also cytotoxic against KB cells [108]. Similarly, four new antiplasmodial tricyclic thiazine alkaloids thiaplakortones A–D (**151**–**154**) (Fig. 22), isolated from the Australian marine sponge *Plakortis lita*, showed nanomolar inhibition against the 3D7 and Dd2 parasites, with low toxicity to human HEK293 cells (SI = > 62 − > 500) [109]. The decalin-tetramic acid metabolite phomasetin (**155**) was obtained following the culturing of the *Pyrenochaetopsis* sp. RK10-F058 fungus. Biological assessment revealed that **155** was active against *P. falciparum* 3D7 with moderate cytotoxicity against cancerous HeLa, HL-60, and src^ts^-NRK cells. The same culture broth yielded two more decalin metabolites, possessing a cyclopentanone-fused decalin skeleton and a serine-derived *N*-methylated amino acid instead of the tetramic acid moiety. However, they were > 21 times less active than **155**, indicating that the cyclized tetramic acid group might be crucial for potent activity [110]. Aplidiopsamine A (**156**) (Fig. 22), with a rare pyrrolo-quinoline conjugated to an adenine nucleobase, was isolated from the Australian ascidian *Aplidiopsis confluata.* The new metabolite was active against the 3D7 and Dd2 strains without significant toxicity against HEK-293 cells [111].

**Fig. 23:**
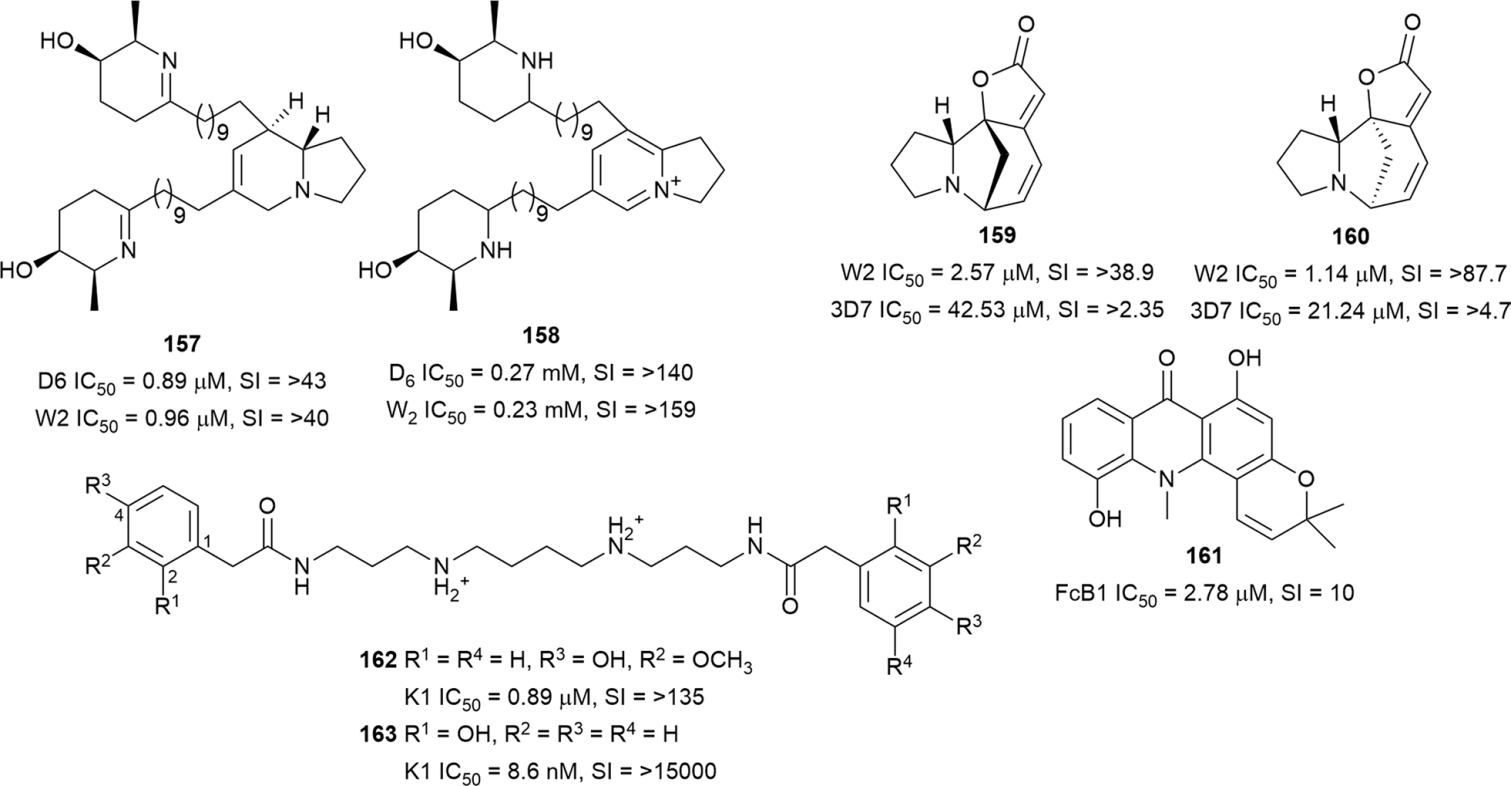
Structures of other alkaloids **157**–**163**

An extract from the leaves of *Prosopis glandulosa* (Fabaceae), collected in Nevada, yielded a new tertiary indolizidine alkaloid Δ^1,6^-juliprosopine (**157**) (Fig. 23). Interestingly, the leaf extract of the same plant collected in Texas produced the known quaternary alkaloid juliprosine (**158**) but not **157**. The two compounds inhibited the D6 and W2 strains without any toxicity to Vero cells at the highest tested concentration of 23.8 µg/mL [112]. Allonorsecurinine (**159**), previously reported as a synthetic compound, was isolated with *ent*-norsecurinine (**160**) from the antiplasmodial plant *Phyllanthus fraternus* (Phyllanthaceae) (methanol extract IC_50_ = 0.44 µg/mL against 3D7) [113]. The two securinega alkaloids were more active against chloroquine-resistant W2 than against chloroquine-sensitive 3D7 parasites. No cytotoxicity was observed against human umbilical vein endothelial cells at the highest concentration of 100 µM [114]. The root bark extract of the Ugandan anti-malarial medicinal plant *Citropsis articulata* (Rutaceae) displayed 77% inhibition of FcB1 *P. falciparum* at 10 µg/mL with low cytotoxicity against Vero cells. A pyranoacridone alkaloid, 5-hydroxynoracronycine (**161**), was isolated as the most active constituent against the same parasite strain. However, the compound was also moderately cytotoxic against Vero cells (SI = 10) [115].

The polyamine diamide orthidine F (**162**) (Fig. 23) from the New Zealand-sourced ascidian *Aplidium orthium* was active against K1 *P. falciparum* without cytotoxicity against L6 cells. A synthetic 2-hydroxyphenylacetamide derivative **163** was > 100 times more active while retaining selectivity. Preliminary SAR indicated that the two arylamide terminals and the spermine fragment are essential for antiplasmodial activity. Similarly, the hydroxy group at C-2 of the aromatic rings is important for improved antiplasmodial activity [116].

## Terpenes

Among the 447 isolated natural products with IC_50_ ≤ 3.0 µM reported in this review, 30.8% are terpenoids.

### Monoterpenes

**Fig. 24:**
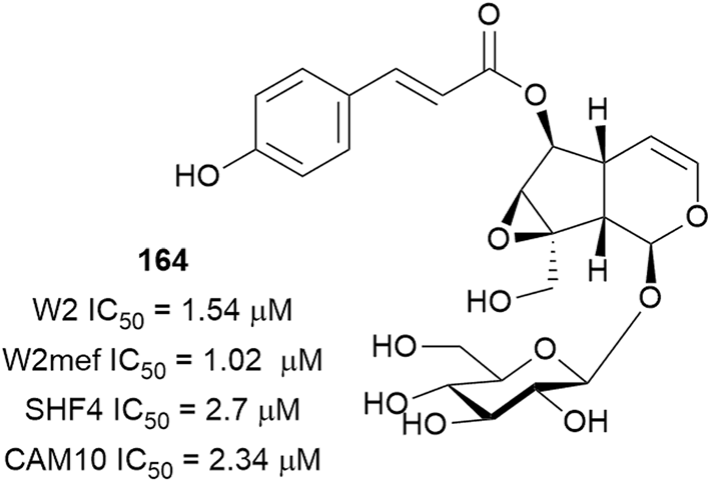
Structure of specioside

The iridoid specicoside (**164**) (Fig. 24), isolated from an antiplasmodial ethyl acetate extract of *Kigelia africana* (Bignoniaceae), was active against the *P. falciparum* W2, CAM10 and SHF4 strains without cytotoxicity against LLC/MK-2 cells. Specicoside (**164**) acted in synergy with artemether in inhibiting the W2mef strain but had an antagonistic effect with *p*-hydroxycinnamic acid, which is also present in *Kigelia africana* [117, 118].

### Sesquiterpenes

**Fig. 25:**
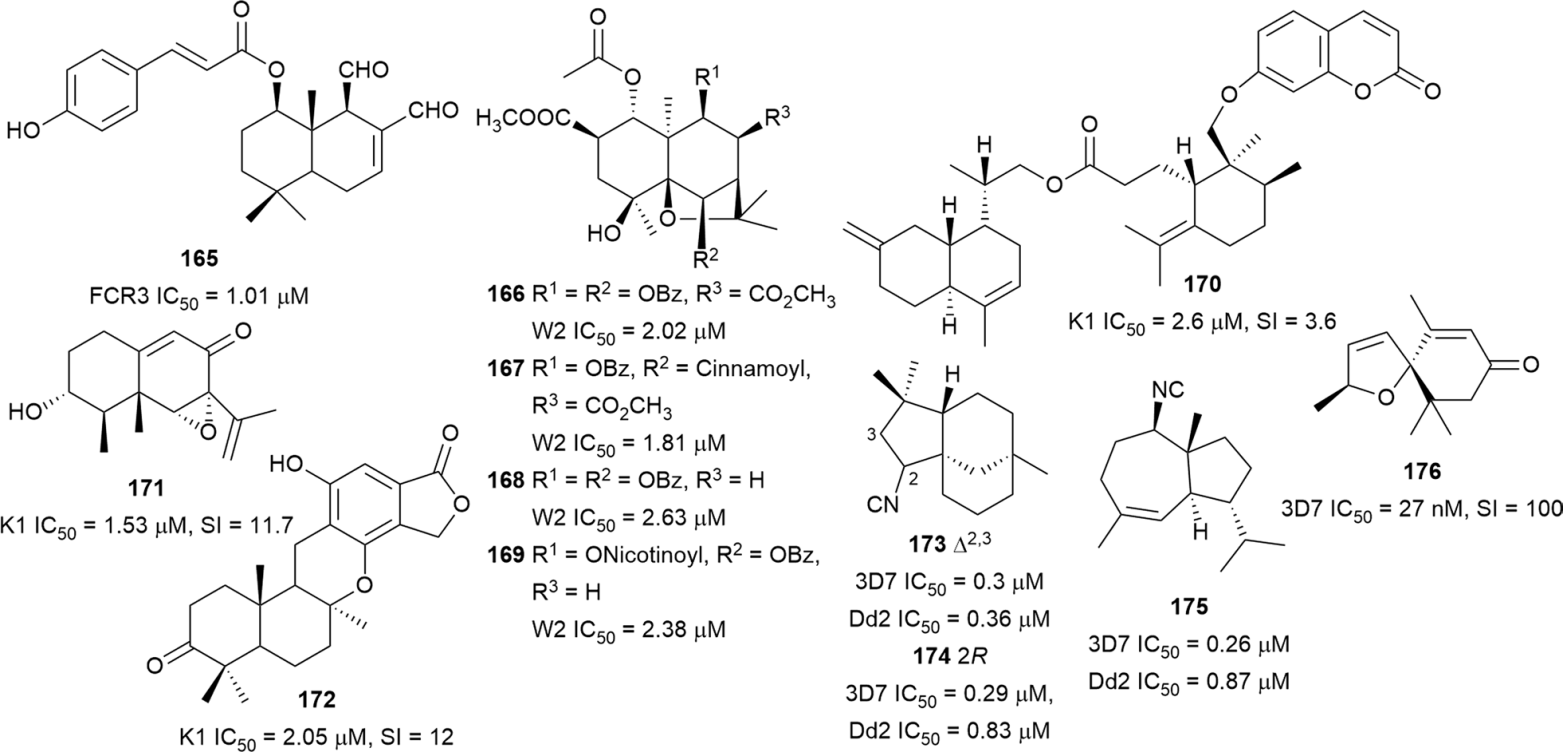
Structures of sesquiterpenes **165**–**176**

A phytochemical investigation of the active chloroform extract of *Drimys brasiliensis* (Winteraceae) stem bark (*P. falciparum* FcR3, IC_50_ 3.0 µg/mL) led to the isolation of drimane sesquiterpenes but the most active compound was 1β-(*p*-coumaroyloxy)polygodial (**165**) (Fig. 25) [119]. Preliminary antiplasmodial screening of *Salacia longipes* var*. camerunensis* (Celastraceae) seed extract showed that it was active against the W2 strain with an IC_50_ of 2.28 µg/mL. Extensive purification of the active extract afforded the β-agarofuran sesquiterpenoids salaterpenes A–D (**166**–**169**) (Fig. 25), which were also active against W2 parasites [120]. The root extract of *Ferula pseudalliacea* (Apiaceae) yielded an antiplasmodial metabolite sanandajin (**170**), the first isolated disesquiterpene-coumarin. This compound, a cadinenyl ester of the sesquiterpene coumarin glabanic acid, inhibited the K1 parasite and had moderate cytotoxicity against L6 cells [121].

The eremophilane sesquiterpenoid sporogen-AO1 (**171**), produced by the soil fungus *Penicillium copticola* PSURSPG138, inhibited K1 parasites but was also cytotoxic against human oral epidermoid carcinoma (KB) and Vero cells [122]. The endophytic fungus *Phomopsis archeri*, isolated from *Vanilla albidia* cortex stem, yielded an extract with antiplasmodial activity (IC_50_ = 5.0 μg/mL) from which an aromatic sesquiterpene phomoarcherin B (**172**) (Fig. 25), with antiplasmodial activity but also moderate cytotoxicity against cholangiocarcinoma and KB cells, was isolated [123]. The Australian nudibranch *Phyllidia ocellata* has produced three new isonitrile sesquiterpenes, 2-isocyanoclovene (**173**), 2-isocyanoclovane (**174**) and 4,5-*epi*-10-isocyanoisodauc-6-ene (**175**), with selective antiplasmodial activity against the 3D7 and Dd2 strains. The isothiocyanate and formamide analogues were significantly less active, further reinforcing the argument that the isonitrile functionality is crucial for the potent activity of isonitrile terpenes [124]. Young et al. adapted a β-haematin inhibition assay to allow for the assaying of small amounts of marine natural products and was able to prove that six terpenoid isonitriles inhibit heme crystallization at different levels [125]. The sesquiterpene-derived spiro heterocycle 3,4-dehydrotheaspirone (**176**) (Fig. 25) has been isolated from *Laumoniera bruceadelpha* (Simaroubaceae) bark extract and was found to inhibit 3D7 parasites selectively [126].

### Sesquiterpene lactones

**Fig. 26:**
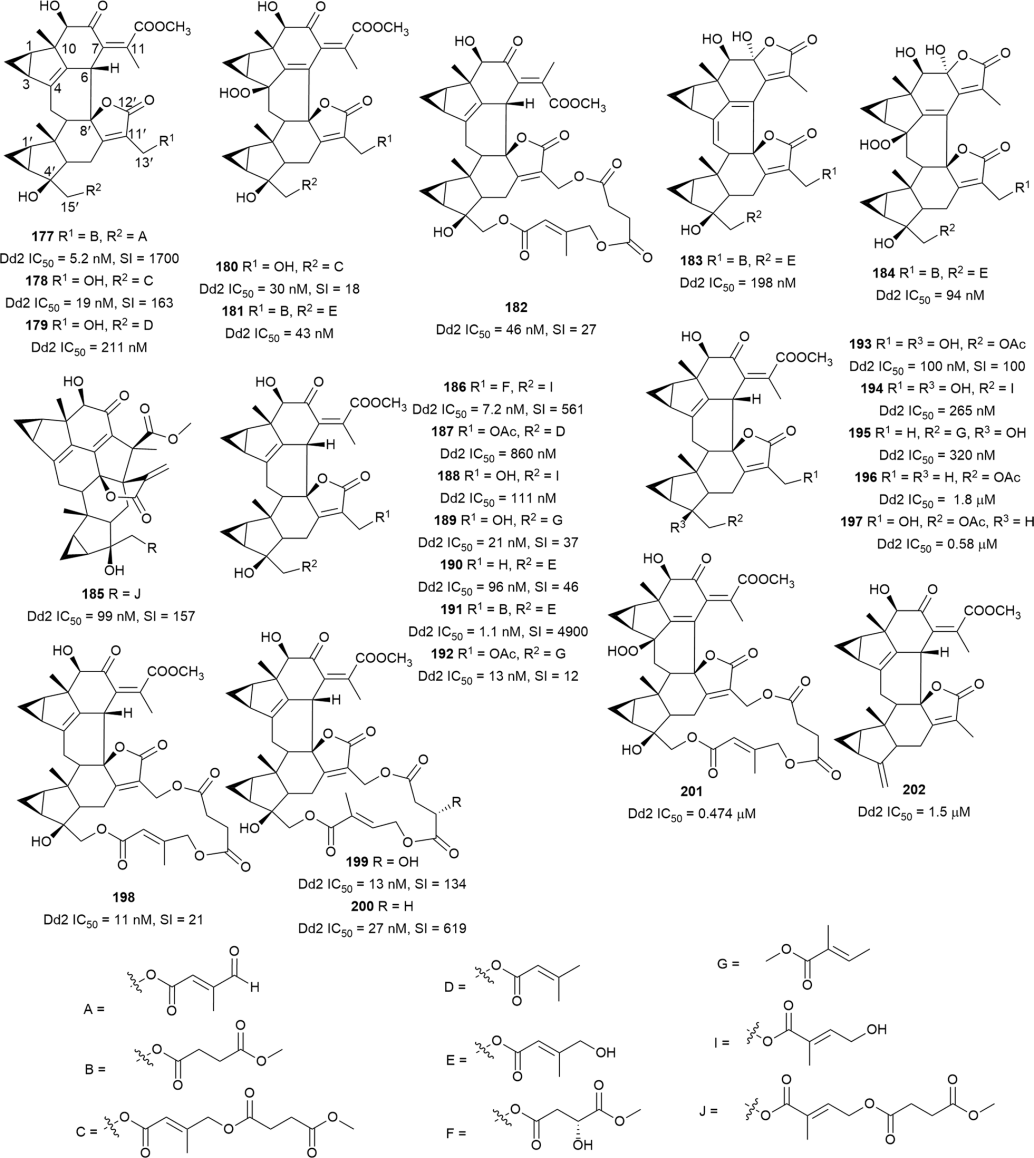
Structures of dimeric sesquiterpene lactones **177**–**202**

Many species in the genus *Chloranthus* (Chloranthaceae), known in traditional Chinese medicine as “Sikuaiwa”, have been documented as a treatment for malaria. A library of 44 lindenane-type sesquiterpenoid monomers and dimers isolated from different *Chloranthus* species and *Sarcandra glabra* (Chloranthaceae) were assayed for antiplasmodial activity. Potent activity was observed for twenty-six of the compounds (**177**–**202**) (Fig. 26). Compounds with IC_50_ ≤ 100 nM were also assessed for cytotoxicity on embryonic lung tissue (WI-38) cells, and some compounds, e.g. fortunilide A (**177**), sarglabolide J (**186**), and chlorajaponilide C (**191**) had potencies comparable to that of artemisinin and were not cytotoxic. Preliminary SAR observations indicated that all the active compounds were dimers, had a Δ^4^ double bond and a hydroxy group at C-4′, and contain a (*Z*)-5-hydroxy-4-oxopent-2-enoate ester. The presence and nature of the ester groups at C-13′ and C-15′ affected the antiplasmodial activity, suggesting that these ester groups could be manipulated to optimize potency [127]. The potent and selective antiplasmodial activity warrants further exploration of this group of compounds.

**Fig. 27:**
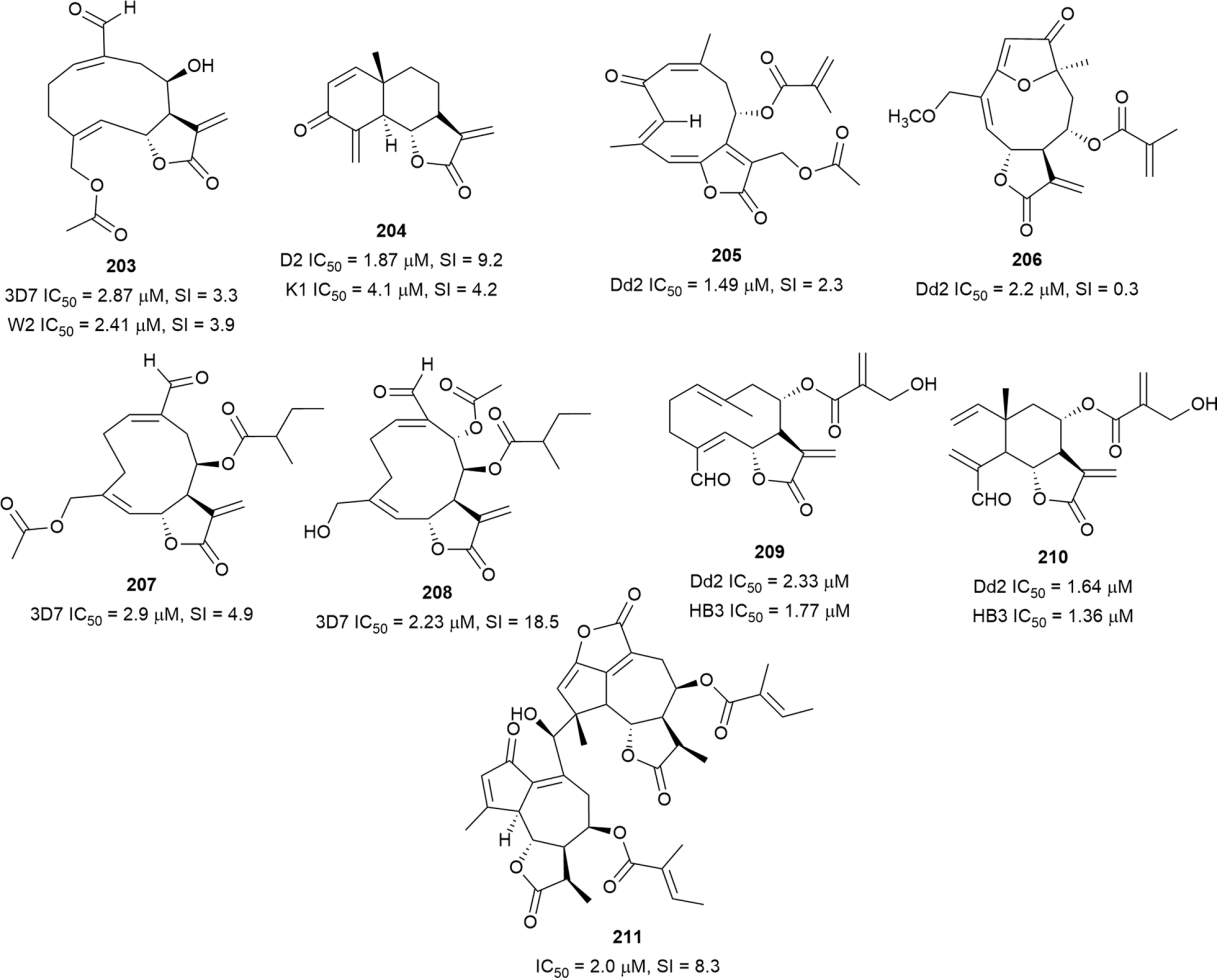
Structures of sesquiterpene lactones **203**–**211**

Thirteen plants used in Burkina Faso to treat malaria were investigated, and due to the promising in vitro and in vivo anti-malarial activity, *Dicoma tomentosa* (Asteraceae) was selected for further studies [128]. Bioassay-guided purification of the whole plant extract yielded the known germacranolide sesquiterpene lactone, urospermal A 15-*O*-acetate (**203**) (Fig. 27) as the major antiplasmodial compounds. The compound exhibited antiplasmodial activity against the 3D7 and W2 strains without evidence of haemolysis, indicating a direct action on the parasite. However, **203** was cytotoxic against WI38 human fibroblasts SI = 3.3, suggesting non-selective activity [129]. The root of an antiplasmodial *Dicoma* species from South Africa, *Dicoma anomala* subsp. *gerrardii* was the source of a eudesmanolide-type sesquiterpene lactone, dehydrobrachylaenolide (**204**), which inhibited D10 *P. falciparum* but was less active against the K1 strain and moderately cytotoxic against CHO cells (SI = 9). Semi-synthetic derivatives of **204**, in which the α-methylene ketone and lactone were reduced, were less active. This indicates that the exocyclic methylene group is essential for activity [130]. The dichloromethane extract of *Trichospira verticillata* (Asteraceae) exhibited antiplasmodial activity against Dd2 *P. falciparum* with an IC_50_ of approximately 5 µg/mL. Fractionation of the extract afforded the new germacranolide trichospirolide A (**205**) (Fig. 27) as the most active constituent. However, it was also toxic to A2780 ovarian cancer and HEK293 cells [131]. Another antiplasmodial germacranolide, 15-*O*-methylgoyazensolide (**206**), was isolated from the leaf and twig extract of *Piptocoma antillana* (Asteraceae). It was equally active against the Dd2 strain and A2780 human ovarian cancer cells, indicating a non-selective antiplasmodial activity [132]. Antiplasmodial screening of 12 plants used in traditional medicine against malaria in Benin resulted in an extract of the aerial parts of *Acanthospermum hispidum* (Asteraceae) with potent activity against 3D7 and W2 parasites (IC_50_ = 7.5 and 4.8 µg/mL, respectively) [133]. Two acanthospermolide-type sesquiterpene lactones (**207** and **208**) were subsequently isolated as the major antiplasmodial compounds without haemolytic activity [134]. Compound **208** was less cytotoxic against WI38 human fibroblasts than **207**, indicating that **208** was more selective in the toxicity to 3D7 parasites [134]. Two other sesquiterpene lactones, vernopicrin (**209**) and vernomelitensin (**210**), from *Vernonia guineensis* (Asteraceae) leaves were also active against Dd2 and Hb3 parasite strains without haemolysis [135]. The dichloromethane extract of *Eupatorium perfoliatum* (Asteraceae) aerial parts inhibited *P. falciparum* with low cytotoxicity (IC_50_ = 2.7 µg/mL and SI = 27). The new dimeric guaianolide, diguaiaperfolin (**211**), was isolated as the main antiplasmodial compound from the active extract, but the compound was moderately cytotoxic against L6 cells (SI = 8) [136].

All the different classes of sesquiterpene lactones reported so far exhibited non-selective antiplasmodial activity. The bioactivities of sesquiterpene lactones have been ascribed to the presence of an α-methylene-γ-lactone moiety in the structures. The conjugate Michael acceptor property of this highly reactive functionality allows it to react with the thiol group of crucial cell proteins hence the unselective activity. SAR studies around this enigmatic functionality to make sesquiterpene lactones more selective are essential if these compounds are to enjoy further development as anti-malarial scaffolds.

### Diterpenes

**Fig. 28:**
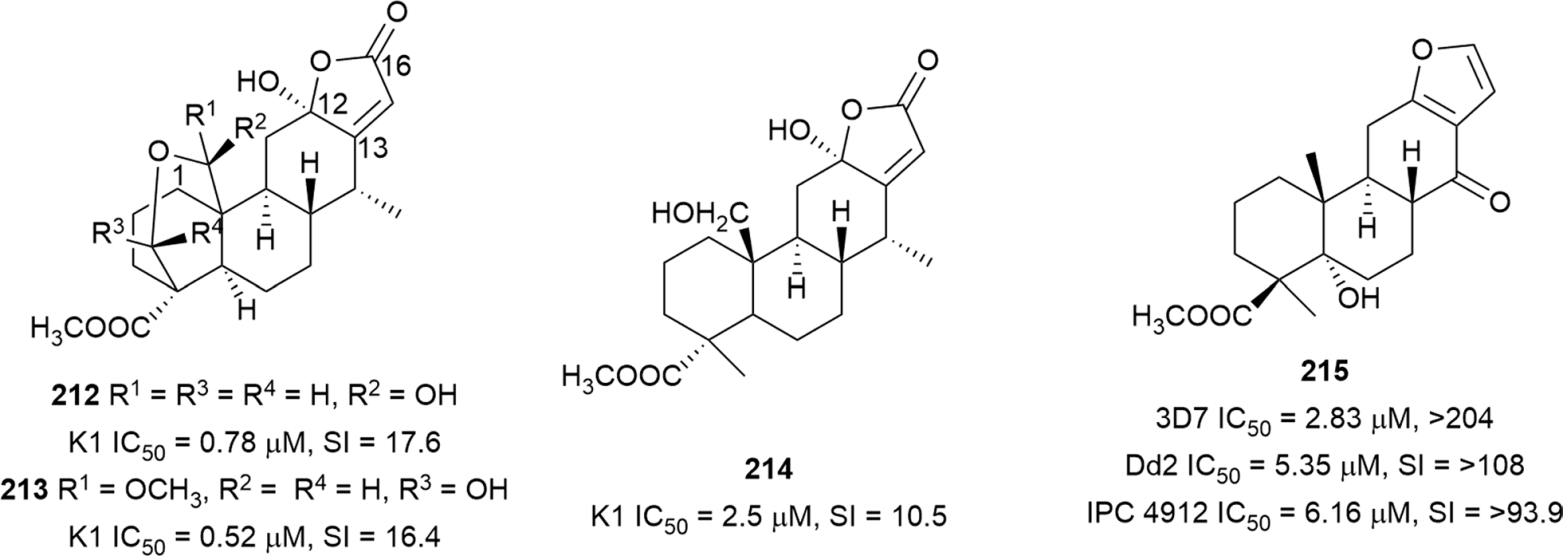
Structures of cassane diterpenes **212**–**215**

Three new cassane diterpenes (Fig. 28) from an active chloroform extract of *Caesalpinia sappan* (Fabaceae) seeds (IC_50_ = 0.38 µg/mL against K1), caesalsappanins G–I (**212**–**214**), inhibited K1 *P. falciparum* [137]. Cytotoxicity studies against a panel of cancer cell lines showed moderate selectivity for the parasite (SI = 10.5–17.6). The three active compounds have a hydroxy group at C-12, whereas compounds lacking the C-12 hydroxy were less active [137]. The dichloromethane extract of *Caesalpinia bonducella* root showed in vivo dose-dependent antiplasmodial activity [138]. A phytochemical investigation of the root afforded norcaesalpin D (**215**) as the antiplasmodial component. This cassane diterpenoid was active against 3D7, Dd2, and artemisinin-resistant (IPC 5202 Battambang, IPC 4912 Mondolkiri-Cambodia) strains. No cytotoxicity was observed against mammalian LLC-MK2 cells at the highest concentration (200 µg/mL) tested [139].

**Fig. 29:**
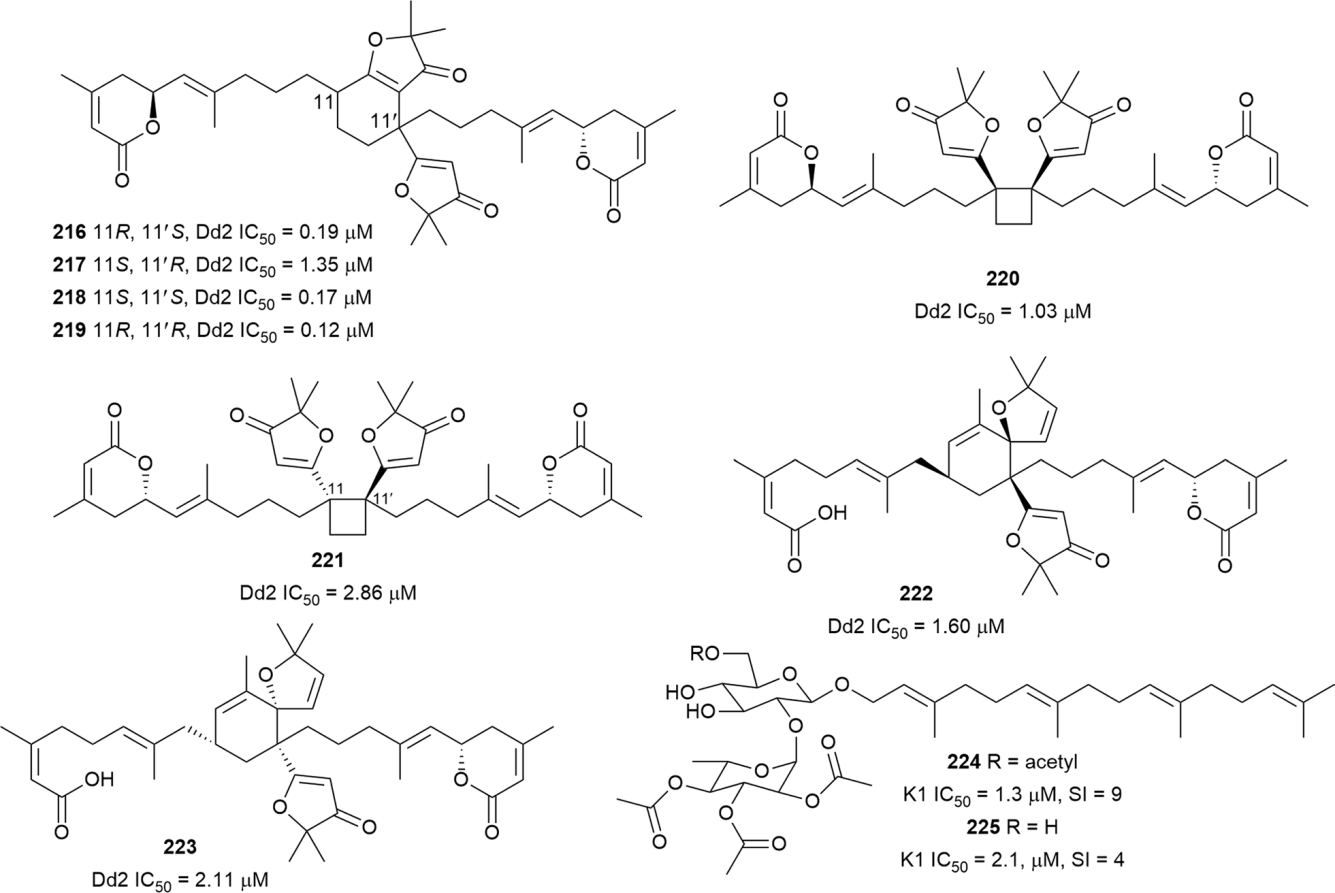
Structures of diterpenes **216**–**225**

The leaf extract of *Aphanamixis grandifolia* (Meliaceae) produced the diterpenoid lactones amphadilactones A–F and H–I (**216**–**223**) (Fig. 29) [140, 141]. The structures of amphadilactones E and F feature a novel carbon skeleton with a 1,1,2,2-tetrasubstituted cyclobutane moiety. Compounds **216**–**223** inhibited the Dd2 strain with sub-micromolar IC_50_ values. The interesting structures and antiplasmodial activity of **216**–**219** have motivated the total synthesis of the compounds [142]. Furthermore, compounds **216**–**219** showed potent inhibition of diacylglycerol *O*-acyltransferase-1 (DGAT-1) isozyme. *P. falciparum* encodes only one DGAT enzyme, *Pf*DGAT, and it is necessary for parasite proliferation during the intraerythrocytic stage [143]. With the unprecedented carbon frameworks, it is worthwhile to investigate whether the new amphadilactones exert antiplasmodial activity by inhibiting *Pf*DGAT [140, 141]. The hexane and dichloromethane bark extracts of *Cupania cinerea* (Sapindaceae), an Ecuadorian ethnobotanical plant, were active against *P. falciparum* K1 strain (IC_50_ = 2.9 and 3.1 µg/mL, respectively) [144]. Subsequent bioassay-guided purification of the extracts yielded the new linear diterpenoid glycosides cupacinoside (**224**) and 6′-de-*O*-acetylcupacinoside (**225**), both displaying antiplasmodial activity against K1 parasites but also cytotoxicity against L6 cells [145].

**Fig. 30:**
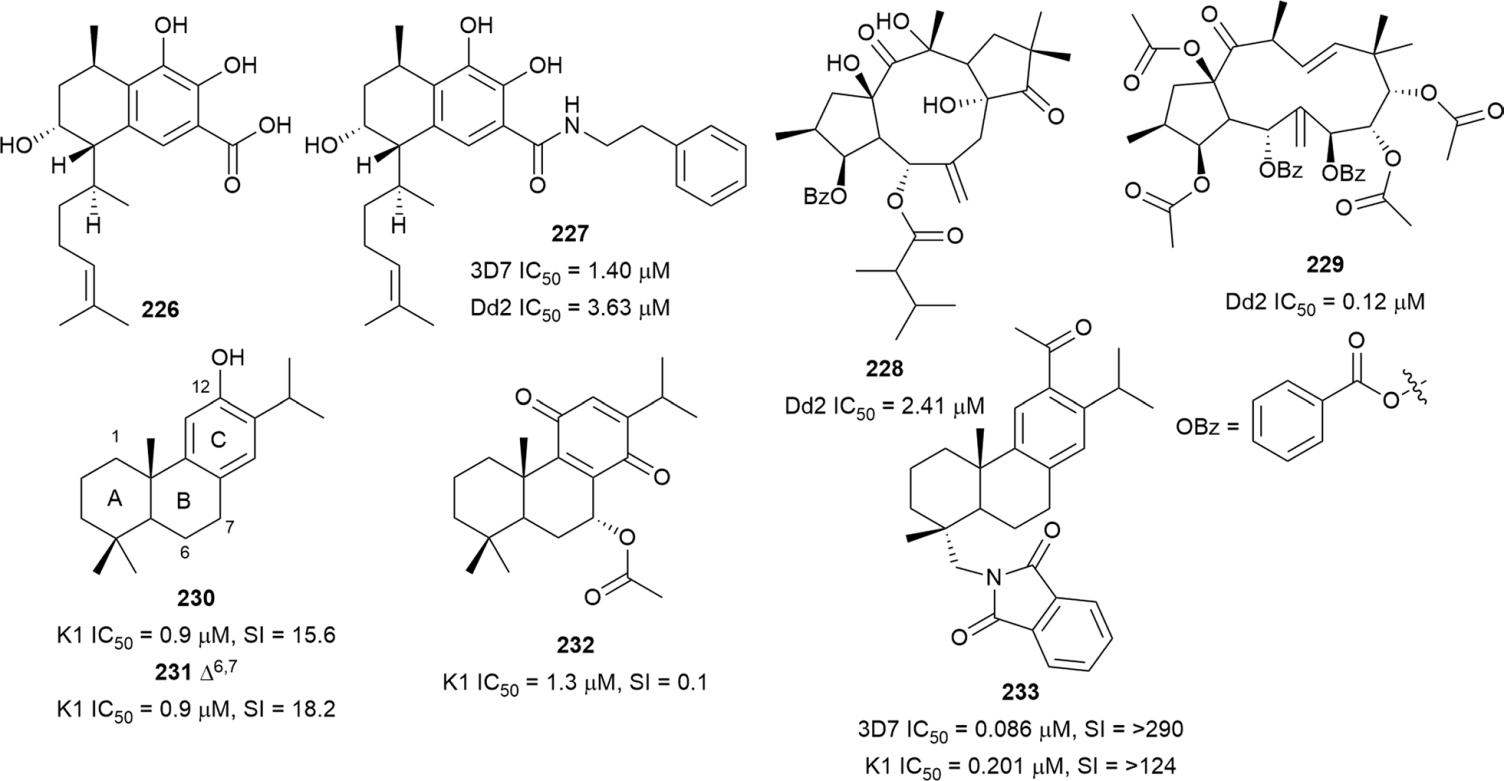
Structures of diterpenes **226**–**233**

The serrulatane diterpenoid **226**, which was isolated from the aerial parts of *Eremophila microtheca* (Scrophulariaceae), was not active at 10 µM against *P. falciparum*. However, a semi-synthetic amide derivative **227** exhibited antiplasmodial activity against the 3D7 and Dd2 strains without being cytotoxic to HEK293 cells at 80 µM [146]. Two new pre-segetane and jatrophane diterpenoids euphorbesulins A (**228**) and G (**229**) (Fig. 30), from the twigs of *Euphorbia esula* (Euphorbiaceae), have also shown activity against the *P. falciparum* Dd2 strain [147]. Antiplasmodial screening of 150 Iranian ethnomedicinal plants identified *Salvia sahendica* (Lamiaceae) hexane root extract with potent activity against the K1 strain (70% inhibition at 0.85 µg/mL) [148]. A subsequent phytochemical investigation led to the isolation of abietane diterpenoids as the bioactive constituents and ferruginol (**230**), Δ^9^-ferruginol (**231**), and 7α-acetoxyroyleanone (**232**) inhibited K1 parasites. However, **232** was also toxic to L6 cells, indicating a non-selective antiplasmodial activity. Chemical modifications, which included deacetylation and dehydrogenation of ring B, and hydroxylation of the benzoquinone ring of **232**, led to a reduction in activity without improving selectivity [148]. However, the semi-synthetic phthalimide derivative **233** of ferruginol had an improved selective antiplasmodial activity. Preliminary SAR studies of a library of semi-synthetic derivatives of ferruginol indicated that a hydroxy group at C-12 is beneficial for activity while an acetate group at C-12 reduced the activity against the K1 strain, but improved the activity against the 3D7 strain. Chlorination of the phthalimide group was detrimental to activity [149].

**Fig. 31:**
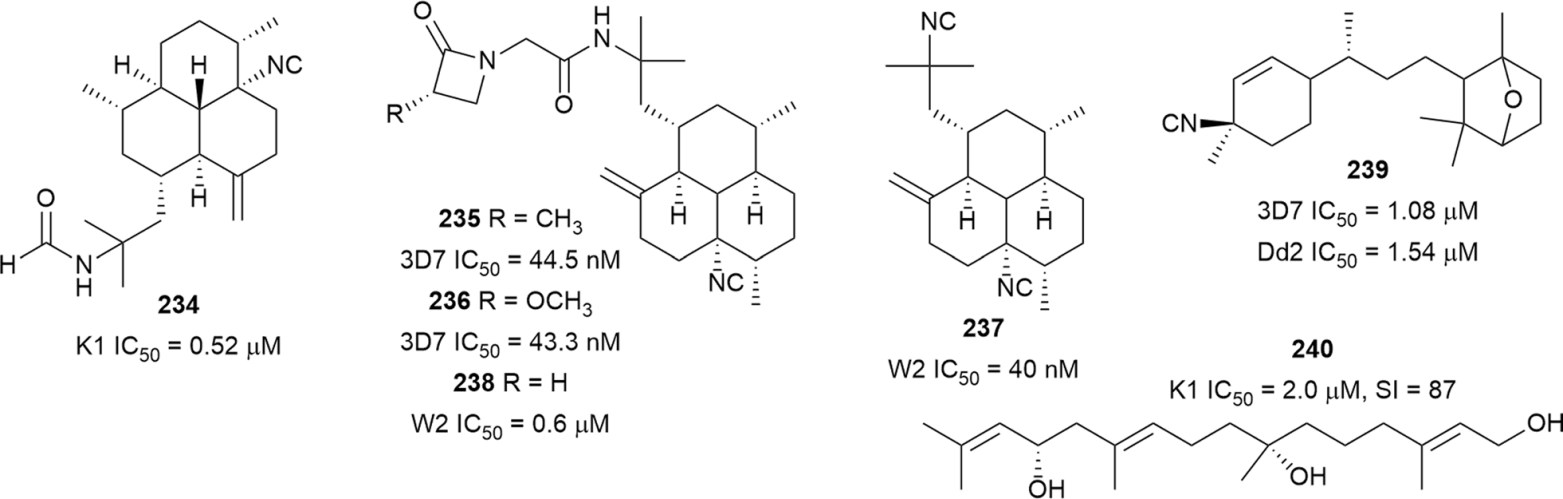
Structures of isocyano diterpenes **234**–**239** and bifurcatriol

A marine sponge from Thailand, *Stylissa cf. massa*, has produced some bifunctionalized amphilectane diterpenoids. The most active metabolite, 8-isocyano-15-formamidoamphilect-11(20)-ene (**234**) (Fig. 31), exhibited antiplasmodial activity against the K1 strain and was not cytotoxic against MCF-7 breast cancer cells. Analogues bearing an isocyanate and isothiocyanate functionalities were up to ten times less active, indicating that the isonitrile group improved activity. Also, an analogue with only the formamide functional group but lacking an isonitrile group, was not active against *P. falciparum*, suggesting that the formamide group does not contribute to antiplasmodial activity [150]. Two more isonitrile amphilectanes, monamphilectines B (**235**) and C (**236**), were isolated from the Carribean marine sponge *Svenzea flava* collected off the coast of Puerto Rico [151]. The new metabolites were described as the first marine natural products with an α-substituted monocyclic β-lactam ring. The compounds were active against the 3D7 parasites with nanomolar IC_50_ values. Interestingly, 8,15-diisocyano-11(20)-amphilectene (**237**), which differs from the new compound by the absence of the substituted β-lactam moiety, was also active, which suggests that the β-lactam moiety does not contribute to antiplasmodial potency and further confirms the crucial role of the isocyanide (isonitirile) functionality [151]. Potent antiplasmodial activity against W2 parasites was also reported for monamphilectine A (**238**), with an unsubstituted β-lactam ring [152]. Pustulosaisonitrile-1 (**239**), which was isolated from the Australian nudibranch *Phyllidiella pustulosa*, exhibited antiplasmodial activity against 3D7 and Dd2 *P. falciparum* strains [153]. Diastereoisomers of **239**, obtained by enantio- and stereoselective total synthesis, were as active as the natural compound, but also showed cross-resistance [153]. The antiplasmodial activity of isonitrile terpenoids has been demonstrated to be due to inhibition of haemozoin formation [125, 154]. Therefore, further development of this class of compound will depend on the ability to avoid cross-resistance. A new linear diterpenoid, bifurcatriol (**240**), featuring two stereogenic centres, was isolated from the Irish brown alga *Bifurcaria bifurcata* and was active against K1 *P. falciparum* with negligible cytotoxicity against L6 cells [155].

**Fig. 32:**
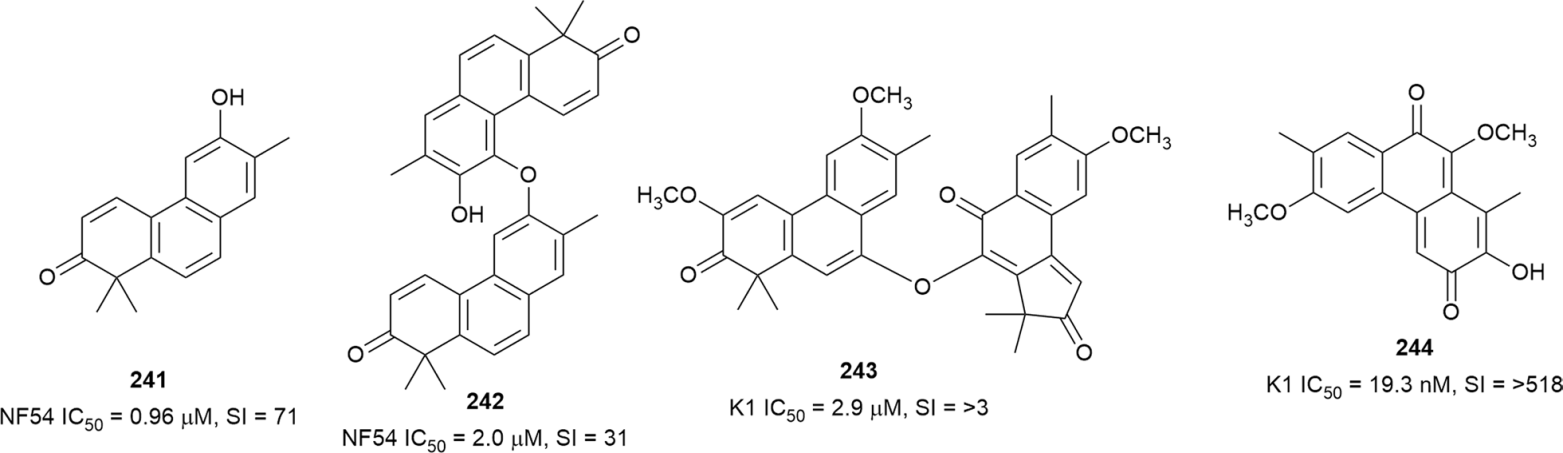
Structures of other diterpenoids

The stem extract of *Drypetes gerrardii* var *gerrardii* (Putranjivaceae) exhibited potent antiplasmodial activity against *P. falciparum* (IC_50_ = 0.5 µg/mL). Two new metabolites, the diterpene-derived phenanthrenone drypetenone D (**241**) and phenanthrenone heterodimer drypetenone E (**242**) (Fig. 32), were subsequently isolated from this extract. These compounds were active against the NF54 strain with low cytotoxicity against L6 cells (SI = 71 and 31, respectively). However, the more active and selective monomer **241** did not show in vivo activity in *P. berghei*-infected mice [156]. The phenanthrenone derivatives fimbricalyx A (**243**) and B (**244**), isolated from *Strophioblachia fimbricalyx* (Euphorbiaceae) root, also inhibited *P. falciparum* K1. Interestingly, the new fimbricalyx B exhibited nanomolar antiplasmodial activity, better than mefloquine, without being cytotoxic against Vero and human cancerous cells at 10 µM [157].

### Triterpenes

**Fig. 33:**
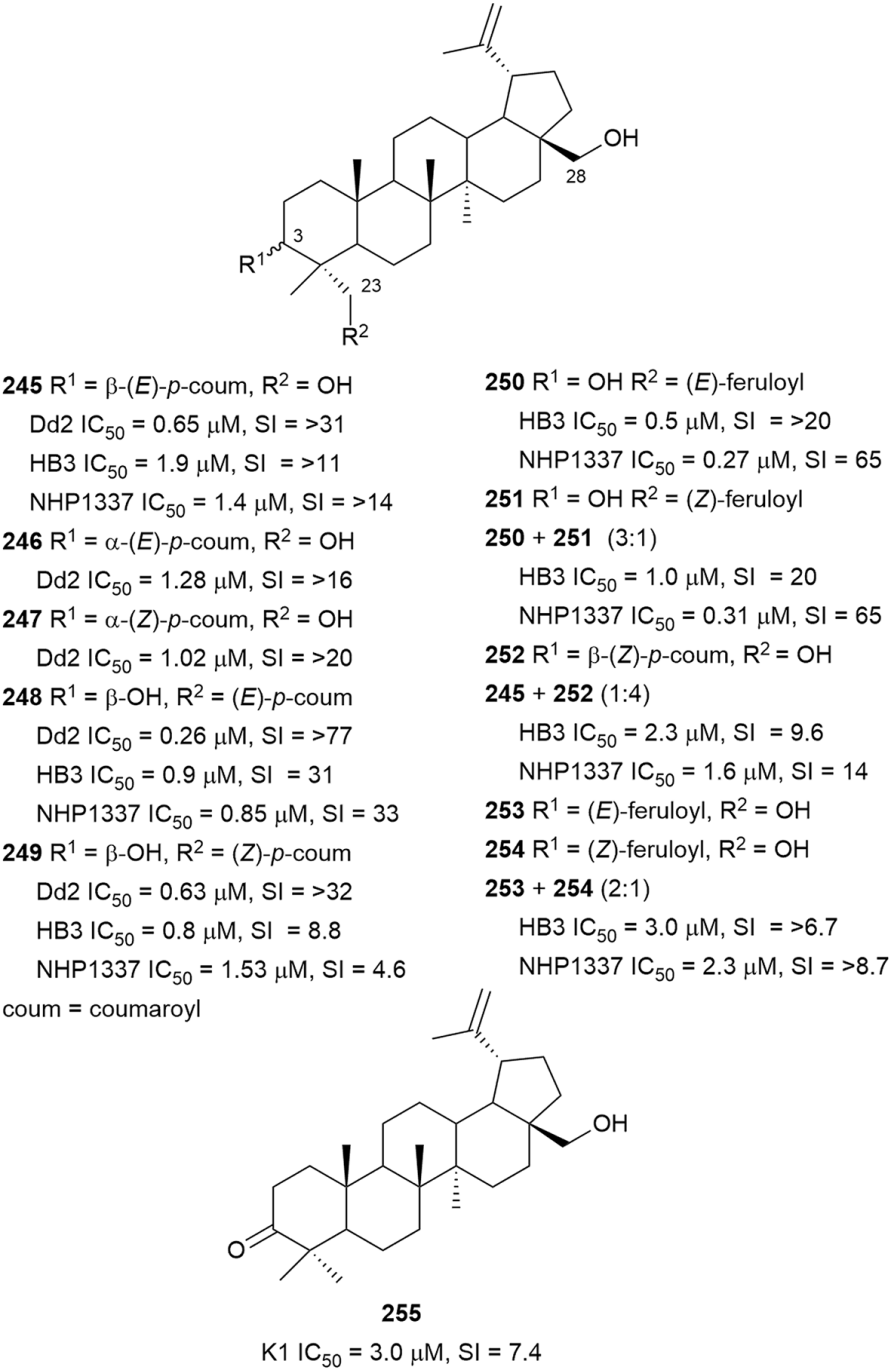
Structures of betulin derivatives

The chloroform extract of a combination of *Buxus cochinchinensis* (Buxaceaea) leaves, twigs, and fruits yielded five betulin coumaroyl esters (**245**–**249**) (Fig. 33) [99]. The lupane esters **245, 248**, and **249** were also isolated from the methanolic supercritical fluid extract of *B. sempervirens* together with five other new coumaroyl and feruloyl esters of betulin (**250**–**254**) [158]. The coumaroyl and feruloyl esters have either a *Z*- or *E*-configuration and are attached to betulin at either C-3 or C-23. The esters were active against *P. falciparum* Dd2, HB3, and NHP1337 strains without cytotoxicity against HeLa cells. Ester **250**, with an *E*-feruloyl group attached at C-23, was the most active with sub-micromolar IC_50_ values, whereas analogues with *E* or *Z* coumaroyl or *Z*-feruloyl at C-23 were slightly less active. Also, C-3 modified analogues were less active than the C-23 modified counterparts. These observations identify the importance of the *E*-feruloyl moiety and suggest that modification at C-23 is more advantageous for activity. Importantly, the esterified betulin derivatives were more active than betulin and 23-hydroxybetulin, and the diacetate ester was inactive [99, 158]. Betulone (**255**), isolated from the bark of *Cupania cinerea* (Sapindaceae), inhibited *P. falciparum* K1 and had moderate cytotoxicity against L6 cells [145].

**Fig. 34:**
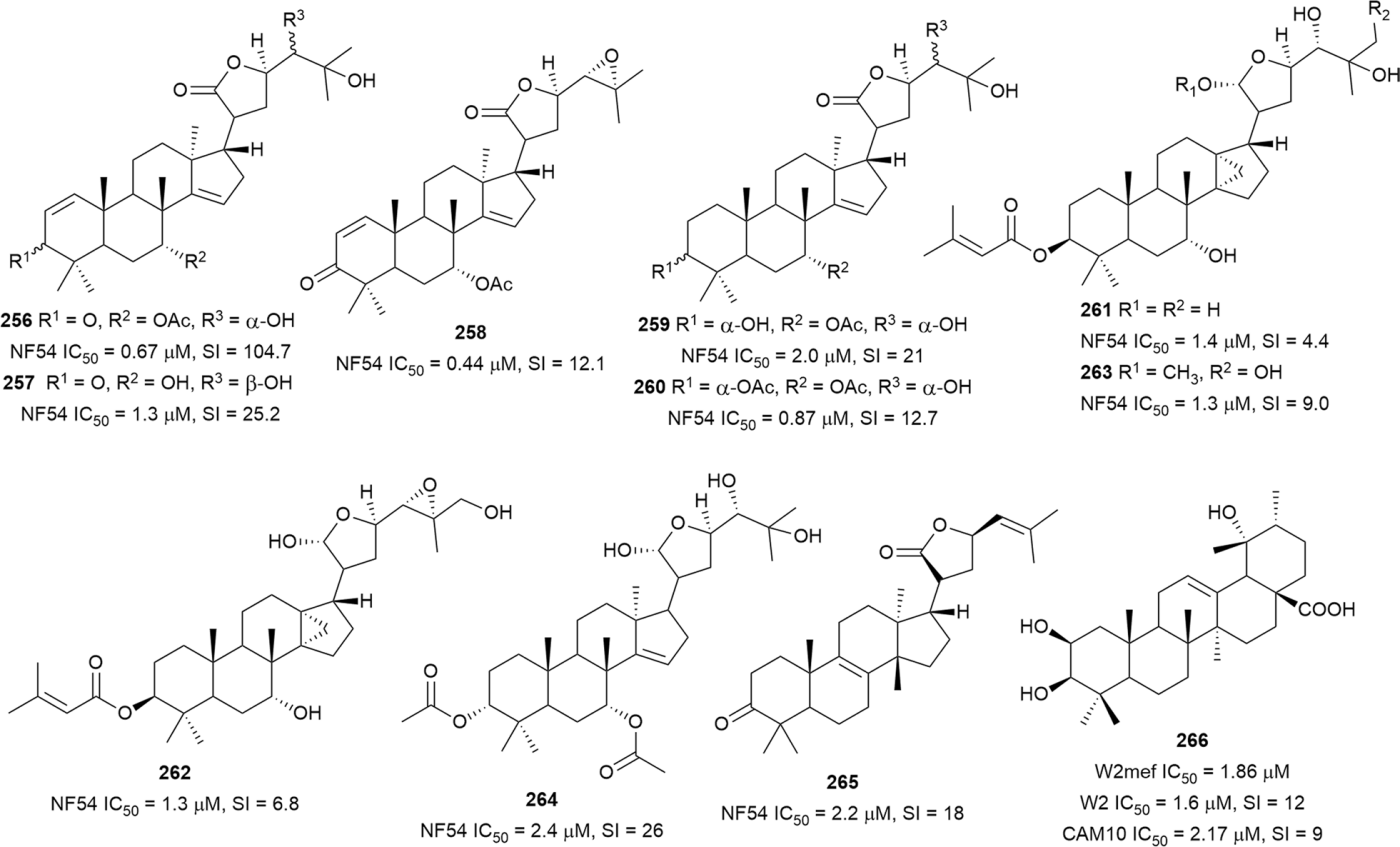
Structures of triterpenes **256**–**266**

Happi et al. investigated the chemical constituents of *Entandrophragma congoense* (Meliaceae) bark, a plant used in Cameroonian traditional medicine against malaria. They isolated the apotirucallane triterpenoids prototiamins A–G (**256**–**262**) and the known **263** as the major constituents, and gladoral A (**264**) was obtained as a minor metabolite (Fig. 34) [159]. The compounds displayed antiplasmodial activity against NF54 *P. falciparum* strain with varying levels of toxicity to L6 cells (SI = 4–107). Triterpenoid **256**, with a sub-micromolar IC_50_ value, was the most selective against the parasite [160, 161]. Comparing the activities of the compounds allowed some preliminary SAR assumptions. Compounds **256** and **257** differ only in the orientation of the hydroxy group at C-24 and presence or absence of acetylation at C-7 of ring B. Analogue **256**, with an α-oriented OH group and acetylation of the OH at C-7, was twice as active and four times more selective than **257**. Compound **258**, which has a similar structure to **256** but with an epoxide ring between C-24 and C-25 instead of the free α-OH in **256**, was > 8 times less selective. These observations suggest a SAR role for these positions that could be exploited further to optimize potency and selectivity. Another tirucallane triterpenoid, isoflindissone lactone (**265**), was isolated from the dichloromethane extract of *Boswellia serrata* (Burseraceae) oleo-gum resin by bioassay-guided purification of an extract that inhibited *P. falciparum* with IC_50_ = 2.6 µg/mL. Compound **265** was active against the NF54 parasite strain with low toxicity against L6 cells (IC_50_ = 40 µM) [162]. The ethyl acetate stem bark extract of *Kigelia africana* (Bignoniaceae) inhibited *P. falciparum* W2 strain and two field isolates, CAM10 and SHF4 (IC_50_ = 11.15, 4.74 and 3.91 µg/mL, respectively). Phytochemical investigations of this active extract yielded the known triterpenoid **266** alongside other metabolites. Compound **266** was active against the W2 and CAM10 strains, with moderate cytotoxic against monkey kidney (LLC-MK2) cells (IC_50_ = 9.4 µg/mL) [117]. A synergistic effect was observed with a combination of **266** and artemether on the W2mef parasite strain, but the compound had an antagonistic effect with quinine [118].

**Fig. 35:**
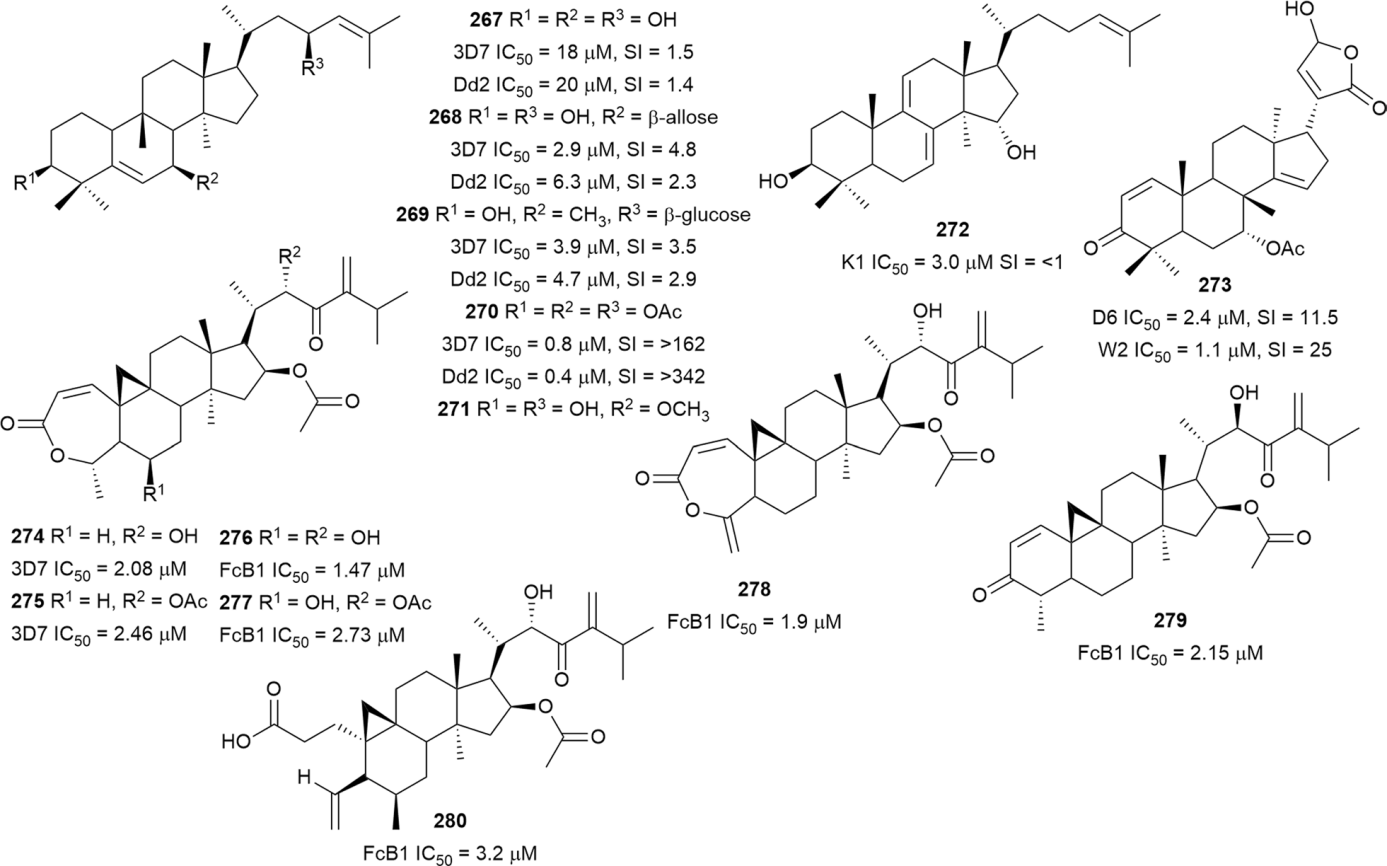
Structures of triterpenes **267**–**280**

Bioassay-guided fractionation of the methanol extract of the aerial parts of *Momordica balsamina* (Cucurbitaceae) afforded the new curcurbitacin balsaminol F (**267**), the glycoside balsaminoside B (**268**), and the known kuguaglycoside A (**269**) (Fig. 35). Glycosides **268** and **269** displayed antiplasmodial activity against the 3D7 and Dd2 strains, while the aglycone **267** was much less active. This suggests that the sugar unit is beneficial to the antiplasmodial activity. However, the compounds were not selective when the cytotoxicity against MCF-7 breast cancer cells is compared to the antiplasmodial activity. Interestingly, the triacetyl semi-synthetic derivative **270** of balsaminol F was 22 and 50 times (for 3D7 and Dd2, respectively) more active than the parent compound without cytotoxicity against MCF-7 cells. However, the activity was lost with the corresponding tribenzoyl ester derivative of balsaminol F [163]. Similar improvement in potency and selectivity was observed when karavilagenin C (**271**), which was isolated from *Momordica balsamina*, and was esterified at C-3 and/or C-23 to give different alkanoyl and benzoyl/cinnamoyl derivatives. For the alkanoyl analogues, the diacetyl and dipropanoyl derivatives were more active than the mono analogues, while the monobutanoyl compound was more active than the dibutanoyl counterpart. Moreover, all the mono-aroyl/cinnamoyl derivatives were superior compared to the diaroyl/cinnamoyl counterparts [164]. These observations allow the conclusion that for bulky groups, mono-esterification is optimal for activity, while di-esterification is favoured for the smaller groups. It will be worthwhile to study the preferred point of esterification for the monoesters.

**Fig. 36:**
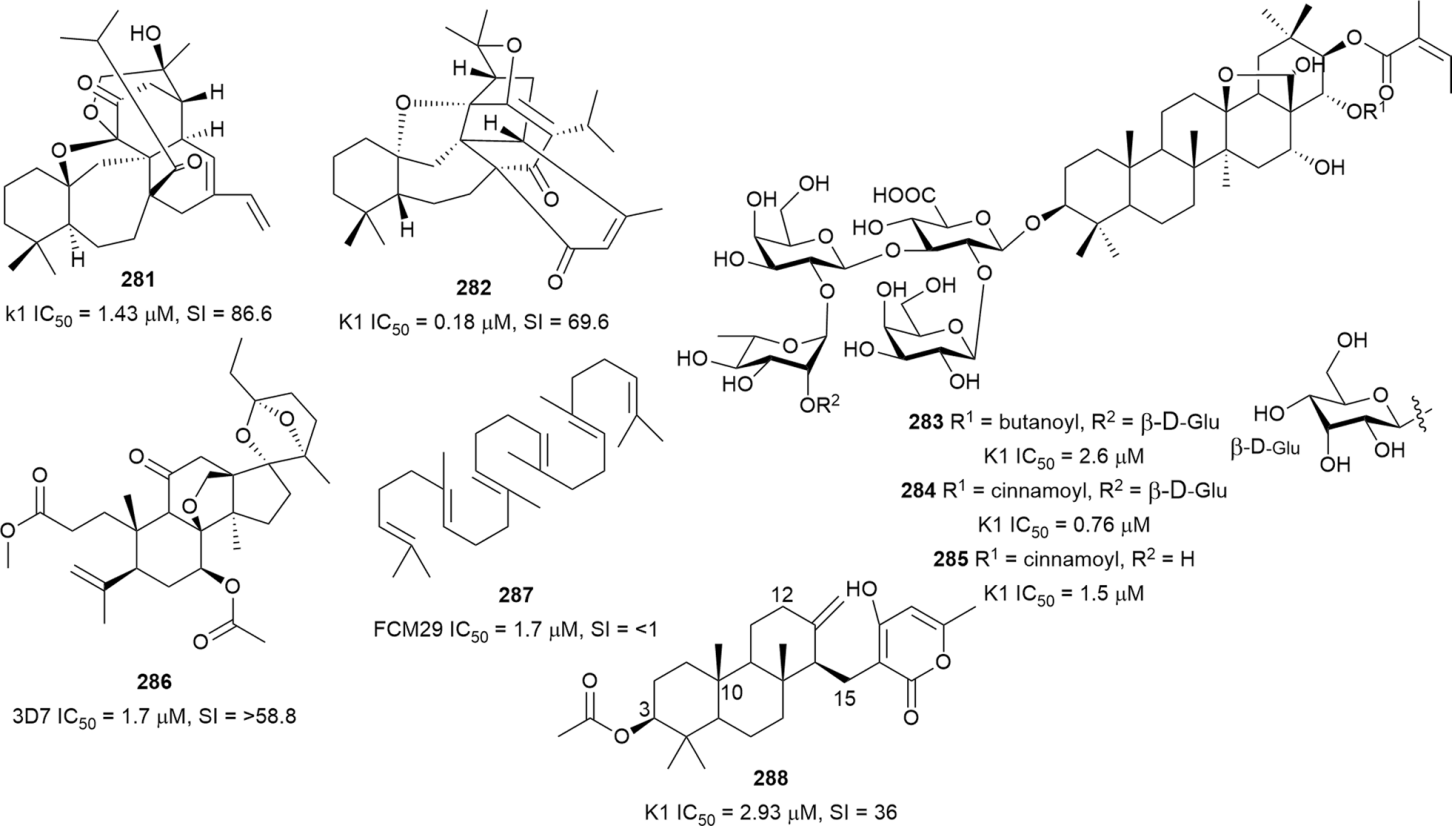
Structures of other triterpenes

The root bark of *Greenwayodendron suaveolens* (Annonaceae) afforded polycarpol (**272**) as one of the active metabolites against K1 *P. falciparum*, but it was also cytotoxic against MRC-5 cells (SI = < 1) [69]. Antiplasmodial assay of 14 Kenyan medicinal plants identified the methanol root bark extract of *Turraea robusta* (Meliaceae) as the most active against NF54 and K1 parasites (IC_50_ = 2.4 and 3.5 µg/mL, respectively) [165]. Azadironolide (**273**) was subsequently isolated as the most active antiplasmodial compound from the plant stem bark with moderate cytotoxicity against Vero cells [166]. An ethnomedicinal survey of plant use in the Northern sector of Kibale National Park in western Uganda indicated that *Neoboutonia macrocalyx* (Euphorbiaceae) is used to treat malaria [167]. Chemical investigation of the plant leaf afforded the new cycloartane triterpenoids neomacrolactone (**274**), 22α-acetoxyneomacrolactone (**275**), 6-hydroxyneomacolactone (**276**), 22α-acetoxy-6-hydroxyneomacrolactone (**277**), and 4-methylene-neomacrolactone (**278**), and the previously reported 22-de-*O*-acetyl-26-deoxyneoboutomellerone (**279**) (Fig. 35). These compounds exhibited antiplasmodial activity against FcB1 *P. falciparum*, but were generally cytotoxic against KB and MRC-5 cells. Interestingly, neomacroin (**280**) with an open ring A, thus lacking an α,β-unsaturated carbonyl conjugated to the cyclopropane ring, showed low cytotoxicity but was slightly less potent (IC_50_ = 3.2 µM) [168]. Two novel triterpenoids, salvadione C (**281**) and perovskone B (**282**) (Fig. 36), with rare carbon skeletons, were isolated from an antiplasmodial hexane extract of *Salvia hydrangea* (Lamiaceae). The antiplasmodial activity against K1 parasites was selective when compared to cytotoxicity against L6 cells. The rare carbon scaffolds can be rationalized by a Diels–Alder-type addition of an acyclic monoterpene to a diterpenoid. The monoterpene in the case of **281** is myrcene and *trans*-β-ocimene for **282**, and the additional oxepane ring in **281** confers structural rigidity. These structural types were only previously reported in salvadiol from *Salvia bucharica* and perovskone from *Perovskia abrotanoides* (Lamiaceae) [169]. Three new triterpenoid saponins, maesargentoside I, III, IV (**283**–**285**) from the leaf extract of *Maesa argentea* (Myrsinaceae) have displayed non-selective antiplasmodial activity against K1 *P. falciparum* [170]. The medicinal mushroom *Ganoderma boninense* produced a new nortriterpenoid with a 3,4-seco-27-norlanostane rearranged skeleton. The metabolite ganoboninketal C (**286**) inhibited *P. falciparum* 3D7 strain with low cytotoxicity against A549 cells [171]. The antiplasmodial activity of squalene (**287**), isolated from *Uapaca paludosa* (Euphorbiaceae) trunk bark extract, was reported for the first time. However, it was also cytotoxic against KB and Vero cells [172].

The ethyl acetate extract of the soil fungus *Neosartorya tatenoi* KKU-2NK23 exhibited antiplasmodial activity (IC_50_ = 3.09 µg/mL). A chemical investigation of the fungal material yielded the known meroterpenoid aszonapyrone A (**288**) (Fig. 36). The compound was active against the K1 strain with low cytotoxicity against KB cells (IC_50_ = 48.18 µg/mL). However, the compound was cytotoxic against cancerous NCI-H187 cells, suggesting some level of selectivity in the toxicity to cells. An analogue of **288**, which had a free hydroxy group at C-3 instead of the acetoxy in **288**, was inactive, suggesting a SAR role at this position and indicating that acetylation of the free hydroxy is beneficial to activity [173].

### Steroids

**Fig. 37:**
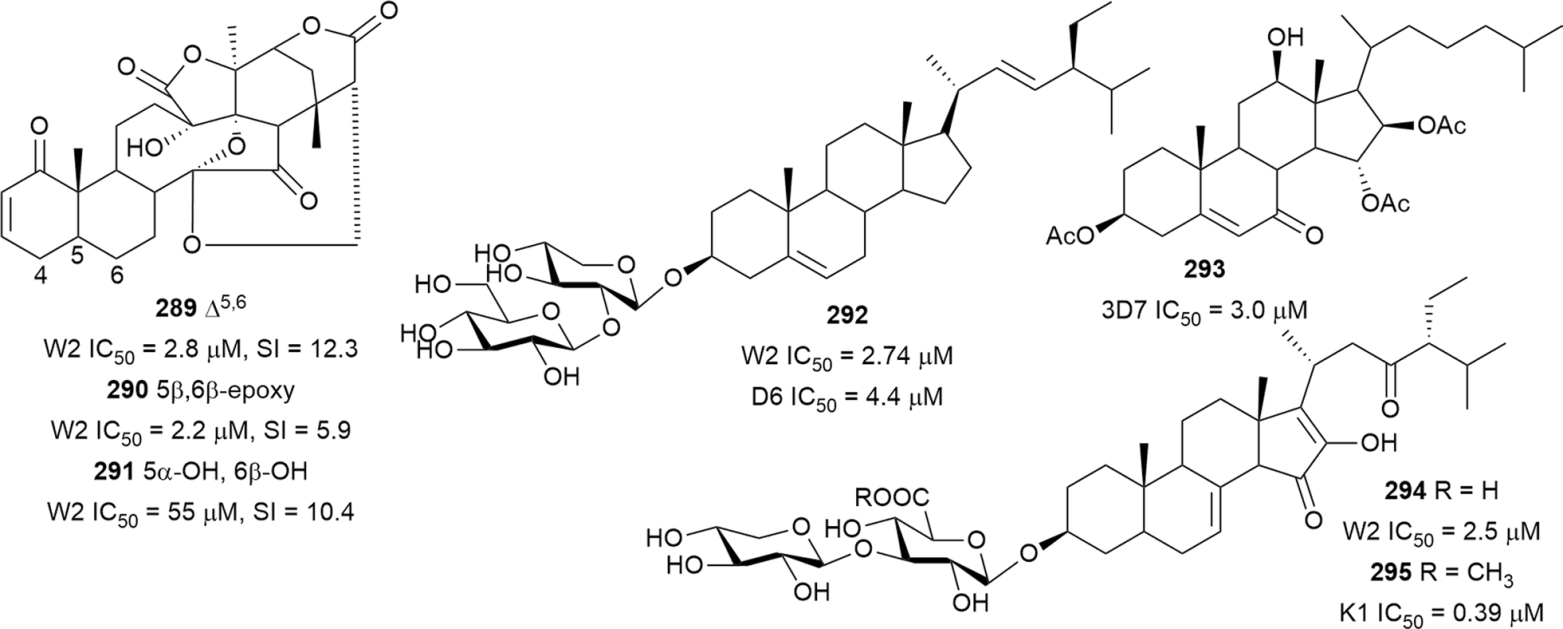
Structures of steroids

An analysis of 65,000 small molecules using the in silico similarity ensemble approach (SEA), predicted antiplasmodial activity for selected physalins. Physalins B, D, F, and G were then isolated from *Physalis angulata* (Solanaceae) and evaluated for in vitro and in vivo anti-malarial activity. Physalins B (**289**) and F (**290**) (Fig. 37) had in vitro activity against W2 parasite but were also cytotoxic (SI = 12 and 6, respectively). Interestingly, all the mice treated with **290** died from an exacerbated infection due to the increase in parasitaemia that was attributed to an immunosuppressive effect of the compound. However, physalin D (**291**), without the immunosuppressive effect, decreased parasitaemia in *P. berghei* infected mice by 65% at 100 mg/kg [174]. The *n*-butanol fraction of *Caesalpinia volkensii* (Fabaceae) methanol stem bark extract inhibited *P. falciparum* D6 and W2 strains (IC_50_ = 4.5 and 1.3 µg/mL, respectively) better than the less polar fractions. Bioassay-guided purification led to the isolation of the new steroid glycoside 3-*O*-[β-glucopyranosy-(1 → 2)-*O*-β-xylopyranosyl]stigmasterol (**292**) with antiplasmodial activity against the D6 and W2 strains. Crucially, the aglycone, which was isolated from the chloroform fraction, was inactive, suggesting that the two sugars potentiate antiplasmodial activity [175]. The marine red alga *Halymenia floresii* has produced a new steroid, halymeniaol (**293**), which inhibited 3D7 *P. falciparum* and was not cytotoxic [176]. Chemical reinvestigation of the Caribbean sponge *Pandaros acanthifolium* has yielded two new steroid glycosides, pandaroside G (**294**) and pandaroside G methyl ester (**295**). The two compounds were active against the K1 strain as well as cytotoxic against L6 cells, indicating non-selective toxicity. The compounds did not inhibit recombinant *P. falciparum* fatty acid biosynthesis enzymes at the highest tested concentration (20 µg/mL) [177].

### Limonoids

**Fig. 38:**
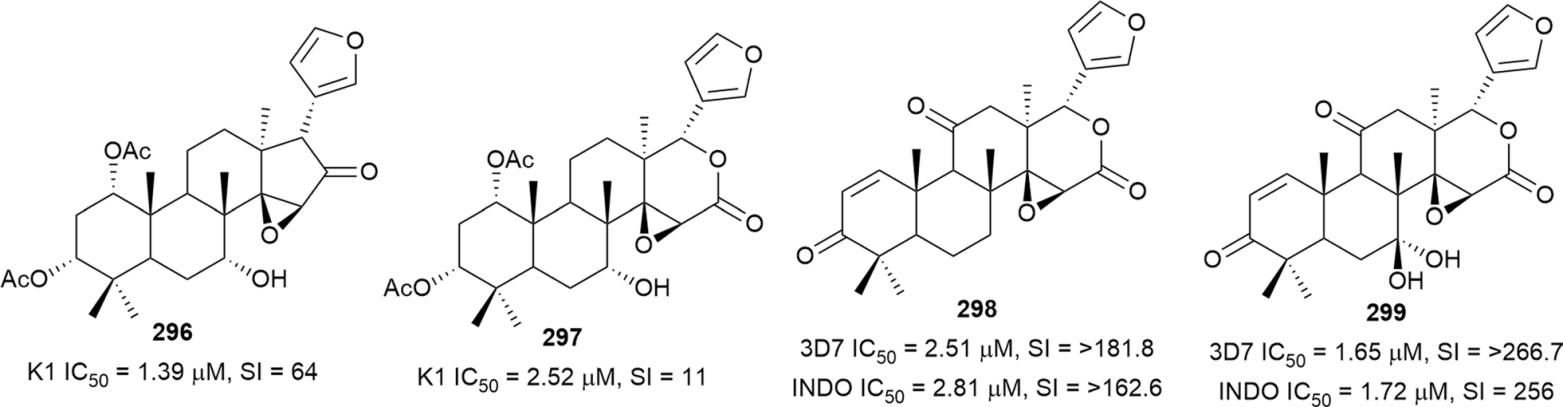
Structures of limonoids

African great apes such as Chimpanzees have been observed to ingest the non-nutritional bitter bark and sap of *Khaya anthotheca* (Meliaceae) in the wild, and it has been proposed that this unusual feeding is for medicinal purposes [178, 179]. In an effort to identify bioactive constituents from this plant, the seed petroleum ether extract was tested against *P. falciparum* and showed good activity (IC_50_ = 0.96 µg/mL). Bioassay-guided purification of the extract yielded the known limonoids grandifolione (**296**) and 7-deacetylkhivorin (**297**) (Fig. 38) as the active antiplasmodial constituents. The compounds inhibited the K1 strain, and **296** was less toxic towards L6 cells than **297**, SI = 64 and 11, respectively [180]. Two new antiplasmodial limonoids, kostchyienones A (**298**) and B (**299**), were isolated from the root extract of *Pseudocedrela kostchyi* (Meliaceae). Previous investigations indicated that the extract inhibited *P. falciparum* schizont development [181]. Antiplasmodial activity of the new compounds against 3D7 and *Pf*INDO strains was selective, and the compounds were not toxic to HEK239T cells (IC_50_ > 200 µg/mL). The antiplasmodial activity of limonoids has been attributed to the presence of the α, β-unsaturated carbonyl moiety in the structures, which may be involved in Michael-type addition reactions [182].

### Quassinoids

**Fig. 39:**
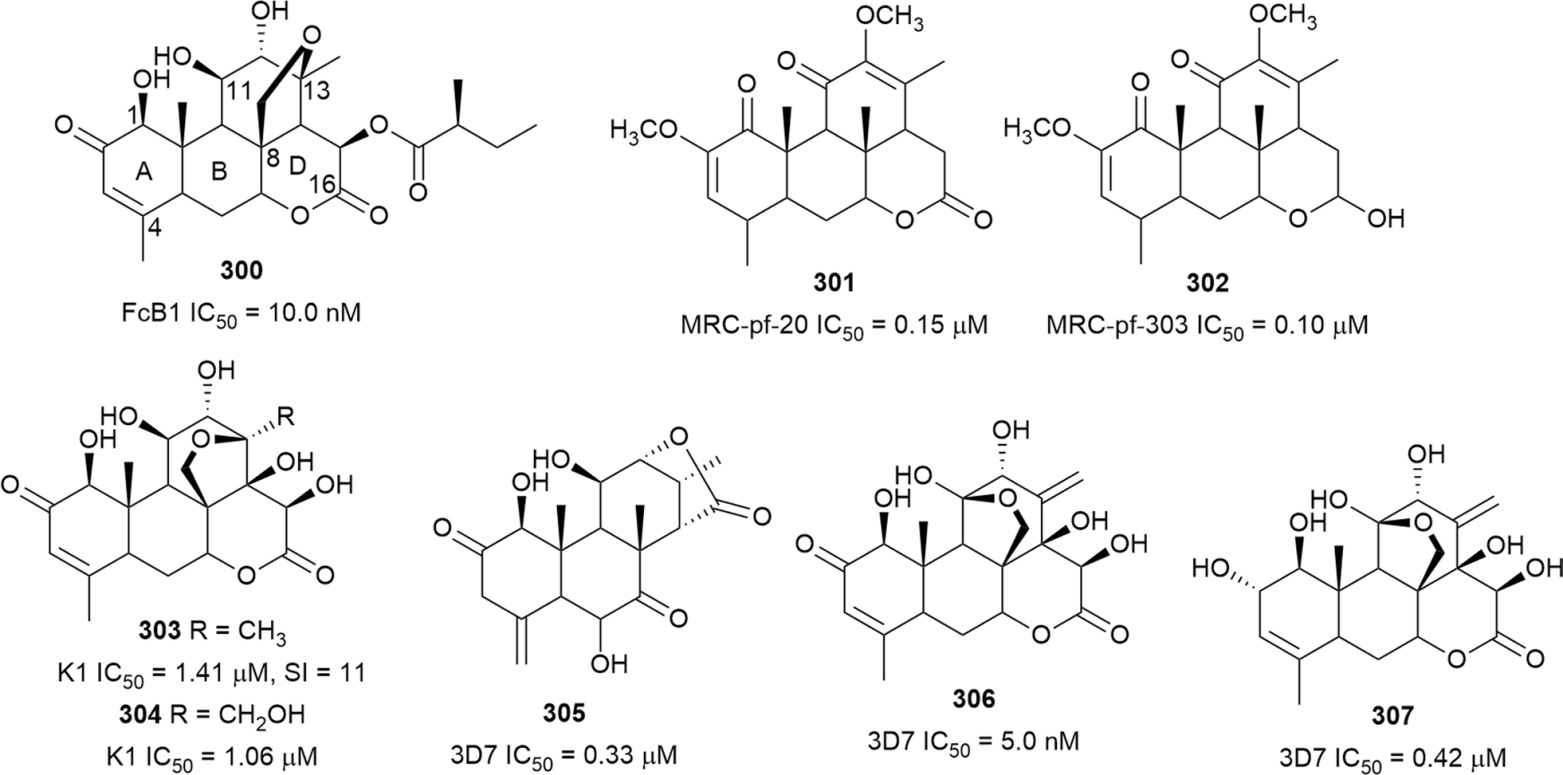
Structures of quassinoids

The leaf of the tropical Amazonian medicinal plant *Quassia amara* (Simaroubaceae) is used traditionally by people in French Guinea for the preparation of popular anti-malarial remedies, alone or in combination with other plants [183]. The plant leaf extract was prepared according to traditional instructions and found to be active against *P. falciparum* in vitro and in vivo, without signs of general toxicity [184]. The known quassinoid simalikalactone D (**300**) (Fig. 39), identified as the main antiplasmodial component of *Quassia amara*, had an IC_50_ of 10 nM against the FcB1 parasite and showed in vivo efficacy (oral) [185]. The known cytotoxicity of quassinoids prompted investigations into the toxicity of **300**. The compound displayed antiproliferative activity against cancerous KB cells (IC_50_ = 6.3 nM) but was less cytotoxic against HeLa and noncancerous Vero cells (IC_50_ = 2 and 10 µM, respectively). Similarly, Raji B cells mitotic activity was inhibited at concentrations larger than 45 nM, but no apoptosis or necrosis was observed at a concentration of up to 200 µM of **300**. The compound did not inhibit heme crystallization or parasite-induced host erythrocyte membrane permeability. Moreover, **300** showed a stage-specific activity by inhibiting DNA replication in mature trophozoites. An additive effect was observed in combination studies with **300** and the traditional anti-malarials chloroquine, artemisinin, and analogues. Interestingly, a synergistic effect was observed with atovaquone, opening the possibility of combination therapy to combat drug resistance and mitigate toxicity [186]. Two more quassinoid metabolites of *Quassia amara*, quassin (**301**) and neo-quassin (**302**), isolated from the stem bark, inhibited the MRC-pf-20 and MRC-pf-303 strains. However, the two compounds and the control (artesunate) were less active than the crude stem bark extract (IC_50_ = 0.0025 µg/mL), and the extract was not toxic to mice. A combination treatment with artesunate and **301** or **302** showed synergism at a ratio of 1:2 and additive interaction at a 2:1 ratio; a similar result was obtained when **301** and **302** were combined [187]. These findings suggest that other metabolites in the stem bark extract might potentiate the antiplasmodial activity of these two compounds. The frequent ethnomedicinal usage, superior activity, and general lack of toxicity of *Quassia amara* extract make it an attractive herbal anti-malarial remedy worthy of further development. The acetone stem extract of *Brucea javanica* (Simaroubaceae) produced the antiplasmodial quassinoids bruceine D (**303**), and H (**304**) (Fig. 39). Bruceine D (**303**) was also isolated from the roots of the plant. Both compounds inhibited *P. falciparum* K1 strain, but **303** was also cytotoxic against cancerous human KB and NCI-H187 cells [188, 189]. *Eurycoma longifolia* (Simaroubaceae) root extract yielded the new 18-dehydro-6α-hydroxyeurycomalactone (**305**), alongside known eurycomanone (**306**) and eurycomanol (**307**), with nanomolar antiplasmodial activity [86]. The bioactivity of quassinoids has been correlated with the presence of an α,β-unsaturated ketone in ring A. Quassinoids possessing an oxymethylene bridge joining C-8 and C-13 have also shown pronounced activity [188]. These two structural features are considered essential for potent antiplasmodial activity but might also be responsible for the cytotoxicity of the compounds. Furthermore, hydroxy groups at C-11 and C-12 have been implicated in the cytotoxicity of quassinoids [189]. Thus, medicinal chemistry approaches could be employed to optimize the antiplasmodial activity and reduce cytotoxicity of this class of compounds, especially since there might be a different mechanism of action from the traditional anti-malarials.

## Polyphenols

Among the 447 isolated natural products with IC_50_ ≤ 3.0 µM reported in this review, 17.4% are polyphenols.

### Biflavonoids

**Fig. 40:**
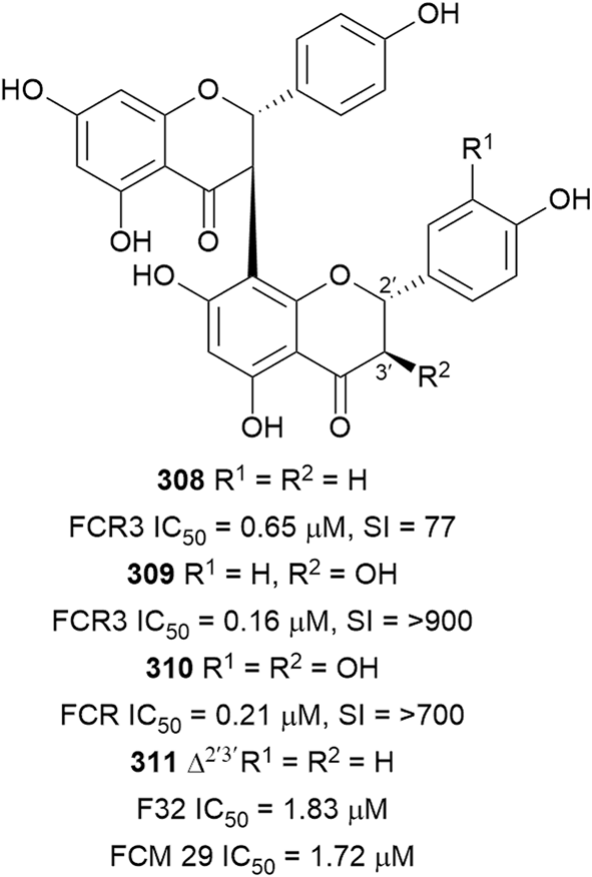
Structures of biflavonoids

Traditional healers in Congo-Kinshasa claim that the chewing of *Garcinia kola* (Clusiaceae) nuts in small quantities daily can ward off malaria. This ethnomedicinal use was validated by the activity of seed extracts against *P. falciparum* in vitro and *P. berghei* in vivo [190, 191]. Moreover, 5 µg/mL of a 70% ethanolic extract of the seed inhibited *P. falciparum* by 87%. Subsequent bioassay-guided fractionation led to the isolation of three biflavanones GB1a (**308**), GB1 (**309**), and GB2 (**310**) (Fig. 40), as the antiplasmodial principles. All three compounds exhibited sub-micromolar antiplasmodial activity against the FCR3 strain with low cytotoxicity against cancerous KB3-1 cells (SI = 77 to 900). In vivo treatment of *P. berghei*-infected mice with 100 mg/kg of the principal and most active constituent, **309**, led to 52% suppression of parasites. The compound was orally active, which is crucial for an anti-malarial lead, and no visible signs of toxicity were observed in the treated mice [192]. Another biflavonoid, volkensiflavone (**311**), with antiplasmodial activity against the F32 and FcM29 *P. falciparum* strains, was isolated from *Allanblackia floribunda* (Clusiaceae). Two structural analogues of **311** with an additional hydroxy at C-3′ on the lower flavone unit were > 10 times less active, suggesting a SAR that could be further exploited [193].

### Prenylated flavonoids

**Fig. 41:**
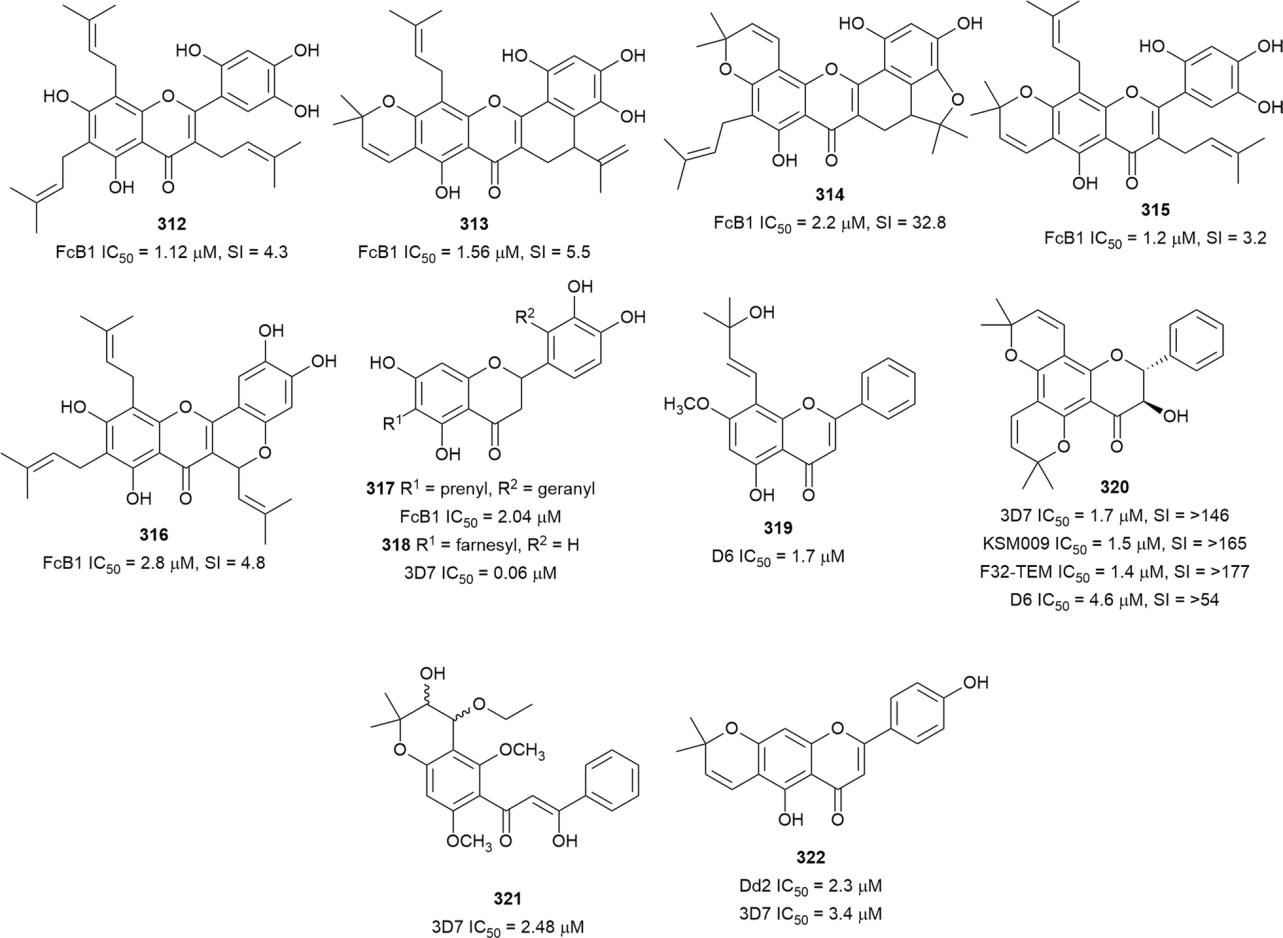
Structures of prenylated flavonoids

The ethyl acetate extract of *Artocarpus styracifolius* (Moraceae) stem bark (10 µg/mL) inhibited FcB1 *P. falciparum* by 87%. A phytochemical investigation of this extract yielded prenylated flavonoids, the new styracifolin B (**312**), together with known artonin B (**313**), artonin F (**314**), heterophyllin (**315**) and artoheterophyllin B (**316**) (Fig. 41) [194]. The compounds displayed antiplasmodial activity against FcB1 parasites with varying degrees of cytotoxicity against KB and MRC-5 cells (IC_50_ = 4.7–97 µM). An assessment of the structural features of the flavonoids shows that the two most cytotoxic compounds **312** and **315** had a prenyl chain at C-3. The C-3 prenyl side chain was transformed into a furan ring that was fused to flavonoid ring B in the most selective analogue (**314**) [194]. A crude extract from *Macaranga triloba* (Euphorbiaceae) inflorescence showed antiplasmodial activity against the 3D7 strain with an IC_50_ of 2.01 µg/mL [195]. The prenylated flavonoids nymphaeol C (**317**) and 6-farnesyl-3′,4′,5,7-tetrahydroxyflavanone (**318**) were isolated from this extract and inhibited the 3D7 parasites. The compounds also displayed varying levels of cytotoxicity to cancerous HL-60, MCF-7, and HeLa cells (IC_50_ = 1.3–23 µg/mL), suggesting some degree of selectivity in the toxicity to cellular components [195]. The antiplasmodial activity of *Tephrosia purpurea* (Fabaceae), a widely distributed medicinal plant, was validated in vitro against the D6 and W2 strains [196, 197]. A phytochemical investigation of *Tephrosia purpurea* subsp. *leptostachya* afforded a new antiplasmodial prenylflavone, (*E*)-5-hydroxytephrostachin (**319**) (Fig. 41). It was not cytotoxic to cancerous HepG2 cells at 100 µM while being moderately cytotoxic against non-cancerous human cells, indicating selectivity against D6 parasites [198]. The aerial parts of the related *Tephrosia subtriflora* were also active against *P. falciparum* 3D7, D6, and KSM 009 field isolate (IC_50_ = 4.5–11.4 µg/mL [199]. The prenylated flavonoid MS-II (**320**) was subsequently isolated from the active extract and inhibited the 3D7, KSM 009, and artemisinin-sensitive F32-TEM strains. The compound was not cytotoxic against Hep2 and Vero cells at 247.5 µM, suggesting a selective antiplasmodial activity [199]. Furthermore, the root extract of *Tephrosia aequilata*, which inhibited 3D7 *P. falciparum* by 100% at 10 µg/mL, produced the new aequichalcone C (**321**). It inhibited the 3D7 strain without being cytotoxic against HEK-293 cells up to 40 µM, indicating selectivity [200]. Another prenylated flavonoid, carpachromene (**322**), was isolated from *Flindersia pimenteliana* (Rutaceae) as one of the antiplasmodial principles, and it was not cytotoxic against HEK-293 cells at 40 µM [71].

Although many prenylated flavonoids are active against the malaria parasite, many of these compounds are also cytotoxic, and selectivity is a problem. Fröhlich et al. reported that prenylated chalcones isolated from hops (*Humulus lupulus*, Cannabaceae) interfere with haem degradation in *P. falciparum*, suggesting a possible mechanism of action [201].

### Other flavonoids

**Fig. 42:**
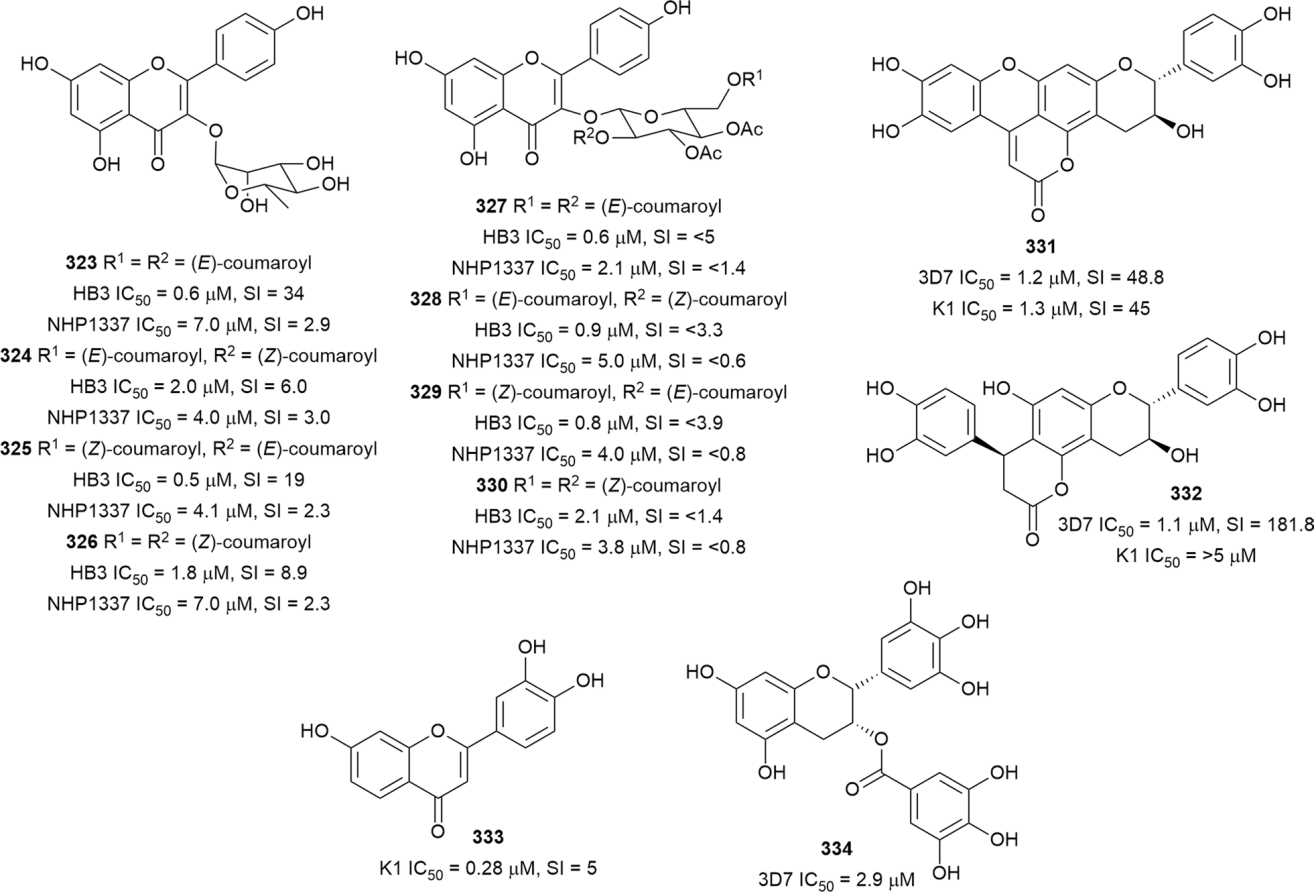
Structures of other flavonoids

Methanol supercritical fluid extraction yielded four kaempferol 3-*O*-rhamnosides (**323**–**326**) from *Platanus occidentalis* (Platanaceae) and three kaempferol 3-*O*-glucosides (**327**–**330**) from *Quercus laceyi* (Fagaceae) (Fig. 42) [158]. The glycoside esters exhibited activity against HB3 parasites but were 2–11 times less active against a multidrug-resistant NHP1337 clone. The glucosides **327**–**330** were also cytotoxic against HeLa cells, while rhamnosides **323**–**326** were more selective against HB3 parasites (SI = 6–34) [158]. An ethnobotanical survey of medicinal plants used by the people in the Comoros Islands indicated that a decoction of *Flacourtia indica* (Salicaceae) stem and leaves is used against malaria. But, extracts prepared from the aerial parts of this plant displayed only weak activity against K1 parasites (IC_50_ = 49 µg/mL) for dichloromethane and > 50 µg/mL for polar extracts [202]. However, in support of the ethnomedical use of the plant, a 95% ethanol extract of *Flacourtia indica* inhibited the 3D7 strain (IC_50_ = 0.5 µg/mL), but was inactive against K1 at the highest tested concentration (10 µg/mL). Chemical investigation of the active extract afforded six phenolic compounds (Fig. 42) of which the flavonolignans, mururin A (**331**), and catechin-[5,6-e]-4β-(3,4-dihydroxyphenyl)dihydro-2(3*H*)-pyranone (**332**) exhibited selective antiplasmodial activity. Compound **331** inhibited 3D7 and K1 *P. falciparum*, while **332** was active only against the 3D7 strain. Both compounds also inhibited β-haematin formation similar to chloroquine and H_2_O_2_-mediated heme degradation, suggesting a possible mechanism of action [203]. The known flavonoid, 3′,4′,7-trihydroxyflavone (**333**), isolated from *Albizia zygia* (Fabaceae) extract, inhibited K1 *P. falciparum* but was also cytotoxic against L6 cells [204]. (−)-Epigallocatechin-3-gallate (EGCG) (**334**), the major polyphenol in green tea (*Camellia sinensis*, Theaceae), displayed antiplasmodial activity against 3D7 *P. falciparum*. EGCG also inhibited chaperone and ATPase functions by targeting *Pf*Hsp70-1 and *Pf*Hsp70-z [205]. A previous study had shown that EGCG did not interfere with the parasite folate pathway. Moreover, the antiplasmodial activity of green tea and **334** was demonstrated previously, and EGCG was shown to have an additive effect in combination with artemisinin [206]. However, a higher IC_50_ value (37.2 µM) for EGCG against 3D7 parasites was reported in that study, even though comparable assay methods were used to evaluate the activity. The availability, low cost, and lack of toxicity of green tea coupled with the potentiating effect on the antiplasmodial activity of artemisinin could be exploited to design new artemisinin combination therapies [206].

### Coumarins and lactones

**Fig. 43:**
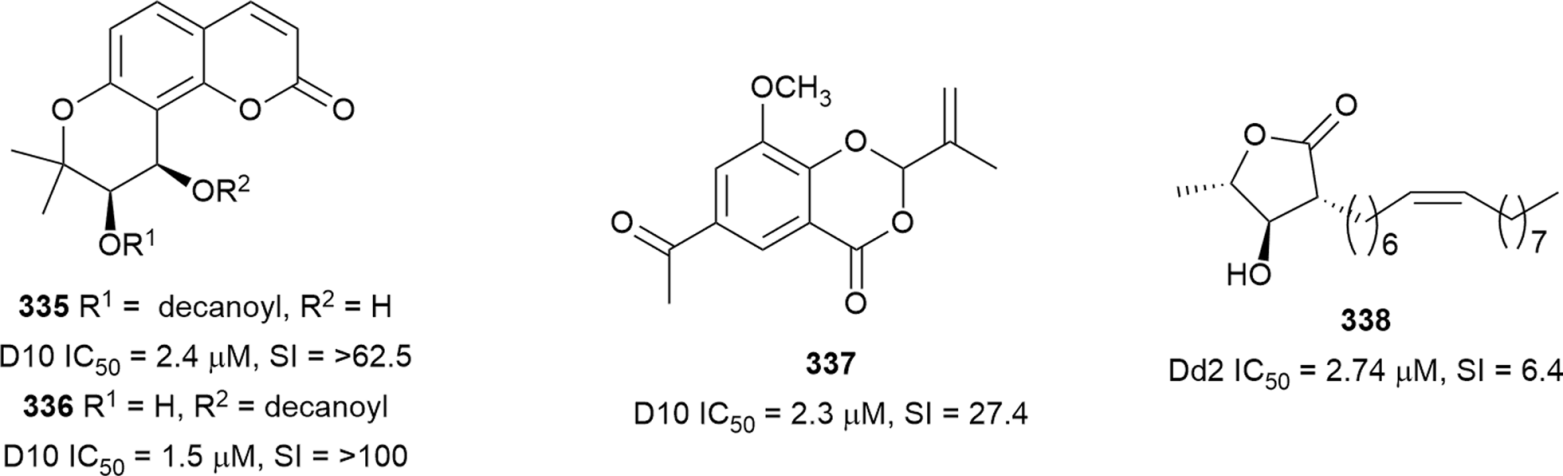
Structures of coumarins and lactones

Bioassay-guided fractionation of the rhizome extract of the Korean medicinal plant, *Angelica purpuraefolia* (Apiaceae) led to the isolation of the pyranocoumarins 3′-decanoyl-*cis*-khellactone (**335**) and 4′-decanoyl-*cis*-khellactone (**336**) as the main antiplasmodial compounds (Fig. 43). Both compounds inhibited D10 parasite strains without cytotoxicity against cancerous SK-OV-3 cells, suggesting selectivity towards the parasite [207]. The known metabolite 6-acetyl-2-isopropenyl-8-methoxy-1,3-benzodioxin-4-one (**337**), with an additional oxygen atom in the lactone ring, was also isolated as the main antiplasmodial constituent from a *Carpesium divaricatum* (Asteraceae) extract [208]. The dichloromethane extract of *Malleastrum* sp. (Meliaceae) showed antiplasmodial activity against the Dd2 strain with IC_50_ = 1.3 µg/mL. A new butanolide lactone, malleastrumolide A (**338**), was subsequently isolated from the active extract and was found to inhibit Dd2 parasites, but it was also cytotoxic against A2780 cancer cells [209].

### Phenolic acids, phenylethanoids, phenylpropanoids, and other shikimic acid-derived metabolites

**Fig. 44:**
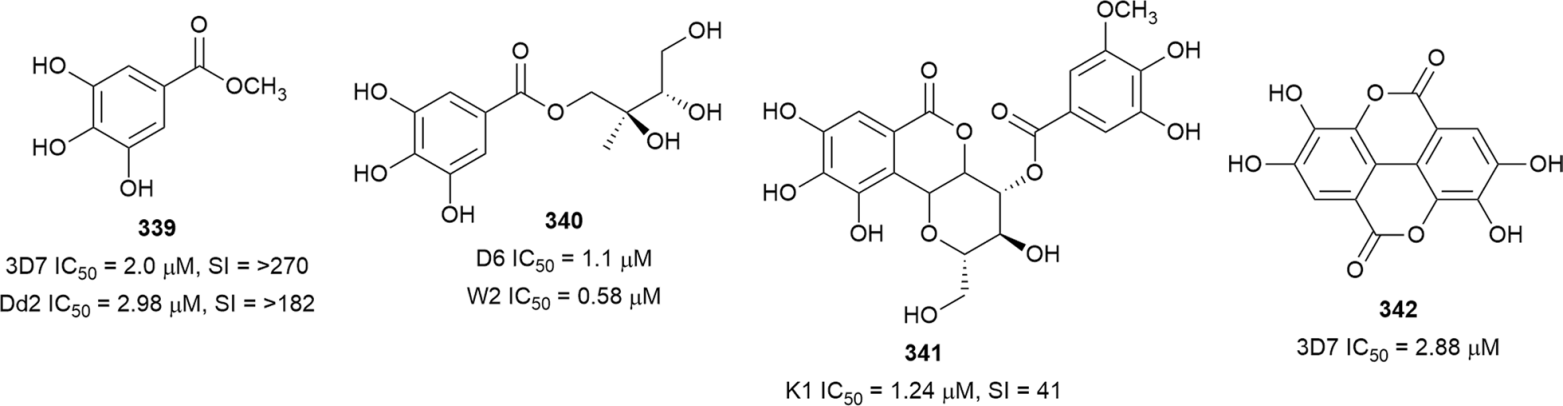
Structures of esters and lactones

The leaf extract of *Dacryodes edulis* (Burseraceae), a West African traditional medicinal plant, exhibited antiplasmodial activity against 3D7 and Dd2 parasites (IC_50_ = 6.45 and 8.62 µg/mL, respectively) [210]. Antiplasmodial screening of the stem bark extract showed that it was also active with an IC_50_ of 4.34 and 6.43 µg/mL against 3D7 and Dd2 strains, respectively. Chemical investigations of the stem bark extract afforded methyl gallate (**339**) (Fig. 44) as the most active compound against the same parasite strains without cytotoxicity against LLC-MK2 cells. Preliminary investigations showed that **339** acts on late-stage parasite trophozoites and schizonts. Compound **339** also acts in synergism with quinine but had an additive effect with artemether [211]. A new gallic acid ester, 2,3,4-trihydroxy-2-methylbutyl gallate (**340**), with activity against D6 and W2 *P. falciparum*, was isolated from the aerial parts of *Limonium leptophyllum* (Plumbaginaceae). The compound was almost twice as active against the chloroquine-resistant W2 strain as against the chloroquine-sensitive D6 strain [212]. Likewise, a galloylated norbegenin derivative, 4-*O*-(3′-methylgalloyl)norbergenin (**341**), was obtained as the most active antiplasmodial constituent of *Diospyros sanza*-*minika* (Ebenaceae) stem bark extract. Norbegenin, which was also isolated from the same extract, was 32 times less active (40.15 µM), indicating that the additional galloyl group is beneficial to the antiplasmodial activity [213]. Antiplasmodial activity was demonstrated for *Anogeissus leiocarpus* (Combretaceae) methanol bark extract (IC_50_ = 18.8 µg/mL), and ellagic acid (**342**) was isolated as the most active antiplasmodial metabolite of the extract against 3D7 *P. falciparum* [214]. The in vitro and in vivo anti-malarial activity of ellagic acid was previously demonstrated with little cytotoxicity. It also has a synergistic effect in combination with chloroquine and artesunate [215].

**Fig. 45:**
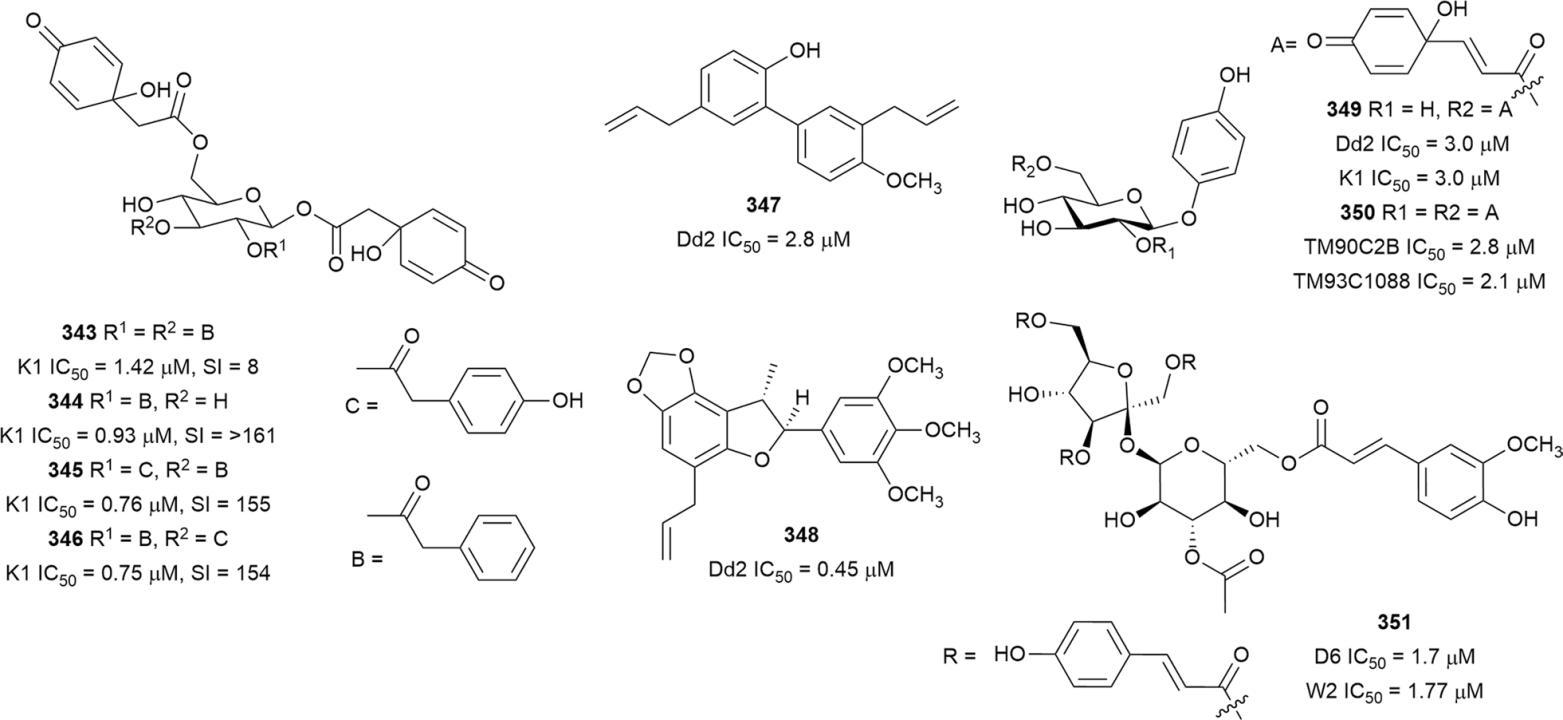
Structures of phenylethanoids and phenylpropanoids

The leaf extract of South American folkloric medicinal plant *Jacaranda glabra* (Bignoniaceae) exhibited antiplasmodial activity against the K1 strain. Bioassay-guided purification was subsequently used to isolate the phenylethanoid glucosides jacaglabrosides A–D (**343**–**346**) (Fig. 45). All four jacaranone-based glucosides (**343**–**346**) inhibited *P. falciparum* K1 strain. Compound **344**–**346** were not cytotoxic, but **343** showed cytotoxicity against L6 cells [216].

The ethyl acetate fraction of *Magnolia grandiflora* (Magnoliaceae) fruit and twig extracts inhibited Dd2 *P. falciparum* (IC_50_ = 10 µg/mL). The known 4′-*O*-methyl honokiol (**347**) was the most active among the isolated bioactive neolignans against the Dd2 strain. The position and number of substituents on the neolignan aromatic rings affected the bioactivity. Honokiol, which is structurally similar to **347** but is not methoxylated, was six times less active (IC_50_ = 16.5 µM). However, magnolol, which is isomeric to honokiol, was only slightly less active (IC_50_ = 3.4 µM) than **347**, while an additional methoxy group on magnolol (3-methoxymagnolol) resulted in a 30 fold decrease in activity [217]. More SAR studies are needed to fully understand the effect of substituents on the antiplasmodial activity of simple neolignans. A new dihydrobenzofuranoid neolignan, ococymosin (**348**), was obtained from the antiplasmodial hexane stem extract (IC_50_ = 1.25 µg/mL) of *Ocotea cymosa* (Lauraceae) as the most active metabolite against the Dd2 strain [218]. The antiplasmodial screening of a library of marine- and plant-derived extracts showed that *Grevillea* (Poorinda Queen, Proteaceae) leaf and twigs extract displayed antiplasmodial activity. Subsequent bioassay-guided purification of the active extract yielded the hemiquinone-containing phenylpropanoid glycosides robustasides D (**349**) and G (**350**) (Fig. 45) as the main antiplasmodial constituents. Both compounds were more active against chloroquine-resistant *P. falciparum* than to chloroquine-sensitive strains. Compound **350** was more active against the Dd2 and K1 strains, whereas **349** was more active against the multidrug-resistant TM93-C1088 and TM90-C2B strains. Treatment of *P. berghei* infected mice with 32 mg/kg dose of **350** twice a day for 4 days suppressed parasitaemia by 95% without evidence of toxicity [219]. The phenylpropanoid glycoside vanicoside F (**351**), isolated as a major constituent of *Polygonum hydropiper* (Polygonaceae) aerial parts, also inhibited *P. falciparum* D6 and W2 strains without cytotoxicity against Vero cells [220].

**Fig. 46:**
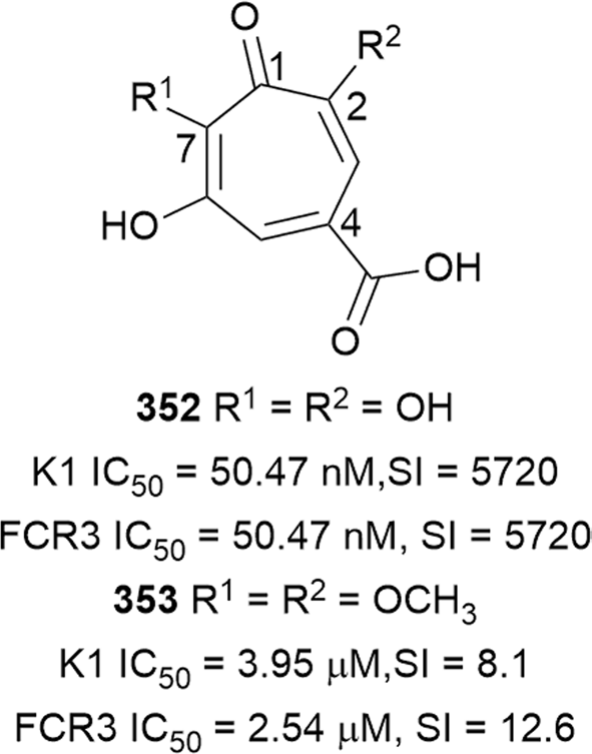
Structures of tropones

The culture broth of the *Penicillium* sp. FKI-4410 (*Penicillium viticola* sp. nov) fungus afforded some tropolones, among which puberulic acid (**352**) and a new derivative viticolin B (**353**) (Fig. 46) showed activity. Compound **352** showed potent nM activity against K1 and FCR3 parasites and was not cytotoxic against MCR-5 cells, whereas **353** was less active and selective. Treatment of *P. berghei*-infected mice with 2 mg/kg of **352** for 3 days suppressed parasitaemia by 69%, comparable to chloroquine and artesunate. A preliminary SAR of the tropone compounds suggests that a hydroxy group at C-7 and a methoxy group at C-2 are important for activity while a carboxylic acid function at C-4 improves selectivity [221].

**Fig. 47:**
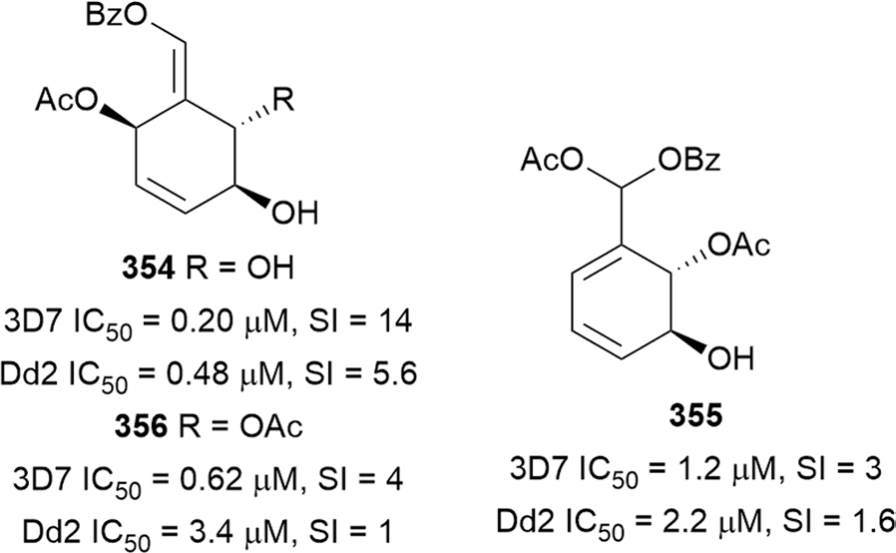
Structures of oxygenated cylcohexenones

A phytochemical reinvestigation of *Cleistochlamys kirkii* (Annonaceae) leaf extract led to the isolation of new polyoxygenated cyclohexenones cleistodienediol (**354**), cleistodienol B (**355**), and the known cleistodienol A (**356**) (Fig. 47). The configuration of the exocyclic double bond in **356** was revised based on the similarity of the nuclear magnetic resonance spectroscopic and specific rotation data with those of **354**. The absolute configuration of **354** was established by X-ray diffraction analysis, which showed that it adopts a half-chair conformation. All three compounds inhibited 3D7 and Dd2 *P. falciparum* strains but were also cytotoxic against noncancerous HEK-293 and cancerous MDA-MB-231 cells. The additional acetylation of compound **356** led to a decrease in activity and selectivity, suggesting a SAR role that could be further explored [222].

**Fig. 48:**
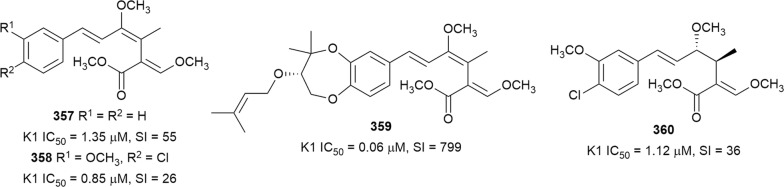
Structures of strobilurins

The strobilurins are used commercially in agriculture as fungicides. An investigation of the extracts from the fungus *Favolaschia tonkinensis* led to the isolation of the β-methoxyacrylate derivatives, 9-methoxystrobilurins A, B, and G (**357**–**359**), and oudemansin B (**360**) (Fig. 48). The compounds were active against K1 *P. falciparum* strain and weakly cytotoxic against Vero cells [223].

### Xanthones

**Fig. 49:**
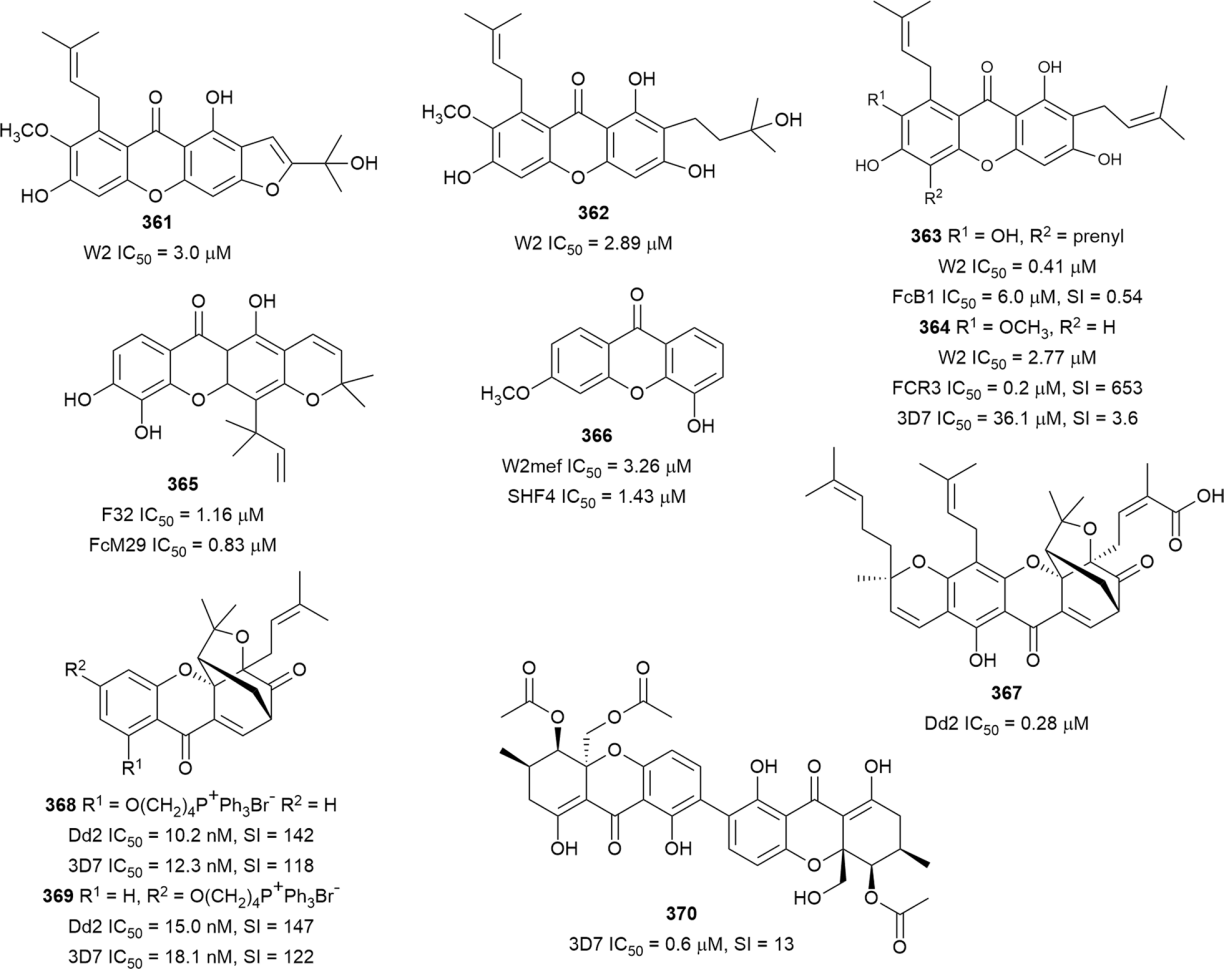
Structures of xanthones

The extract from fruit pericarp of *Pentadesma butyracea* (Clusiaceae) exhibited antiplasmodial activity against W2 parasites with IC_50_ = 1.8 µg/mL. Bioassay-guided purification of the extract yielded the new prenylxanthone pentadexanthone (**361**), together with the known cratoxylone (**362**), garcinone E (**363**) and α-mangostin (**364**) (Fig. 49). All four xanthones were active against *P. falciparum* W2 strain [224]. The antiplasmodial activity of **363** from the same plant against FcB1 was previously reported, but it was also cytotoxic against MCF-7 cells [225]. Xanthone **364** was isolated from *Garcinia mangostana* (mangosteen, Clusiaceae) husk and it inhibited FCR3 *P. falciparum* but, surprisingly, it was much less active against the 3D7 strain. Intraperitoneal treatment of *P. berghei* infected mice with **364** at 100 mg/kg suppressed parasitaemia by 80%. Oral treatment produced only 27% suppression. The compound was not cytotoxic to U-937 cells (LC_50_ = 130.6 µM), but caused haemolysis of red blood cells at 69.7 µM [226]. Another prenylated xanthone, macluraxanthone (**365**), was isolated from an antiplasmodial extract of *Allanblackia floribunda* (Clusiaceae) and had activity against the F32 and FcM29 parasites [193]. *Hypericum lanceolatum* (Hypericaceae) is a multipurpose medicinal plant that is used by the people in the southwest province of Cameroon to treat fever [227]. In an effort to rationalize this ethnomedicinal use scientifically, the stem bark ethyl acetate fraction was assayed and antiplasmodial activity was observed against the W2mef parasite, IC_50_ = 5.02 µg/mL. A bioassay-guided purification of the extract afforded 5-hydroxy-3-methoxyxanthone (**366**) as the most active compound. It inhibited *P. falciparum* SHF4 field isolate but was slightly less active against the W2mef strain. The compound was not cytotoxic against LLC-MK2 cells at the highest concentration tested (100 µg/mL). Interestingly, the 3-hydroxy-5-methoxy isomer of **366** was inactive, suggesting that substitution pattern of the xanthone skeleton play a crucial role in the bioactivity [228]. *Garcinia* species produce a unique group of metabolites called caged *Garcinia* xanthones (CGXs) in which the C-ring of the xanthone skeleton has a prenyl substituent that has been transformed to form a tricyclic ring. It was observed that these compounds with interesting bioactivities tend to localize in cell mitochondria and cause damage to it [229]. Gambogic acid (**367**) (Fig. 49), the representative CGX, was isolated from *Garcinia* (Gamboge) resin and exhibited antiplasmodial activity against Dd2 parasites with a sub-micromolar IC_50_ value. Chemically modified analogues **368** and **369** that incorporated a triphenylphosphonium group into the CGX skeleton displayed low nanomolar activity against parasite trophozoites and schizonts, whereas replacement of the CGX scaffold with a planar xanthone structure led to a reduction in activity. These observations indicate the beneficial effect of conjugating a triphenylphosphonium moiety to the CGXs and the vital role of the CGX scaffold in the activity of these compounds. The CGXs caused mitochondrial fragmentation and morphological changes within the parasite but did not affect the mitochondrial electron transport chain. This suggests a different mechanism of action from other anti-malarial drugs, such as atovaquone, that target parasite mitochondria. The cytotoxicity of the compounds against HEK293 cells was in the µM range, making them selectively toxic to the parasite at the active concentration (SI > 100) [230]. A tetrahydroxanthone dimer, dicerandrol D (**370**), from the endophytic fungus *Diaporthe* sp. (CY-5188) also inhibited *P. falciparum* with a sub-micromolar IC_50_ value and moderate cytotoxicity. The bioactivity is influenced by the configuration, and the C-12 epimer, dicerandrol B, was inactive [231].

### Phloroglucinol derivatives

**Fig. 50:**
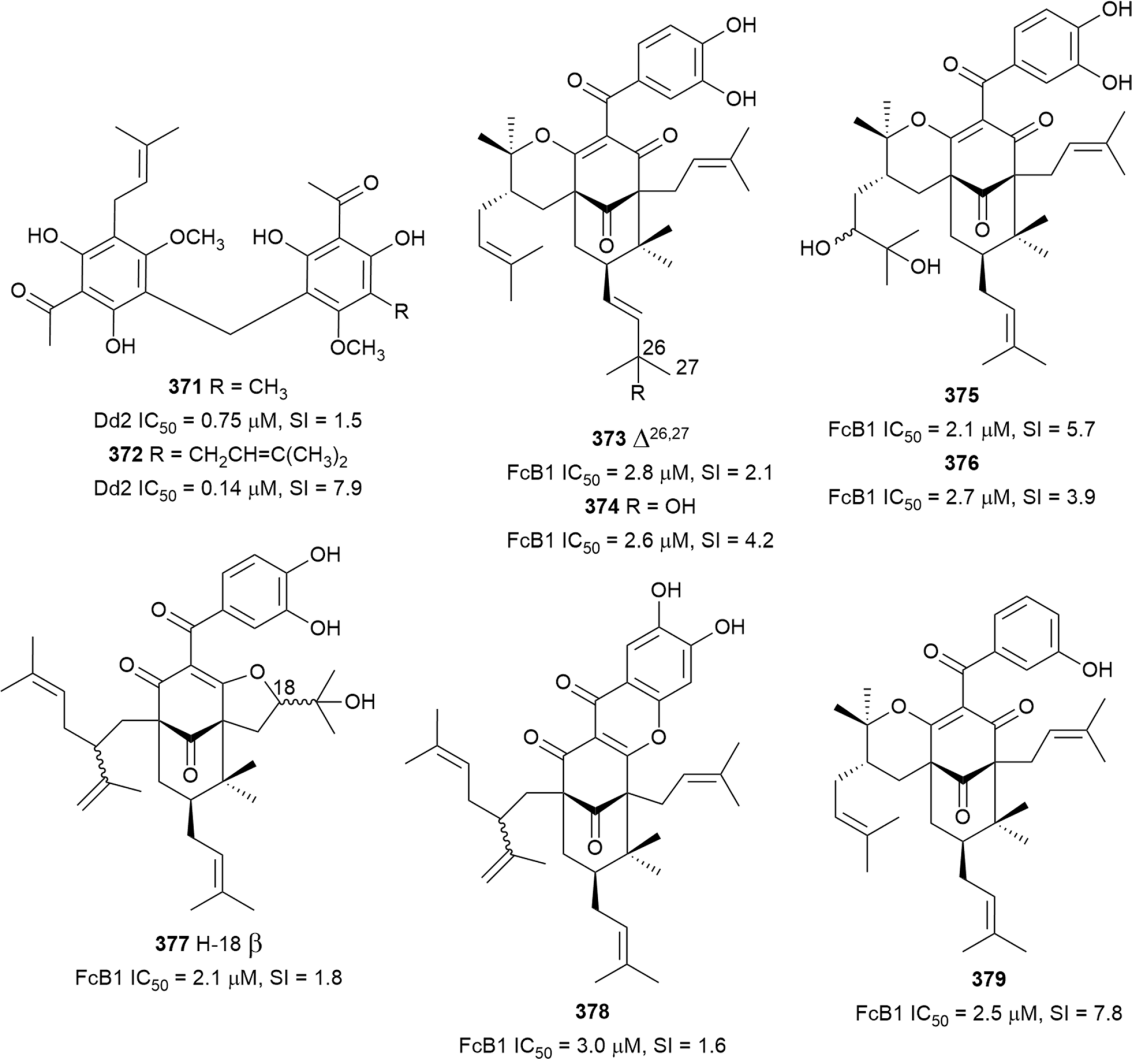
Structures of phloroglucinol derivatives

Two new dimeric phloroglucinols, mallotojaponins B (**371**) and C (**372**) (Fig. 50), were obtained from *Mallotus oppositifolius* (Euphorbiaceae) leaf and inflorescence ethanol extract. Both compounds were active against the Dd2 parasite and cytotoxic against cancerous A2780 cells. Also, the compounds displayed cytocidal activity against the HB3 (LD_50_ 14.6 and 0.81 µM for **371** and **372**, respectively) and Dd2 strains (LD_50_ = 6.7 and 0.8 µM for **371** and **372**, respectively). Mallotophenone, isolated from the same plant, lacked the prenyl chain of the mallotojaponins and was inactive. Also, **372**, with two prenyl chains, was more active than the monoprenylated **371**, suggesting that prenylation is essential for the antiplasmodial activity of these compounds [232]. The synthesis of mallotojaponin C (**372**) was demonstrated in a three-step procedure [233]. Seven new polycyclic polyprenylated acylphloroglucinols (PPAPs), symphonones A (**373**), C–E (**374**–**376**), G (**377**), H (**378**), and 14-deoxy-7-epi-isogarcinol (**379**) have been isolated from *Symphonia globulifera* (Clusiaceae) root bark extract. It was previously observed that the extract inhibited *P. falciparum* by 97% at a concentration of 10 µg/mL. These benzophenone derivatives were active against FcB1 *P. falciparum* but were also cytotoxic against MRC-5 cells [234].

### β-Triketones

**Fig. 51:**
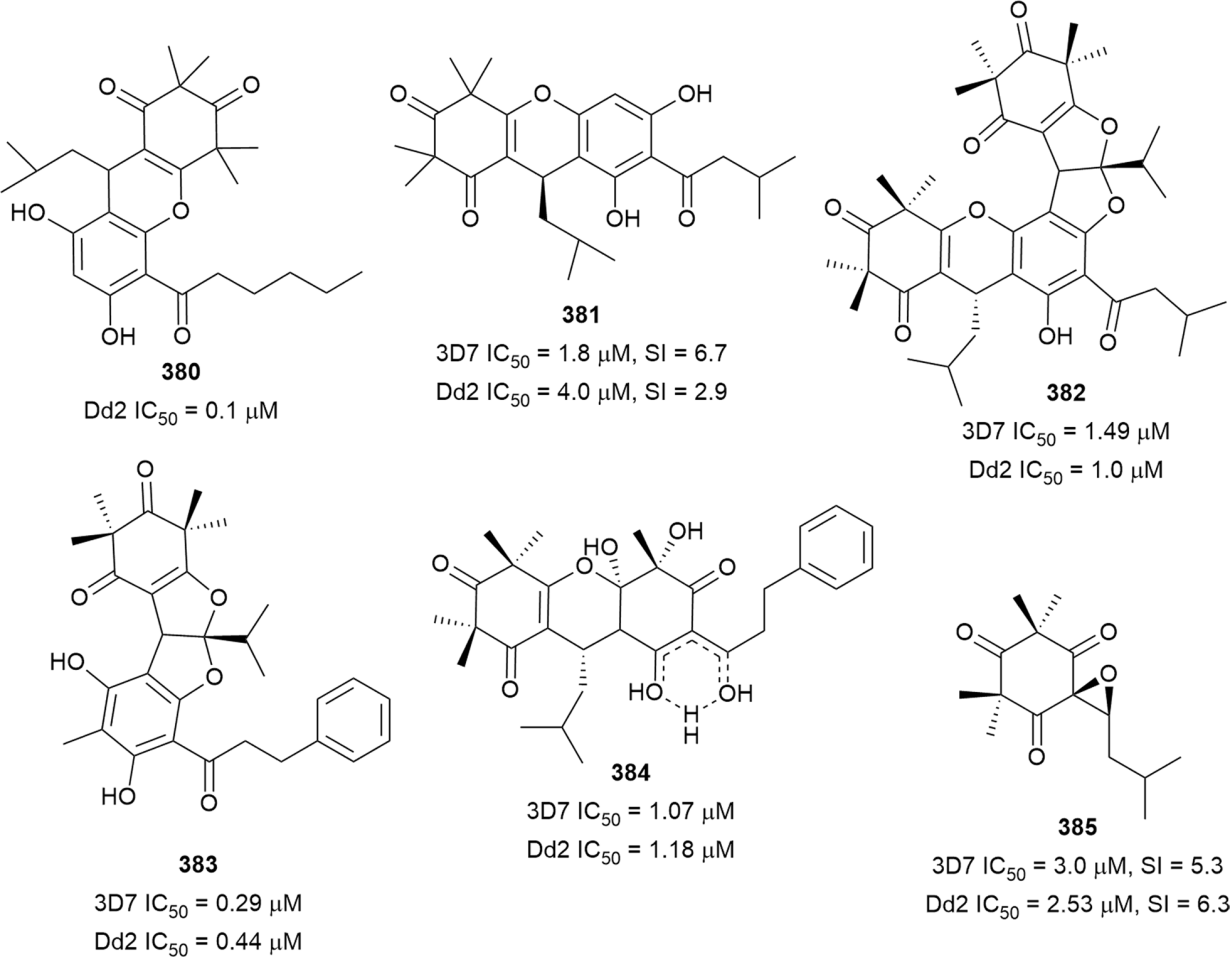
Structures of β-triketones

This interesting antiplasmodial scaffold is present in compounds isolated from plants of the Myrtaceae. These compounds have been isolated as adducts of phloroglucinol and terpenes, as well as simple acylated syncarpic acid derivatives. A new phloroglucinol β-triketone rhodomyrtosone F (**380**) (Fig. 51) from *Syncarpia glomulifera* (Myrtaceae) stem bark extract, displayed sub-micromolar inhibition of Dd2 *P. falciparum* and only inhibited HEK293 cells by 58% at 50 µM, suggesting selective toxicity to parasites [235]. Likewise, rhodomyrtone (**381**), from the flower extract of *Angophora woodsiana* (Myrtaceae), was active against 3D7 and Dd2 parasites but moderately cytotoxic [236]. Tomentosone A (**382**), with a novel hexacyclic ring system that features a bisfurano moiety, was isolated from the dichloromethane extract of *Rhodomyrtus tomentosa* leaves (Myrtaceae). The compound inhibited 3D7 and Dd2 parasites without being cytotoxic against HEK293 cells at 40 µM [237]. Watsonianones B (**383**) and C (**384**), from the flowers of *Corymbia watsoniana* (Myrtaceae), have also shown antiplasmodial activity [238]. Watsonianone C is the first 4,4a,9,9a-tetrahydro-2*H*-xanthene-1,3,5,7(6*H*,8*H*)-tetraone to be reported while watsonianone B possesses the rare bisfurano moiety present in **382** and is only the fourth fused bisfurano-β-triketone to be reported. Compounds **383** and **384** exerted antiplasmodial activity on the parasite ring stage, with **383** acting predominantly on early ring stage trophozoites. Tomentosone A (**382**), with an additional syncarpic acid moiety and an isobutyl instead of the ethylphenyl group, was less active than **383**. The beneficial role of the ethylphenyl over the isobutyl chain was further demonstrated by the activity of rhodomyrtosone A, which was 50 times less than that of **383** [238]. Woodsianone B (**385**), a simple acylated syncarpic acid derivative with an epoxide-containing isopentyl side chain, was also obtained from *Angophora woodsiana* and inhibited the 3D7 and Dd2 parasites [236]. Unfortunately, the terpene-β-triketone adducts demonstrated only weak antiplasmodial activity, and this was attributed to poor water solubility of the compounds. Indeed, the more water-soluble analogues have shown better activity. It is plausible that improving the water solubility might lead to more active compounds. The β-triketone pharmacophore can be considered as a novel antiplasmodial scaffold [236].

### Other polyphenols

**Fig. 52:**
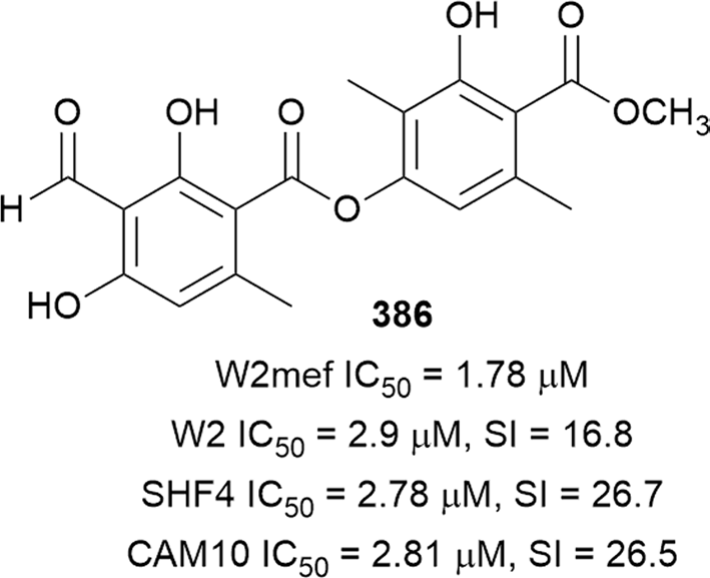
Structure of atranorin

Atranorin (**386**) (Fig. 52) was isolated from the antiplasmodial *Kigelia africana* (Bignoniaceae) bark extract and inhibited the CAM10, SHF4, and W2 parasite strains. The polyphenolic depside **386** also inhibited W2mef parasites and showed synergism in combination with artemether [117, 118].

## Quinones and polyketides

### Anthraquinones

**Fig. 53:**
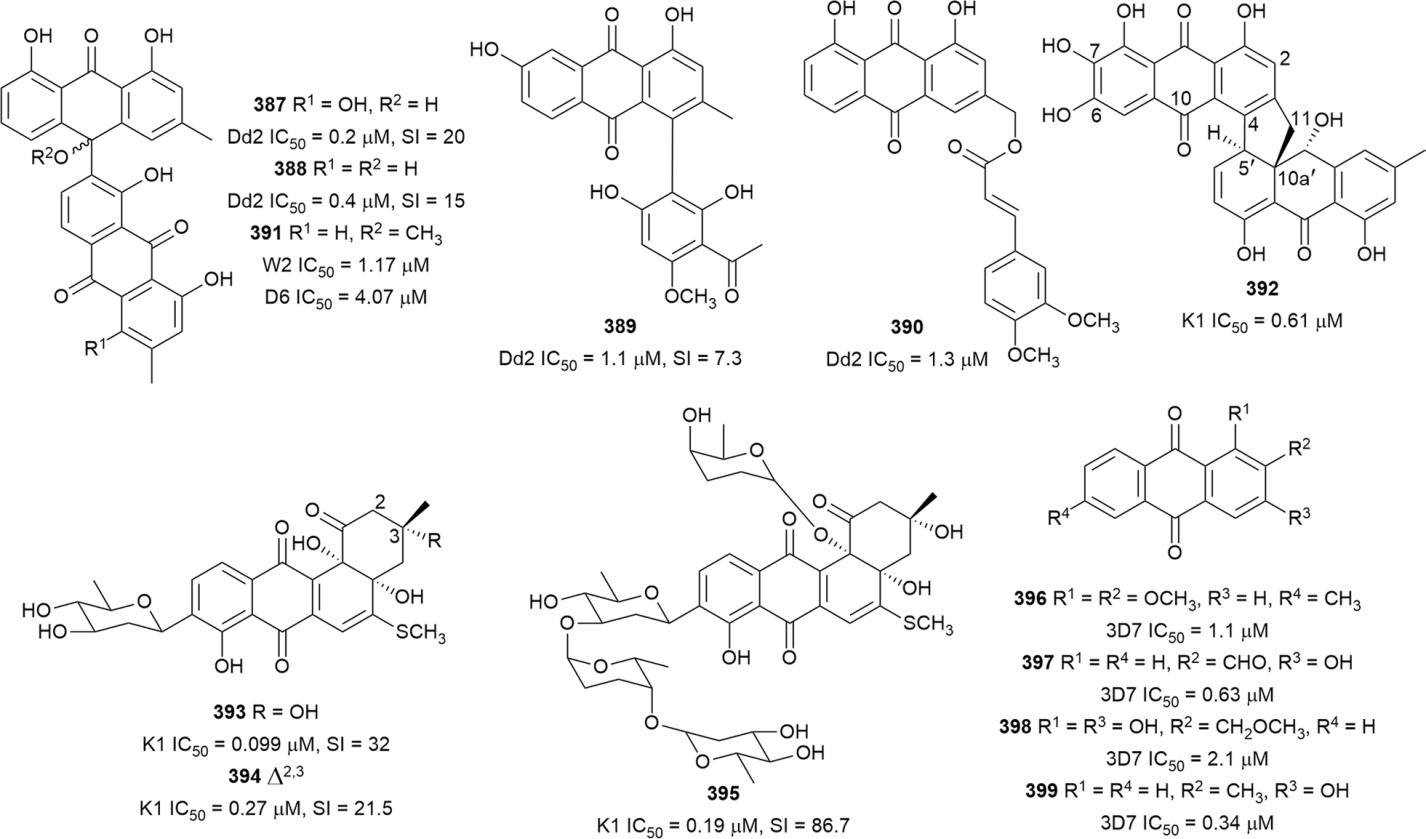
Structures of anthraquinones

An antiplasmodial screening of 6900 extracts identified *Kniphofia ensifolia* (Asphodelaceae) dichloromethane extract as active against Dd2 parasites (IC_50_ = 6 µg/mL) [239]. Bioassay-guided fractionation afforded the bisanthraquinones chryslandicin (**387**) and 10-(chrysophanol-7-yl)-10-hydroxy-chrysophanol anthrone (**388**), and the phenylanthraquinone, knipholone (**389**) as active principles (Fig. 53). The compounds displayed activity against Dd2 *P. falciparum* but were also moderately cytotoxic to cancerous A2780 cells [239]. Inhibition of the 3D7 strain was previously reported for all the three compounds, but **387** and **388** were not cytotoxic against KB cells [240]. Aloe-emodin, which was also isolated from *Kniphofia ensifolia*, was less active against the Dd2 parasites (IC_50_ = 58 µM) and did not exhibit antiproliferative activity against A2780 cells. However, a semi-synthetic derivative of aloe-emodin, the 3,4-di-*O*-methylcaffeoyl ester **390**, potently inhibited *P. falciparum* without being cytotoxic [239]. The crude extract of *Kniphofia foliosa* root also inhibited *P. falciparum* W2 and D6 strains with IC_50_ = 11.29 and 8.92 µg/mL, respectively. A reinvestigation of the extract yielded a new bisanthraquinone, 10-(chrysophanol-7-yl)-10-methoxy-chrysophanol anthrone (**391**), and the compound was more active against the chloroquine-resistant W2 strain than against the D6 strain [241]. The leafhopper pathogenic fungus *Torrubiella sp.* BCC 28517 also produced a dimeric anthraquinone, torrubiellin B (**392**), with activity against K1 *P. falciparum*. The new compound, which had rare C-4–C-5′ and C-11–C-10a′ linkages, was cytotoxic against cancerous human KB, NCI-H187, and MCF-7 cells. The structural analogue torrubiellin A, with two less hydroxy groups (C-6 and C-7), was ten times less active against parasites [242]. Antiplasmodial screening of crude extracts of marine actinomycetes from Thailand identified *Streptomyces sp.* BCC45596 to have potent activity with IC_50_ = 1.45–3.56 µg/mL. Bioassay-guided purification of the extracts led to the isolation of two new *C*-glycosylated benz[α]anthraquinones, urdamycinone E (**393**) and G (**394**), and the known urdamycin E (**395**). Sub-micromolar antiplasmodial activity was obtained for these compounds against the K1 strain. However, the compounds also showed antiproliferative activity against cancerous KB, MCF-7, and NCI-H187 cells, but were less cytotoxic against non-cancerous Vero cells [243]. The antiplasmodial activity of four more anthraquinones (**396**–**399**) from an active root extract of *Rennellia elliptica* (Rubiaceae) was demonstrated [244].

### Naphthoquinones

**Fig. 54:**
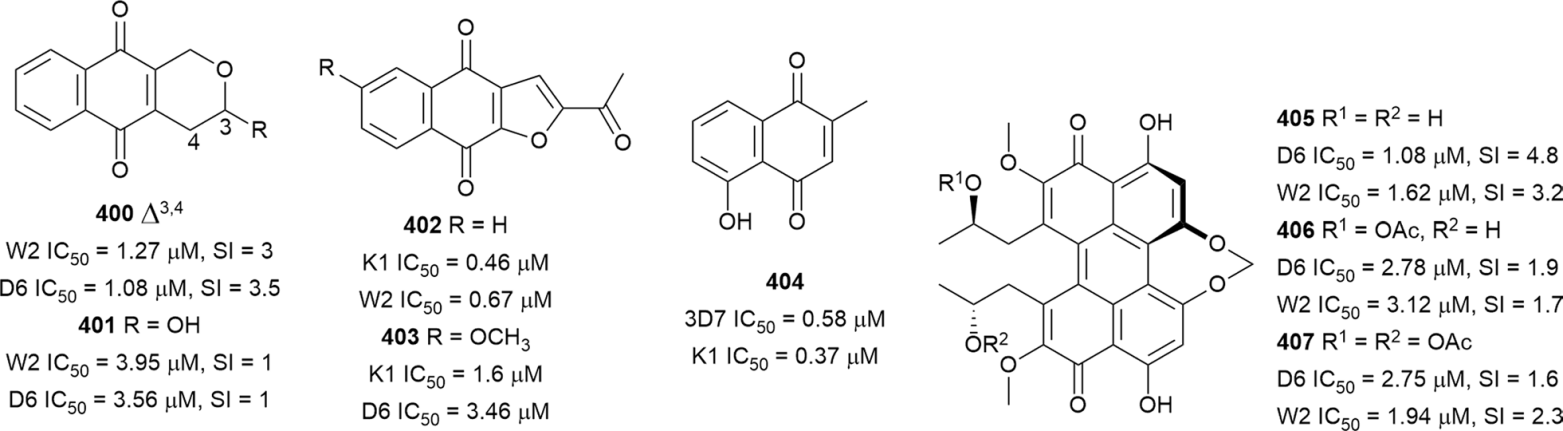
Structures of other quinones

The leaf extract of *Pentas longiflora* (Rubiaceae), which is used in Kenyan folk medicine to treat malaria, was active against *P. falciparum* [245]. Antiplasmodial investigation of the root extract showed a better activity against the W2 and D6 parasites than the leaf extract (IC_50_ = 0.93 and 0.99 µg/mL, respectively). Phytochemical investigation yielded the pyranonaphthoquinones pentalongin (**400**) and psychorubrin (**401**) (Fig. 54) with antiplasmodial activity against W2 and D6 strains. However, both compounds were also cytotoxic [246]. The ethyl acetate extract of *Markhamia tomentosa* (Bignoniaceae) stem bark showed activity against the W2 and K1 strains (IC_50_ = 1.46 and 2.81 µg/mL, respectively). Two furanonaphthoquinones (**402** and **403**) were isolated from the extract and inhibited the W2 and K1 parasites. However, **402** and **403** were also cytotoxic against L6 cells [247]. Plumbagin (**404**) is the major phytochemical in the extracts of several *Plumbago* species (Plumbaginaceae), including *Plumbago indica* and *Plumbago zeylanica*, with anti-malarial activity [248, 249]. Plumbagin (**404**) inhibited 3D7 and K1 *P. falciparum* strains and suppressed parasitaemia in *P. berghei*-infected mice by 41% after treatment (25 mg/kg body weight). Acute and subacute toxicity was observed in mice after oral administration of **404** above 100 and 25 mg/kg body weight, respectively. The relatively poor in vivo anti-malarial activity of **404** might be due to low bioavailability in living cells [250].

### Perylenequinones

The perylenequinones cercosporin (**405**), 14-*O*-acetylcercosporin (**406**), and di-*O*-acetylcercosporin (**407**) (Fig. 54) were isolated from the culture medium of the plant pathogenic fungus, *Septoria pistaciarum*. Compound **405** was also obtained from an extract of the endophytic fungus *Mycosphaerella* sp. F2140. The phytotoxins **405**–**407** inhibited *P. falciparum* D6 and W2 strain but were also cytotoxic against MCF-7 and Vero cells. Interestingly, a new cercosporin analogue with a hydroxy and a methoxy group instead of the methylenedioxy bridge in **405**–**407** was inactive. This suggests a SAR role at these two positions that could be exploited for optimized antiparasitic activity [251, 252].

### Other polyketides

**Fig. 55:**
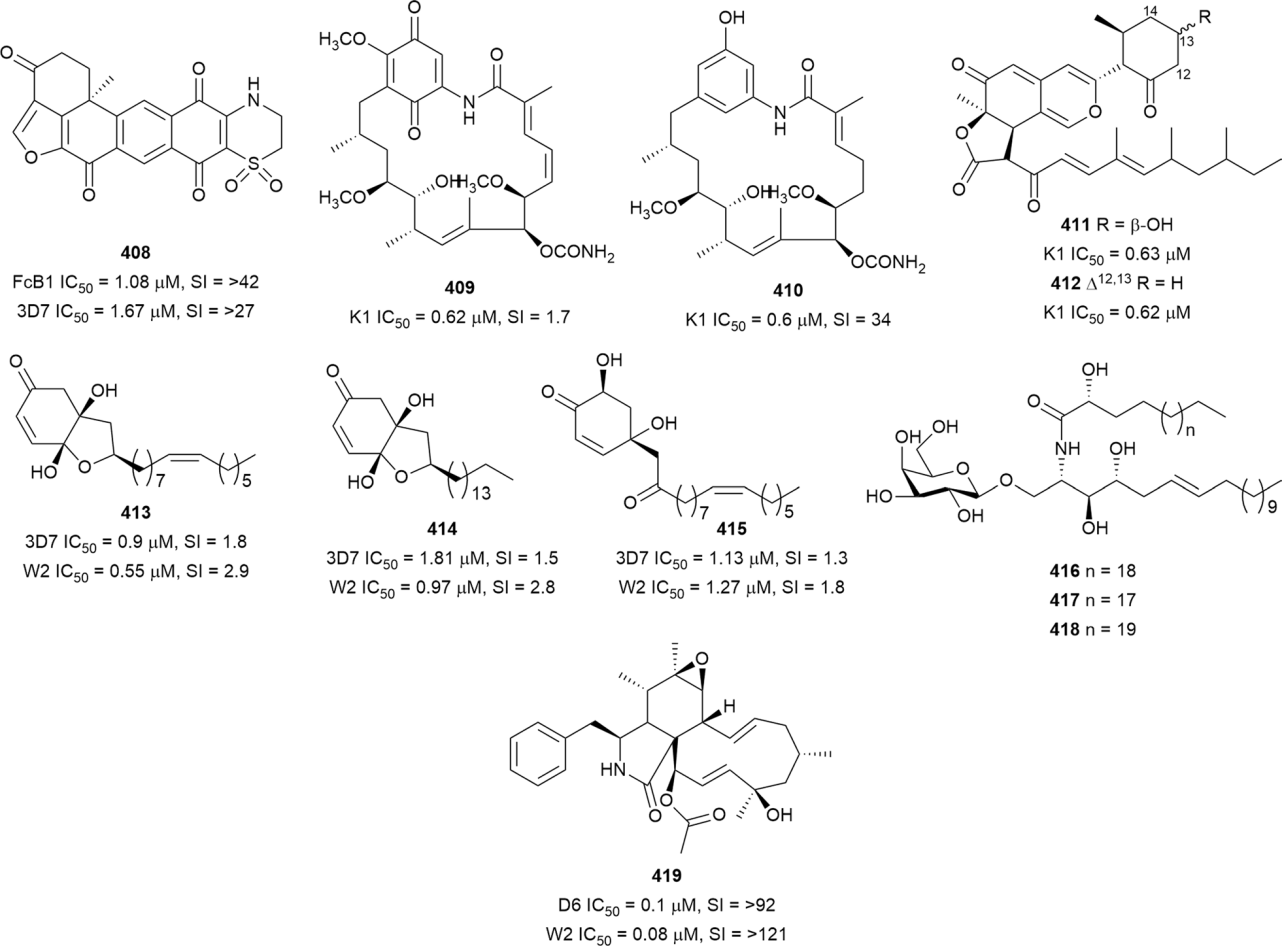
Structures of other polyketides

The Solomon Island-sourced marine sponge *Xestospongia testudinaria* produced a halenaquinone-type polyketide 3-ketoadociaquinone A (**408**) (Fig. 55), which selectively inhibited the FcB1 and 3D7 strains. Halenaquinone, which differs from **408** only in the absence of the dioxothiazine ring, was inactive, indicating that a dioxothiazine ring is necessary for the antiplasmodial activity of the compound [253]. The crude extract of terrestrial *Streptomyces* sp. BCC71188 from Thailand inhibited *P. falciparum* (IC_50_ = 0.19 µg/mL). Two benzoquinone polyketides, geldanamycin (**409**) and 17-demethoxyreblastatin (**410**), were isolated from the extract and showed antiplasmodial activity against the K1 strain. The quinone moiety in **409** appears to contribute to the cytotoxicity since the structural analogue **410** with a phenol group instead of the quinone was more selective against the parasite [254]. Two new antiplasmodial azaphilones, longirostrerone A (**411**) and C (**412**), were isolated from the ethyl acetate extract of the Thailand soil fungus *Chaetomium longirostre*. Both compounds inhibited K1 *P. falciparum* but were also cytotoxic against cancerous KB cells. Compound **411** was also cytotoxic against NCIH-187 and MCF-7 cancer cells, whereas **412** was inactive, indicating selective cytotoxicity [255]. Longirostrerone D, which differs from **411** in the configuration of the isochromeno-lactone ring junction and a double bond in the butyrolactone ring, was inactive. Likewise, longirostrerone B, which lacks the lactone moiety, was less active. These observations suggest that the nature of the six-membered ring attached to isochromene, the lactone ring, and the configuration of the compounds play a role in the activity, and further modifications might produce analogues that are more selective.

Three other new cyclohexenones, poupartones A–C (**413**–**415**) were isolated from the ethyl acetate leaf extract of *Poupartia borbonica* (Anacardiaceae) by bioassay-guided fractionation [256]. The extract was previously shown to inhibit the 3D7 strain of *P. falciparum* with IC_50_ = 3.28 µg/mL. The compounds were active against W2 and 3D7 parasites but were also cytotoxic against HeLa and WI38 cells. No haemolytic activity was observed with the compounds, indicating that the antiplasmodial activity was due to direct action on the parasites. Treatment of *P. berghei*-infected mice with **413** (15 mg/kg/day for 4 days) led to 69.5% parasite suppression, but it was also toxic to the mice. A toxicity assay of **413** using the zebrafish embryo model indicated that the compound might be cardiotoxic [256].

The Senegalese marine sponge *Axinyssa djiferi* produced an antiplasmodial mixture containing three glycosphingolipids axidjiferosides A–C (**416**–**418**) (Fig. 55). The mixture represented 2.16% of the dried sponge lipid content, while axidjiferoside A (**416**) constituted 60% of the mixture. The compounds were identified as homologs of β-galactopyranosylceramide, containing a 2-amino-1,3,4-trihydroxy-octadecene sphingoid base. Moreover, the fatty acid methyl ester of the major compound (**416**) was identified as 2-hydroxytetracosanoic acid. The mixture exhibited antiplasmodial activity against the FcB1 strain (IC_50_ = 0.53 µM) and was not cytotoxic against a panel of cancerous cells. The activity also appeared to be parasite selective because *Leishmania donovani* and *Trypanosoma brucei* were not susceptible to the mixture [257]. The endophytic fungus *Diaporthe miriciae* produced epoxycytochalasin H (**419**) with potent antiplasmodial activity against D6 and W2 parasite strains. The compound was not cytotoxic against Vero cells, indicating that the toxicity to the parasite is selective [258].

## Macrocycles

### Macrolides

**Fig. 56:**
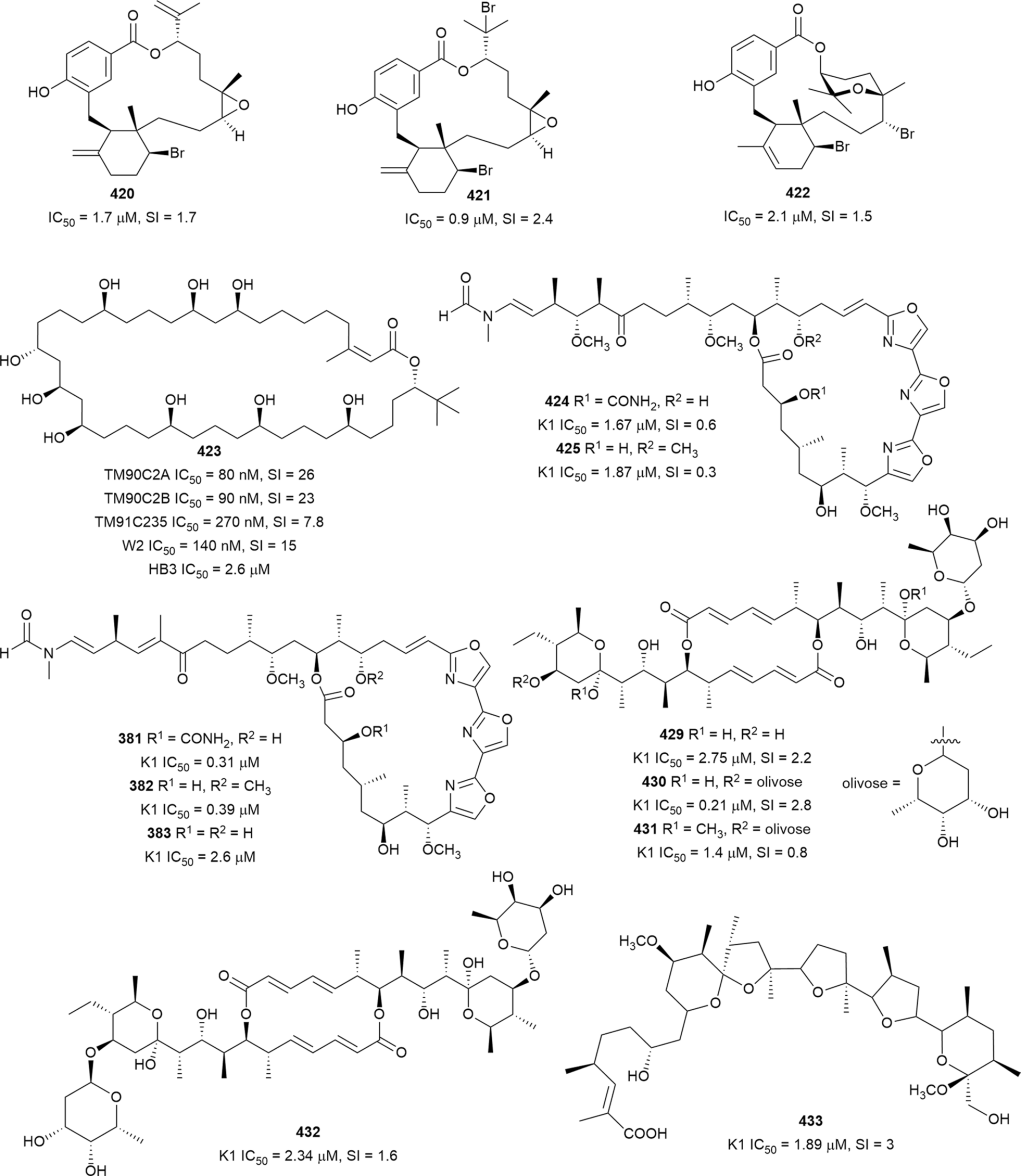
Structures of macrolides

The bromophycolides are diterpene-benzoate macrolides that were isolated from the Fijian marine red alga *Callophycus serratus* and had antiplasmodial activity [259]. A reinvestigation of *Callophycus serratus* afforded bromophycolides R (**420**), S (**421**), and U (**422**) (Fig. 56) with antiplasmodial activity and moderate cytotoxicity against cancerous cells [260]. Bromophycolides with 15- and 16-membered rings have shown potent antiplasmodial activity. No significant influence of the lactone ring size on activity was observed between 15- and 16-membered lactone rings. The macrolide ring appears to improve bioactivity, considering that non-macrocyclic diterpene-benzoic acids and diterpene-phenols were less active. Furthermore, isolation of the less active 14-membered ring callophycolide A (IC_50_ = 5.4 µM), which lacked the bromine atoms and a cyclohexane ring, showed that these features are not essential but improve the potency [259, 261]. Some bromophycolides target haem crystallization, suggesting that the mechanism of action involves the inhibition of haemozoin formation [262]. Bastimolide A (**423**), a 40-membered ring polyhydroxy macrolide with 10 stereocentres and a rare *tert*-butyl terminus, was isolated from marine cyanobacterium, *Okeania hirsuta* (PAB-19MAY11-4) [242]. The planar structure and absolute configuration of the macrocyclic lactone, which consist of a 1,3-diol, one 1,3,5-triol, and six 1,5-diols, were established by X-ray crystallography. It was hypothesized that the rare *tert*-butyl group near the lactone ester in the bastimolide structure protects the lactone ring against hydrolysis. The compound inhibited multidrug-resistant strains TM90-C2A, TM90-C2A, TM91-C235, and W2 with nanomolar IC_50_ values. Surprisingly, the chloroquine-sensitive HB3 strain was less susceptible to **423** (IC_50_ in µM). Additionally, the semi-synthetic (2*E*)-isomer was more active against the HB3 strain than the (2*Z*) natural product. Further investigation of *Okeania hirsuta* yielded bastimolide B, in which lactonization produced a 24-membered ring and a highly oxidized side chain that terminated in a *tert*-butyl group. Bastimolide B was not as active as **423**, suggesting that the lactone ring size affects the potency [263, 264]. The Thailand marine sponge *Pachastrissa nux* exhibited antiplasmodial activity against K1 *P. falciparum* (IC_50_ = 0.7 µg/mL). The 25-membered ring trisoxazole macrolides kabiramides B (**424**), D (**425**), and J–L (**426**–**428**) (Fig. 56) were subsequently isolated from the extract and were shown to inhibit K1 parasites. However, the compounds were also cytotoxic against cancerous MCF-7 cells and normal human fibroblasts [265, 266]. Likewise, the crude extract of terrestrial *Streptomyces* sp. BCC71188 afforded the macrolides monoglycosylelaiolide (**429**), azalomycin B (**430**), and 11,11′-*O*-dimethylelaiophylin (**431**) as active metabolite against the K1 strain, but these compounds were also cytotoxic against cancerous and Vero cells [254]. *Streptomyces* sp. BCC72023, isolated from rice stems, produced the macrolide efomycin G (**432**) and 29-*O*-methylabierixin (**433**), with activity against K1 *P. falciparum* [267].

### Resorcylic acid lactones

**Fig. 57:**
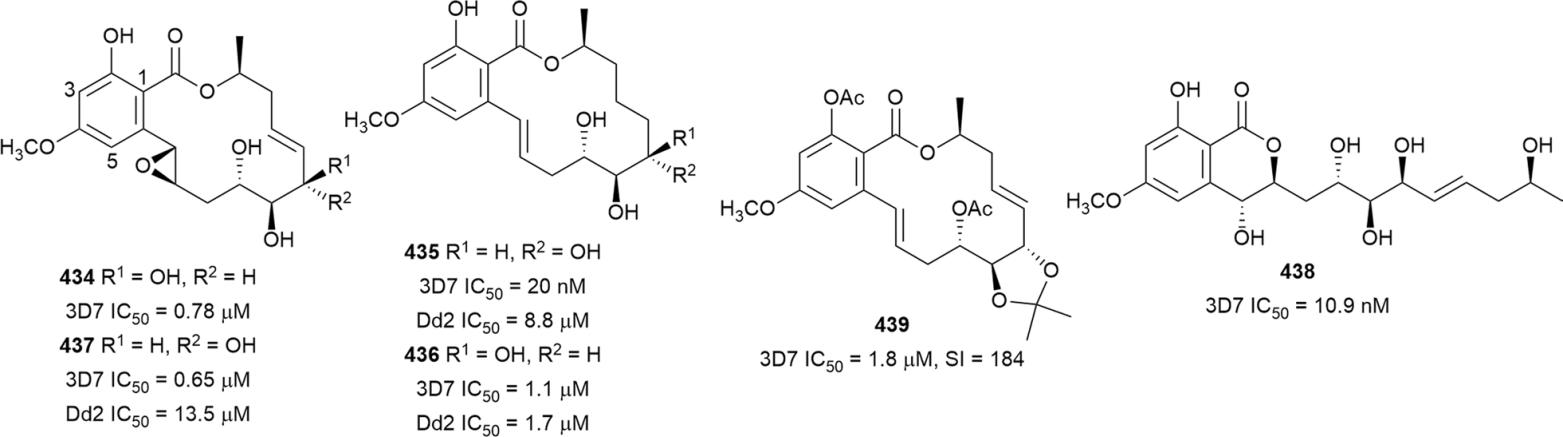
Structures of resorcyclic acid lactones

The mycelial culture of the filamentous fungus, *Paecilomyces* sp. SC0924 produced the new 14-membered ring β-resorcylic acid lactones (RALs, Fig. 57) paecilomycins A (**434**), E (**435**), F (**436**), together with aigilomycin B (**437**) and aigialomycin F (**438**), all with potent antiplasmodial activity. Paecilomycin E (**435**) and aigialomycin F (**438**) showed sub-micromolar inhibition of 3D7 parasites, with all the compounds more potent against the 3D7 than the Dd2 strain, suggesting resistance by the Dd2 strain. The compounds were not cytotoxic against Vero cells at 50 µM [268]. Cochliomycins A–F are structural analogues of the paecilomycins and were initially isolated from the culture broth of *Cochliobolus lunatus* in trace quantities [269, 270]. Two cochliomycin analogues were subsequently isolated in larger amounts from an optimized fermentation broth, and the natural cochliomycins and some derivatives were obtained by semi-synthesis. In contrast to the paecilomycins, the cochliomycins generally showed poor antiplasmodial activity. However, semi-synthetic acetonide derivative **439** inhibited *P. falciparum* (SI = 184) selectively, whereas the hydroxylated parent was inactive. Also, cochliomycin C, the chlorinated derivative of paecilomycin F (**436**), was inactive. SAR deductions from the RAL structures indicate that the presence of acetyl and acetonide groups on the lactone ring hydroxy groups improve activity, with the acetonide derivative having a superior activity. A chlorine atom on the aromatic C-5 decreases activity, while the C-2 phenolic group is important for selective activity. The presence of an enone moiety in the lactone macrocycle contributes to cytotoxicity, whereas the configuration of the 1,2,3-triol or 1,2-diol stereocentres had a negligible effect on the activity [271].

### Cyclodepsipeptides and other peptides

**Fig. 58:**
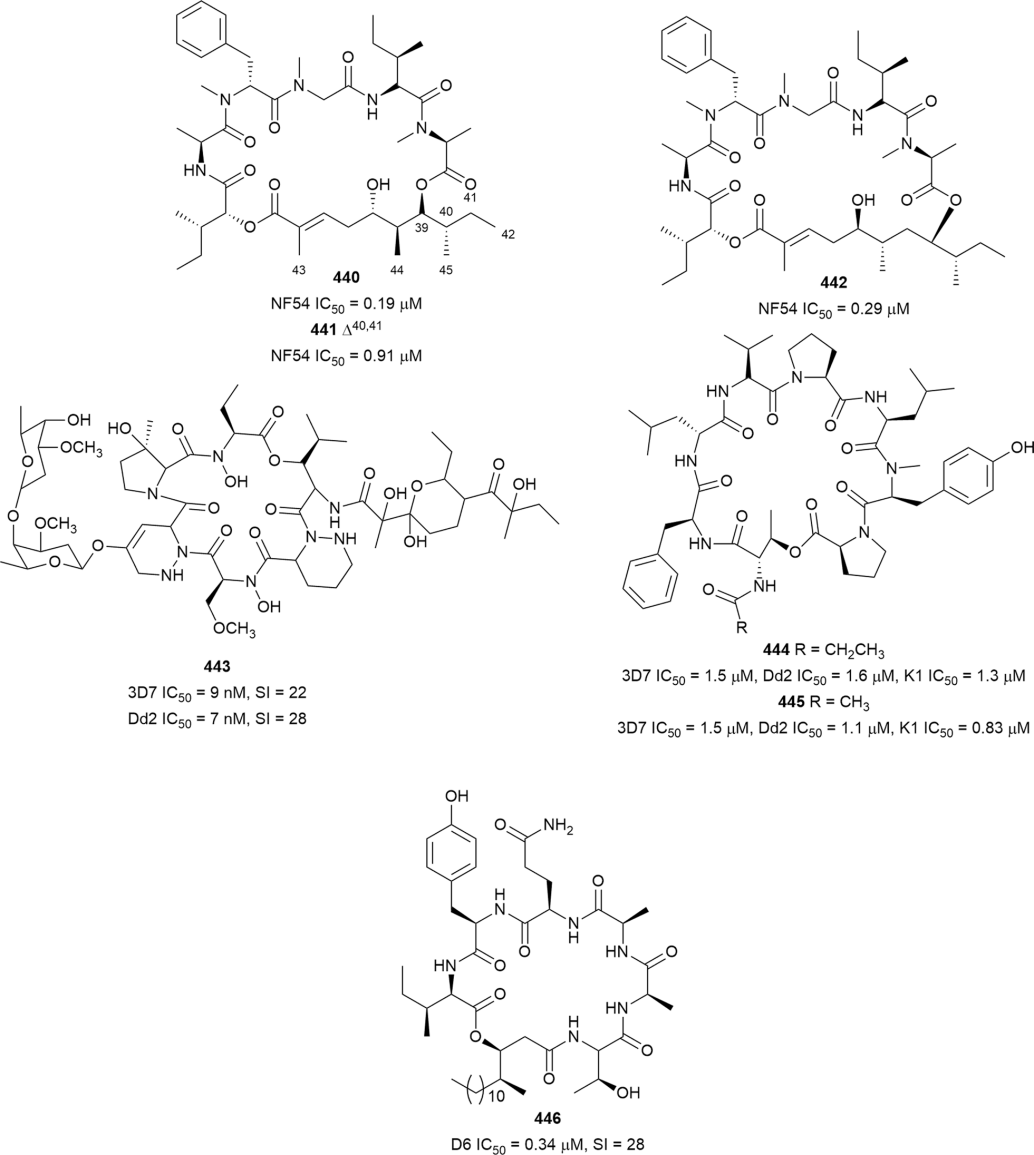
Structures of cyclodepsipeptides

The cyclodepsipeptides lagunamides A–C (**440**–**442**) (Fig. 58) were obtained from the marine cyanobacterium, *Lyngbya majuscula.* The planar lagunamide macrocyclic scaffold consists of peptide and polyketide substructures, and the main differences are in the polyketide part. Lagunamides A and B are 26-membered macrocycles, whereas lagumanide C has an additional methylene carbon in the polyketide structure. The compounds exhibited potent antiplasmodial activity against NF54 *P. falciparum* strain and were also cytotoxic against cancerous cells [272, 273]. The double bond in the side chain of **441** might be responsible for the lower activity. Mollemycin A (**443**), a glyco-hexadepsipeptide with a polyketide residue, was isolated from an Australian *Streptomyces* sp. CMBM0244 and had a potent nanomolar antiplasmodial activity. It was equally active against the 3D7 and Dd2 strains and only slightly cytotoxic against human fibroblast cells (SI > 20), suggesting selectivity against the parasites [274]. Two other antiplasmodial octacyclodepsipeptides, octaminomycins A (**444**) and B (**445**), were isolated from Indonesian soil *Streptomyces* sp. RK85-270. The octaminomycin amino acid sequence was established as cyclo-(Pro-*N*-MeTyr-Leu-Pro-Val-Leu-Phe-Thr). Furthermore, the only structural difference is the presence of propionyl and acetyl chains on the threonine nitrogen in **444** and **445**, respectively. Both compounds were active against 3D7, Dd2, and K1 strains, and were not cytotoxic at 30 µM [275]. A new cyclodepsipeptide incorporating a 3-hydroxy-4-methylpentadecanoic acid moiety, fusaripeptide A (**446**), was obtained from *Mentha longifolia* (Lamiaceae) root endophytic fungus, *Fusarium* sp. from Saudi Arabia. The amino acid sequence was established as cyclo-(Ala-Ala-Thr-Ile-Tyr-Glu). Compound **446** exhibited antiplasmodial activity against D6 *P. falciparum* and was moderately cytotoxic against cancerous L5178Y and PC12 cells [276].

**Fig. 59:**
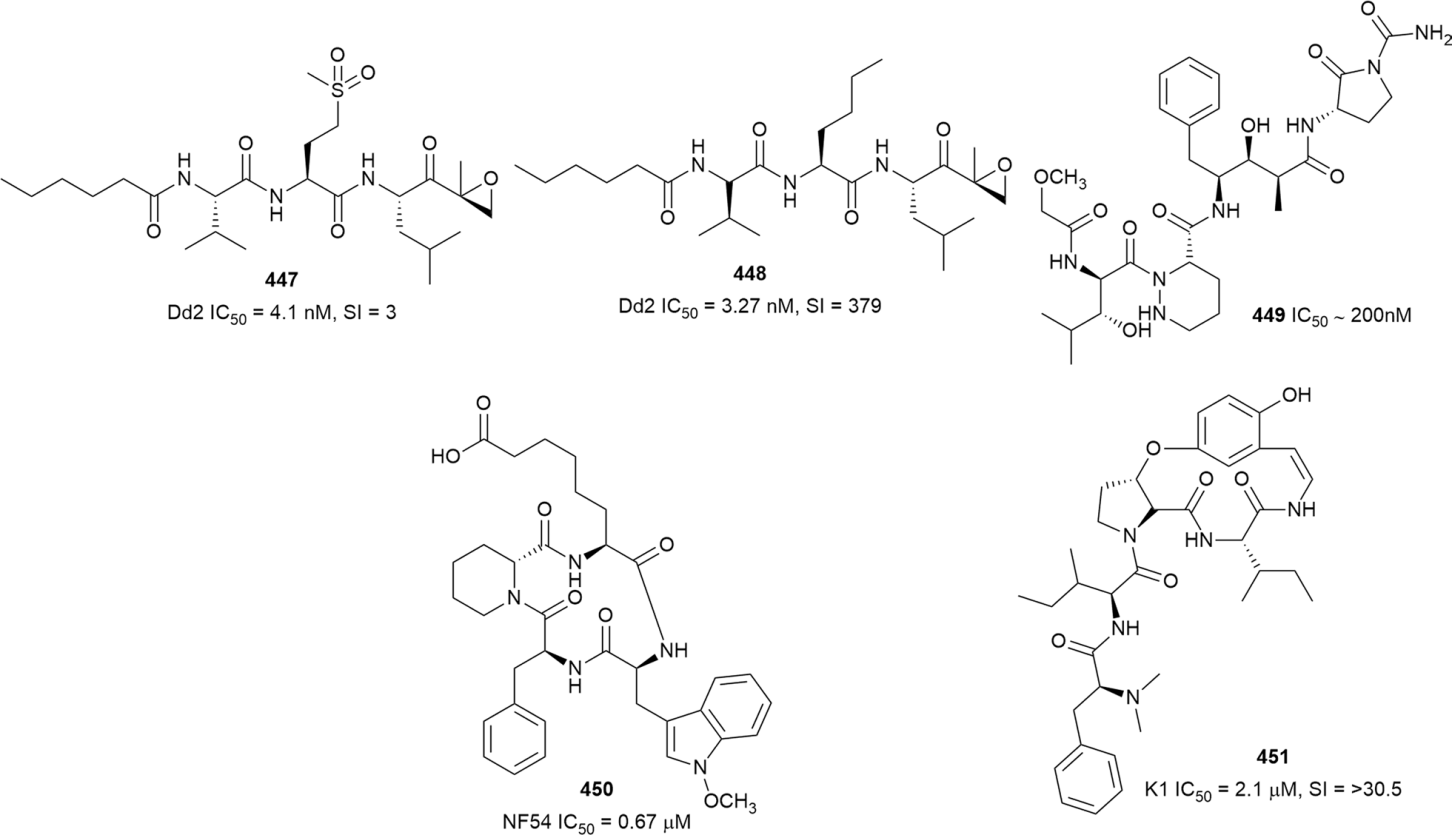
Structures of other peptides

Carmaphycin B (**447**) (Fig. 59), with an amino acid sequence of l-Val-l-Met sulfone-l-Leu, was isolated from the cyanobacterium, *Symploca* sp. The tripeptide with hexanoyl and an α,β-epoxyketone groups on the *N* and carboxyl ends, respectively, had potent nM in vitro and in vivo antiplasmodial activity, but it was also cytotoxic against HepG2 cells. The synthetically modified analogue **448** with a d-Val-l-Nle-l-Leu amino acid sequence showed improved antiplasmodial activity and selectivity (SI = 379). The peptides act in synergy with artemisinin and kill the parasite by targeting the β5 subunit of *Plasmodium* proteasome [277]. A marine actinobacteria from Papua New Guinea, *Streptomyces bangulaensis*, produced the antiplasmodial tetrapeptide, actinoramide A (**449**). The compound displayed sub-micromolar antiplasmodial activity against *P. falciparum* Dd2, HB3, 7G8, GB4, and cp250 strains, and no cytotoxicity. The 25-epimer of **449**, 25-*epi*-actinoramide A was about 20-fold less active, suggesting an influence of the configuration on activity. Actinoramide F, which is structurally similar to **449** but has a 5-amino-5,6-dihydrouracil terminus instead of the cyclic 2-amino-4-ureidobutanoic acid of **449,** was inactive. This suggests that the terminal substructure of the actinoramides is crucial for antiplasmodial activity [278]. A new antiplasmodial cyclic tetrapeptide, apicidin F (**450**), was isolated from the rice fungus pathogen, *Fusarium fujikuroi*, and the amino acid composition was established to be l-tryptophan, d-pipecolic acid, l-phenylalanine, and l-2-aminooctanedioic acid [279].

Cyclopeptide alkaloids are 13, 14, or 15-membered ring polyamides with a styrylamine unit, a β-hydroxy amino acid and other common amino acid forming the macrocycle. The macrocyclic polyamide has an attached side chain, which could be basic or neutral. Spinanine B (**451**) (Fig. 59), a cyclopeptide alkaloid from the stem bark of *Ziziphus spina*-*christi* (Rhamnaceae), inhibited the K1 strain of *P. falciparum* without cytotoxicity to MRC-5 cells at 64 µM [280]. Evaluation of the antiplasmodial activity of 19 cyclopeptide alkaloids facilitated some SAR conclusions. Preliminary SAR studies indicated that the 13-membered ring cyclopeptide alkaloids were generally more active than the 14 and 15 membered analogues. Also, a methoxy group on the styrylamine moiety was more favourable for antiplasmodial activity than a hydroxy group [281, 282].

## Cyclic phosphotriesters

**Fig. 60:**
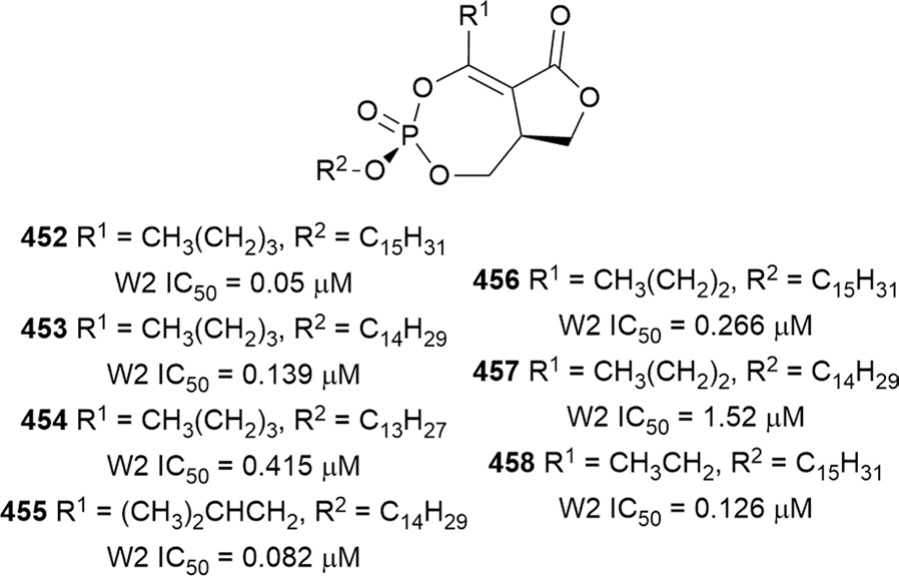
Structures of cyclic phosphotriesters

A marine actinobacterium, *Salinospora* sp., produced a new class of antiplasmodial scaffold based on a bicyclic phosphotriester core, substituted with alkyl chains. Among the new compounds, salinipostins A-D, F-G, and I (**452**–**458**) (Fig. 60) potently inhibited W2 *P. falciparum* without cytotoxicity. Preliminary SAR findings indicated that an increase in alkyl chain length attached to the phosphoester oxygen (R^2^) and vinyl carbon (R^1^) led to increased activity while branching of the R^1^ alkyl causes a slight reduction in activity. The most active **452** had sub-micromolar IC_50_ values and stage-specific activity on early stage parasite ring forms, but parasite trophozoites were less susceptible. The compound did not affect parasite schizonts, indicating that it acts by disrupting the processes required for the establishment or growth of intracellular parasites. Salinispostin A (**452**) did not inhibit haemozoin formation but appears to cause cellular disorganization and disintegration of internal structures. Moreover, experiments for resistance selection under three different conditions failed to identify mutant resistant strains, suggesting that the compound may be less susceptible to resistance development. These compounds represent a new class of antiplasmodial agents, and further biological studies on them are warranted [283].

## Mechanism of action of antiplasmodial natural products: *Plasmodium* cellular targets identified for natural products

Understanding the mechanism of action of bioactive molecules facilitates the development of leads into improved therapeutic compounds and aids in the understanding of resistance evolution. Natural products have proven to be a prolific source of drug leads, but limited knowledge about the mechanism of action often impedes further development [284]. In order to understand the mechanism of action of anti-malarial natural products, it is necessary to identify the molecular targets in the parasite. The anti-malarial drugs in current use are based on pharmacophores acting on a small number of targets as their mechanism of action. The use of these compounds as monotherapy resulted in the emergence of multidrug-resistant parasites [285, 286]. Many of the candidates currently in the anti-malarial drug pipeline are based on chemical modifications to overcome the handicaps of the traditional anti-malarials [287]. Eventual development of resistance to these new therapies when they become clinically useful is inevitable. Therefore, it is imperative to discover new therapeutics with novel targets and mechanisms of action. The increased understanding of the *Plasmodium* parasite biology following the sequencing of its genome has led to the identification of novel potential drug targets that are thought to be essential for parasite survival [288].

Crucial enzymes and macromolecules in the parasite fatty acid, haemoglobin, protein, Ca^2+^ metabolism, glyoxalase detoxification system, and protein folding pathways have been inhibited by natural products. Reports on natural products as inhibitors of potential and proven antiplasmodial targets together with the mechanism of action are summarized in Table 1. Natural products have also modulated their parasite killing effect by disrupting the haem detoxification systems as well as induction of oxidative stress and lipid peroxidation in the parasite (Table 1). Some natural products have also been reported to mediate antiplasmodial activity by inducing morphological and ultrastructural changes that are detrimental to parasite viability [230, 289].

**Table 1:** Mechanism of action of antiplasmodial natural products

Pathway	Mechanism of action	Target	Compound	Compound class	References
Fatty acid metabolism	Inhibitionof type II fatty acid synthase (FAS II) enzymes	*Plasmodium falciparum* enoyl-ACP reductase (*Pf*FabI)	Oroidin	Bromopyrrole alkaloid	[290]
			Luteolin 7-*O*-β-d-glucopyranoside	Flavonoid glycoside	[291]
			Anthecularin	Sesquiterpene lactone	[292]
			4-Hydroxyanthecotulide	Sesquiterpene lactone	[293]
			4-Acetoxyanthecotulide	Sesquiterpene lactone	[293]
			Mucusisoflavone C	Flavonoid	[294]
			3-*O*-Methylquercetin	Flavonoid	[294]
			Isowighteone	Flavonoid	[294]
			Evernic acid	Phenolic acid	[295]
			Psoromic acid	Phenolic acid	[295]
			Methylenebissantin	Flavonoid	[296]
		*Plasmodium falciparum* β-ketoacyl-ACP reductase (*Pf*FabG)	Anthecularin	Sesquiterpene lactone	[292]
			4-Hydroxyanthecotulide	Sesquiterpene lactone	[293]
			4-Acetoxyanthecotulide	Sesquiterpene lactone	[293]
			Psoromic acid	Phenolic acid	[295]
		*Plasmodium falciparum* β-hydroxyacyl-ACP dehydratase (*Pf* FabZ)	Evernic acid	Phenolic acid	[295]
			Vulpic acid	Phenolic acid	[295]
			Psoromic acid	Phenolic acid	[295]
			Catechin gallate	Catechin	[297]
			Bromopyrrolohomoarginin	Bromopyrrole alkaloid	[93]
Detoxification of haem	Inhibition of β-haematin formation	Haem crystallization	Bromophycolide A	Macrocyclic meroditerpene	[262]
			Bergenin	Phenolic glycoside	[298]
			Fraxetin	Coumarin	[299]
			1,3,6-Trihydroxy-2-(3-methyl but-dienyl)-7-methoxy-8-(3- methyl but-2-enyl)xanthen-9-one	Xanthone	[300]
			2-(6-*O*-Benzoyl-β-d-glucopyranosyloxy)-7-(1α, 2α, 6α-trihydroxy-3-oxocyclohex-4-enoyl)-5-hydroxybenzyl alcohol	Phenolic glycoside	[301]
			Dimethylisoborreverine	Indole alkaloid	[46]
			Nitidine	Benzophenanthridine alkaloid	[62]
	Inhibition of β-haematin formation, inhibition of H_2_O_2_ and glutathione mediated hemin degradation		Axisonitrile-3	Sesquiterpene isonitrile	[154]
			Diisocyanoadociane	Diterpene isonitrile	[154]
	Inhibition of β-haematin formation, inhibition of H_2_O_2_ mediated haemin degradation		Catechin-[5,6-e]-4β-(3,4-dihydroxyphenyl) dihydro-2(3H)-pyranone	Phenylpropanoid catechin	[203]
			Mururin A	Phenylpropanoid catechin	[203]
		*Plasmodium falciparum* glutathione transferase (*Pf*GST)	JB42C	Sesquiterpene lactone	[302]
			Tral-1	Coumarin catechin	[302]
Oxidative stress	Production of ROS and lipid peroxidation product	Trafficking, transmembrane and vesicular transport parasite proteins	Plakortin	Polyketide endoperoxide	[303]
Haemoglobin degradation	Inhibition of food vacuole falcipains	Falcipain 2	2,3,6-Trihydroxy benzoic acid	Phenolic acid	[304]
			2,3,6-Trihydroxy methyl benzoate	Phenolic ester	[304]
			Symplostatin 4	Depsipeptide	[284]
		Falcipain 2′ and 3	Symplostatin 4	Depsipeptide	[284]
Glyoxalase detoxification system	Inhibition of *Plasmodium falciparum* glyoxalase I (PfGLOI)	(*Pf*GLOI)	Puberulic acid	Tropone	[305]
			Hinokitiol	Tropone	[305]
			Tropolone	Tropone	[305]
Protein folding	Inhibition of *Plasmodium falciparum* Hsp70-1 (PfHsp70-1) chaperone function	(*Pf*Hsp70-1)	Malonganenone A	Purine alkaloid	[306]
			Malonganenone B	Purine alkaloid	[306]
			Malonganenone C	Alkaloid	[306]
			Lapachol	Naphthoquinone	[306]
	Inhibition of chaperone and ATPase functions	PfHsp70-1, PfHsp70-z	Epigallocatechin 3-gallate	Catechin	[205]
Protein degradation	inhibition of Pf20S proteasome	β5 subunit	Carmaphycin B	Tripeptide	[277]
Ca^2+^ metabolism	Inhibition of SERCA-type Ca^2+^-ATPase	PfATP6	Artemisinins	Sesquiterpene endoperoxide	[307]
Protein biosynthesis	Inhibition of cytoplasmic lysyl-tRNA synthetase	*Pf* lysyl-tRNA synthetase	Cladosporin	Isocoumarin	[308]

## Natural products with transmission-blocking potentials

Malaria chemotherapy has focused mainly on controlling the disease. Thus, most anti-malarial drugs act on asexual blood stage parasites that are responsible for the clinical manifestations of the disease. However, there has been a shift in focus towards malaria elimination strategies. In this regard, compounds with activity against asymptomatic gametocytes and liver stage parasites, including hypnozoites (prophylaxis), are crucial to the eradication agenda. As yet, artesunate, artemether, methylene blue, and primaquine are the known gametocidal agents, with primaquine being the only approved prophylactic and gametocytocidal drug [309]. Some natural products have shown transmission blocking potential by exhibiting activity against one or more of these parasite life cycle stages. These natural products are summarized in Table 2. Similarly, extracts from *Zanthoxylum heitzii*, *Vernonia amygdalina*, *Artemisia afra*, *Trichilia emetica*, *Turraea floribunda*, and *Leonotis leonurus* have shown gametocidal activity [65, 310, 311]. The standardized commercial preparation NeemAzal^®^, an azadirachtin-enriched neem extract, has also demonstrated potent transmission-blocking activity ex vivo and in vivo [312, 313]. The targeting of gametocytes is indeed of great importance in the fight against malaria. The limited information available in the literature on drugs with this activity may be a reflection of the difficulty of assaying gametocidal activity.

**Table 2:** Natural products with transmission-blocking potential

Compound	Parasite	Active on	Activity	IC_50_	References
Dihydronitidine	*Plasmodium berghei*	Early mosquito stage	Inhibition of ookinete formation	1.7 µM	[65]
Heitziquinone	*P. berghei*	Early mosquito stage	Inhibition of ookinete formation	17 µM	[65]
Mallotojaponin C	*P. falciparum* NF54	Stage V gametocytes	Gametocytocidal	3.6 µM	[232]
Carmaphycin B	*P. falciparum* NF54	Stage V gametocytes	Gametocytocidal	160 nM	[277]
Carmaphycin B	*P. berghei*	Liver stage		61.6 nM	
Parthenine	*P. falciparum* NF54	Stage V gametocytes	Gametocytocidal. Inhibition of microgamete exflagellation. Prevent oocysts development in mosquito at 3.84 µM		[314]
		Early mosquito stage	Inhibition of ookinete formation	100% inhibition at 191 µM	[314]
Parthenolide	*P. falciparum* NF54	stage V gametocytes	Gametocytocidal. Inhibition of microgamete exflagellation. prevent oocysts development in mosquito at 4 µM		[314]
Deacetylnimbin	*P. berghei*	Early mosquito stage	Inhibition of ookinete formation	100% inhibition at 100 µM	[315]
Azadirachtin	*P. falciparum* NF54	Stage V gametocytes	100% inhibition of microgamete exflagellation		[316]
	*P. berghei*	Stage V gametocytes	Gametocytocidal. Inhibition of microgamete exflagellation	3.25 µM	[316]
Vernodalol	*P. berghei*	Early mosquito stage	Inhibition of early sporogenic stages development	18.7 µM	[310]
Vernolide	*P. berghei*	Early mosquito stage	9–33% inhibition of early sporogenic stages development at 50 µM		[310]
Usnic acid	*P. berghei*	Liver stage		2.3 µM	[295]
Vulpic acid	*P. berghei*	Liver stage		10.2 µM	[295]
Psoromic acid	*P. berghei*	Liver stage		31.6 µM	[295]
Evernic acid	*P. berghei*	Liver stage		77.3 µM	[295]
Balsaminol F	*P. berghei*	Liver stage	Inhibition of intracellular development	> 95% inhibition at 15 µM	[163]
BalsaminosideB	*P. berghei*	Liver stage	Inhibition of intracellular development	> 95% inhibition at 15 µM	[163]
Triacetylbalsaminol F	*P. berghei*	Liver stage	Inhibition of intracellular development	> 95% inhibition at 15 µM	[163]
6-Deoxy-8-*O*-methylrabelomycin	*P. berghei*	Liver stage		18.5 µM	[317]
X-14881 E	*P. berghei*	Liver stage		3.0 µM	[317]
Marilone A	*P. berghei*	Liver stage	Inhibition of liver cell infection	12.1 µM	[318]

## Conclusions

**Fig. 61:**
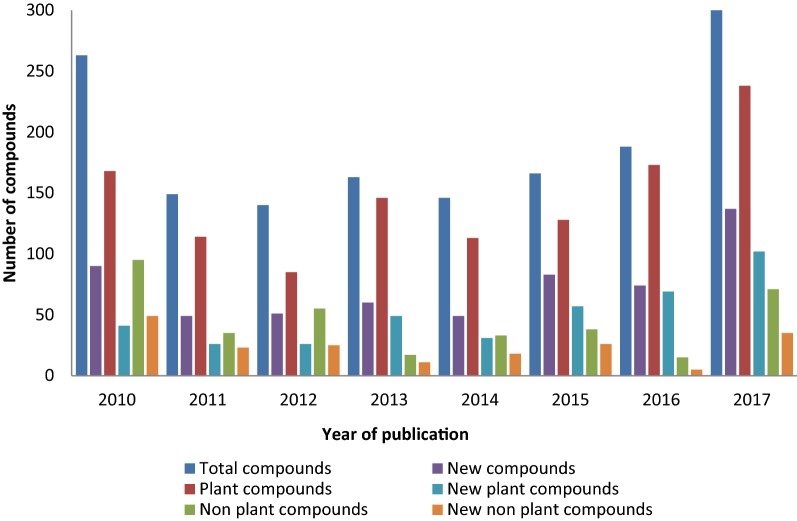
Summary of biological sources from which antiplasmodial natural products were isolated between 2010 and 2017

The present review covered the antiplasmodial natural products reported between 2010 and 2017. A breakdown of the statistics of compounds and the biological source reported per year is given in Fig. 61.

A total of 447 compounds derived from plant, microrganisms, and marine organisms were found to have IC_50_ ≤ 3.0 µM against at least one strain of asexual blood-stage *P. falciparum*. Some of these compounds showed potent selective activity against parasites. However, others were equally cytotoxic against cancerous and/or noncancerous cells. Notwithstanding the cytotoxicity, we have considered these compounds to be promising anti-malarial hits of which the cytotoxicity could be mitigated by medicinal chemistry approaches, as was demonstrated, for example, with ferruginol (**230**) and carmaphycin B (**447**) [149, 277]. Also, some of these compounds could serve as templates for designing novel antiplasmodial pharmacophores using the strategy of diversity-oriented synthesis. More than half of the compounds belong to three major chemical classes comprising alkaloids (31.9%), terpenoids (30.8%), and polyphenols (17.4%). This is consistent with the phytochemical distribution of antiplasmodial compounds from earlier reviews [12, 13]. This review also discussed compounds with potent selective antiplasmodial activities that belong to classes from which antiplasmodial activity has not been previously reported. Such novel antiplasmodial scaffolds, e.g. the tropolones and cyclic phosphotriesters, are important in the battle against malaria because these compounds might possess a novel mechanism of action and expand the therapeutic arsenal against the disease.

Many of the plants were investigated based on the ethnobotanical history of use against malaria, while the non-plant materials were mostly investigated because of their chemical profiles. This underscores the need to employ the dual approach of ethnobotanical reputation and chemical profiling in the search for anti-malarial natural products. Although fewer marine and microorganisms have been screened for antiplasmodial compounds compared to plants, the diverse nature of metabolites produced by these alternative sources presents a compelling case for intensive exploration. Only a small number of in vivo studies to validate the in vitro efficacy of these compounds have been conducted. This is not surprising considering that natural products are usually isolated in small amounts, which is barely sufficient for in vitro testing. Nevertheless, the need for in vivo confirmation of observed in vitro potency could not be overemphasized. Therefore, efficient total synthesis of the most promising compounds identified in this review should be prioritized.

It is now accepted that the *Plasmodium* parasite transmission from an infected host to uninfected mosquitoes should be blocked to curtail the spread of malaria. Compounds with activity against sexual and liver stage parasites are therefore crucial to the malaria eradication agenda. Sadly, only a few chemical compounds have a proven ability to kill liver and sexual stage parasites. In spite of the fruitful relationship between malaria chemotherapy and natural products, only a few natural products have been evaluated for activity against the parasite gametocytes and liver forms. As this review has shown, the few investigated natural products have shown promise. Therefore, the exploration of natural products in this regard cannot be overemphasized. The compounds covered in this review will be a good starting point, since natural products, unlike the synthetic counterparts, might have multi-stage activity. Priority should be given to compounds of which the mode of action does not involve the inhibition of haemozoin formation since stage V gametes, which are the transmissible form of the parasite, do not digest haemoglobin. Standardized assay methods for evaluating late stage gametocidal and liver stage activities have been developed that will aid in the high-throughput screening of natural products [319, 320].

## Supplementary information

**Additional file 1.** Antiplasmodial activity reported (2010–2017) for all natural products, irrespective of level of activity (no cutoff value for activity).

## Data Availability

Not applicable.

## Abbreviations

ACT: artemisinin-based combination therapy
ATPase: adenosine triphosphatase
CGX: Garcinia xanthones
DDT: dichlorodiphenyltrichloroethane
DGAT: diacylglycerol *O*-acyltransferase
DNA: deoxyribonucleic acid
EGCG: epigallocatechin-3-gallate
ELISA: enzyme-linked immunosorbent assays
Glu: glutamic acid
HRP2: histidine-rich protein 2
Ile: isoleucine
LC-MS: liquid chromatography-mass spectrometry
Leu: leucine
Met: methionine
MeTyr: methyl tyrosine
Nle: norleucine
µM: micromolar
nM: nanomolar
NMR: nuclear magnetic resonance spectroscopy
PfDGAT: *Plasmodium falciparum* diacylglycerol *O*-acyltransferase
PfHsp: *Plasmodium falciparum* heat shock protein
Phe: phenylalanine
pLDH: Plasmodium lactate dehydrogenase protein
PPAP: polyprenylated acylphloroglucinol
Pro: proline
ROS: recactive oxygen species
SAR: structure–activity relationship
SI: selectivity index
Thr: threonine
USA: United States of America
Val: valine
WHO: World Health Organization

## References

[1] WHO (2018). World Malaria Report 2017.

[2] Kiszewski A, Mellinger A, Spielman A, Malaney P, Sachs SE, Sachs J (2004). A global index representing the stability of malaria transmission. Am J Trop Med Hyg.

[3] Snow RW, Guerra CA, Noor AM, Myint HY, Hay SI (2005). The global distribution of clinical episodes of *Plasmodium falciparum* malaria. Nature.

[4] Hay SI, Guerra CA, Tatem AJ, Noor AM, Snow RW (2004). The global distribution and population at risk of malaria: past, present, and future. Lancet Infect Dis..

[5] Russell PF (1956). World-wide malaria distribution, prevalence, and control. Am J Trop Med Hyg.

[6] Snow RW, Sartorius B, Kyalo D, Maina J, Amratia P, Mundia CW (2017). The prevalence of *Plasmodium falciparum* in sub-Saharan Africa since 1900. Nature.

[7] Howard J. Tick- and mosquito-borne diseases more than triple, since 2004, in the US. https://edition.cnn.com/2018/05/01/health/ticks-mosquito-borne-diseases-cdc-study/index.html. Accessed 30 July 2018.

[8] Lu F, Culleton R, Zhang M, Ramaprasad A, von Seidlein L, Zhou H (2017). Emergence of indigenous artemisinin-resistant *Plasmodium falciparum* in Africa. N Engl J Med.

[9] Talisuna AO, Bloland P, d’Alessandro U (2004). History, dynamics, and public health importance of malaria parasite resistance. Clin Microbiol Rev.

[10] Wells TN, Van Huijsduijnen RH, Van Voorhis WC (2015). Malaria medicines: a glass half full?. Nat Rev Drug Discovery.

[11] Schwikkard S, van Heerden FR (2002). Antimalarial activity of plant metabolites. Nat Prod Rep.

[12] Bero J, Frédérich M, Quetin-Leclercq J (2009). Antimalarial compounds isolated from plants used in traditional medicine. J Pharm Pharmacol.

[13] Bero J, Quetin-Leclercq J (2011). Natural products published in 2009 from plants traditionally used to treat malaria. Planta Med.

[14] Nogueira CR, Lopes LM (2011). Antiplasmodial natural products. Molecules.

[15] Wright CW (2010). Recent developments in research on terrestrial plants used for the treatment of malaria. Nat Prod Rep.

[16] Laurent D, Pietra F (2006). Antiplasmodial marine natural products in the perspective of current chemotherapy and prevention of malaria. A review. Mar Biotechnol..

[17] Fattorusso E, Taglialatela-Scafati O (2009). Marine antimalarials. Mar Drugs..

[18] Gertsch J (2009). How scientific is the science in ethnopharmacology? Historical perspectives and epistemological problems. J Ethnopharmacol.

[19] Krettli AU, Adebayo JO, Krettli LG (2009). Testing of natural products and synthetic molecules aiming at new antimalarials. Curr Drug Targets.

[20] Wein S, Maynadier M, Van Ba CT, Cerdan R, Peyrottes S, Fraisse L (2010). Reliability of antimalarial sensitivity tests depends on drug mechanisms of action. J Clin Microbiol.

[21] Mokgethi-Morule T, N’Da DD (2016). Cell based assays for anti-*Plasmodium* activity evaluation. Eur J Pharm Sci.

[22] Chianese G, Persico M, Yang F, Lin H-W, Guo Y-W, Basilico N (2014). Endoperoxide polyketides from a Chinese *Plakortis simplex:* further evidence of the impact of stereochemistry on antimalarial activity of simple 1,2-dioxanes. Bioorg Med Chem.

[23] Fattorusso C, Persico M, Calcinai B, Cerrano C, Parapini S, Taramelli D (2010). Manadoperoxides A–D from the Indonesian sponge *Plakortis* cfr. *simplex.* Further insights on the structure-activity relationships of simple 1,2-dioxane antimalarials. J Nat Prod.

[24] Fattorusso E, Parapini S, Campagnuolo C, Basilico N, Taglialatela-Scafati O, Taramelli D (2002). Activity against *Plasmodium falciparum* of cycloperoxide compounds obtained from the sponge *Plakortis simplex*. J Antimicrob Chemother.

[25] Taglialatela-Scafati O, Fattorusso E, Romano A, Scala F, Barone V, Cimino P (2010). Insight into the mechanism of action of plakortins, simple 1,2-dioxane antimalarials. Org Biomol Chem.

[26] Jiménez-Romero C, Ortiz I, Vicente J, Vera B, Rodríguez AD, Nam S (2010). Bioactive cycloperoxides isolated from the Puerto Rican sponge *Plakortis halichondrioides*. J Nat Prod.

[27] Yang F, Wang R-P, Xu B, Yu H-B, Ma G-Y, Wang G-F (2016). New antimalarial norterpene cyclic peroxides from Xisha Islands sponge *Diacarnus megaspinorhabdosa*. Bioorg Med Chem Lett.

[28] Yang F, Zou Y, Wang R-P, Hamann MT, Zhang H-J, Jiao W-H (2014). Relative and absolute stereochemistry of diacarperoxides: antimalarial norditerpene endoperoxides from marine sponge *Diacarnus megaspinorhabdosa*. Mar Drugs..

[29] Bringmann G, Tasler S (2001). Oxidative aryl coupling reactions: a biomimetic approach to configurationally unstable or axially chiral biaryl natural products and related bioactive compounds. Tetrahedron.

[30] Li J, Seupel R, Feineis D, Mudogo V, Kaiser M, Brun R (2017). Dioncophyllines C2, D2, and F and related naphthylisoquinoline alkaloids from the Congolese liana *Ancistrocladus ileboensis* with potent activities against *Plasmodium falciparum* and against multiple myeloma and leukemia cell lines. J Nat Prod.

[31] Hallock YF, Manfredi KP, Dai J-R, Cardellina JH, Gulakowski RJ, McMahon JB (1997). Michellamines D–F, new HIV-inhibitory dimeric naphthylisoquinoline alkaloids, and korupensamine E, a new antimalarial monomer, from *Ancistrocladus korupensis*. J Nat Prod.

[32] Bringmann G, Zhang G, Ölschläger T, Stich A, Wu J, Chatterjee M (2013). Highly selective antiplasmodial naphthylisoquinoline alkaloids from *Ancistrocladus tectorius*. Phytochemistry.

[33] Bringmann G, Seupel R, Feineis D, Xu M, Zhang G, Kaiser M (2017). Antileukemic ancistrobenomine B and related 5,1′-coupled naphthylisoquinoline alkaloids from the Chinese liana *Ancistrocladus tectorius*. Fitoterapia.

[34] Xu M, Bruhn T, Hertlein B, Brun R, Stich A, Wu J (2010). Shuangancistrotectorines A-E, dimeric naphthylisoquinoline alkaloids with three chiral biaryl axes from the Chinese plant Ancistrocladus tectorius. Chem Eur J.

[35] Bringmann G, Zhang G, Büttner T, Bauckmann G, Kupfer T, Braunschweig H (2013). Jozimine A2: the first dimeric Dioncophyllaceae-type naphthylisoquinoline alkaloid, with three chiral axes and high antiplasmodial activity. Chem Eur J.

[36] Bringmann G, Lombe BK, Steinert C, Ioset KN, Brun R, Turini F (2013). Mbandakamines A and B, unsymmetrically coupled dimeric naphthylisoquinoline alkaloids, from a Congolese *Ancistrocladus* species. Org Lett.

[37] Lombe BK, Bruhn T, Feineis D, Mudogo V, Brun R, Bringmann G (2017). Antiprotozoal spirombandakamines A1 and A2, fused naphthylisoquinoline dimers from a Congolese *Ancistrocladus* plant. Org Lett.

[38] Li J, Seupel R, Bruhn T, Feineis D, Kaiser M, Brun R (2017). Jozilebomines A and B, naphthylisoquinoline dimers from the Congolese liana *Ancistrocladus ileboensis*, with antiausterity activities against the PANC-1 human pancreatic cancer cell Line. J Nat Prod.

[39] Tshitenge DT, Feineis D, Mudogo V, Kaiser M, Brun R, Bringmann G (2017). Antiplasmodial ealapasamines A-C, ‘mixed’naphthylisoquinoline dimers from the Central African liana *Ancistrocladus ealaensis*. Sci Rep..

[40] Bringmann G, Gulder T, Hertlein B, Hemberger Y, Meyer F (2010). Total synthesis of the N, C-coupled naphthylisoquinoline alkaloids ancistrocladinium A and B and related analogues. J Am Chem Soc.

[41] Deguchi J, Hirahara T, Hirasawa Y, Ekasari W, Widyawaruyanti A, Shirota O (2012). New tricyclic alkaloids, cassiarins G, H, J, and K from leaves of *Cassia siamea*. Chem Pharm Bull.

[42] Morita H, Oshimi S, Hirasawa Y, Koyama K, Honda T, Ekasari W (2007). Cassiarins A and B, novel antiplasmodial alkaloids from *Cassia siamea*. Org Lett.

[43] Zahari A, Cheah FK, Mohamad J, Sulaiman SN, Litaudon M, Leong KH (2014). Antiplasmodial and antioxidant isoquinoline alkaloids from *Dehaasia longipedicellata*. Planta Med.

[44] Carraz M, Jossang A, Franetich JF, Siau A, Ciceron L, Hannoun L (2006). A plant-derived morphinan as a novel lead compound active against malaria liver stages. Plos Medicine..

[45] Fernandez LS, Jobling MF, Andrews KT, Avery VM (2008). Antimalarial activity of natural product extracts from Papua New Guinean and Australian plants against *Plasmodium falciparum*. Phytother Res..

[46] Fernandez LS, Sykes ML, Andrews KT, Avery VM (2010). Antiparasitic activity of alkaloids from plant species of Papua New Guinea and Australia. Int J Antimicrob Agents.

[47] Likhitwitayawuid K, Angerhofer CK, Chai H, Pezzuto JM, Cordell GA, Ruangrungsi N (1993). Cytotoxic and antimalarial alkaloids from the tubers of *Stephania pierrei*. J Nat Prod.

[48] Le PM, Srivastava V, Nguyen TT, Pradines B, Madamet M, Mosnier J (2017). Stephanine from *Stephania venosa* (Blume) Spreng showed effective antiplasmodial and anticancer activities, the latter by inducing apoptosis through the reverse of mitotic exit. Phytother Res..

[49] Ropivia J, Derbré S, Rouger C, Pagniez F, Le Pape P, Richomme P (2010). Isoquinolines from the roots of *Thalictrum flavum* L. and their evaluation as antiparasitic compounds. Molecules.

[50] Wangchuk P, Bremner JB, Rattanajak R, Kamchonwongpaisan S (2010). Antiplasmodial agents from the Bhutanese medicinal plant *Corydalis calliantha*. Phytother Res..

[51] Wangchuk P, Keller PA, Pyne SG, Taweechotipatr M, Tonsomboon A, Rattanajak R (2011). Evaluation of an ethnopharmacologically selected Bhutanese medicinal plants for their major classes of phytochemicals and biological activities. J Ethnopharmacol.

[52] Wangchuk P, Keller PA, Pyne SG, Lie W, Willis AC, Rattanajak R (2013). A new protoberberine alkaloid from *Meconopsis simplicifolia* (D. Don) Walpers with potent antimalarial activity against a multidrug resistant *Plasmodium falciparum* strain. J Ethnopharmacol.

[53] Chea A, Bun S-S, Azas N, Gasquet M, Bory S, Ollivier E (2010). Antiplasmodial activity of three bisbenzylisoquinoline alkaloids from the tuber of *Stephania rotunda*. Nat Prod Res.

[54] Chea A, Hout S, Bun S-S, Tabatadze N, Gasquet M, Azas N (2007). Antimalarial activity of alkaloids isolated from *Stephania rotunda*. J Ethnopharmacol.

[55] Baghdikian B, Mahiou-Leddet V, Bory S, Bun S-S, Dumetre A, Mabrouki F (2013). New antiplasmodial alkaloids from *Stephania rotunda*. J Ethnopharmacol.

[56] Desgrouas C, Chapus C, Desplans J, Travaille C, Pascual A, Baghdikian B (2014). *In vitro* antiplasmodial activity of cepharanthine. Malar J..

[57] Desgrouas C, Dormoi J, Chapus C, Ollivier E, Parzy D, Taudon N (2014). *In vitro* and in vivo combination of cepharanthine with anti-malarial drugs. Malar J..

[58] Sun YF, Wink M (2014). Tetrandrine and fangchinoline, bisbenzylisoquinoline alkaloids from *Stephania tetrandra* can reverse multidrug resistance by inhibiting P-glycoprotein activity in multidrug resistant human cancer cells. Phytomedicine.

[59] Ye Z, van Dyke K (2015). Antimalarial activity of various bisbenzylisoquinoline and aporphine-benzylisoquinoline alkaloids and their structure-activity relationships against chloroquine—sensitive and resistant *Plasmodium falciparum* malaria *in vitro*. Malar Contr Elim..

[60] Nasrullah AA, Zahari A, Mohamad J, Awang K (2013). Antiplasmodial alkaloids from the bark of *Cryptocarya nigra* (Lauraceae). Molecules.

[61] Kubo M, Yatsuzuka W, Matsushima S, Harada K, Inoue Y, Miyamoto H (2016). Antimalarial phenanthroindolizine alkaloids from *Ficus septica*. Chem Pharm Bull.

[62] Bouquet J, Rivaud M, Chevalley S, Deharo E, Jullian V, Valentin A (2012). Biological activities of nitidine, a potential anti-malarial lead compound. Malar J..

[63] Muganga R, Angenot L, Tits M, Frédérich M (2014). *In vitro* and in vivo antiplasmodial activity of three Rwandan medicinal plants and identification of their active compounds. Planta Med.

[64] Gakunju D, Mberu E, Dossaji S, Gray A, Waigh R, Waterman P (1995). Potent antimalarial activity of the alkaloid nitidine, isolated from a Kenyan herbal remedy. Antimicrob Agents Chemother.

[65] Goodman CD, Austarheim I, Mollard V, Mikolo B, Malterud KE, McFadden GI (2016). Natural products from *Zanthoxylum heitzii* with potent activity against the malaria parasite. Malar J..

[66] Dolabela MF, Póvoa MM, Brandão GC, Rocha FD, Soares LF, de Paula RC (2015). *Aspidosperma* species as sources of anti-malarials: uleine is the major anti-malarial indole alkaloid from *Aspidosperma parvifolium* (Apocynaceae). Malar J..

[67] de Oliveira AB, Dolabela MF, Póvoa MM, Santos CAM, de Pilla Varotti F (2010). Antimalarial activity of ulein and proof of its action on the *Plasmodium falciparum* digestive vacuole. Malar J..

[68] Chierrito TP, Aguiar AC, de Andrade IM, Ceravolo IP, Gonçalves RA, de Oliveira AJ (2014). Anti-malarial activity of indole alkaloids isolated from *Aspidosperma olivaceum*. Malar J..

[69] Muganza DM, Fruth B, Nzunzu JL, Tuenter E, Foubert K, Cos P (2016). *In vitro* antiprotozoal activity and cytotoxicity of extracts and isolated constituents from *Greenwayodendron suaveolens*. J Ethnopharmacol.

[70] Fernandez LS, Buchanan MS, Carroll AR, Feng YJ, Quinn RJ, Avery VM (2008). Flinderoles A–C: antimalarial bis-indole alkaloids from *Flindersia* species. Org Lett.

[71] Robertson LP, Duffy S, Wang Y, Wang D, Avery VM, Carroll AR (2017). Pimentelamines A-C, indole alkaloids isolated from the leaves of the Australian tree Flindersia pimenteliana. J Nat Prod.

[72] Girardot M, Deregnaucourt C, Deville A, Dubost L, Joyeau R, Allorge L (2012). Indole alkaloids from *Muntafara sessilifolia* with antiplasmodial and cytotoxic activities. Phytochemistry.

[73] Ramanitrahasimbola D, Rasoanaivo P, Ratsimamanga-Urverg S, Federici E, Palazzino G, Galeffi C (2001). Biological activities of the plant-derived bisindole voacamine with reference to malaria. Phytother Res..

[74] Fox Ramos AE, Alcover C, Evanno L, Maciuk A, Litaudon M, Duplais C (2017). Revisiting previously investigated plants: a molecular networking-based study of *Geissospermum laeve*. J Nat Prod.

[75] Mbeunkui F, Grace MH, Lategan C, Smith PJ, Raskin I, Lila MA (2012). In vitro antiplasmodial activity of indole alkaloids from the stem bark of *Geissospermum vellosii*. J Ethnopharmacol.

[76] Tchinda AT, Ngono AR, Tamze V, Jonville MC, Cao M, Angenot L (2012). Antiplasmodial alkaloids from the stem bark of *Strychnos malacoclados*. Planta Med.

[77] Tchinda AT, Jansen O, Nyemb J-N, Tits M, Dive G, Angenot L (2014). Strychnobaillonine, an unsymmetrical bisindole alkaloid with an unprecedented skeleton from *Strychnos icaja* roots. J Nat Prod.

[78] Frédérich M, Jacquier M-J, Thépenier P, De Mol P, Tits M, Philippe G (2002). Antiplasmodial activity of alkaloids from various *Strychnos* species. J Nat Prod.

[79] Silva L, Montoia A, Amorim R, Melo M, Henrique M, Nunomura SM (2012). Comparative in vitro and in vivo antimalarial activity of the indole alkaloids ellipticine, olivacine, cryptolepine and a synthetic cryptolepine analog. Phytomedicine.

[80] Montoia A, Silva LF, Torres ZE, Costa DS, Henrique MC, Lima ES (2014). Antiplasmodial activity of synthetic ellipticine derivatives and an isolated analog. Bioorg Med Chem Lett.

[81] Rajachan O-A, Kanokmedhakul K, Sanmanoch W, Boonlue S, Hannongbua S, Saparpakorn P (2016). Chevalone C analogues and globoscinic acid derivatives from the fungus *Neosartorya spinosa* KKU-1NK1. Phytochemistry.

[82] Liew LP, Fleming JM, Longeon A, Mouray E, Florent I, Bourguet-Kondracki M-L (2014). Synthesis of 1-indolyl substituted β-carboline natural products and discovery of antimalarial and cytotoxic activities. Tetrahedron.

[83] Pereira MD, da Silva T, Aguiar ACC, Oliva G, Guido RV, Yokoyama-Yasunaka JK (2017). Chemical composition, antiprotozoal and cytotoxic activities of indole alkaloids and benzofuran neolignan of *Aristolochia cordigera*. Planta Med.

[84] Huang H, Yao Y, He Z, Yang T, Ma J, Tian X (2011). Antimalarial β-carboline and indolactam alkaloids from *Marinactinospora thermotolerans*, a deep sea isolate. J Nat Prod.

[85] Chan ST, Pearce AN, Page MJ, Kaiser M, Copp BR (2011). Antimalarial β-carbolines from the New Zealand ascidian *Pseudodistoma opacum*. J Nat Prod.

[86] Yusuf H, Mustofa M, Susidarti RA, Asih PBS, Suryawati S (2013). A new quassinoid of four isolated compounds from extract *Eurycoma longifolia* Jack roots and their in vitro antimalarial activity. Int J Res Pharm Biomed Sci..

[87] Julianti T, De Mieri M, Zimmermann S, Ebrahimi SN, Kaiser M, Neuburger M (2014). HPLC-based activity profiling for antiplasmodial compounds in the traditional Indonesian medicinal plant *Carica papaya* L. J Ethnopharmacol.

[88] Pivatto M, Baccini LR, Sharma A, Nakabashi M, Danuello A, Viegas Júnior C (2014). Antimalarial activity of piperidine alkaloids from *Senna spectabilis* and semisynthetic derivatives. J Braz Chem Soc.

[89] Ilias M, Ibrahim MA, Khan SI, Jacob MR, Tekwani BL, Walker LA (2012). Pentacyclic ingamine alkaloids, a new antiplasmodial pharmacophore from the marine sponge *Petrosid* Ng5 Sp5. Planta Med.

[90] Mani L, Petek S, Valentin A, Chevalley S, Folcher E, Aalbersberg W (2011). The in vivo anti-plasmodial activity of haliclonacyclamine A, an alkaloid from the marine sponge, Haliclona sp. Nat Prod Res..

[91] Kumarihamy M, Fronczek FR, Ferreira D, Jacob M, Khan SI, Nanayakkara ND (2010). Bioactive 1,4-dihydroxy-5-phenyl-2-pyridinone alkaloids from *Septoria pistaciarum*. J Nat Prod.

[92] Gros E, Al-Mourabit A, Martin MT, Sorres J, Vacelet J, Frederich M (2014). Netamines H-N, tricyclic alkaloids from the marine sponge Biemna laboutei and their antimalarial activity. J Nat Prod.

[93] Scala F, Fattorusso E, Menna M, Taglialatela-Scafati O, Tierney M, Kaiser M (2010). Bromopyrrole alkaloids as lead compounds against protozoan parasites. Mar Drugs..

[94] Davis RA, Buchanan MS, Duffy S, Avery VM, Charman SA, Charman WN (2012). Antimalarial activity of pyrroloiminoquinones from the Australian marine sponge *Zyzzya* sp. J Med Chem.

[95] Na M, Ding Y, Wang B, Tekwani BL, Schinazi RF, Franzblau S (2009). Anti-infective discorhabdins from a deep-water Alaskan sponge of the genus *Latrunculia*. J Nat Prod.

[96] Neves JM, Matos C, Moutinho C, Queiroz G, Gomes LR (2009). Ethnopharmacological notes about ancient uses of medicinal plants in Trás-os-Montes (northern of Portugal). J Ethnopharmacol.

[97] Leporatti ML, Pavesi A, Posocco E (1985). Phytotherapy in the *Valnerina marche* (central Italy). J Ethnopharmacol.

[98] Althaus JB, Jerz G, Winterhalter P, Kaiser M, Brun R, Schmidt TJ (2014). Antiprotozoal activity of *Buxus sempervirens* and activity-guided isolation of *O*-tigloylcyclovirobuxeine-B as the main constituent active against *Plasmodium falciparum*. Molecules.

[99] Pan L, Acuña UM, Chai H, Park H-Y, Ninh TN, Van Thanh B (2015). New bioactive lupane triterpene coumaroyl esters isolated from *Buxus cochinchinensis*. Planta Med.

[100] Cheenpracha S, Boapun P, Limtharakul T, Laphookhieo S, Pyne SG (2017). Antimalarial and cytotoxic activities of pregnene-type steroidal alkaloids from *Holarrhena pubescens* roots. Nat Prod Res.

[101] Ma G, Sun Z, Sun Z, Yuan J, Wei H, Yang J (2014). Antimalarial diterpene alkaloids from the seeds of *Caesalpinia minax*. Fitoterapia.

[102] Hao B, Shen S-F, Zhao Q-J (2013). Cytotoxic and antimalarial Amaryllidaceae alkaloids from the bulbs of *Lycoris radiata*. Molecules.

[103] Presley CC, Krai P, Dalal S, Su Q, Cassera M, Goetz M (2016). New potently bioactive alkaloids from *Crinum erubescens*. Bioorg Med Chem.

[104] Presley CC, Du Y, Dalal S, Merino EF, Butler JH, Rakotonandrasana S (2017). Isolation, structure elucidation, and synthesis of antiplasmodial quinolones from *Crinum firmifolium*. Bioorg Med Chem.

[105] Yang X, Davis RA, Buchanan MS, Duffy S, Avery VM, Camp D (2010). Antimalarial bromotyrosine derivatives from the Australian marine sponge *Hyattella* sp. J Nat Prod.

[106] Xu M, Andrews KT, Birrell GW, Tran TL, Camp D, Davis RA (2011). Psammaplysin H, a new antimalarial bromotyrosine alkaloid from a marine sponge of the genus *Pseudoceratina*. Bioorg Med Chem Lett.

[107] Mani L, Jullian V, Mourkazel B, Valentin A, Dubois J, Cresteil T (2012). New antiplasmodial bromotyrosine derivatives from *Suberea ianthelliformis* Lendenfeld, 1888. Chem Biodiversity..

[108] Campos P-E, Wolfender J-L, Queiroz EF, Marcourt L, Al-Mourabit A, Frederich M (2017). Unguiculin A and ptilomycalins E-H, antimalarial guanidine alkaloids from the marine sponge Monanchora unguiculata. J Nat Prod.

[109] Davis RA, Duffy S, Fletcher S, Avery VM, Quinn RJ (2013). Thiaplakortones A–D: antimalarial thiazine alkaloids from the Australian marine sponge Plakortis lita. J Org Chem.

[110] Nogawa T, Kato N, Shimizu T, Okano A, Futamura Y, Takahashi S (2018). Wakodecalines A and B, new decaline metabolites isolated from a fungus *Pyrenochaetopsis* sp. RK10-F058. J Antibiot.

[111] Carroll AR, Duffy S, Avery VM (2010). Aplidiopsamine A, an antiplasmodial alkaloid from the temperate Australian ascidian, *Aplidiopsis confluata*. J Org Chem..

[112] Rahman AA, Samoylenko V, Jacob MR, Sahu R, Jain SK, Khan SI (2011). Antiparasitic and antimicrobial indolizidines from the leaves of *Prosopis glandulosa* var *glandulosa*. Planta Med.

[113] Komlaga G, Cojean S, Dickson RA, Beniddir MA, Suyyagh-Albouz S, Mensah ML (2016). Antiplasmodial activity of selected medicinal plants used to treat malaria in Ghana. Parasitol Res.

[114] Komlaga G, Genta-Jouve G, Cojean S, Dickson RA, Mensah ML, Loiseau PM (2017). Antiplasmodial *Securinega* alkaloids from *Phyllanthus fraternus*: discovery of natural (+)-allonorsecurinine. Tetrahedron Lett.

[115] Lacroix D, Prado S, Kamoga D, Kasenene J, Bodo B (2011). Structure and in vitro antiparasitic activity of constituents of *Citropsis articulata* root bark. J Nat Prod.

[116] Liew LP, Kaiser M, Copp BR (2013). Discovery and preliminary structure–activity relationship analysis of 1,14-sperminediphenylacetamides as potent and selective antimalarial lead compounds. Bioorg Med Chem Lett.

[117] Zofou D, Kengne ABO, Tene M, Ngemenya MN, Tane P, Titanji VP (2011). *In vitro* antiplasmodial activity and cytotoxicity of crude extracts and compounds from the stem bark of *Kigelia africana* (Lam.) Benth (Bignoniaceae). Parasitol Res.

[118] Zofou D, Tene M, Tane P, Titanji VP (2012). Antimalarial drug interactions of compounds isolated from *Kigelia africana* (Bignoniaceae) and their synergism with artemether, against the multidrug-resistant W2mef *Plasmodium falciparum* strain. Parasitol Res.

[119] Claudino VD, Da Silva KC, Cechinel Filho V, Yunes RA, Monache FD, Giménez A (2013). Drimanes from *Drimys brasiliensis* with leishmanicidal and antimalarial activity. Mem Inst Oswaldo Cruz.

[120] Mbaning BM, Lenta BN, Noungoué DT, Antheaume C, Fongang YF, Ngouela SA (2013). Antiplasmodial sesquiterpenes from the seeds of *Salacia longipes* var *camerunensis*. Phytochemistry.

[121] Dastan D, Salehi P, Gohari AR, Zimmermann S, Kaiser M, Hamburger M (2012). Disesquiterpene and sesquiterpene coumarins from *Ferula pseudalliacea,* and determination of their absolute configurations. Phytochemistry.

[122] Daengrot C, Rukachaisirikul V, Tansakul C, Thongpanchang T, Phongpaichit S, Bowornwiriyapan K (2015). Eremophilane sesquiterpenes and diphenyl thioethers from the soil fungus *Penicillium copticola* PSU-RSPG138. J Nat Prod.

[123] Hemtasin C, Kanokmedhakul S, Kanokmedhakul K, Hahnvajanawong C, Soytong K, Prabpai S (2011). Cytotoxic pentacyclic and tetracyclic aromatic sesquiterpenes from *Phomopsis archeri*. J Nat Prod.

[124] White AM, Pierens GK, Skinner-Adams T, Andrews KT, Bernhardt PV, Krenske EH (2015). Antimalarial isocyano and isothiocyanato sesquiterpenes with tri-and bicyclic skeletons from the nudibranch *Phyllidia ocellata*. J Nat Prod.

[125] Young RM, Adendorff MR, Wright AD, Davies-Coleman MT (2015). Antiplasmodial activity: the first proof of inhibition of heme crystallization by marine isonitriles. Eur J Med Chem.

[126] Morita H, Mori R, Deguchi J, Oshimi S, Hirasawa Y, Ekasari W (2012). Antiplasmodial decarboxyportentol acetate and 3,4-dehydrotheaspirone from *Laumoniera bruceadelpha*. J Nat Med.

[127] Zhou B, Wu Y, Dalal S, Merino EF, Liu Q-F, Xu C-H (2016). Nanomolar antimalarial agents against chloroquine-resistant *Plasmodium falciparum* from medicinal plants and their structure–activity relationships. J Nat Prod.

[128] Jansen O, Angenot L, Tits M, Nicolas JP, De Mol P, Nikiéma J-B (2010). Evaluation of 13 selected medicinal plants from Burkina Faso for their antiplasmodial properties. J Ethnopharmacol.

[129] Jansen O, Tits M, Angenot L, Nicolas J-P, De Mol P, Nikiema J-B (2012). Anti-plasmodial activity of *Dicoma tomentosa* (Asteraceae) and identification of urospermal A-15-O-acetate as the main active compound. Malar J..

[130] Becker JV, Merwe MM, van Brummelen AC, Pillay P, Crampton BG, Mmutlane EM (2011). *In vitro* anti-plasmodial activity of *Dicoma anomala* subsp. *gerrardii* (Asteraceae): identification of its main active constituent, structure-activity relationship studies and gene expression profiling. Malar J..

[131] Du Y, Pearce KC, Dai Y, Krai P, Dalal S, Cassera MB (2017). Antiplasmodial sesquiterpenoid lactones from *Trichospira verticillata*: structure elucidation by spectroscopic methods and comparison of experimental and calculated ECD data. J Nat Prod.

[132] Liu Y, Rakotondraibe LH, Brodie PJ, Wiley JD, Cassera MB, Goetz M (2014). Antiproliferative and antimalarial sesquiterpene lactones from *Piptocoma antillana* from Puerto Rico. Nat Prod Commun.

[133] Bero J, Ganfon H, Jonville M-C, Frédérich M, Gbaguidi F, DeMol P (2009). *In vitro* antiplasmodial activity of plants used in Benin in traditional medicine to treat malaria. J Ethnopharmacol.

[134] Ganfon H, Bero J, Tchinda AT, Gbaguidi F, Gbenou J, Moudachirou M (2012). Antiparasitic activities of two sesquiterpenic lactones isolated from *Acanthospermum hispidum* DC. J Ethnopharmacol.

[135] Toyang NJ, Krause MA, Fairhurst RM, Tane P, Bryant J, Verpoorte R (2013). Antiplasmodial activity of sesquiterpene lactones and a sucrose ester from *Vernonia guineensis* Benth (Asteraceae). J Ethnopharmacol.

[136] Maas M, Hensel A, da Costa FB, Brun R, Kaiser M, Schmidt TJ (2011). An unusual dimeric guaianolide with antiprotozoal activity and further sesquiterpene lactones from *Eupatorium perfoliatum*. Phytochemistry.

[137] Ma G, Wu H, Chen D, Zhu N, Zhu Y, Sun Z (2015). Antimalarial and antiproliferative cassane diterpenes of *Caesalpinia sappan*. J Nat Prod.

[138] Nondo RS, Erasto P, Moshi MJ, Zacharia A, Masimba PJ, Kidukuli AW (2016). *In vivo* antimalarial activity of extracts of Tanzanian medicinal plants used for the treatment of malaria. J Adv Pharm Technol Res..

[139] Nondo RSO, Moshi MJ, Erasto P, Masimba PJ, Machumi F, Kidukuli AW (2017). Anti-plasmodial activity of norcaesalpin D and extracts of four medicinal plants used traditionally for treatment of malaria. BMC Complementary Altern Med..

[140] Liu J, He X-F, Wang G-H, Merino EF, Yang S-P, Zhu R-X (2013). Aphadilactones A-D, four diterpenoid dimers with DGAT inhibitory and antimalarial activities from a Meliaceae plant. J Org Chem.

[141] Zhang H, Liu J, Gan L-S, Dalal S, Cassera MB, Yue J-M (2016). Antimalarial diterpenoid dimers of a new carbon skeleton from *Aphanamixis grandifolia*. Org Biomol Chem.

[142] Yin J-P, Gu M, Li Y, Nan F-J (2014). Total synthesis of aphadilactones A-D. J Org Chem.

[143] Palacpac NMQ, Hiramine Y, Seto S, Hiramatsu R, Horii T, Mitamura T (2004). Evidence that *Plasmodium falciparum* diacylglycerol acyltransferase is essential for intraerythrocytic proliferation. Biochem Biophys Res Commun.

[144] Gachet MS, Lecaro JS, Kaiser M, Brun R, Navarrete H, Muñoz RA (2010). Assessment of anti-protozoal activity of plants traditionally used in Ecuador in the treatment of leishmaniasis. J Ethnopharmacol.

[145] Gachet MS, Kunert O, Kaiser M, Brun R, Zehl M, Keller W (2011). Antiparasitic compounds from *Cupania cinerea* with activities against *Plasmodium falciparum* and *Trypanosoma brucei rhodesiense*. J Nat Prod.

[146] Kumar R, Duffy S, Avery VM, Davis RA (2017). Synthesis of antimalarial amide analogues based on the plant serrulatane diterpenoid 3,7,8-trihydroxyserrulat-14-en-19-oic acid. Bioorg Med Chem Lett.

[147] Zhou B, Wu Y, Dalal S, Cassera MB, Yue J-M (2016). Euphorbesulins A-P, structurally diverse diterpenoids from *Euphorbia esula*. J Nat Prod.

[148] Ebrahimi SN, Zimmermann S, Zaugg J, Smiesko M, Brun R, Hamburger M (2013). Abietane diterpenoids from *Salvia sahendica*—antiprotozoal activity and determination of their absolute configurations. Planta Med.

[149] González MA, Clark J, Connelly M, Rivas F (2014). Antimalarial activity of abietane ferruginol analogues possessing a phthalimide group. Bioorg Med Chem Lett.

[150] Chanthathamrongsiri N, Yuenyongsawad S, Wattanapiromsakul C, Plubrukarn A (2012). Bifunctionalized amphilectane diterpenes from the sponge *Stylissa* cf. *massa*. J Nat Prod.

[151] Avilés E, Prudhomme J, Le Roch KG, Rodríguez AD (2015). Structures, semisyntheses, and absolute configurations of the antiplasmodial α-substituted β-lactam monamphilectines B and C from the sponge *Svenzea flava*. Tetrahedron.

[152] Avilés E, Rodríguez AD (2010). Monamphilectine A, a potent antimalarial β-lactam from marine sponge *Hymeniacidon* sp: isolation, structure, semisynthesis, and bioactivity. Org Lett.

[153] White AM, Dao K, Vrubliauskas D, Könst ZA, Pierens GK, Mándi A (2017). Catalyst-controlled stereoselective synthesis secures the structure of the antimalarial isocyanoterpene pustulosaisonitrile-1. J Org Chem.

[154] Wright AD, Wang H, Gurrath M, König GM, Kocak G, Neumann G (2001). Inhibition of heme detoxification processes underlies the antimalarial activity of terpene isonitrile compounds from marine sponges. J Med Chem.

[155] Smyrniotopoulos V, Merten C, Kaiser M, Tasdemir D (2017). Bifurcatriol, a new antiprotozoal acyclic diterpene from the brown alga *Bifurcaria bifurcata*. Mar Drugs..

[156] Hata Y, De Mieri M, Ebrahimi SN, Mokoka T, Fouche G, Kaiser M (2014). Identification of two new phenathrenones and a saponin as antiprotozoal constituents of *Drypetes gerrardii*. Phytochem Lett.

[157] Seephonkai P, Pyne SG, Willis AC, Lie W (2013). Bioactive compounds from the roots of *Strophioblachia fimbricalyx*. J Nat Prod.

[158] Cai S, Risinger AL, Nair S, Peng J, Anderson TJ, Du L (2015). Identification of compounds with efficacy against malaria parasites from common North American plants. J Nat Prod.

[159] Bickiia J, Tchouyab G, Tchouankeub J, Tsamo E (2007). The antiplasmodial agents of the stem bark of *Entandrophragma angolense* (Meliaceae). Afr J Tradit Complement Altern Med.

[160] Happi GM, Kouam SF, Talontsi FM, Zühlke S, Lamshöft M, Spiteller M (2015). Minor secondary metabolites from the bark of *Entandrophragma congoense* (Meliaceae). Fitoterapia.

[161] Happi GM, Kouam SF, Talontsi FM, Lamshöft M, Zühlke S, Bauer JO (2015). Antiplasmodial and cytotoxic triterpenoids from the bark of the Cameroonian medicinal plant *Entandrophragma congoënse*. J Nat Prod.

[162] Greve HL, Kaiser M, Brun R, Schmidt TJ (2017). Terpenoids from the oleo-gum-resin of *Boswellia serrata* and their antiplasmodial effects *in vitro*. Planta Med.

[163] Ramalhete C, da Cruz FP, Lopes D, Mulhovo S, Rosário VE, Prudêncio M (2011). Triterpenoids as inhibitors of erythrocytic and liver stages of *Plasmodium* infections. Bioorg Med Chem.

[164] Ramalhete C, Lopes D, Molnár J, Mulhovo S, Rosário VE, Ferreira M-JU (2011). Karavilagenin C derivatives as antimalarials. Bioorg Med Chem.

[165] Irungu BN, Rukunga GM, Mungai GM, Muthaura CN (2007). *In vitro* antiplasmodial and cytotoxicity activities of 14 medicinal plants from Kenya. S Afr J Bot.

[166] Irungu BN, Adipo N, Orwa JA, Kimani F, Heydenreich M, Midiwo JO (2015). Antiplasmodial and cytotoxic activities of the constituents of *Turraea robusta* and *Turraea nilotica*. J Ethnopharmacol.

[167] Namukobe J, Kasenene JM, Kiremire BT, Byamukama R, Kamatenesi-Mugisha M, Krief S (2011). Traditional plants used for medicinal purposes by local communities around the Northern sector of Kibale National Park, Uganda. J Ethnopharmacol..

[168] Namukobe J, Kiremire BT, Byamukama R, Kasenene JM, Dumontet V, Guéritte F (2014). Cycloartane triterpenes from the leaves of *Neoboutonia macrocalyx* L. Phytochemistry.

[169] Farimani MM, Bahadori MB, Taheri S, Ebrahimi SN, Zimmermann S, Brun R (2011). Triterpenoids with rare carbon skeletons from *Salvia hydrangea*: antiprotozoal activity and absolute configurations. J Nat Prod.

[170] Foubert K, Gorella T, Faizal A, Cos P, Maes L, Apers S (2016). Triterpenoid saponins from *Maesa argentea* leaves. Planta Med.

[171] Ma K, Ren J, Han J, Bao L, Li L, Yao Y (2014). Ganoboninketals A-C, antiplasmodial 3,4-seco-27-norlanostane triterpenes from Ganoderma boninense Pat. J Nat Prod.

[172] Banzouzi J, Soh PN, Ramos S, Toto P, Cavé A, Hemez J (2015). Samvisterin, a new natural antiplasmodial betulin derivative from *Uapaca paludosa* (Euphorbiaceae). J Ethnopharmacol.

[173] Yim T, Kanokmedhakul K, Kanokmedhakul S, Sanmanoch W, Boonlue S (2014). A new meroterpenoid tatenoic acid from the fungus *Neosartorya tatenoi* KKU-2NK23. Nat Prod Res.

[174] Sá MS, de Menezes MN, Krettli AU, Ribeiro IM, Tomassini TC, dos Santos R (2011). Antimalarial activity of physalins B, D, F, and G. J Nat Prod.

[175] Ochieng CO, Manguro LA, Owuor PO, Akala H (2013). Voulkensin C-E, new 11-oxocassane-type diterpenoids and a steroid glycoside from *Caesalpinia volkensii* stem bark and their antiplasmodial activities. Bioorg Med Chem Lett.

[176] Meesala S, Gurung P, Karmodiya K, Subrayan P, Watve MG (2017). Isolation and structure elucidation of halymeniaol, a new antimalarial sterol derivative from the red alga *Halymenia floresii*. J Asian Nat Prod Res.

[177] Regalado EL, Tasdemir D, Kaiser M, Cachet N, Amade P, Thomas OP (2010). Antiprotozoal steroidal saponins from the marine sponge *Pandaros acanthifolium*. J Nat Prod.

[178] Huffman MA (1997). Current evidence for self-medication in primates: a multidisciplinary perspective. Am J Phys Anthropol.

[179] Newton-Fisher NE (1999). The diet of chimpanzees in the Budongo Forest Reserve, Uganda. Afr J Ecol.

[180] Obbo C, Makanga B, Mulholland D, Coombes P, Brun R (2013). Antiprotozoal activity of *Khaya anthotheca,* (Welv.) CDC a plant used by chimpanzees for self-medication. J Ethnopharmacol.

[181] Kassim OO, Loyevsky M, Amonoo H, Lashley L, Ako-Nai KA, Gordeuk VR (2009). Inhibition of in vitro growth of *Plasmodium falciparum* by *Pseudocedrela kotschyi* extract alone and in combination with *Fagara zanthoxyloides* extract. Trans R Soc Trop Med Hyg.

[182] Sidjui LS, Nganso YO, Toghueo RM, Wakeu BN, Dameue JT, Mkounga P (2018). Kostchyienones A and B, new antiplasmodial and cytotoxicity of limonoids from the roots of *Pseudocedrela kotschyi* (Schweinf) Harms. Z Naturforsch C Bio Sci..

[183] Vigneron M, Deparis X, Deharo E, Bourdy G (2005). Antimalarial remedies in French Guiana: a knowledge attitudes and practices study. J Ethnopharmacol.

[184] Bertania S, Bourdyb G, Landaua I, Robinsonc J, Esterred P, Deharo E (2005). Evaluation of French Guiana traditional antimalarial remedies. J Ethnopharmacol.

[185] Bertani S, Houel E, Stien D, Chevolot L, Jullian V, Garavito G (2006). Simalikalactone D is responsible for the antimalarial properties of an Amazonian traditional remedy made with *Quassia amara* L. (Simaroubaceae). J Ethnopharmacol.

[186] Bertani S, Houël E, Jullian V, Bourdy G, Valentin A, Stien D (2012). New findings on simalikalactone D, an antimalarial compound from *Quassia amara* L. (Simaroubaceae). Exp Parasitol.

[187] Mishra K, Chakraborty D, Pal A, Dey N (2010). *Plasmodium falciparum:* in vitro interaction of quassin and neo-quassin with artesunate, a hemisuccinate derivative of artemisinin. Exp Parasitol.

[188] Chumkaew P, Pechwang J, Srisawat T (2017). Two new antimalarial quassinoid derivatives from the stems of *Brucea javanica*. J Nat Med.

[189] Chumkaew P, Srisawat T (2017). Antimalarial and cytotoxic quassinoids from the roots of *Brucea javanica*. J Asian Nat Prod Res.

[190] Tona L, Ngimbi N, Tsakala M, Mesia K, Cimanga K, Apers S (1999). Antimalarial activity of 20 crude extracts from nine African medicinal plants used in Kinshasa, Congo. J Ethnopharmacol..

[191] Oluwatosin A, Tolulope A, Ayokulehin K, Patricia O, Aderemi K, Catherine F (2014). Antimalarial potential of kolaviron, a biflavonoid from *Garcinia kola* seeds, against *Plasmodium berghei* infection in Swiss albino mice. Asian Pac J Trop Med..

[192] Konziase B (2015). Protective activity of biflavanones from *Garcinia kola* against *Plasmodium* infection. J Ethnopharmacol.

[193] Azebaze AGB, Teinkela JEM, Nguemfo EL, Valentin A, Dongmo AB, Vardamides JC (2015). Antiplasmodial activity of some phenolic compounds from Cameroonians *Allanblackia*. Afr Health Sci..

[194] Bourjot M, Apel C, Martin M-T, Grellier P, Guéritte F, Litaudon M (2010). Antiplasmodial, antitrypanosomal, and cytotoxic activities of prenylated flavonoids isolated from the stem bark of *Artocarpus styracifolius*. Planta Med.

[195] Zakaria I, Ahmat N, Jaafar FM, Widyawaruyanti A (2012). Flavonoids with antiplasmodial and cytotoxic activities of *Macaranga triloba*. Fitoterapia.

[196] Juma WP, Akala HM, Eyase FL, Muiva LM, Heydenreich M, Okalebo FA (2011). Terpurinflavone: an antiplasmodial flavone from the stem of *Tephrosia purpurea*. Phytochem Lett.

[197] Muiva-Mutisya L, Macharia B, Heydenreich M, Koch A, Akala HM, Derese S (2014). 6α-Hydroxy-α-toxicarol and (+)-tephrodin with antiplasmodial activities from *Tephrosia* species. Phytochem Lett.

[198] Atilaw Y, Muiva-Mutisya L, Ndakala A, Akala HM, Yeda R, Wu YJ (2017). Four prenylflavone derivatives with antiplasmodial activities from the stem of *Tephrosia purpurea* subsp. *leptostachya*. Molecules.

[199] Muiva-Mutisya LM, Atilaw Y, Heydenreich M, Koch A, Akala HM, Cheruiyot AC, Brown ML, Irungu B, Okalebo FA, Derese S, Mutai C (2018). Antiplasmodial prenylated flavanonols from Tephrosia subtriflora. Nat Prod Res.

[200] Atilaw Y, Duffy S, Heydenreich M, Muiva-Mutisya L, Avery VM, Erdélyi M (2017). Three chalconoids and a pterocarpene from the roots of *Tephrosia aequilata*. Molecules.

[201] Frolich S, Schubert C, Bienzle U, Jenett-Siems K (2005). *In vitro* antiplasmodial activity of prenylated chalcone derivatives of hops (*Humulus lupulus*) and their interaction with haemin. J Antimicrob Chemother.

[202] Kaou AM, Mahiou-Leddet V, Hutter S, Aïnouddine S, Hassani S, Yahaya I (2008). Antimalarial activity of crude extracts from nine African medicinal plants. J Ethnopharmacol.

[203] Sashidhara KV, Singh SP, Singh SV, Srivastava RK, Srivastava K, Saxena J (2013). Isolation and identification of β-hematin inhibitors from *Flacourtia indica* as promising antiplasmodial agents. Eur J Med Chem.

[204] Abdalla MA, Laatsch H (2012). Flavonoids from Sudanese *Albizia zygia* (Leguminosae, subfamily Mimosoideae), a plant with antimalarial potency. Afr J Tradit Complement Altern Med.

[205] Zininga T, Ramatsui L, Makhado PB, Makumire S, Achilinou I, Hoppe H (2017). (−)-Epigallocatechin-3-gallate inhibits the chaperone activity of *Plasmodium falciparum* Hsp70 chaperones and abrogates their association with functional partners. Molecules.

[206] Sannella AR, Messori L, Casini A, Vincieri FF, Bilia AR, Majori G (2007). Antimalarial properties of green tea. Biochem Biophys Res Commun.

[207] Chung IM, Ghimire BK, Kang EY, Moon HI (2010). Antiplasmodial and cytotoxic activity of khellactone derivatives from *Angelica purpuraefolia* Chung. Phytother Res..

[208] Chung IM, Seo SH, Kang EY, Park WH, Park SD, Moon HI (2010). Antiplasmodial activity of isolated compounds from *Carpesium divaricatum*. Phytother Res..

[209] Du Y, Abedi AK, Valenciano AL, Fernández-Murga ML, Cassera MB, Rasamison VE (2017). Isolation of the new antiplasmodial butanolide, malleastrumolide A, from *Malleastrum* sp. (Meliaceae) from Madagascar. Chem Biodiversity..

[210] Zofou D, Tene M, Ngemenya MN, Tane P, Titanji VP (2011). *In vitro* antiplasmodial activity and cytotoxicity of extracts of selected medicinal plants used by traditional healers of Western Cameroon. Malar Res Treat..

[211] Zofou D, Tematio EL, Ntie-Kang F, Tene M, Ngemenya MN, Tane P (2013). New antimalarial hits from *Dacryodes edulis* (Burseraceae)—Part I: isolation, in vitro activity, in silico “drug-likeness” and pharmacokinetic profiles. PLoS One.

[212] Gadetskaya AV, Mohamed SM, Tarawneh AH, Mohamed NM, Ma G, Ponomarev BN (2017). Phytochemical characterization and biological activity of secondary metabolites from three *Limonium* species. Med Chem Res.

[213] Tangmouo JG, Ho R, Matheeussen A, Lannang AM, Komguem J, Messi BB (2010). Antimalarial activity of extract and norbergenin derivatives from the stem bark of Diospyros sanza-minika A. Chevalier (Ebenaceae). Phytother Res..

[214] Ndjonka D, Bergmann B, Agyare C, Zimbres FM, Lüersen K, Hensel A (2012). *In vitro* activity of extracts and isolated polyphenols from West African medicinal plants against *Plasmodium falciparum*. Parasitol Res.

[215] Soh PN, Witkowski B, Olagnier D, Nicolau M-L, Garcia-Alvarez M-C, Berry A (2009). *In vitro* and in vivo properties of ellagic acid in malaria treatment. Antimicrob Agents Chemother.

[216] Gachet MS, Kunert O, Kaiser M, Brun R, Munoz RA, Bauer R (2010). Jacaranone-derived glucosidic esters from *Jacaranda glabra* and their activity against *Plasmodium falciparum*. J Nat Prod.

[217] Latif A, Du Y, Dalal SR, Merino EF, Cassera MB, Goetz M (2017). Bioactive neolignans and other compounds from *Magnolia grandiflora* L.: isolation and antiplasmodial activity. Chem Biodiversity..

[218] Rakotondraibe LH, Graupner PR, Xiong Q, Olson M, Wiley JD, Krai P (2015). Neolignans and other metabolites from *Ocotea cymosa* from the Madagascar rain forest and their biological activities. J Nat Prod.

[219] Ovenden SP, Cobbe M, Kissell R, Birrell GW, Chavchich M, Edstein MD (2010). Phenolic glycosides with antimalarial activity from *Grevillea* “Poorinda Queen”. J Nat Prod.

[220] Xiao H, Rao Ravu R, Tekwani BL, Li W, Liu W-B, Jacob MR (2017). Biological evaluation of phytoconstituents from *Polygonum hydropiper*. Nat Prod Res.

[221] Iwatsuki M, Takada S, Mori M, Ishiyama A, Namatame M, Nishihara-Tsukashima A (2011). In vitro and in vivo antimalarial activity of puberulic acid and its new analogs, viticolins A-C, produced by Penicillium sp. FKI-4410. J Antibiot.

[222] Nyandoro SS, Munissi JJ, Gruhonjic A, Duffy S, Pan F, Puttreddy R (2016). Polyoxygenated cyclohexenes and other constituents of *Cleistochlamys kirkii* leaves. J Nat Prod.

[223] Kornsakulkarn J, Thongpanchang C, Chainoy R, Choowong W, Nithithanasilp S, Thongpanchang T (2010). Bioactive metabolites from cultures of basidiomycete *Favolaschia tonkinensis*. J Nat Prod.

[224] Lenta BN, Kamdem LM, Ngouela S, Tantangmo F, Devkota KP, Boyom FF (2011). Antiplasmodial constituents from the fruit pericarp of *Pentadesma butyracea*. Planta Med.

[225] Zelefack F, Guilet D, Fabre N, Bayet C, Chevalley S, Ngouela S (2009). Cytotoxic and antiplasmodial xanthones from *Pentadesma butyracea*. J Nat Prod.

[226] Upegui Y, Robledo SM, Gil Romero JF, Quiñones W, Archbold R, Torres F (2015). In vivo antimalarial activity of α-mangostin and the new xanthone δ-mangostin. Phytother Res..

[227] Focho D, Ndam W, Fonge B (2009). Medicinal plants of Aguambu-Bamumbu in the Lebialem highlands, southwest province of Cameroon. Afr J Pharm Pharmacol..

[228] Zofou D, Kowa TK, Wabo HK, Ngemenya MN, Tane P, Titanji VP (2011). *Hypericum lanceolatum* (Hypericaceae) as a potential source of new anti-malarial agents: a bioassay-guided fractionation of the stem bark. Malar J..

[229] Guizzunti G, Batova A, Chantarasriwong O, Dakanali M, Theodorakis EA (2012). Subcellular localization and activity of gambogic acid. ChemBioChem.

[230] Ke H, Morrisey JM, Qu S, Chantarasriwong O, Mather MW, Theodorakis EA (2017). Caged *Garcinia* xanthones, a novel chemical scaffold with potent antimalarial activity. Antimicrob Agents Chemother.

[231] Calcul L, Waterman C, Ma WS, Lebar MD, Harter C, Mutka T (2013). Screening mangrove endophytic fungi for antimalarial natural products. Mar Drugs..

[232] Harinantenaina L, Bowman JD, Brodie PJ, Slebodnick C, Callmander MW, Rakotobe E (2013). Antiproliferative and antiplasmodial dimeric phloroglucinols from *Mallotus oppositifolius* from the Madagascar dry forest. J Nat Prod.

[233] Eaton AL, Dalal S, Cassera MB, Zhao S, Kingston DG (2016). Synthesis and antimalarial activity of mallatojaponin C and related compounds. J Nat Prod.

[234] Marti G, Eparvier V, Moretti C, Prado S, Grellier P, Hue N (2010). Antiplasmodial benzophenone derivatives from the root barks of *Symphonia globulifera* (Clusiaceae). Phytochemistry.

[235] Su Q, Dalal S, Goetz M, Cassera MB, Kingston DG (2016). Antiplasmodial phloroglucinol derivatives from *Syncarpia glomulifera*. Bioorg Med Chem.

[236] Senadeera SP, Duffy S, Avery VM, Carroll AR (2017). Antiplasmodial β-triketones from the flowers of the Australian tree *Angophora woodsiana*. Bioorg Med Chem Lett.

[237] Hiranrat A, Mahabusarakam W, Carroll AR, Duffy S, Avery VM (2011). Tomentosones A and B, hexacyclic phloroglucinol derivatives from the Thai shrub *Rhodomyrtus tomentosa*. J Org Chem.

[238] Carroll AR, Avery VM, Duffy S, Forster PI, Guymer GP (2013). Watsonianone A-C, anti-plasmodial β-triketones from the Australian tree, *Corymbia watsoniana*. Org Biomol Chem.

[239] Dai Y, Harinantenaina L, Bowman JD, Da Fonseca IO, Brodie PJ, Goetz M (2014). Isolation of antiplasmodial anthraquinones from *Kniphofia ensifolia*, and synthesis and structure–activity relationships of related compounds. Bioorg Med Chem.

[240] Wube AA, Bucar F, Asres K, Gibbons S, Rattray L, Croft SL (2005). Antimalarial compounds from *Kniphofia foliosa* roots. Phytother Res..

[241] Abdissa N, Induli M, Akala HM, Heydenreich M, Midiwo JO, Ndakala A (2013). Knipholone cyclooxanthrone and an anthraquinone dimer with antiplasmodial activities from the roots of *Kniphofia foliosa*. Phytochem Lett.

[242] Isaka M, Palasarn S, Tobwor P, Boonruangprapa T, Tasanathai K (2012). Bioactive anthraquinone dimers from the leafhopper pathogenic fungus *Torrubiella* sp. BCC 28517. J Antibiot.

[243] Supong K, Thawai C, Suwanborirux K, Choowong W, Supothina S, Pittayakhajonwut P (2012). Antimalarial and antitubercular C-glycosylated benz[α]anthraquinones from the marine-derived *Streptomyces* sp. BCC45596. Phytochem Lett.

[244] Osman CP, Ismail NH, Ahmad R, Ahmat N, Awang K, Jaafar FM (2010). Anthraquinones with antiplasmodial activity from the roots of *Rennellia elliptica* Korth. (Rubiaceae). Molecules.

[245] Wanyoike G, Chhabra S, Lang’at-Thoruwa C, Omar S (2004). Brine shrimp toxicity and antiplasmodial activity of five Kenyan medicinal plants. J Ethnopharmacol.

[246] Endale M, Alao JP, Akala HM, Rono NK, Eyase FL, Derese S (2012). Antiplasmodial quinones from *Pentas longiflora* and *Pentas lanceolata*. Planta Med.

[247] Tantangmo F, Lenta B, Boyom F, Ngouela S, Kaiser M, Tsamo E (2010). Antiprotozoal activities of some constituents of *Markhamia tomentosa* (Bignoniaceae). Ann Trop Med Parasitol.

[248] Simonsen HT, Nordskjold JB, Smitt UW, Nyman U, Palpu P, Joshi P (2001). In vitro screening of Indian medicinal plants for antiplasmodial activity. J Ethnopharmacol.

[249] Thiengsusuk A, Chaijaroenkul W, Na-Bangchang K (2013). Antimalarial activities of medicinal plants and herbal formulations used in Thai traditional medicine. Parasitol Res.

[250] Sumsakul W, Plengsuriyakarn T, Chaijaroenkul W, Viyanant V, Karbwang J, Na-Bangchang K (2014). Antimalarial activity of plumbagin in vitro and in animal models. BMC Complementary Altern Med..

[251] Moreno E, Varughese T, Spadafora C, Arnold AE, Coley PD, Kursar TA (2011). Chemical constituents of the new endophytic fungus *Mycosphaerella* sp. novds and their anti-parasitic activity. Nat Prod Commun.

[252] Kumarihamy M, Khan SI, Jacob M, Tekwani BL, Duke SO, Ferreira D (2012). Antiprotozoal and antimicrobial compounds from the plant pathogen *Septoria pistaciarum*. J Nat Prod.

[253] Longeon A, Copp BR, Roué M, Dubois J, Valentin A, Petek S (2010). New bioactive halenaquinone derivatives from South Pacific marine sponges of the genus *Xestospongia*. Bioorg Med Chem.

[254] Supong K, Sripreechasak P, Tanasupawat S, Danwisetkanjana K, Rachtawee P, Pittayakhajonwut P (2017). Investigation on antimicrobial agents of the terrestrial *Streptomyces* sp. BCC71188. Appl Microbiol Biotechnol.

[255] Panthama N, Kanokmedhakul S, Kanokmedhakul K, Soytong K (2011). Cytotoxic and antimalarial azaphilones from *Chaetomium longirostre*. J Nat Prod.

[256] Ledoux A, St-Gelais A, Cieckiewicz E, Jansen O, Bordignon A, Illien B (2017). Antimalarial activities of alkyl cyclohexenone derivatives isolated from the leaves of *Poupartia borbonica*. J Nat Prod.

[257] Farokhi F, Grellier P, Clément M, Roussakis C, Loiseau PM, Genin-Seward E (2013). Antimalarial activity of axidjiferosides, new β-galactosylceramides from the African sponge *Axinyssa djiferi*. Mar Drugs..

[258] Ferreira MC, Cantrell CL, Wedge DE, Gonçalves VN, Jacob MR, Khan S (2017). Antimycobacterial and antimalarial activities of endophytic fungi associated with the ancient and narrowly endemic neotropical plant *Vellozia gigantea* from Brazil. Mem Inst Oswaldo Cruz.

[259] Lane AL, Stout EP, Lin A-S, Prudhomme J, Le Roch K, Fairchild CR (2009). Antimalarial bromophycolides J–Q from the Fijian red alga *Callophycus serratus*. J Org Chem.

[260] Lin A-S, Stout EP, Prudhomme J, Roch KL, Fairchild CR, Franzblau SG (2010). Bioactive bromophycolides R–U from the Fijian red alga *Callophycus serratus*. J Nat Prod.

[261] Stout EP, Prudhomme J, Le Roch K, Fairchild CR, Franzblau SG, Aalbersberg W (2010). Unusual antimalarial meroditerpenes from tropical red macroalgae. Bioorg Med Chem Lett.

[262] Stout EP, Cervantes S, Prudhomme J, France S, La Clair JJ, Le Roch K (2011). Bromophycolide A targets heme crystallization in the human malaria parasite *Plasmodium falciparum*. ChemMedChem.

[263] Shao C-L, Linington RG, Balunas MJ, Centeno A, Boudreau P, Zhang C (2015). Bastimolide A, a potent antimalarial polyhydroxy macrolide from the marine cyanobacterium *Okeania hirsuta*. J Org Chem.

[264] Shao C-L, Mou X-F, Cao F, Spadafora C, Glukhov E, Gerwick L (2018). Bastimolide B, an antimalarial 24-membered marine macrolide possessing a *tert*-butyl group. J Nat Prod.

[265] Sirirak T, Kittiwisut S, Janma C, Yuenyongsawad S, Suwanborirux K, Plubrukarn A (2011). Kabiramides J and K, trisoxazole macrolides from the sponge *Pachastrissa nux*. J Nat Prod.

[266] Sirirak T, Brecker L, Plubrukarn A, Kabiramide L (2013). a new antiplasmodial trisoxazole macrolide from the sponge *Pachastrissa nux*. Nat Prod Res.

[267] Supong K, Thawai C, Choowong W, Kittiwongwattana C, Thanaboripat D, Laosinwattana C (2016). Antimicrobial compounds from endophytic *Streptomyces* sp. BCC72023 isolated from rice (*Oryza sativa* L.). Res Microbiol.

[268] Xu L, He Z, Xue J, Chen X, Wei X (2010). β-Resorcylic acid lactones from a *Paecilomyces* fungus. J Nat Prod.

[269] Shao C-L, Wu H-X, Wang C-Y, Liu Q-A, Xu Y, Wei M-Y (2011). Potent antifouling resorcylic acid lactones from the gorgonian-derived fungus *Cochliobolus lunatus*. J Nat Prod.

[270] Liu Q-A, Shao C-L, Gu Y-C, Blum M, Gan L-S, Wang K-L (2014). Antifouling and fungicidal resorcylic acid lactones from the sea anemone-derived fungus *Cochliobolus lunatus*. J Agric Food Chem.

[271] Zhang X-Q, Spadafora C, Pineda LM, Ng MG, Sun J-H, Wang W (2017). Discovery, semisynthesis, antiparasitic and cytotoxic evaluation of 14-membered resorcylic acidlLactones and their derivatives. Sci Rep..

[272] Tripathi A, Puddick J, Prinsep MR, Rottmann M, Tan LT (2010). Lagunamides A and B: cytotoxic and antimalarial cyclodepsipeptides from the marine cyanobacterium *Lyngbya majuscula*. J Nat Prod.

[273] Tripathi A, Puddick J, Prinsep MR, Rottmann M, Chan KP, Chen DYK (2011). Lagunamide C, a cytotoxic cyclodepsipeptide from the marine cyanobacterium *Lyngbya majuscula*. Phytochemistry.

[274] Raju R, Khalil ZG, Piggott AM, Blumenthal A, Gardiner DL, Skinner-Adams TS (2014). Mollemycin A: an antimalarial and antibacterial glyco-hexadepsipeptide-polyketide from an Australian marine-derived *Streptomyces* sp. (CMB-M0244). Org Lett.

[275] Son S, Ko SK, Kim JW, Lee JK, Jang M, Ryoo IJ (2016). Structures and biological activities of azaphilones produced by *Penicillium* sp. KCB11A109 from a ginseng field. Phytochemistry.

[276] Ibrahim SR, Abdallah HM, Elkhayat ES, Al Musayeib NM, Asfour HZ, Zayed MF (2018). Fusaripeptide A: new antifungal and anti-malarial cyclodepsipeptide from the endophytic fungus *Fusarium* sp. J Asian Nat Prod Res.

[277] LaMonte GM, Almaliti J, Bibo-Verdugo B, Keller L, Zou BY, Yang J (2017). Development of a potent inhibitor of the *Plasmodium* proteasome with reduced mammalian toxicity. J Med Chem.

[278] Cheng KC-C, Cao S, Raveh A, MacArthur R, Dranchak P, Chlipala G (2015). Actinoramide A identified as a potent antimalarial from titration-based screening of marine natural product extracts. J Nat Prod.

[279] Von Bargen KW, Niehaus E-M, Bergander K, Brun R, Tudzynski B, Humpf H-U (2013). Structure elucidation and antimalarial activity of apicidin F: an apicidin-like compound produced by *Fusarium fujikuroi*. J Nat Prod.

[280] Tuenter E, Foubert K, Staerk D, Apers S, Pieters L (2017). Isolation and structure elucidation of cyclopeptide alkaloids from *Ziziphus nummularia* and *Ziziphus spina*-*christi* by HPLC-DAD-MS and HPLC-PDA-(HRMS)-SPE-NMR. Phytochemistry.

[281] Tuenter E, Segers K, Kang KB, Viaene J, Sung SH, Cos P (2017). Antiplasmodial activity, cytotoxicity and structure-activity relationship study of cyclopeptide alkaloids. Molecules.

[282] Yu J, Zhou B, Dalal S, Liu Q, Cassera MB, Yue J (2018). Cipaferoids A-C, three limonoids represent two different scaffolds from Cipadessa baccifera. Chin J Chem.

[283] Schulze CJ, Navarro G, Ebert D, DeRisi J, Linington RG (2015). Salinipostins A-K, long-chain bicyclic phosphotriesters as a potent and selective antimalarial chemotype. J Org Chem.

[284] Stolze SC, Deu E, Kaschani F, Li N, Florea BI, Richau KH (2012). The antimalarial natural product symplostatin 4 is a nanomolar inhibitor of the food vacuole falcipains. Chem Biol.

[285] Olliaro P (2001). Mode of action and mechanisms of resistance for antimalarial drugs. Pharmacol Ther.

[286] Olliaro P, Wells T (2009). The global portfolio of new antimalarial medicines under development. Clin Pharmacol Ther.

[287] Rottmann M, McNamara C, Yeung BK, Lee MC, Zou B, Russell B (2010). Spiroindolones, a potent compound class for the treatment of malaria. Science.

[288] Kissinger JC, Brunk BP, Crabtree J, Fraunholz MJ, Gajria B, Milgram AJ (2002). The *Plasmodium* genome database. Nature.

[289] López ML, Vommaro R, Zalis M, de Souza W, Blair S, Segura C (2010). Induction of cell death on *Plasmodium falciparum* asexual blood stages by *Solanum nudum* steroids. Parasitol Int.

[290] Tasdemir D, Topaloglu B, Perozzo R, Brun R, O’Neill R, Carballeira NM (2007). Marine natural products from the Turkish sponge *Agelas oroides* that inhibit the enoyl reductases from *Plasmodium falciparum*, *Mycobacterium tuberculosis* and *Escherichia coli*. Bioorg Med Chem.

[291] Kirmizibekmez H, Calis I, Perozzo R, Brun R, Donmez AA, Linden A (2004). Inhibiting activities of the secondary metabolites of *Phlomis brunneogaleata* against parasitic protozoa and plasmodial enoyl-ACP reductase, a crucial enzyme in fatty acid biosynthesis. Planta Med.

[292] Karioti A, Skaltsa H, Linden A, Perozzo R, Brun R, Tasdemir D (2007). Anthecularin: a novel sesquiterpene lactone from *Anthemis auriculata* with antiprotozoal activity. J Org Chem.

[293] Karioti A, Skaltsa H, Zhang X, Tonge PJ, Perozzo R, Kaiser M (2008). Inhibiting enoyl-ACP reductase (FabI) across pathogenic microorganisms by linear sesquiterpene lactones from *Anthemis auriculata*. Phytomedicine.

[294] Bankeu JJ, Khayala R, Lenta BN, Noungoué DT, Ngouela SA, Mustafa SA (2011). Isoflavone dimers and other bioactive constituents from the figs of *Ficus mucuso*. J Nat Prod.

[295] Lauinger IL, Vivas L, Perozzo R, Stairiker C, Tarun A, Zloh M (2013). Potential of lichen secondary metabolites against *Plasmodium* liver stage parasites with FAS-II as the potential target. J Nat Prod.

[296] Muhammad A, Anis I, Ali Z, Awadelkarim S, Khan A, Khalid A (2012). Methylenebissantin: a rare methylene-bridged bisflavonoid from *Dodonaea viscosa* which inhibits *Plasmodium falciparum* enoyl-ACP reductase. Bioorg Med Chem Lett.

[297] Tasdemir D, Lack G, Brun R, Rüedi P, Scapozza L, Perozzo R (2006). Inhibition of *Plasmodium falciparum* fatty acid biosynthesis: evaluation of FabG, FabZ, and FabI as drug targets for flavonoids. J Med Chem.

[298] Singh SV, Manhas A, Kumar Y, Mishra S, Shanker K, Khan F (2017). Antimalarial activity and safety assessment of *Flueggea virosa* leaves and its major constituent with special emphasis on their mode of action. Biomed Pharmacother.

[299] Singh DK, Cheema HS, Saxena A, Singh S, Darokar MP, Bawankule DU (2017). Fraxetin and ethyl acetate extract from *Lawsonia inermis* L. ameliorate oxidative stress in *P. berghei* infected mice by augmenting antioxidant defence system. Phytomedicine.

[300] Wahyuono S, Simanjuntak P (2013). Heme polymerization inhibitory activities of xanthone from *G. parvifolia* (Miq) Miq stem bark as an antimalarial agent. Asian J Chem.

[301] Singh SV, Manhas A, Singh SP, Mishra S, Tiwari N, Kumar P (2017). A phenolic glycoside from *Flacourtia indica* induces heme mediated oxidative stress in *Plasmodium falciparum* and attenuates malaria pathogenesis in mice. Phytomedicine.

[302] Mangoyi R, Hayeshi R, Ngadjui B, Ngandeu F, Bezabih M, Abegaz B (2010). Glutathione transferase from *Plasmodium falciparum*—Interaction with malagashanine and selected plant natural products. J Enzyme Inhib Med Chem.

[303] Skorokhod OA, Davalos-Schafler D, Gallo V, Valente E, Ulliers D, Notarpietro A (2015). Oxidative stress-mediated antimalarial activity of plakortin, a natural endoperoxide from the tropical sponge *Plakortis simplex*. Free Radical Biol Med.

[304] Kamkumo RG, Ngoutane AM, Tchokouaha LR, Fokou PV, Madiesse EA, Legac J (2012). Compounds from *Sorindeia juglandifolia* (Anacardiaceae) exhibit potent anti-plasmodial activities in vitro and *in vivo*. Malar J..

[305] Ishiyama A, Iwatsuki M, Yamamoto T, Miura H, Ōmura S, Otoguro K (2014). Antimalarial tropones and their *Plasmodium falciparum* glyoxalase I (pfGLOI) inhibitory activity. J Antibiot.

[306] Cockburn IL, Pesce E-R, Pryzborski JM, Davies-Coleman MT, Clark PG, Keyzers RA (2011). Screening for small molecule modulators of Hsp70 chaperone activity using protein aggregation suppression assays: inhibition of the plasmodial chaperone PfHsp70-1. Biol Chem.

[307] Eckstein-Ludwig U, Webb R, Van Goethem I, East J, Lee A, Kimura M (2003). Artemisinins target the SERCA of *Plasmodium falciparum*. Nature.

[308] Hoepfner D, McNamara CW, Lim CS, Studer C, Riedl R, Aust T (2012). Selective and specific inhibition of the *Plasmodium falciparum* lysyl-tRNA synthetase by the fungal secondary metabolite cladosporin. Cell Host Microbe.

[309] Birkholtz L-M, Coetzer TL, Mancama D, Leroy D, Alano P (2016). Discovering new transmission-blocking antimalarial compounds: challenges and opportunities. Trends Parasitol..

[310] Abay SM, Lucantoni L, Dahiya N, Dori G, Dembo EG, Esposito F (2015). *Plasmodium* transmission blocking activities of *Vernonia amygdalina* extracts and isolated compounds. Malar J..

[311] Moyo P, Botha ME, Nondaba S, Niemand J, Maharaj VJ, Eloff JN (2016). I*n vitro* inhibition of *Plasmodium falciparum* early and late stage gametocyte viability by extracts from eight traditionally used South African plant species. J Ethnopharmacol.

[312] Lucantoni L, Yerbanga RS, Lupidi G, Pasqualini L, Esposito F, Habluetzel A (2010). Transmission blocking activity of a standardized neem (*Azadirachta indica*) seed extract on the rodent malaria parasite *Plasmodium berghei* in its vector *Anopheles stephensi*. Malar J..

[313] Yerbanga R, Lucantoni L, Ouédraogo R, Da DF, Yaméogo K, Churcher T (2014). Transmission blocking activity of *Azadirachta indica* and *Guiera senegalensis* extracts on the sporogonic development of *Plasmodium falciparum* field isolates in *Anopheles coluzzii* mosquitoes. Parasites Vectors..

[314] Balaich JN, Mathias DK, Torto B, Jackson BT, Tao D, Ebrahimi B (2016). The non-artemisinin sesquiterpene lactones parthenin and parthenolide block *Plasmodium falciparum* sexual stage transmission. Antimicrob Agents Chemother.

[315] Tapanelli S, Chianese G, Lucantoni L, Yerbanga RS, Habluetzel A, Taglialatela-Scafati O (2016). Transmission blocking effects of neem (*Azadirachta indica*) seed kernel limonoids on *Plasmodium berghei* early sporogonic development. Fitoterapia.

[316] Jones IW, Denholm AA, Ley SV, Lovell H, Wood A, Sinden RE (1994). Sexual development of malaria parasites is inhibited in vitro by the neem extract azadirachtin, and its semi-synthetic analogues. FEMS Microbiol Lett.

[317] Carr G, Derbyshire ER, Caldera E, Currie CR, Clardy J (2012). Antibiotic and antimalarial quinones from fungus-growing ant-associated *Pseudonocardia* sp. J Nat Prod.

[318] Almeida C, Kehraus S, Prudêncio M, König GM (2011). Marilones A-C, phthalides from the sponge-derived fungus Stachylidium sp. Beilstein J Org Chem.

[319] Plouffe DM, Wree M, Du AY, Meister S, Li F, Patra K (2016). High-throughput assay and discovery of small molecules that interrupt malaria transmission. Cell Host Microbe.

[320] Peatey CL, Spicer TP, Hodder PS, Trenholme KR, Gardiner DL (2011). A high-throughput assay for the identification of drugs against late-stage *Plasmodium falciparum* gametocytes. Mol Biochem Parasitol.

