# Supplementary material for: Antiplasmodial natural products: an update
Source: Malar J. 2019 Dec 5;18:404. doi: 10.1186/s12936-019-3026-1 (PMC6896759; doi:10.1186/s12936-019-3026-1)
Supplement: Supplementary file 1 — Additional file 1. Antiplasmodial activity reported (2010–2017) for all natural products, irrespective of level of activity (no cutoff value for activity). [file 12936_2019_3026_MOESM1_ESM.docx]

**Additional file1. Antiplasmodial activity reported (2010 - 2017) for all natural products, irresepctive of level of activity (no cutoff value for activity).**

**References are given below Table.**

**Antiplasmodial Natural Products – An Update**

Nasir Tajuddeen and Fanie R. van Heerden*

School of Chemistry and Physics, University of KwaZulu-Natal, Private Bag X01, Scottsville 3209, Pietermaritzburg, South Africa. E-mail: vanheerdenf@ukzn.ac.za

**Abstract**

**Background:** Malaria remains a significant public health challenge in regions of the world where it is endemic. An unprecedented decline in malaria incidences was recorded during the last decade due to the availability of effective control interventions such as deployment of artemisinin combination therapy and insecticide-treated nets. However, according to the WHO, malaria is staging a comeback, in part due to development of drug resistance. Therefore, there is an urgent need to discover new antimalarial drugs. This article reviewed the literature on antiplasmodial compounds that were reported in the literature between 2010 and 2017.

**Methods:** Relevant literature was sourced by searching the major scientific databases including Web of Science, ScienceDirect, Scopus, SciFinder, Pubmed and Google Scholar using appropriate keyword combination.

**Results and Discussion:** A total of 1524 compounds from 397 relevant references, assayed against at least one strain of *Plasmodium* were reported in the period under review. Out of these, 39% were described as new natural products and 29% of the compounds had IC_50_ ≤ 3.0 µM against at least one strain of *Plasmodium*. Several of these compounds have the potential to be developed into viable antimalarial drugs. Also, some of these compounds could play a role in malaria eradication by targeting gametocytes.

**Keywords**: Malaria, *Plasmodium*, Antiplasmodial, Natural products, Plant metabolites, Marine natural products

2017

| s/no | Compound | Compound Class | IC_50_ | Biological source | Organism | ref |
| --- | --- | --- | --- | --- | --- | --- |
| 1 | Chrysophanol | Anthraquinone | 21.05 µg/ml (D6)  82.79 µM  36.09 µg/ml (W2)  141.95 µM | Plant | *Aloe pulcherrima* | 1 |
| 2 | aloesaponarin I | Anthraquinone | 7.80 µg/ml (D6)  21.82 µM  20.13 µg/ml (W2)  64.05 µM | Plant | *Aloe pulcherrima* | 1 |
| 3 | aloesaponarin II | Anthraquinone | 5.00 µg/ml (D6)  19.67 µM  18.6 µg/ml (W2)  73.16 µM | Plant | *Aloe pulcherrima* | 1 |
| 4 | 1,4-dihydroxy-3-methoxy powellan (New) | Crinane Alkaloid | 37 µM (Dd2) | Plant | *Amaryllis belladonna* (bulbs) | 2 |
| 5 | Distichamine | Crinane alkaloid | >50 µM (Dd2) | Plant | *Amaryllis belladonna* (bulbs) | 2 |
| 6 | 11-*O*-acetylambelline | Crinane alkaloid | 35 µM (Dd2) | Plant | *Amaryllis belladonna* (bulbs) | 2 |
| 7 | Ambelline | Crinane alkaloid | 7.3 µM (Dd2) | Plant | *Amaryllis belladonna* (bulbs) | 2 |
| 8 | Acetylcaranine | Lycorane alkaloid | 3.5 µM (Dd2) | Plant | *Amaryllis belladonna* (bulbs) | 2 |
| 9 | 9,10-dehydropellitorine (New) | Alkamide | 7.13 µg/ml (NF54)  32.23 µM | Plant | *Anacyclus pyrethrum* root | 3 |
| 10 | deca-2E,4E-dienoic acid 2-phenylethylamide | Alkamide | 5 µg/ml (NF54)  18.44 µM | Plant | *Anacyclus pyrethrum* root | 3 |
| 11 | undeca-2E,4E-dien-8,10-diynoic acid isopentylamide | Alkamide | 10.3 µg/ml (NF54)  42.36 µM | Plant | *Anacyclus pyrethrum* root | 3 |
| 12 | tetradeca-2E,4E,12Z-trien-8,10-diynoic acid isobutylamide | Alkamide | 7.19 µg/ml (NF54)  26.71 µM | Plant | *Anacyclus pyrethrum* root | 3 |
| 13 | dodeca-2E,4E-dien acid-4-hydroxy-2 phenylethylamide | Alkamide | 3.18 µg/ml (NF54)  10.09 µM | Plant | *Anacyclus pyrethrum* root | 3 |
| 14 | Mixture (1:4) of undeca-2E,4E-dien-8,10-diynoic acid  2-phenylethylamide and deca-2E,4E-dienoic acid 4-hydroxy-2-phenylethylamide | Alkamide | 7.64 µg/ml (NF54) | Plant | *Anacyclus pyrethrum* root | 3 |
| 15 | Ealapasamine A (New) | Naphthylisoquinoline | 418 nM (NF54)  452 nM (K1) | Plant | *Ancistrocladus ealaensis* (leaf) | 4 |
| 16 | Ealapasamine B (New) | Naphthylisoquinoline | 210 nM(NF54)  138 nM (K1) | Plant | *Ancistrocladus ealaensis* leaf | 4 |
| 17 | Ealapasamine C (New) | Naphthylisoquinoline | 34 nM (NF54)  6.3 nM (K1) | Plant | *Ancistrocladus ealaensis* leaf | 4 |
| 18 | Jozilebomine A (New) | Naphthylisoquinoline | 0.043 µM (NF54) | Plant | *Ancistrocladus ileboensis* (root) | 5 |
| 19 | Jozilebomine B (New) | Naphthylisoquinoline | 0.102 µM (NF54) | Plant | *Ancistrocladus ileboensis* (root) | 5 |
| 20 | Dioncophylline C2 (New) | Naphthylisoquinoline | 0.702 µM (NF54) | Plant | *Ancistrocladus*  *Ileboensis* (root bark) | 6 |
| 21 | Dioncophylline D2 (New) | Naphthylisoquinoline | 0.107 µM (NF54) | Plant | *Ancistrocladus*  *Ileboensis* (leaf) | 6 |
| 22 | Dioncophylline F (New) | Naphthylisoquinoline | 0.09 µM (NF54)  0.045 µM (K1) | Plant | *Ancistrocladus*  *Ileboensis* (root bark) | 6 |
| 23 | Ancistrocladisine A | Naphthylisoquinoline | 0.726 µM (NF54) | Plant | *Ancistrocladus*  *Ileboensis* (root bark) | 6 |
| 24 | 5'-O-methyldioncophylline D | Naphthylisoquinoline | 0.228 µM (NF54) | Plant | *Ancistrocladus*  *Ileboensis* (root bark) | 6 |
| 25 | Ancistrobrevine C | Naphthylisoquinoline | 6.51 µM (NF54) | Plant | *Ancistrocladus*  *Ileboensis* (root bark) | 6 |
| 26 | Ancistrobenomine B (5) (New) | Naphthylisoquinoline | 11.84 µM (NF54)  4.755 µM (K1) | Plant | *Ancistrocladus*  *tectorius* (stem and twigs) | 7 |
| 27 | Ancistrobenomine C (6) (New) | Naphthylisoquinoline | 6.226 µM (NF54)  5.081 µM (K1) | Plant | *Ancistrocladus*  *tectorius* (stem and twigs) | 7 |
| 28 | Ancistrocline | Naphthylisoquinoline | 8.546 µM (NF54)  2.872 µM (K1) | Plant | *Ancistrocladus*  *tectorius* (stem and twigs) | 7 |
| 29 | Woodsianone A (New) | β – triketone | 26.9 µM (3D7) | Plant | *Angophora woodsiana* (flower) | 8 |
| 30 | Woodsianone B (New) | β – triketone | 3.0 µM (3D7)  2.53 µM (Dd2) | Plant | *Angophora woodsiana* (flower) | 8 |
| 31 | Rhodomyrtosone A | β – triketone | 10.5 µM (3D7) | Plant | *Angophora woodsiana* (flower) | 8 |
| 32 | Rhodomyrtosone D | β – triketone | 14.0 µM (3D7) | Plant | *Angophora woodsiana* (flower) | 8 |
| 33 | Rhodomyrtone | β – triketone | 1.8 µM (3D7)  4.0 µM (Dd2) | Plant | *Angophora woodsiana* (flower) | 8 |
| 34 | Tomentodione A | β – triketone | 8.9 µM (3D7) | Plant | *Angophora woodsiana* (flower) | 8 |
| 35 | Tomentodione B | β – triketone | 11.3 µM (3D7) | Plant | *Angophora woodsiana* (flower) | 8 |
| 36 | 4S-Ficifolidione | β – triketone | 6.7 µM (3D7) | Plant | *Angophora woodsiana* (flower) | 8 |
| 37 | Kunzeanone A | β – triketone | 10.7 µM (3D7) | Plant | *Angophora woodsiana* (flower) | 8 |
| 38 | Watsonianone A | β – triketone | 5.3 µM (3D7)  8.8 µM (Dd2) | Plant | *Angophora woodsiana* (flower) | 8 |
| 39 | Watsonianone B | β – triketone | 0.29 µM (3D7)  0.44 µM (Dd2) | Plant | *Angophora woodsiana* (flower) | 8 |
| 40 | Tomentosone A | β – triketone | 1. µM (3D7)   1.49 µM (Dd2) | Plant | *Angophora woodsiana* (flower) | 8 |
| 41 | Irehline | steroidal alkaloid | 1.2 µM (K1) | Plant | *Holarrhena pubescens* (root) | 9 |
| 42 | Mokluangin A | steroidal alkaloid | 2.0 µM (K1) | Plant | *Holarrhena pubescens* (root) | 9 |
| 43 | Conessine (5) | steroidal alkaloid | 5.9 µM (K1) | Plant | *Holarrhena pubescens* (root) | 9 |
| 44 | Conimin (6) | steroidal alkaloid | 8.0 µM (K1) | Plant | *Holarrhena pubescens* (root) | 9 |
| 45 | Holaphyllaminol | steroidal alkaloid | 11.7 µM (K1) | Plant | *Holarrhena pubescens* (root) | 9 |
| 46 | Holaphylline (8) | steroidal alkaloid | 4.1 µM (K1) | Plant | *Holarrhena pubescens* (root) | 9 |
| 47 | Methylholaphyllamine (10) | steroidal alkaloid | 10.6 µM (K1) | Plant | *Holarrhena pubescens* (root) | 9 |
| 48 | 5-hydroxy-1H-indole-3-carboxylic acid ethyl ester | Indole alkaloid | >50 µM (3D7) | Plant | *Aristolochia cordigera* (root) | 10 |
| 49 | Hyrtiosin B | Indole alkaloid | 10 µM (3D7) | Plant | *Aristolochia cordigera* (root) | 10 |
| 50 | Hyrtiosulawesine (19) | Indole alkaloid | 2.0 µM (3D7) | Plant | *Aristolochia cordigera* (root) | 10 |
| 51 | 3,4-dihydro-hyrtiosulawes ine (New) | Indole alkaloid | 8.1 µM (3D7) | Plant | *Aristolochia cordigera* (root) | 10 |
| 52 | 6-O-(β -glucopyranosyl) hyrtiosulawesine (21) (New) | Indole alkaloid | 4.5 µM (3D7) | Plant | *Aristolochia cordigera* (root) | 10 |
| 53 | (1S,3E,7E,11R)-verticilla-3,7,12 (18)-triene | Diterpene | 9.2 µM (NF54) | Plant | *Boswellia serrate* (oleo resin) | 11 |
| 54 | Cembrene A | Diterpene | 9.9 µM (NF54) | Plant | *Boswellia serrate* (oleo resin) | 11 |
| 55 | Serratol | Diterpene | 83 µM (NF54) | Plant | *Boswellia serrate* (oleo resin) | 11 |
| 56 | 1S,3E,7R,8R,11E-7,8-epoxy-cembra-3,11-dien-1-ol | Diterpene | 32 µM (NF54) | Plant | *Boswellia serrate* (oleo resin) | 11 |
| 57 | isoincensole oxide | Diterpene | 9.6 µM (NF54) | Plant | *Boswellia serrate* (oleo resin) | 11 |
| 58 | Isodecaryiol | Diterpene | 7.5 µM (NF54) | Plant | *Boswellia serrate* (oleo resin) | 11 |
| 59 | oleanolic acid | Triterpene | 8.3 µM (NF54) | Plant | *Boswellia serrate* (oleo resin) | 11 |
| 60 | 11-keto-β-boswellic acid (10) | Triterpene | 6.6 µM (NF54) | Plant | *Boswellia serrate* (oleo resin) | 11 |
| 61 | 3-epi-neoilexonol (11) (New) | Triterpene | 7.9 µM (NF54) | Plant | *Boswellia serrate* (oleo resin) | 11 |
| 62 | β-boswellic aldehyde (13) | Triterpene | 15 µM (NF54) | Plant | *Boswellia serrate* (oleo resin) | 11 |
| 63 | Isoflindissone lactone (15) | Triterpene | 2.2 µM (NF54) | Plant | *Boswellia serrate* (oleo resin) | 11 |
| 64 | Isoflindissol lactone (16) (New) | Triterpene | 4.2 µM (NF54) | Plant | *Boswellia serrate* (oleo resin) | 11 |
| 65 | (8R,9S,20R)-tirucall-24-ene-3β,20-diol (New) | Triterpene | 18 µM (NF54) | Plant | *Boswellia serrate* (oleo resin) | 11 |
| 66 | β-bourbonene | Sesquiterpene | 108 µM (NF54) | Plant | *Boswellia serrate* (oleo resin) | 11 |
| 67 | Methyleugenol | phenyl propanoid | 32 µM (NF54) | Plant | *Boswellia serrate* (oleo resin) | 11 |
| 68 | P-methoxycinnamaldehyde | phenyl propanoid | 30 µM (NF54) | Plant | *Boswellia serrate* (oleo resin) | 11 |
| 69 | Sucupiranin A (New) | cassane diterpenoid | >30 µM (K1) | Plant | *Bowdichia virgilioides* (seed) | 12 |
| 70 | Sucupiranin G (New) | cassane diterpenoid | >27 µM (K1) | Plant | *Bowdichia virgilioides* (seed) | 12 |
| 71 | Sucupiranin H (8) (New) | cassane diterpenoid | >28.8 µM (K1) | Plant | *Bowdichia virgilioides* (seed) | 12 |
| 72 | Sucupiranin J (10) (New) | cassane diterpenoid | 32.2 µM (K1) | Plant | *Bowdichia virgilioides* (seed) | 12 |
| 73 | Sucupiranin K (New) | cassane diterpenoid | 23.5 µM (K1) | Plant | *Bowdichia virgilioides* (seed) | 12 |
| 74 | Sucupiranin L (New) | cassane diterpenoid | >27.9 µM (K1) | Plant | *Bowdichia virgilioides* (seed) | 12 |
| 75 | brujavanol C (1) (New) | Quassinoid | 25.35 µM (K1) | Plant | *Brucea javanica* (stem) | 13 |
| 76 | brujavanol D (2) (New) | Quassinoid | 30.49 µM (K1) |  | *Brucea javanica* (stem) | 13 |
| 77 | brujavanol A (3) | Quassinoid | 11.72 µM (K1) |  | *Brucea javanica* (stem) | 13 |
| 78 | Bruceine E (4) | Quassinoid | 4.37 µM (K1) |  | *Brucea javanica* (stem) | 13 |
| 79 | 5α,14β,15β –trihydroxy klaineanone | Quassinoid | 5.39 µM (K1) |  | *Brucea javanica* (stem) | 13 |
| 80 | bruceine D (6) | Quassinoid | 1.41 µM (K1) |  | *Brucea javanica* (stem) | 13 |
| 81 | Bruceine H (7) | Quassinoid | 1.06 µM (K1) |  | *Brucea javanica* (stem) | 13 |
| 82 | bruceine F (8) | Quassinoid | 3.85 µM (K1) |  | *Brucea javanica* (stem) | 13 |
| 83 | Norcaesalpin D | Norcassane diterpene | 0.98 µg/ml (3D7)  2.83 µM  1.85 µg/ml (Dd2)  5.35 µM |  | *Caesalpinia bonducella* (root) | 14 |
| 84 | Caesalsappanin R (New) | Cassane diterpene | 3.6 µM (K1) |  | *Caesalpinia sappan* (seed) | 15 |
| 85 | Caesalsappanin S (New) | Cassane diterpene | 25.1 µM (K1) |  | *Caesalpinia sappan* (seed) | 15 |
| 86 | Cipaferoids B (2) (New) | Limonoid | 9.3 µM (Dd2) |  | *Cipadessa baccifera (twigs and leaf)* | 16 |
| 87 | Cipaferoids C (3) (New) | Limonoid | 14.7 µM (Dd2) |  | *Cipadessa baccifera (twigs and leaf)* | 16 |
| 88 | Zizyberenalic acid | triterpene | 4.18 µg/ml  9.23 µM |  | *Colubrina asiatica*  (branch) | 17 |
| 89 | ergosterol peroxide | sterol | 3.08 µg/ml  7.15 µM |  | *Colubrina asiatica*  *(branch* | 17 |
| 90 | 2-Heptylquinolin-4(1H)-one | Quinolone | 1315 nM (Dd2)  1356 nM (3D7) |  | *Crinum firmifolium* (leaf) | 18 |
| 91 | 2-(6-Methylheptyl)quinolin-4(1H)-one (New) | Quinolone | 538 nM (Dd2)  501 nM (3D7) |  | *Crinum firmifolium* (leaf) | 18 |
| 92 | Nummularine-U (New) | Cyclopeptide alkaloid | 23.0 µM (K1) |  | *Ziziphus nummularia* | 19 |
| 93 | Mauritine-F (New) | Cyclopeptide alkaloid | 34.2 µM (K1) |  | *Z. nummularia* | 19 |
| 94 | Spinanine-B (New) | Cyclopeptide alkaloid | 2.1 µM (K1) |  | *Z. spina-christi* | 19 |
| 95 | Nummularine-E (New) | Cyclopeptide alkaloid | >64 µM (K1) |  | *Z. spina-christi* | 19 |
| 96 | Amphibine-D (New) | Cyclopeptide alkaloid | 8.9 µM (K1) |  | *Z. spina-christi* | 19 |
| 97 | Jubanine-F (6) (New) | Cyclopeptide alkaloid | 12.8 µM (K1) |  | *Z. jujuba* | 19 |
| 98 | Jubanine-G (7) (New) | Cyclopeptide alkaloid | 4.7 µM (K1) |  | *Z. jujuba* | 19 |
| 99 | Nummularine-B (New) | Cyclopeptide alkaloid | 3.6 µM (K1) |  | *Z. jujuba* | 19 |
| 100 | Adouetine-X (New) | Cyclopeptide alkaloid | 7.5 µM (K1) |  | *Z. jujuba* | 19 |
| 101 | Frangulanine (New) | Cyclopeptide alkaloid | 14.9 µM (K1) |  | *Hovenia dulcis* | 19 |
| 102 | Hymenocardine (New) | Cyclopeptide alkaloid | 16.4 µM (K1) |  | *Hymenocardia acida* | 19 |
| 103 | Hymenocardinol (New) | Cyclopeptide alkaloid | 17.5 µM (K1) |  | *H. acida* | 19 |
| 104 | Hymenocardine N-oxide (New) | Cyclopeptide alkaloid | 12.2 µM (K1) |  | *H. acida* | 19 |
| 105 | Hymenocardine-H (New) | Cyclopeptide alkaloid | 27.9 µM (K1) |  | *H. acida* | 19 |
| 106 | Nummularine-R (New) | Cyclopeptide alkaloid | 3.2 µM (K1) |  | *Ziziphus oxyphylla* | 19 |
| 107 | O-desmethylnummularine-R (New) | Cyclopeptide alkaloid | 7.1 µM (K1) |  | *Z. oxyphylla* | 19 |
| 108 | Hemsine-A (New) | Cyclopeptide alkaloid | 13.6 µM (K1) |  | *Z. oxyphylla* | 19 |
| 109 | Ramosine-A (New) | Cyclopeptide alkaloid | >32.0 µM (K1) |  | *Z. oxyphylla* | 19 |
| 110 | Oxyphylline-F (New) | Cyclopeptide alkaloid | 7.4 µM (K1) |  | *Z. oxyphylla* | 19 |
| 111 | Pinocembrin | flavonoid | 81% *P.berghei* suppression at 40mg/kg |  | *Dodonaea angustifolia* (leaf) | 20 |
| 112 | Santin | flavonoid | 80% *P.berghei* suppression at 50mg/kg |  | *Dodonaea angustifolia* (leaf) | 20 |
| 113 | 2*-*hydroxy-15,16-epoxyceloda-3,13(16),14-trien-18-oic acid | clerodane diterpene | 70% *P.berghei* suppression at 40mg/kg |  | *Dodonaea angustifolia* (leaf) | 20 |
| 114 | 5-(penta-1, 3-diynyl)-2-(3,4-dihydroxybut-1-ynyl)-thiophene | Acetylene thiophene | 50.2% *P.berghei* suppression at 100mg/kg |  | *Echinops hoehnelii* (root) | 21 |
| 115 | 5-(penta-1,3-diynyl)-2-(3-chloro-4-acetoxy-but-1-yn)  -thiophene | Acetylene thiophene | 32.7% *P.berghei* suppression at 100mg/kg |  | *Echinops hoehnelii (root)* | 21 |
| 116 | Embelin | Benzoquinone | 47.8% *P.berghei* suppression at 100mg/kg |  | *Embelia schimperi* (fruit) | 22 |
| 117 | seco-tiaminic acid B (New) | seco-tirucallane triterpene | 2.3 µg/ml (NF54)  5.09 µM |  | *Entandrophragma congoënse* (root) | 23 |
| 118 | seco-tiaminic acid C (New) | seco-tirucallane triterpene | 2.9 µg/ml (NF54)  6.22 µM |  | *Entandrophragma congoënse* (root) | 23 |
| 119 | 3,7,8-trihydroxyserrulat-14-en-19-oic acid | Serrulatane diterpenoid | NA at 10 µM (synthetic amide derivative 1.4 µM) |  | *Eremophila microtheca* (aerial part) | 24 |
| 120 | 3-acetoxy-7,8-dihydroxy  serrulat-14-en-19-oic acid | Serrulatane diterpenoid | NA at 10 µM |  | *Eremophila microtheca* (aerial part) | 24 |
| 121 | 3,19-diacetoxy-8-hydroxyserrulat-14-ene | Serrulatane diterpenoid | 6.89 µM (3D7)  8.76 µM (Dd2) |  | *Eremophila microtheca* (aerial part) | 24 |
| 122 | 2-(6-benzoyl-β-D-glucopyranosyloxy)-7-(1α, 2α, 6α-trihydroxy-3-oxocyclohex-4-enoyl)-5-hydroxybenzyl alcohol | Phenolic glycoside | 3.2 µM  70.82% *P.berghei* suppression at 75mg/kg  Inhibit hemozoin formation, induce oxidative stress |  | *Flacourtia indica* (aerial part) | 25 |
| 123 | Pimentelamine C (New) | indole alkaloid | 3.6 µM (3D7)  2.7 µM (Dd2) |  | *Flindersia pimenteliana* (leaf) | 26 |
| 124 | 4-methylborreverine | indole alkaloid | 0.51 µM (3D7)  0.34 µM (Dd2) |  | *Flindersia pimenteliana (leaf)* | 26 |
| 125 | Borreverine | indole alkaloid | 0.31 µM (3D7)  0.22 µM (Dd2) |  | *Flindersia pimenteliana (leaf)* | 26 |
| 126 | Dimethylisoborreverine | indole alkaloid | 0.67 µM (3D7)  0.19 µM (Dd2) |  | *Flindersia pimenteliana (leaf)* | 26 |
| 127 | Carpachromene | Flavonoid | 3.4 µM (3D7)  2.3 µM (Dd2) |  | *Flindersia pimenteliana (leaf)* | 26 |
| 128 | Bergenin | Phenolic | 8.07 µM (3D7)  >20 µM (K1)  85% *P.berghei* suppression at 100mg/kg  Inhibit heme polymerization |  | *Flueggea virosa* (leaf) | 27 |
| 129 | Geissolaevine (New) | Indole alkaloid | 74.2 µM FcB1 |  | *Geissospermum leave* (bark) | 28 |
| 130 | O-methylgeissolaevine (New) | Indole alkaloid | 56.0 µM FcB1 |  | *Geissospermum leave (bark)* | 28 |
| 131 | 3’,4’,5’,6’-tetradehydrogeissospermine (New) | Indole alkaloid | 2.9 µM FcB1 |  | *Geissospermum leave (bark)* | 28 |
| 132 | Leuconolam | Indole alkaloid | >100 µM FcB1 |  | *Geissospermum leave (bark)* | 28 |
| 133 | Geissolosimine | Indole alkaloid | 1.9 µM FcB1 |  | *Geissospermum leave (bark)* | 28 |
| 134 | Lamiridoside tri-acetate (New) | Iridoid | 16.39 µg/ml (3D7)  30.01 µM |  | *Heinsia crinite* (stem bark) | 29 |
| 135 | Lamiridosin tri-acetate (New) | Iridoid | 44.56 µg/ml (3D7)  115.40 µM |  | *Heinsia crinite (stem bark)* | 29 |
| 136 | Pectolinarigenin | Flavonoid | 41.8 µM (K1) |  | *Kickxia ramosissima* (whole plant) | 30 |
| 137 | (3E,7E)-9-Hydroxy-1-[(S)-6-hydroxy-2,8-dimethylchroman-2-yl]-  4,8-dimethylnona-3,7-dien-2-one (New) | Chromane | >112 µM (Dd2) |  | *Koeberlinia spinosa* (whole plant) | 31 |
| 138 | (3E,7Z)-9-Hydroxy-1-[(S)-6-hydroxy-2,8-dimethylchroman-2-yl]-  4,8-dimethylnona-3,7-dien-2-one (New) | Chromane | 24 µM (Dd2) |  | *Koeberlinia spinosa (whole plant* | 31 |
| 139 | (3Z,7E)-9-Hydroxy-1-[(S)-6-hydroxy-2,8-dimethylchroman-2-yl]-  4,8-dimethylnona-3,7-dien-2-one (New) | Chromane | >112 µM (Dd2) |  | *Koeberlinia spinosa (whole plant* | 31 |
| 140 | (3Z,7Z)-9-Hydroxy-1-[(S)-6-hydroxy-2,8-dimethylchroman-2-yl]-  4,8-dimethylnona-3,7-dien-2-one (New) | Chromane | >112 µM (Dd2) |  | *Koeberlinia spinosa (whole plant* | 31 |
| 141 | (3E,7E)-9-Hydroxy-1-[(S)-6-methoxy-2,8-dimethylchroman-2-yl]-  4,8-dimethylnona-3,7-dien-2-one (New) | Chromane | 24 µM (Dd2) |  | *Koeberlinia spinosa (whole plant* | 31 |
| 142 | 1-[(S or R)-2,6-Dimethyl-2,3,4,7-tetrahydrooxepin-2-yl]-3-[(S)-6-hydroxy-2,8-dimethylchroman-2-yl]propan-2-one (6) (New) | Chromane | 109 µM (Dd2) |  | *Koeberlinia spinosa (whole plant* | 31 |
| 143 | 1-[(R or S)-2,6-Dimethyl-2,3,4,7-tetrahydrooxepin-2-yl]-3-[(S)-6-hydroxy-2,8-dimethylchroman-2-yl]propan-2-one (7) (New) | Chromane | 23 µM (Dd2) |  | *Koeberlinia spinosa (whole plant* | 31 |
| 144 | (3E,7E)-9-Hydroxy-1-[(R)-6-hydroxy-2,8-dimethyl-2H-chromen-2-yl]-4,8-dimethylnona-3,7-dien-2-one (8) (New) | Chromene | >112 µM (Dd2) |  | *Koeberlinia spinosa (whole plant* | 31 |
| 145 | (R)-2-[(3E,7E)-9-Hydroxy-4,8-dimethylnona-3,7-dien-1-yl]-2,8-dimethyl-2H-chromen-6-ol (9) (New) | Chromene | >117 µM (Dd2) |  | *Koeberlinia spinosa (whole plant* | 31 |
| 146 | Fraxetin | Coumarin | 19.21 µM (NF54)  78.77% *P.berghei* suppression at 25mg/kg |  | *Lawsonia inermis* (leaf) | 32 |
| 147 | (2R,3S)-2,3,4-trihydroxy-2-  methylbutyl gallate (New) | Phenolic | 1.097 µM (D6)  0.583 µM (W2) |  | *Limonium leptophyllum* (aerial part) | 33 |
| 148 | 7-O-galloyl-D-sedoheptulose | Phenolic glycoside | 3.947 µM (D6)  3.657 µM (W2) |  | *Limonium leptophyllum* (aerial part) | 33 |
| 149 | Parthenolide | Sesquiterpee lactone | 85.18% *P.berghei* suppression at 20mg/kg  Strong intereaction with SERCA and DHFR in silico |  | *Magnolia champaca* (flower) | 34 |
| 150 | Costunolide diepoxide | Sesquiterpene lactone | 83.65% *P.berghei* suppression at 20mg/kg  Strong intereaction with SERCA and DHFR in silico |  | *Magnolia champaca (flower)* | 34 |
| 151 | 4,4´-diallyl-1,2,6,4´-tetrahydrodibenzo[*b*,*d*]furan-3´-ol (New) | Neolignan | 37.5 µM (Dd2) |  | *Magnolia grandiflora* (fruit and twig) | 35 |
| 152 | 3,3'-diallyl-4'-((4-hydroxyphenethyl)amino)-[1,1'-biphenyl]-4-ol (New) | Neolignan | 22.7 µM (Dd2) |  | *Magnolia grandiflora (fruit and twig)* | 35 |
| 153 | 4′-*O*-methyl honokiol | Neolignan | 2.8 µM (Dd2) |  | *Magnolia grandiflora (fruit and twig)* | 35 |
| 154 | Magnolol | Neolignan | 3.4 µM (Dd2) |  | *Magnolia grandiflora (fruit and twig)* | 35 |
| 155 | Honokiol | Neolignan | 16.6 µM (Dd2) |  | *Magnolia grandiflora (fruit and twig)* | 35 |
| 156 | 3-methoxymagnolol | Neolignan | 86.1 µM (Dd2) |  | *Magnolia grandiflora (fruit and twig)* | 35 |
| 157 | Isomagnolol | Neolignan | 44.4 µM (Dd2) |  | *Magnolia grandiflora (fruit and twig)* | 35 |
| 158 | malleastrumolide A (New) | Butanolide lactone | 2.74 µM (Dd2) |  | *Malleastrum sp.* (wood) | 36 |
| 159 | Mezobenthamic acid A (New) | Diterpene | >10 µg/ml (3D7)  >30.10 µM |  | *Mezoneuron benthamianum* (leaf) | 37 |
| 160 | Mezobenthamic acid B (New) | Diterpene | >10 µg/ml (3D7)  >28.56 µM |  | *Mezoneuron benthamianum* (leaf) | 37 |
| 161 | Neocaesalpin H (New) | Diterpene | >10 µg/ml (3D7)  >28.70 µM |  | *Mezoneuron benthamianum* (leaf) | 37 |
| 162 | Kaempferol | Flavonoid | >10 µg/ml (3D7)  >34.94 µM |  | *Mezoneuron benthamianum* (leaf) | 37 |
| 163 | Ethylgallate | Phenolic ester | 6.2 µg/ml (3D7)  31.29 µM |  | *Mezoneuron benthamianum* (leaf) | 37 |
| 164 | Resveratrol | Stilbenoid | >10 µg/ml (3D7)  >43.81 µM |  | *Mezoneuron benthamianum* (leaf) | 37 |
| 165 | Quercetin | Flavonoid | 9.5 µg/ml (3D7)  31.43 µM |  | *Mezoneuron benthamianum* (leaf) | 37 |
| 166 | 13b-OH-pheophorbide a | Porphyrin | 5.1 µg/ml (3D7)  8.55 µM |  | *Mezoneuron benthamianum* (leaf) | 37 |
| 167 | Gallic acid | Phenolic acid | >10 µg/ml (3D7)  >58.78 µM |  | *Mezoneuron benthamianum* (leaf) | 37 |
| 168 | β-sitosterol-Glc | Steroid glycoside | >10 µg/ml (3D7)  >17.33 µM |  | *Mezoneuron benthamianum* (leaf) | 37 |
| 169 | Epigallocatechin gallate (EGCG) | Catechin | 2.9 µM (3D7) |  | *Green tea* | 38 |
| 170 | (+)-allonorsecurinine (New) | Securinega alkaloid | 42.53 µM (3D7)  2.57 µM (W2) |  | *Phyllanthus fraternus* (whole plant) | 39 |
| 171 | *ent*-norsecurinine | Securinega alkaloid | 21.24 µM (3D7)  1.14 µM (W2) |  | *Phyllanthus fraternus (whole plant)* | 39 |
| 172 | Nirurine | Securinega alkaloid | 4.19 µM (3D7)  59.0 µM (W2) |  | *Phyllanthus fraternus (whole plant)* | 39 |
| 173 | Bubbialine | Securinega alkaloid | 6.24 µM (3D7)  27.69 µM (W2) |  | *Phyllanthus fraternus (whole plant)* | 39 |
| 174 | Epibubbialine | Securinega alkaloid | 27.23 µM (3D7)  23.67 µM (W2) |  | *Phyllanthus fraternus (whole plant)* | 39 |
| 175 | Phyllanthin | Lignan | 26.23 µM (3D7)  5.65 µM (W2) |  | *Phyllanthus fraternus (whole plant)* | 39 |
| 176 | Piplartine | 5,6-dihydropyridin-2-one alkaloid (Chalcone??) | 3.2 µg/ml (Unb169)  10.09 µM |  | *Piper tuberculatum* (root) | 40 |
| 177 | Pleiokomenines A (1) (New) | monoterpene indole alkaloid | 3.7 µM (FcB1) |  | *Pleiocarpa mutica* (stem bark) | 41 |
| 178 | Pleiokomenines B (2) (New) | monoterpene indole alkaloid | 44.5 µM (FcB1) |  | *Pleiocarpa mutica* (stem bark) | 41 |
| 179 | Poupartone A (New) | Alkyl cyclohexenone | 0.9 µM (3D7)  0.55 µM (W2)  69.5% *P.berghei* suppression at 15mg/kg |  | *Poupartia borbonica (leaf)* | 42 |
| 180 | Poupartone B (New) | Alkyl cyclohexenone | 1.81 µM (3D7)  0.97 µM (W2) |  | *Poupartia borbonica* (leaf) | 42 |
| 181 | Poupartone C (New) | Alkyl cyclohexenone | 1.13 µM (3D7)  1.27 µM (W2) |  | *Poupartia borbonica* (leaf) | 42 |
| 182 | kostchyienone A (New) | Limonoid | 1.1 µg/ml (3D7)  2.51 µM  1.23 µg/ml (Pf INDO)  2.81 µM |  | *Pseudocedrela kostchyi* (root) | 43 |
| 183 | kostchyienones B (New) | Limonoid | 0.75 µg/ml (3D7)  1.65 µM  0.78 µg/ml (Pf INDO)  1.72 µM |  | *Pseudocedrela kostchyi* (root) | 43 |
| 184 | Andirobin | Limonoid | 7.39 µg/ml (3D7)  15.78 µM  9.05 µg/ml (Pf INDO)  19.33 µM |  | *Pseudocedrela kostchyi* (root) | 43 |
| 185 | Methylangolensate | Limonoid | 5.66 µg/ml (3D7)  12.03 µM  5.62 µg/ml (Pf INDO)  11.95 µM |  | *Pseudocedrela kostchyi* (root) | 43 |
| 186 | 7-deacetylgedunin | Limonoid | 1.79 µg/ml (3D7)  4.08 µM  2.23 µg/ml (Pf INDO)  5.09 µM |  | *Pseudocedrela kostchyi* (root) | 43 |
| 187 | 7-deacetyl-7-oxogedunin | Limonoid | 2.67 µg/ml (3D7)  6.07 µM  3.29 µg/ml (Pf INDO)  7.47 µM |  | *Pseudocedrela kostchyi* (root) | 43 |
| 188 | 3,4-*seco*-lanosta-4(28),7,24-triene-3,21-dioic acid | *seco*-lanostane triterpenoid | 10.05 µg/ml (3D7)  20.73 µM  >20 µg/ml (Pf INDO)  >41.26 µM |  | *Pseudocedrela kostchyi* (root) | 43 |
| 189 | β-sitosterol | Sterol | 8.05 µg/ml (3D7)  19.48 µM  9.05 µg/ml (Pf INDO)  21.82 µM |  | *Pseudocedrela kostchyi* (root) | 43 |
| 190 | Stigmasterol | Sterol | 10.05 µg/ml (3D7)  24.35 µM  8.05 µg/ml (Pf INDO)  19.51 µM |  | *Pseudocedrela kostchyi* (root) | 43 |
| 191 | betulinic acid | Lupane triterpene | 19.05 µg/ml (3D7)  41.71 µM  10.05 µg/ml (Pf INDO)  22.01 µM |  | *Pseudocedrela kostchyi* (root) | 43 |
| 192 | 3-O-β-D-glucopyranosyl β-sitosterol | Sterol glucoside | 19.05 µg/ml (3D7)  33.02 µM  >20 µg/ml (Pf INDO)  >34.67 µM |  | *Pseudocedrela kostchyi* (root) | 43 |
| 193 | 3-O-β-D-glucopyranosyl stigmasterol | Sterol glycoside | 18.05 µg/ml (3D7)  31.40 µM  >20 µg/ml (Pf INDO)  >34.79 µM |  | *Pseudocedrela kostchyi* (root) | 43 |
| 194 | 3,6,8-trihydroxy-2-(3,4-dihydroxylphenyl)-4H-chrom-4-one | Flavonoid | 3.11 µg/ml (3D7)  10.29 µM  4.61 µg/ml (Pf INDO)  15.25 µM |  | *Pseudocedrela kostchyi* (root) | 43 |
| 195 | quercetin, 3,4′,7-trimethyl ether | Flavonoid | 2.67 µg/ml (3D7)  7.75 µM  3.29 µg/ml (Pf INDO)  9.56 µM |  | *Pseudocedrela kostchyi* (root) | 43 |
| 196 | Spilanthol | N-alkylamide | 23.22 µg/ml (3D7)  104.99 µM |  | *Spilanthes paniculata* (flower head) | 44 |
| 197 | (2E,4Z)-N-isobutyl-2,4-undecadiene-8,10-diynamide | N-alkylamide | 14.64 µg/ml (3D7)  63.86 µM |  | *Spilanthes paniculata* (flower head) | 44 |
| 198 | Stephanine | Quinoline alkaloid | 0.69 µM (3D7)  1.32 µM (W2) |  | *Stephania venosa* (tuber) | 45 |
| 199 | Crebanine | Quinoline alkaloid | 1.56 µM (3D7)  2.16 µM (W2) |  | *Stephania venosa* (tuber) | 45 |
| 200 | O-methylbulbocapnine | Quinoline alkaloid | 2.81 µM (3D7)  5.71 µM (W2) |  | *Stephania venosa* (tuber) | 45 |
| 201 | Aequichalcone A (New) | Chalcone | 9.20 µM (3D7) |  | *Tephrosia aequilata* (root) | 46 |
| 202 | Aequichalcone B (New) | Chalcone | 9.75 µM (3D7) |  | *Tephrosia aequilata* (root) | 46 |
| 203 | Aequichalcone C (New) | Chalcone | 2.48 µM (3D7) |  | *Tephrosia aequilata* (root) | 46 |
| 204 | 3,4:8,9-Dimethylenedioxypterocarpene (New) | Pterocarpan | >40 µM (3D7) |  | *Tephrosia aequilata* (root) | 46 |
| 205 | Obovatachalcone | Chalcone | 4.23 µM (3D7) |  | *Tephrosia aequilata* (root) | 46 |
| 206 | Praecansone B | Chalcone | 4.14 µM (3D7) |  | *Tephrosia aequilata* (root) | 46 |
| 207 | Praecansone A | Chalcone | 6.45 µM (3D7) |  | *Tephrosia aequilata* (root) | 46 |
| 208 | Isopongaflavone | Flavonoid | 8.19 µM (3D7) |  | *Tephrosia aequilata* (root) | 46 |
| 209 | (E)-5-Hydroxytephrostachin (New) | Flavonoid | 1.7 µM (D6) |  | *Tephrosia purpurea subsp. Leptostachya* (stem) | 47 |
| 210 | Terpurlepflavone (New) | Flavonoid | 14.8 µM (D6) |  | *Tephrosia purpurea subsp. Leptostachya* (stem) | 47 |
| 211 | Tachrosin | Flavonoid | 27.1 µM (D6) |  | *Tephrosia purpurea subsp. Leptostachya* (stem) | 47 |
| 212 | Subtriflavanonol (New) | Flavonoid | 12.5 µM (D6)  24.2 µM (3D7) |  | *Tephrosia subtriflora* (aerial parts) | 48 |
| 213 | 3-hydroxy-di-(2,2-dimethylchromene-5:6,7:8)flavanonol | Flavonoid | 4.6 µM (D6)  1.7 µM (3D7) |  | *Tephrosia subtriflora* (aerial parts) | 48 |
| 214 | Spinosaflavanone B | Flavonoid | 5.9 µM (D6)  5.5 µM (3D7) |  | *Tephrosia subtriflora* (aerial parts) | 48 |
| 215 | Mundulinol | Flavonoid | 35.6 µM (D6)  27.8 µM (3D7) |  | *Tephrosia subtriflora* (aerial parts) | 48 |
| 216 | Trichospirolide A (New) | Sesquiterpene lactone | 1.49 µM (Dd2) |  | *Trichospira verticillata* (whole plant) | 49 |
| 217 | Trichospirolide B (New) | Sesquiterpene lactone | 37.05 µM (Dd2) |  | *Trichospira verticillata* (whole plant) | 49 |
| 218 | Trichospirolide C (New) | Sesquiterpene lactone | 12.10 µM (Dd2) |  | *Trichospira verticillata* (whole plant) | 49 |
| 219 | Trichospirolide D (New) | Sesquiterpene lactone | 44.33 µM (Dd2) |  | *Trichospira verticillata* (whole plant) | 49 |
| 220 | Tripteryol A (New) | Flavonoid | 3.15 µg/ml (D6)  6.99 µM  3.35 µg/ml (W2)  7.44 µM |  | *Tripterygium wilfordii* (stem and root) | 50 |
| 221 | Tripteryol B (New) | Flavonoid | 4.63 µg/ml (D6)  10.57 µM  4.18 µg/ml (W2)  9.54 µM |  | *Tripterygium wilfordii* (stem and root) | 50 |
| 222 | Cherrevenaphthalene A (New) | Phenylnaphthalene | 21.1 µM (TM4/8.2)  24.2 µM (K1CB1) |  | *Uvaria cherrevensis* (stem and root) | 51 |
| 223 | Cherrevenaphthalene B (New) | Phenylnaphthalene | 18.8 µM (TM4/8.2)  23.4 µM (K1CB1) |  | *Uvaria cherrevensis* (stem and root) | 51 |
| 224 | Cherrevenaphthalene C (New) | Phenylnaphthalene | 40.4 µM (TM4/8.2) |  | *Uvaria cherrevensis* (stem and root) | 51 |
| 225 | 2-hydroxy-3-methoxy-6-(4 -hydroxyphenyl)naphthalene | Phenylnaphthalene | 22.3 µM (TM4/8.2)  23.4 µM (K1CB1) |  | *Uvaria cherrevensis* (stem and root) | 51 |
| 226 | 2’,4’-dihydroxy-3’-(2-hydroxy  benzyl)-6’-methoxychalcone | Chalcone | 32.4 µM (TM4/8.2) |  | *Uvaria cherrevensis* (stem and root) | 51 |
| 227 | 8'',9''-dihydrowelwitschin H (New) | Chalcone | 3.10 µg/ml (K1)  5.2 µM |  | *Uvaria siamensis* (root) | 52 |
| 228 | Uvarin B (New) | Chalcone | 3.02 µg/ml (K1)  4.93 µM |  | *Uvaria siamensis* (root) | 52 |
| 229 | Uvarin C (New) | Chalcone | 3.09 µg/ml (K1)  5.04 µM |  | *Uvaria siamensis* (root) | 52 |
| 230 | Dependensin | Chalcone | 4.21 µg/ml (K1)  7.06 µM |  | *Uvaria siamensis* (root) | 52 |
| 231 | Welwitschin E | Chalcone | 3.99 µg/ml (K1)  6.56 µM |  | *Uvaria siamensis* (root) | 52 |
| 232 | 8-(4’-hydroxymethacrylate)-dehydromelitensin | Sesquiterpene lactone | 2.96 µg/ml (3D7)  8.50 µM |  | *Vernonia fimbrillifera* (leaf) | 53 |
| 233 | Onopordopicrin | Sesquiterpene lactone | 3.37 µg/ml (3D7)  9.67 µM |  | *Vernonia fimbrillifera* (leaf) | 53 |
| 234 | 8α-[4’-hydroxymethacryloyl  oxy]-4-epi-sonchucarpolide | Sesquiterpene lactone | 3.27 µg/ml (3D7)  9.02 µM |  | *Vernonia fimbrillifera* (leaf) | 53 |
| 235 | Sesamine | Lignan | 1.92 µg/ml (W2)  5.42 µM  3.23 µg/ml (D6)  9.12 µM |  | *Zanthoxylum gilletii* (stem bark) | 54 |
| 236 | Fagaramide | Alkamide | 15.15 µg/ml (W2)  61.26 µM  7.73 µg/ml (D6)  31.26 µM |  | *Zanthoxylum gilletii (stem bark)* | 54 |
| 237 | 8-acetonyldihydrochelerythrine | Benzophenanthridine alkaloid | 4.02 µg/ml (W2)  9.92 µM  4.06 µg/ml (D6)  10.01 µM |  | *Zanthoxylum gilletii (stem bark)* | 54 |
| 238 | Syncarpamide | norepinephrine alkaloid | 3.9 µM (3D7)  2.56 µM (K1)  2.04 µM (D6)  3.06 µM (W2) |  | *Zanthoxylum*  *Syncarpum* (leaf) | 55 |
| 239 | Astraeusin M (New) | lanostane triterpenoids | 3.0 µg/ml (K1)  5.99 µM | Mushroom | *Astraeus asiaticus* | 56 |
| 240 | Astraeusin N (New) | lanostane triterpenoids | >10 µg/ml (K1)  >22.6 µM | Mushroom | *Astraeus asiaticus* | 56 |
| 241 | Astraeusin O (New) | lanostane triterpenoids | >10 µg/ml (K1)  >21.17 µM | Mushroom | *Astraeus asiaticus* | 56 |
| 242 | Astraeusin P (New) | lanostane triterpenoids | >10 µg/ml (K1)  >21.91 µM | Mushroom | *Astraeus asiaticus* | 56 |
| 243 | Astraeusin Q (New) | lanostane triterpenoids | >10 µg/ml (K1)  >20.07 µM | Mushroom | *Astraeus asiaticus* | 56 |
| 244 | 26-epi-Artabotryol C1 (New) | lanostane triterpenoids | >10 µg/ml (K1)  >21.17 µM | Mushroom | *Astraeus asiaticus* | 56 |
| 245 | 26-epi-Astrasiaone (New) | lanostane triterpenoids | >10 µg/ml (K1)  >21.26 µM | Mushroom | *Astraeus asiaticus* | 56 |
| 246 | epi-Inotodiol | lanostane triterpenoids | >10 µg/ml (K1)  >22.59 µM | Mushroom | *Astraeus asiaticus* | 56 |
| 247 | Astraodoric acid C | lanostane triterpenoids | >10 µg/ml (K1)  >21.16 µM | Mushroom | *Astraeus asiaticus* | 56 |
| 248 | Artabotryol C1 | lanostane triterpenoids | >10 µg/ml (K1)  >21.15 µM | Mushroom | *Astraeus asiaticus* | 56 |
| 249 | Astrasiaone | lanostane triterpenoids | >10 µg/ml (K1)  >21.24 µM | Mushroom | *Astraeus asiaticus* | 56 |
| 250 | Artabotryol D | lanostane triterpenoids | >10 µg/ml (K1)  >21.79 µM | Mushroom | *Astraeus asiaticus* |  |
| 251 | Astrasiate | lanostane triterpenoids | >10 µg/ml (K1)  >20.46 µM | Mushroom | *Astraeus asiaticus* | 56 |
| 252 | Bifurcatriol (New) | Diterpene | 0.65 µg/ml (K1)  2.00 µM | Brown algae | *Bifurcaria bifurcata* | 57 |
| 253 | Cochliomycin A | Resorcylic acid lactones (RALs) | 30.7 µM  1.84 µM for synthetically modified derivative | Fungi | *Cochliobolus lunatus* (M351) | 58 |
| 254 | LL-Z1640-2 | Resorcylic acid lactones (RALs) | 14.9 µM | Fungi | *Cochliobolus lunatus* (TA26-46) | 58 |
| 255 | (7’E)-6’-oxozeaenol | Resorcylic acid lactones (RALs) | 11.0 µM | Fungi | *Cochliobolus lunatus* (TA26-46) | 58 |
| 256 | Hitoyol B (New) | norsesquiterpenoid | 59 µM (3D7) | Basidiomycete Fungi | *Coprinopsis cinerea* (NBRC 100011) | 59 |
| 257 | Epoxycytochalasin H | Cytochalasin | 51.7 ng/ml (D6)  0.1 µM  39.4 ng/ml (W2)  0.08 µM | Endophytic fungi | *Diaporthe miriciae* UFMGCB 9720 | 60 |
| 258 | Fusaripeptide A (New) | Cyclodepsipeptide | 0.34 µM (D6) | Endophytic fungi | *Fusarium sp.* | 61 |
| 259 | Incarnatin A (New) | Sesquiterpene | 9.8 µg/ml (K1)  39.50 µM | Mushroom | *Gloeostereum incarnatum* BCC41461 | 62 |
| 260 | Incarnatin B (New) | Sesquiterpene | 3.93 µg/ml (K1)  14.99 µM | Mushroom | *Gloeostereum incarnatum* BCC41461 | 62 |
| 261 | Incarnatin C (New) | Sesquiterpene | >10 µg/ml (K1)  >35.95 µM | Mushroom | *Gloeostereum incarnatum* BCC41461 | 62 |
| 262 | Incarnolactone A (New) | Sesquiterpene | >10 µg/ml (K1)  >40.63 µM | Mushroom | *Gloeostereum incarnatum* BCC41461 | 62 |
| 263 | Incarnolactone C (New) | Sesquiterpene | >10 µg/ml (K1)  >38.15 µM | Mushroom | *Gloeostereum incarnatum* BCC41461 | 62 |
| 264 | Incarnate methyl ester (New) | Sesquiterpene | >10 µg/ml (K1)  >38.15 µM | Mushroom | *Gloeostereum incarnatum* BCC41461 | 62 |
| 265 | Chondrosterin B | Sesquiterpene | 3.1 µg/ml (K1)  12.59 µM | Mushroom | *Gloeostereum incarnatum* BCC41461 | 62 |
| 266 | (E)-dictyochromenol | Benzopyran | >10 µg/ml (K1)  >32.00 µM | Mushroom | *Gloeostereum incarnatum* BCC41461 | 62 |
| 267 | Ircinin-1 | Furanosesterterpenoid | 58 µM | marine sponge | *Ircinia oros* | 63 |
| 268 | Ircinin-2 | Furanosesterterpenoid | 56 µM | marine sponge | *Ircinia oros* | 63 |
| 269 | Ircinialactam E (New) | Furanosesterterpenoid | 95 µM | marine sponge | *Ircinia oros* | 63 |
| 270 | Ircinialactam F (New) | Furanosesterterpenoid | >100 µM | marine sponge | *Ircinia oros* | 63 |
| 271 | Unguiculin A (New) | Guanidine alkaloid | 12.89 µM (3D7) | marine sponge | *Monanchora unguiculata* | 64 |
| 272 | Ptilomycalin E (New) | Guanidine alkaloid | 0.35 µM (3D7) | marine sponge | *Monanchora unguiculata* | 64 |
| 273 | Ptilomycalins F (New) | Guanidine alkaloid | 0.23 µM (3D7) | marine sponge | *Monanchora unguiculata* | 64 |
| 274 | Ptilomycalins G (New) + H | Guanidine alkaloid | 0.46 µM (3D7) | marine sponge | *Monanchora unguiculata* | 64 |
| 275 | Crambescidin 800 | Guanidine alkaloid | 0.52 µM (3D7) | marine sponge | *Monanchora unguiculata* | 64 |
| 276 | Fromiamycalin | Guanidine alkaloid | 0.24 µM (3D7) | marine sponge | *Monanchora unguiculata* | 64 |
| 277 | Nigrosporone A (New) | Anthraquinone | >33.97 µM (K1) | Endophytic fungus | *Nigrospora sp.* BCC 47789 | 65 |
| 278 | Nigrosporone B (New) | Anthraquinone | 10.81 µM (K1) | Endophytic fungus | *Nigrospora sp.* BCC 47789 | 65 |
| 279 | Fusaquinon A | Anthraquinone | >32.67 µM (K1) | Endophytic fungus | *Nigrospora sp.* BCC 47789 | 65 |
| 280 | Bastimolide A | Macrolide | 2.6 µM (HB3) | Cyanobacterium | *Okeania hirsuta* (PAB-19MAY11-4) | 66 |
| 281 | Bastimolide B (New) | Macrolide | 5.7 µM (HB3) | Cyanobacterium | *Okeania hirsuta* (PAB-19MAY11-4) | 66 |
| 282 | Pustulosaisonitrile-1 (New) | Isocyano diterpene | 1.08 µM (3D7)  1.54 µM (Dd2) | Nudibranch | *Phyllidiella pustulosa* | 67 |
| 283 | Wakodecaline A (New) |  | 28 µg/ml (3D7)  62.6 µM | Fungus | *Pyrenochaetopsis sp.* RK10-F058 | 68 |
| 284 | Wakodecaline B (New) |  | 16 µg/ml (3D7)  35.94 µM | Fungus | *Pyrenochaetopsis sp.* RK10-F058 | 68 |
| 285 | Phomasetin | Pyrrolidin-2,4-dione alkaloid  3-(Decalinoyltetramic acids) | 0.74 µg/ml (3D7)  1.8 µM | Fungus | *Pyrenochaetopsis sp.* RK10-F058 | 68 |
| 286 | Nocardamine | Cyclic peptide | 3.2 µg/ml (K1)  5.32 µM | Actinomycete bacteria | *Streptomyces sp.* BCC71188 | 69 |
| 287 | Dehydroxynocardamine | Cyclic peptide | 2.63 µg/ml (K1)  4.5 µM | Actinomycete bacteria | *Streptomyces sp.* BCC71188 | 69 |
| 288 | Monoglycosylelaiolide | Macrolide | 2.46 µg/ml (K1)  2.75 µM | Actinomycete bacteria | *Streptomyces sp.* BCC71188 | 69 |
| 289 | Azalomycin | Macrolide | 0.22 µg/ml (K1)  0.21 µM | Actinomycete bacteria | *Streptomyces sp.* BCC71188 | 69 |
| 290 | 11,11′- O-dimethylelaiophylin | Macrolide | 1.47 µg/ml (K1)  1.4 µM | Actinomycete bacteria | *Streptomyces sp.* BCC71188 | 69 |
| 291 | 2-Amino-6-hydroxyl-7-methyl-1,4-naphthoquinone (New) | Naphthoquinone | >10 µg/ml (K1)  >49.22 µM | Actinomycete bacteria | *Streptomyces sp.* BCC71188 | 69 |
| 292 | Nahuoic acid C | Decalin polyketide | >10 µg/ml (K1)  >19.74 µM | Actinomycete bacteria | *Streptomyces sp.* BCC71188 | 69 |
| 293 | Geldanamycin | Macrocyclic polyketide | 0.35 µg/ml (K1)  0.62 µM | Actinomycete bacteria | *Streptomyces sp.* BCC71188 | 69 |
| 294 | 17-O-demethylgeldanamycin | Macrocyclic polyketide | 1.9 µg/ml (K1)  3.48 µM | Actinomycete bacteria | *Streptomyces sp.* BCC71188 | 69 |
| 295 | 17-Demethoxyreblastatin | Macrocyclic polyketide | 0.31 µg/ml (K1)  0.6 µM | Actinomycete bacteria | *Streptomyces sp.* BCC71188 | 69 |
| 296 | 4,5-Dihydrothiazinogelda  Namycin | Macrocyclic polyketide | >10 µg/ml (K1)  >15.73 µM | Actinomycete bacteria | *Streptomyces sp.* BCC71188 | 69 |
| 297 | Cyclooctatin | Diterpene | 7.14 µg/ml (K1)  22.14 µM | Actinomycete bacteria | *Streptomyces sp.* BCC71188 | 69 |
| 298 | Germicidin B | Pyranone | >10 µg/ml (K1)  >54.88 µM | Actinomycete bacteria | *Streptomyces sp.* BCC71188 | 69 |
| 299 | Germicidin A | Pyranone | >10 µg/ml (K1)  >50.96 µM | Actinomycete bacteria | *Streptomyces sp.* BCC71188 | 69 |
| 300 | Octaminomycin A (New) | Cyclodepsipeptide | 1.5 µM (3D7)  1.6 µM (Dd2)  1.3 µM (K1) | Actinomycete bacteria | *Streptomyces sp.* RK85-270 | 70 |
| 301 | Octaminomycin B (New) | Cyclodepsipeptide | 1.5 µM (3D7)  1.1 µM (Dd2)  0.83 µM (K1) | Actinomycete bacteria | *Streptomyces sp.* RK85-270 | 70 |
| 302 | Gancidin W | Diketopiperazine | 78.46% inhibition of P.berghei parasite at 3.125 µg/kg | Actinomycete bacteria | *Streptomyces* SUK10 | 71 |
| 303 | Carmaphycin B | Peptide | 4.1 nM(Dd2)  Plasmodium proteasome inhibitor | Cyanobacterium | *Symploca sp.* | 72 |
| 304 | Swinholide A | Macrolide | 4.75 µg/ml  3.42 µM | Marine sponge | *Theonella aff. swinhoei* | 73 |
| 305 | Spirombandakamine A1(New) | Naphthylisoquinoline | 7.0 nM (K1)  40 nM (NF54) | Plant | *Ancistrocladus sp.* (leaf) | 74 |
| 306 | Spirombandakamine A2 (New) | Naphthylisoquinoline | 94.0 nM (K1)  226 nM (NF54) | Plant | *Ancistrocladus sp.* (leaf) | 74 |
| 307 | Mbandakamine B2 (New) | Naphthylisoquinoline | 4.0 nM  170 nM (NF54) | Plant | *Ancistrocladus sp.* (leaf) | 74 |
| 308 | Halymeniaol (New) | Sterol | 3.0 µM | Alga | *Halymenia floresii* | 75 |
| 309 | Dragmacidin G (New) | Indole alkaloid | 6.4 µM | Sponge | *Spongosorite sp.* | 76 |

2016

| 1 | Harpagide | Iridoid glycoside | >50 µg/ml  >137.23 µM | Plant | *Ajuga laxmannii* (aerial parts) | 77 |
| --- | --- | --- | --- | --- | --- | --- |
| 2 | 8-O-acetylharpagide | Iridoid glycoside | >50 µg/ml  >123.03 µM | Plant | *Ajuga laxmannii* (aerial parts) | 77 |
| 3 | Cis-melilotoside | Phenyl ethanoid glycoside | >50 µg/ml  >153.23 µM | Plant | *Ajuga laxmannii* (aerial parts) | 77 |
| 4 | Trans-melilotoside | Phenyl ethanoid glycoside | 48.7 µg/ml  149.24 µM | Plant | *Ajuga laxmannii* (aerial parts) | 77 |
| 5 | Dihydromelilotoside | Phenyl ethanoid glycoside | >50 µg/ml  >152.29 µM | Plant | *Ajuga laxmannii* (aerial parts) | 77 |
| 6 | Verbascoside | Phenyl ethanoid glycoside | >50 µg/ml  >80.05 µM | Plant | *Ajuga laxmannii* (aerial parts) | 77 |
| 7 | Galactosylmartynoside | Phenyl ethanoid glycoside | >50 µg/ml  >61.36 µM | Plant | *Ajuga laxmannii* (aerial parts) | 77 |
| 8 | Isoorientin | Flavonoid glycoside | 9.7 µg/ml  19.86 µM | Plant | *Ajuga laxmannii* (aerial parts) | 77 |
| 9 | ent-3-*α-*hydroxy-kaur-16-en-18-ol | Kaurane diterpene | 3.5 µM (NF54) |  | *Aldama discolour* (leaf) | 78 |
| 10 | ent-7-oxo-pimara-8,15-diene-18-ol (New) | Primarane diterpene | 3.8 µM (NF54) |  | *Aldama discolour* (leaf) | 78 |
| 11 | ent-2S,4S-2-19-epoxy-pimara-8(3),15-diene-7 β-ol (New) | Primarane diterpene | 16.5 µM (NF54) |  | *Aldama discolour* (leaf) | 78 |
| 12 | ent-7-oxo-pimara-8,15-diene-3β-ol (New) | Primarane diterpene | 16.1 µM (NF54) |  | *Aldama discolour* (leaf) | 78 |
| 13 | Nataloin | Anthrone | 40.4% *P.berghei* suppression at 200mg/kg |  | *Aloe pulcherrima* (leaf latex) | 79 |
| 14 | 7-hydroxyaloin | Anthrone | 56.2% *P.berghei* suppression at 15mg/kg |  | *Aloe pulcherrima* (leaf latex) | 79 |
| 15 | Aphadilactone E (New) | Diterpenoid | 1.03 µM (Dd2) |  | *Aphanamixis grandifolia* (leaf) | 80 |
| 16 | Aphadilactone F (New) | Diterpenoid | 2.86 µM (Dd2) |  | *Aphanamixis grandifolia* (leaf) | 80 |
| 17 | Aphadilactone G (New) | Diterpenoid | 20 µM (Dd2) |  | *Aphanamixis grandifolia* (leaf) | 80 |
| 18 | Aphadilactone H (New) | Diterpenoid | 1.6 µM (Dd2) |  | *Aphanamixis grandifolia* (leaf) | 80 |
| 19 | Aphadilactone I (New) | Diterpenoid | 2.11 µM (Dd2) |  | *Aphanamixis grandifolia* (leaf) | 80 |
| 20 | Brujavanol A (New) | Quassinoid | 4.48 µg/ml (K1)  11.72 µM |  | *Brucea javanica* (root) | 81 |
| 21 | Brujavanol B (New) | Quassinoid | 5.31 µg/ml (K1)  14.50 µM |  | *Brucea javanica* (root) | 81 |
| 22 | Bruceine D | Quassinoid | 0.58 µg/ml (K1)  1.41 µM |  | *Brucea javanica* (root) | 81 |
| 23 | 11-dehydroklaineanone | Quassinoid | 5.26 µg/ml (K1)  14.59 µM |  | *Brucea javanica* (root) | 81 |
| 24 | 15β-hydroxyklaineanone | Quassinoid | 5.34 µg/ml (K1)  14.1 µM |  | *Brucea javanica* (root) | 81 |
| 25 | 14,15β-dihydroxyklaineanone | Quassinoid | 5.02 µg/ml (K1)  12.72 µM |  | *Brucea javanica* (root) | 81 |
| 26 | 15β-*O*-acetyl-14-hydroxyklaineanone | Quassinoid | 13.71 µg/ml (K1)  31.40 µM |  | *Brucea javanica* (root) | 81 |
| 27 | Betulonic acid | Lupane triterpene | 2.0 µg/ml (NF54)  4.40 µM |  | *Buddleja saligna* (leaf) | 82 |
| 28 | Betulone | Lupane triterpene | 3.6 µg/ml (NF54)  8.17 µM |  | *Buddleja saligna (leaf)* | 82 |
| 29 | Spinasterol | Sterol | 14 µg/ml (NF54)  33.95 µM |  | *Buddleja saligna (leaf)* | 82 |
| 30 | 23-O-(Z)-p-Coumaroyl-23-hydroxybetulin | Lupane triterpene | 0.8 µM (HB3)  1.53µM (NHP1337) |  | *Buxus sempervirens* | 83 |
| 31 | 23-O-(E)-p-Coumaroyl-23-hydroxybetulin | Lupane triterpene | 0.9 µM (HB3)  0.85 µM (NHP1337) |  | *Buxus sempervirens* | 83 |
| 32 | 23-O-(trans)-Feruloyl-23-hydroxybetulin (New) | Lupane triterpene | 0.5 µM (HB3)  0.27 µM (NHP1337) |  | *Buxus sempervirens* | 83 |
| 33 | 23-O-(cis)-Feruloyl-23-hydroxy  betulin and 23-O-(trans)-feruloyl-  23-hydroxybetulin (3:1 mixture) (New) | Lupane triterpene | 1.0 µM (HB3)  0.31 µM (NHP1337) |  | *Buxus sempervirens* | 83 |
| 34 | 3-O-(cis)-p-Coumaroyl-23-hydroxybetulin and 3-O-(E)-p-coumaroyl-23-hydroxybetulin  (4:1 mixture)(New) | Lupane triterpene | 2.3 µM (HB3)  1.6 µM (NHP1337) |  | *Buxus sempervirens* | 83 |
| 35 | 3-O-(E)-p-Coumaroyl-23-hydroxybetulin | Lupane triterpene | 1.9 µM (HB3)  1.4 µM (NHP1337) |  | *Buxus sempervirens* | 83 |
| 36 | 3-O-(trans)-Feruloyl-23-hydroxybetulin and 3-O-(cis)-feruloyl-23-hydroxybetulin  (2:1 mixture)(New) | Lupane triterpene | 3.0 µM (HB3)  2.3 µM (NHP1337) |  | *Buxus sempervirens* | 83 |
| 37 | 23-Hydroxybetulin | Lupane triterpene | 3.4 µM (HB3)  5.2 µM (NHP1337) |  | *Buxus sempervirens* | 83 |
| 38 | kaempferol 3-O-α-L- (2’’,3’’-di-E-p-coumaroyl)rhamnoside | Flavonoid glycoside | 0.6 µM (HB3)  7.0 µM (NHP1337) |  | *Platanus occidentalis* | 83 |
| 39 | kaempferol 3-O-α-L-(2’’-E-p-coumaroyl-3’’-Z-p-coumaroyl)  rhamnoside | Flavonoid glycoside | 2.0 µM (HB3)  4.0 µM (NHP1337) |  | *Platanus occidentalis* | 83 |
| 40 | kaempferol 3-O-α-L-(2’’-Z-p-coumaroyl-3’’-E-p-coumaroyl)-  rhamnoside | Flavonoid glycoside | 0.5 µM (HB3)  4.1 µM (NHP1337) |  | *Platanus occidentalis* | 83 |
| 41 | kaempferol 3-O-α-L-(2’’,3’’-di-Z-p-coumaroyl) rhamnoside | Flavonoid glycoside | 1.8 µM (HB3)  7 µM (NHP1337) |  | *Platanus occidentalis* | 83 |
| 42 | kaempferol-3-O-(3’’,’’-diacetyl-2’’,6’’-di-E-p-coumaroyl)-glucoside | Flavonoid glycoside | 0.6 µM (HB3)  2.1 µM (NHP1337) |  | *Quercus laceyi* | 83 |
| 43 | kaempferol 3-O-(2’’-cis-p-coumaroyl-3’’,4’’-diacetyl-6’’-trans-p-coumaroyl)-β-D-glucopyranoside | Flavonoid glycoside | 0.9 µM (HB3)  5 µM (NHP1337) |  | *Quercus laceyi* | 83 |
| 44 | kaempferol-3-O-(2’’-trans-p-coumaroyl-3’’, 4’’-diacetyl-6’’-cis-pcoumaroyl)-β-D-glucopyranoside | Flavonoid glycoside | 0.8 µM (HB3)  4.0 µM (NHP1337) |  | *Quercus laceyi* | 83 |
| 45 | kaempferol-3-O-(3’’,4’’-diacetyl-2’’,6’’-di-Z-p-coumaroyl)glucoside | Flavonoid glycoside | 2.1 µM (HB3)  3.8 µM (NHP1337) |  | *Quercus laceyi* | 83 |
| 46 | Knipholone | Anthraquinone | 4.9 µM (HB3)  5 µM (NHP1337) |  | *Bulbine frutescens* | 83 |
| 47 | Amentoflavone | Flavonoid | 25 µM (HB3)  19 µM (NHP1337) |  | *Rhus virens* | 83 |
| 48 | Fortunilide A (New) | Sesquiterpenoid | 5.2 nM (Dd2) |  | *Chloranthus fortunei* | 84 |
| 49 | Fortunilide B (New) | Sesquiterpenoid | 19 nM (Dd2) |  | *Chloranthus fortunei* | 84 |
| 50 | Fortunilide C (New) | Sesquiterpenoid | 211 nM (Dd2) |  | *Chloranthus fortunei* | 84 |
| 51 | Fortunilide D (New) | Sesquiterpenoid | 30 nM (Dd2) |  | *Chloranthus fortunei* | 84 |
| 52 | FortunilideE (New) | Sesquiterpenoid | 43 nM (Dd2) |  | *Chloranthus fortunei* | 84 |
| 53 | Fortunilide F (New) | Sesquiterpenoid | 5300 nM (Dd2) |  | *Chloranthus fortunei* | 84 |
| 54 | Fortunilide G (New) | Sesquiterpenoid | 46 nM (Dd2) |  | *Chloranthus fortunei* | 84 |
| 55 | Fortunilide H (New) | Sesquiterpenoid | 198 nM (Dd2) |  | *Chloranthus fortunei* | 84 |
| 56 | Fortunilide I (New) | Sesquiterpenoid | 94 nM (Dd2) |  | *Chloranthus fortunei* | 84 |
| 57 | Fortunilide J (New) | Sesquiterpenoid | 9900 nM (Dd2) |  | *Chloranthus fortunei* | 84 |
| 58 | Fortunilide K (New) | Sesquiterpenoid | 4700 nM (Dd2) |  | *Chloranthus fortunei* | 84 |
| 59 | Fortunilide L (New) | Sesquiterpenoid | 99 nM (Dd2) |  | *Chloranthus fortunei* | 84 |
| 60 | Sarglabolide I | Sesquiterpenoid | 4600 nM (Dd2) |  | *Chloranthus fortunei* | 84 |
| 61 | Sarglabolide J | Sesquiterpenoid | 7.2 nM (Dd2) |  | *Chloranthus fortunei* | 84 |
| 62 | Shizukaol K | Sesquiterpenoid | 860 nM (Dd2) |  | *Chloranthus fortunei* | 84 |
| 63 | Shizukaol I | Sesquiterpenoid | 111 nM (Dd2) |  | *Chloranthus fortunei* | 84 |
| 64 | Shizukaol C | Sesquiterpenoid | 21 nM (Dd2) |  | *Chloranthus fortunei* | 84 |
| 65 | Schizukaol M | Sesquiterpenoid | 96 nM (Dd2) |  | *Chloranthus fortunei* | 84 |
| 66 | Chlorajaponilide C | Sesquiterpenoid | 1.1 nM (Dd2) |  | *Chloranthus sp.* | 84 |
| 67 | Chlorahololide D | Sesquiterpenoid | 13 nM (Dd2) |  | *Chloranthus fortunei* | 84 |
| 68 | Shizukaol N | Sesquiterpenoid | 100 nM (Dd2) |  | *Chloranthus sp.* | 84 |
| 69 | Sarcandrolide B | Sesquiterpenoid | 265 nM (Dd2) |  | *Sarcandra glabra* | 84 |
| 70 | Sarcandrolide A | Sesquiterpenoid | 320 nM (Dd2) |  | *Sarcandra glabra* | 84 |
| 71 | Sarcandrolide J | Sesquiterpenoid | 11400 nM (Dd2) |  | *Sarcandra glabra* | 84 |
| 72 | Shizukaol E | Sesquiterpenoid | 1800 nM (Dd2) |  | *Chloranthus sp.* | 84 |
| 73 | Shizukaol D | Sesquiterpenoid | 580 nM (Dd2) |  | *Chloranthus sp.* | 84 |
| 74 | Shizukaol F | Sesquiterpenoid | 11 nM (Dd2) |  | *Chloranthus sp.* | 84 |
| 75 | Shizukaol G | Sesquiterpenoid | 13 nM (Dd2) |  | *Chloranthus sp.* | 84 |
| 76 | Shizukaol B | Sesquiterpenoid | 27 nM (Dd2) |  | *Chloranthus sp.* | 84 |
| 77 | Spicachlorantin D | Sesquiterpenoid | 474 nM (Dd2) |  | *Chloranthus sp.* | 84 |
| 78 | Shizukaol A | Sesquiterpenoid | 1500 nM (Dd2) |  | *Chloranthus sp.* | 84 |
| 79 | Chloramultilide B | Sesquiterpenoid | 7100 nM (Dd2) |  | *Chloranthus multisachys* | 84 |
| 80 | Cleistodienediol (New) | Polyoxygenated cylcohexene | 0.2 µM (3D7)  0.48 µM (Dd2) |  | *Cleistochlamys kirkii* (leaf) | 85 |
| 81 | Cleistodienol A | Polyoxygenated cylcohexene | 0.62 µM (3D7)  3.4 µM (Dd2) |  | *Cleistochlamys kirkii* (leaf) | 85 |
| 82 | Cleistodienol B (New) | Polyoxygenated cylcohexene | 1.2 µM (3D7)  2.2 µM (Dd2) |  | *Cleistochlamys kirkii* (leaf) | 85 |
| 83 | Cleistenechlorohydrin A (New) | Polyoxygenated cylcohexene | 72% inhibition at 40 µM (3D7) |  | *Cleistochlamys kirkii* (leaf) | 85 |
| 84 | Cleistenechlorohydrin B (New) | Polyoxygenated cylcohexene | 95% inhibition at 40 µM (3D7) |  | *Cleistochlamys kirkii* (leaf) | 85 |
| 85 | Cleistenediol F (New) | Polyoxygenated cylcohexene | 99% inhibition at 40 µM (3D7) |  | *Cleistochlamys kirkii* (leaf) | 85 |
| 86 | Cleistenonal (New) | Polyoxygenated cylcohexene | 98% inhibition at 40 µM (3D7) |  | *Cleistochlamys kirkii* (leaf) | 85 |
| 87 | Cleistophenolide (New) | Benzylbenzoate | 67% inhibition at 40 µM (3D7) |  | *Cleistochlamys kirkii* (leaf) | 85 |
| 88 | *ent*-subglain C | Polyoxygenated cylcohexene | 54% inhibition at 40 µM (3D7) |  | *Cleistochlamys kirkii* (leaf) | 85 |
| 89 | Melodorinol | Heptalactone | 92% inhibition at 40 µM (3D7) |  | *Cleistochlamys kirkii* (leaf) | 85 |
| 90 | Acetylmelodorinol | Heptalactone | 6.67 µM (Dd2)  100% inhibition at 40 µM (Dd2) |  | *Cleistochlamys kirkii* (leaf) | 85 |
| 91 | Tetramethylscutellarein | Flavonoid | 95% inhibition at 40 µM (3D7) |  | *Cleistochlamys kirkii* (leaf) | 85 |
| 92 | 2-hydroxybenzaldehyde | Aromatic aldehyde | 25% inhibition at 40 µM (3D7) |  | *Cleistochlamys kirkii* (leaf) | 85 |
| 93 | Fagaramide | Amide | 16.6 µg/ml (NF54)  67.12 µM  2.83 µg/ml (FCR3)  11.44 µM |  | *Zanthoxylum chalybeum* (stem bark) | 86 |
| 94 | Cripowellin A | Macrocyclic lactam | 30 nM (Dd2) |  | *Crinum erubescens* | 87 |
| 95 | Cripowellin B | Macrocyclic lactam | 180 nM (Dd2) |  | *Crinum erubescens* | 87 |
| 96 | Cripowellin C(New) | Macrocyclic lactam | 26 nM (Dd2) |  | *Crinum erubescens* | 87 |
| 97 | Cripowellin D(New) | Macrocyclic lactam | 260 nM (Dd2) |  | *Crinum erubescens* | 87 |
| 98 | Erythrinasinate B | Phenyl propanoid ester | 24.4 µg/ml (NF54)  41.6 µM |  | *Erythrina caffra* (stem bark) | 88 |
| 99 | Lupeol | Triterpene | 41.7 µg/ml (NF54)  97.7 µM |  | *Erythrina caffra* (stem bark) | 88 |
| 100 | Euphorbesulin A (New) | Diterpenoid | 2.41 µM (Dd2) |  | *Euphorbia esula* (twigs) | 89 |
| 101 | Euphorbesulin B (New) | Diterpenoid | >5 µM (Dd2) |  | *Euphorbia esula* (twigs) | 89 |
| 102 | Euphorbesulin C (New) | Diterpenoid | >5 µM (Dd2) |  | *Euphorbia esula* (twigs) | 89 |
| 103 | Euphorbesulin D (New) | Diterpenoid | >5 µM (Dd2) |  | *Euphorbia esula* (twigs) | 89 |
| 104 | Euphorbesulin E (New) | Diterpenoid | >5 µM (Dd2) |  | *Euphorbia esula* (twigs) | 89 |
| 105 | Euphorbesulin G(New) | Diterpenoid | 0.12 µM (Dd2) |  | *Euphorbia esula* (twigs) | 89 |
| 106 | Euphorbesulin H(New) | Diterpenoid | >5 µM (Dd2) |  | *Euphorbia esula* (twigs) | 89 |
| 107 | Euphorbesulin I (New) | Diterpenoid | >5 µM (Dd2) |  | *Euphorbia esula* (twigs) | 89 |
| 108 | Euphorbesulin J (New) | Diterpenoid | >5 µM (Dd2) |  | *Euphorbia esula* (twigs) | 89 |
| 109 | Euphorbesulin K(New) | Diterpenoid | >10 µM (Dd2) |  | *Euphorbia esula* (twigs) | 89 |
| 110 | Euphorbesulin L (New) | Diterpenoid | >5 µM (Dd2) |  | *Euphorbia esula* (twigs) | 89 |
| 111 | Euphorbesulin N (New) | Diterpenoid | >5 µM (Dd2) |  | *Euphorbia esula* (twigs) | 89 |
| 112 | *seco*-Dehydroantofine (New) | Seco-Phenanthroindo  lizine alkaloid | 4.0 µM (3D7) |  | *Ficus septica* (twigs) | 90 |
| 113 | Dehydrotylophorine | Phenanthroindolizine alkaloid | 0.42 µM (3D7) |  | *Ficus septica* (twigs) | 90 |
| 114 | Dehydroantofine | Phenanthroindolizine alkaloid | 0.028 µM (3D7) |  | *Ficus septica* (twigs) | 90 |
| 115 | Tylophoridicine | Phenanthroindolizine alkaloid | 0.058 µM (3D7) |  | *Ficus septica* (twigs) | 90 |
| 116 | Gambogic acid | Xanthone | 0.28 µM (Dd2) |  | *Garcinia*  (Gamboge) resin | 91 |
| 117 | Seco dienurvilleic acid (New) | Secocycloartane triterpene | 32.4 µM (FcB1) |  | *Gardenia urvillei* (bud exudate) | 92 |
| 118 | Gardheptlactone (New) | Secocycloartane triterpene | 26.7 µM (FcB1) |  | *Gardenia urvillei* (bud exudate) | 92 |
| 119 | Gardenolic B acid | Cycloartane triterpene | 54.1 µM (FcB1) |  | *Gardenia urvillei* (bud exudate) | 92 |
| 120 | Sootepin C | Secocycloartane triterpene | 10.1 µM (FcB1) |  | *Gardenia urvillei* (bud exudate) | 92 |
| 121 | Secaubryolide | Secocycloartane triterpene | 4.1 µM (FcB1) |  | *Gardenia urvillei* (bud exudate) | 92 |
| 122 | Polycarpol | Triterpene | 3.0 µM (K1) |  | *Greenwayodendron suaveolens* (root bark) | 93 |
| 123 | Dihydropolycarpol (New) | Triterpene | 10.3 µM (K1) |  | *Greenwayodendron suaveolens* (root bark) | 93 |
| 124 | Polyalthenol | Indolosesquiterpene alkaloid | 2.6 µM (K1) |  | *Greenwayodendron suaveolens* (root bark) | 93 |
| 125 | N-acetyl-polyveoline | Indolosesquiterpene alkaloid | 2.8 µM (K1) |  | *Greenwayodendron suaveolens* (root bark) | 93 |
| 126 | 3α-angeloyloxy-15-hydroxylabda-7,13-dien-16,15-olid-18-oic acid (New) | Labdane diterpenoid | 10.4 µM (Dd2) |  | *Gutierrezia sarothrae* | 94 |
| 127 | Ethyl gallate | Phenolic ester | 6.46 µM (D6)  9.34 µM (W) |  | *Koelreuteria paniculata* (arial parts) | 95 |
| 128 | Methyl gallate | Phenolic ester | 6.95 µM (D6)  4.18 µM (W2) |  | *Koelreuteria paniculata* (arial parts) | 95 |
| 129 | 3-O-trans-caffeoylbetulinic acid | Triterpene caffeate | 4.5 µM (K1) |  | *Lepisanthes senegalensis* (stem) | 96 |
| 130 | Maesargentoside I (New) | Triterpene saponin | 2.60 µM (K1) |  | *Maesa argentea* (leaf) | 97 |
| 131 | Maesargentoside II (New) | Triterpene saponin | 5.23 µM (K1) |  | *Maesa argentea* (leaf) | 97 |
| 132 | Maesargentoside III (New) | Triterpene saponin | 0.76 µM (K1) |  | *Maesa argentea* (leaf) | 97 |
| 133 | Maesargentoside IV (New) | Triterpene saponin | 1.50 µM (K1) |  | *Maesa argentea* (leaf) | 97 |
| 134 | Mallatojaponin C | Phenolic (Phlorogluciol dimer) | 0.64 µM (Dd2)  0.75 µM (FcB1) |  | *Mallotus oppositifolius* | 98 |
| 135 | Bergenin | Phenolic glycoside | 6.92 µM (D10) |  | *Mallotus philippensis* (stem wood) | 99 |
| 136 | 11-*O*-Galloylbergenin | Phenolic glycoside | 7.85 µM (D10) |  | *Mallotus philippensis (stem wood)* | 99 |
| 137 | Heliparvifoline | Furoquinoline alkaloid | 35 µM Dd2 |  | *Melicope madagascariensis* (stem bark) | 100 |
| 138 | Miliusacunine A (New) | Oxoprotoberberine Alkaloid | 19.3 µM (TM4)  26.3 µM (K1) |  | *Miliusa cuneata* (leaf) | 101 |
| 139 | Miliusacunine B (New) | Oxoprotoberberine Alkaloid | 25.6 µM (TM4)  10.8 µM (K1) |  | *Miliusa cuneata* (leaf) | 101 |
| 140 | Miliusacunine C(New) | Oxoprotoberberine Alkaloid | 41.4 µM (TM4)  32.4 µM (K1) |  | *Miliusa cuneata* (leaf) | 101 |
| 141 | Miliusacunine D(New) | Oxoprotoberberine Alkaloid | 38.1 µM (TM4)  26.1 µM (K1) |  | *Miliusa cuneata* (leaf) | 101 |
| 142 | Miliusacunine E (New) | Oxoprotoberberine Alkaloid | 29.1 µM (K1) |  | *Miliusa cuneata* (leaf) | 101 |
| 143 | 4’-hydroxy-3,5,7,3’-tetramethoxyflavone | Flavonoid | 31.5 µM (TM4)  27.9 µM (K1) |  | *Miliusa cuneata* (leaf) | 101 |
| 144 | (+)-miliusol | Lactone | 11.1 µM (TM4)  9.1 µM (K1) |  | *Miliusa cuneata* (leaf) | 101 |
| 145 | Chrysoplenetin | Flavonoid | 39.5 µM (TM4)  12.4 µM (K1) |  | *Miliusa cuneata* (twigs) | 101 |
| 146 | N-trans-feruloyltyramine | Amide | 54.9 µM (K1) |  | *Miliusa cuneata* (twigs) | 101 |
| 147 | N-trans-caffeoyltyramine | Amide | 23.8 µM (K1) |  | *Miliusa cuneata* (twigs) | 101 |
| 148 | Peperophthalide A (New) | Phthalide | 8.5 µM (F32) |  | *Peperomia nivalis* (aerial part) | 102 |
| 149 | Vanicoside F | Phenylpropanoid glycoside | 1.7 µg/ml (D6)  1.70 µM  1.77 µg/ml (W2)  1.77 µM |  | *Polygonum hydropiper* (aerial part) | 103 |
| 150 | 6,6’-((1α,2α,3β,4β)-2,4-diphenyl  cyclobutane-1,3-diyl)bis(4-methoxy-2*H*-pyran-2-one) | 2-Pyrone | 2.64 µg/ml (D6)  5.76 µM  1.91 µg/ml (W2)  4.17 µM |  | *Polygonum hydropiper* (aerial part) | 103 |
| 151 | Catechin | Flavonoid | 72.3 µM (FcB1) |  | *Psidium acutangulum* (aerial part) | 104 |
| 152 | Wayanin | Flavonoid glucoside | 5.5 µM (FcB1) |  | *Psidium acutangulum* (aerial part) | 104 |
| 153 | Reynoutrin | Flavonoid glucoside | 26.5 µM (FcB1) |  | *Psidium acutangulum* (aerial part) | 104 |
| 154 | Guaijaverin | Flavonoid glucoside | 6.9 µM (FcB1) |  | *Psidium acutangulum* (aerial part) | 104 |
| 155 | Avicularin | Flavonoid glucoside | 64.5 µM (FcB1) |  | *Psidium acutangulum* (aerial part) | 104 |
| 156 | Quercitrin | Flavonoid glucoside | 71.4 µM (FcB1) |  | *Psidium acutangulum* (aerial part) | 104 |
| 157 | 9-oxoeuryopsin | Furanosesquiterpene | 5.2 µM (INDO) |  | *Senecio smithioides* (aerial part) | 105 |
| 158 | (±)-rhodomyrtosone F (New) | Phloroglucinol | 0.1 µM (Dd2) |  | *Syncarpia glomulifera* (stem bark) | 106 |
| 159 | (±)-calliviminone C (New) | Phloroglucinol | 3.81 µM (Dd2) |  | *Syncarpia glomulifera* (stem bark) | 106 |
| 160 | Betulinic acid | Triterpene | 3.44 µM (Dd2) |  | *Syncarpia glomulifera* (stem bark) | 106 |
| 161 | Ursolic acid-3-acetate | Triterpene | 12.09 µM (Dd2) |  | *Syncarpia glomulifera* (stem bark) | 106 |
| 162 | Dihydronitidine | Benzophenanthridine alkaloid | 0.025 µM (3D7) |  | *Zanthoxylum heitzii* (bark) | 107 |
| 163 | Pellitorine | Alkamide | 8.8 µM (3D7) |  | *Zanthoxylum heitzii* (bark) | 107 |
| 164 | Heitziquinone | Benzophenanthridine alkaloid | 9.7 µM (3D7) |  | *Zanthoxylum heitzii* (bark) | 107 |
| 165 | Nummularine-R | Cyclopeptide alkaloid | 3.2 µM (K1) |  | *Ziziphus oxyphylla* (root) | 108 |
| 166 | O-desmethylnummularine-R (New) | Cyclopeptide alkaloid | 7.1 µM (K1) |  | *Ziziphus oxyphylla* (root) | 108 |
| 167 | Hemsine-A | Cyclopeptide alkaloid | 13.6 µM (K1) |  | *Ziziphus oxyphylla* (root) | 108 |
| 168 | Ramosine-A | Cyclopeptide alkaloid | >32.0 µM (K1) |  | *Ziziphus oxyphylla* (root) | 108 |
| 169 | Oxyphylline-F (New) | Cyclopeptide alkaloid | 7.4 µM (K1) |  | *Ziziphus oxyphylla* (root) | 108 |
| 170 | Hymenocardine | Cyclopeptide alkaloid | 16.4 µM (K1) |  | *Hymenocardia acida* (root bark) | 109 |
| 171 | Hymenocardinol (New) | Cyclopeptide alkaloid | 17.5 µM (K1) |  | *Hymenocardia acida* (root bark) | 109 |
| 172 | Hymenocardine N-Oxide (New) | Cyclopeptide alkaloid | 12.2 µM (K1) |  | *Hymenocardia acida* (root bark) | 109 |
| 173 | Hymenocardine-H (New) | Cyclopeptide alkaloid | 27.9 µM (K1) |  | *Hymenocardia acida* (root bark) | 109 |
| 174 | Diacarnuperoxide M (New) | Norditerpene cyclic peroxide | 4.2 µM (W2)  5.6 µM (D6) | Sponge | *Diacarnus megaspinorhabdosa* | 110 |
| 175 | Diacarnuperoxide N (New) | Norditerpene cyclic peroxide | 3.0 µM (W2)  6.6 µM (D6) | Sponge | *Diacarnus megaspinorhabdosa* | 110 |
| 176 | (+)-2,3,6-epihurghaperoxide (New) | Norditerpene cyclic peroxide | 1.6 µM (W2)  2.2 µM (D6) | Sponge | *Diacarnus megaspinorhabdosa* | 110 |
| 177 | (+)-2,3,6-epihurghaperoxide acid (New) | Norditerpene cyclic peroxide | 4.9 µM (W2)  7.3 µM (D6) | Sponge | *Diacarnus megaspinorhabdosa* | 110 |
| 178 | (-)-muqubilin A | Norditerpene cyclic peroxide | 5.6 µM (W2)  8.6 µM (D6) | Sponge | *Diacarnus megaspinorhabdosa* | 110 |
| 179 | Nuapapuin A | Norditerpene cyclic peroxide | 5.5 µM (W2)  8.1 µM (D6) | Sponge | *Diacarnus megaspinorhabdosa* | 110 |
| 180 | Diacarperoxide A | Norditerpene cyclic peroxide | 1.9 µM (W2)  2.0 µM (D6) | Sponge | *Diacarnus megaspinorhabdosa* | 110 |
| 181 | 1-acetoxychevalone C (New) | Meroterpenoid | 6.67 µM (K1) | Fungus | *Neosartorya spinosa* KKU-1NK1 | 111 |
| 182 | Tryptoquivaline | Indole alkaloid | 2.65 µM (K1) | Fungus | *Neosartorya spinosa* KKU-1NK1 | 111 |
| 183 | Isochromophilone VI | Azaphilone (Isochrmomene) | 12.2 µM (3D7) | Fungus | *Penicillium sp.* KCB11A109 | 112 |
| 184 | Efomycin M | Macrolide | 5.23 µg/ml (K1)  7.17 µM | Actinomycete | *Streptomyces sp.* BCC72023 | 113 |
| 185 | Efomycin G | Macrolide | 2.37 µg/ml (K1)  2.34 µM | Actinomycete | *Streptomyces sp.* BCC72023 | 113 |
| 186 | Oxohygrolidin | Macrolide | 2.30 µg/ml (K1)  4.00 µM | Actinomycete | *Streptomyces sp.* BCC72023 | 113 |
| 187 | Abierixin | Macrolide | 2.58 µg/ml (K1)  3.56 µM | Actinomycete | *Streptomyces sp.* BCC72023 | 113 |
| 188 | 29-O-methylabierixin | Macrolide | 1.40 µg/ml (K1)  1.89 µM | Actinomycete | *Streptomyces sp.* BCC72023 | 113 |

2015

| 1 | 1,7-dihydroxyxanthone | Xanthone | 16.05 µg/ml (F32)  70.33 µM  17.93 µg/ml (FcM29)  78.57 µM | Plant | *Allanblackia sp.* | 114 |
| --- | --- | --- | --- | --- | --- | --- |
| 2 | Macluraxanthone | Xanthone | 0.46 µg/ml (F32)  1.16 µM  0.33 µg/ml (FcM29)  0.83 µM | Plant | *Allanblackia sp.* | 114 |
| 3 | Allaxanthone B | Xanthone | 3.70 µg/ml (F32)  7.93 µM  3.93 µg/ml (FcM29)  8.42 µM | Plant | *Allanblackia sp.* | 114 |
| 4 | Morelloflavone | Biflavonoid | 11.77 µg/ml (F32)  21.15 µM  12.59 µg/ml (FcM29)  22.62 µM | Plant | *Allanblackia sp.* | 114 |
| 5 | Volkensiflavone | Biflavonoid | 0.99 µg/ml (F32)  1.83 µM  0.93 µg/ml (FcM29)  1.72 µM | Plant | *Allanblackia sp.* | 114 |
| 6 | Morelloflavone -7-*O-*glucoside | Biflavonoid glycoside | 11.45 µg/ml (F32)  15.93 µM  28.92 µg/ml (FcM29)  40.25 µM | Plant | *Allanblackia sp.* | 114 |
| 7 | Gallic acid | Phenolic | 3.32 µg/ml (W2)  19.51 µM |  | *Annona muricata* (stem bark) | 115 |
| 8 | Antrocarine A(New) | Sterol | 22.0 µM (3D7) |  | *Antrocaryon klaineanum* (stem bark) | 116 |
| 9 | Antrocarine B(New) | Sterol | 11.2 µM (3D7) |  | *Antrocaryon klaineanum (stem bark)* | 116 |
| 10 | 7,20S-dihydroxyergosta-4,24(28)-dien-3-one | Sterol | 21.3 µM (3D7) |  | *Antrocaryon klaineanum (stem bark)* | 116 |
| 11 | Amorphaquinone | Benzpyran quinone | 5.7 µM (Dd2) |  | *Apoplanesia paniculata* (root) | 117 |
| 12 | Pendulone | Benzpyran quinone | 7.0 µM (Dd2) |  | *Apoplanesia paniculata* (root) | 117 |
| 13 | Melilotocarpan C | Pterocarpan | 41.8 µM (Dd2) |  | *Apoplanesia paniculata* (root) | 117 |
| 14 | Uleine | Indole alkaloid | 11.9 µg/ml (3D7)  44.67 µM  0.75 µg/ml (W2)  2.81 µM |  | *Aspidosperma parvifolium* (bark) | 118 |
| 15 | 3-*O*-(*E*)-*p*-coumaroyl-23-hydroxybetulin (New) | Lupane triterpene | 0.65 µM (Dd2) |  | *Buxus cochinchinensis* (leaf, twig and fruit) |  |
| 16 | 3-*O*-(*E*)-*p*-coumaroyl-23-hydroxy-3-*epi*-betulin (New) | Lupane triterpene | 1.28 µM (Dd2) |  | *Buxus cochinchinensis* (leaf, twig and fruit) |  |
| 17 | 3-*O*-(*Z*)-*p*-coumaroyl-23-hydroxy-3-*epi*-betulin (New) | Lupane triterpene | 1.02 µM (Dd2) |  | *Buxus cochinchinensis* (leaf, twig and fruit) | 119 |
| 18 | 23-*O*-(*E*)-*p*-coumaroyl-23-hydroxybetulin (New) | Lupane triterpene | 0.26 µM (Dd2) |  | *Buxus cochinchinensis* (leaf, twig and fruit) | 119 |
| 19 | 23-*O*-(*Z*)-*p*-coumaroyl-23-hydroxybetulin (New) | Lupane triterpene | 0.63 µM (Dd2) |  | *Buxus cochinchinensis* (leaf, twig and fruit) | 119 |
| 20 | *N*-3-benzoyldihydrocyclomicrophy  lline F | Alkaloid | 2.07 µM (Dd2) |  | *Buxus cochinchinensis* (leaf, twig and fruit) | 119 |
| 21 | 4,4'-dihydroxy-2'-methoxy-chalc  One | Chalcone | 33 µM (FcR3) |  | *Caesalpinia bonduc* (twig) | 120 |
| 22 | Caesalsappanin A (New) | Cassane diterpene | 7.4 µM (K1) |  | *Caesalpinia sappan* (seed) | 121 |
| 23 | Caesalsappanin B (New) | Cassane diterpene | 34.8 µM (K1) |  | *Caesalpinia sappan* (seed) | 121 |
| 24 | Caesalsappanin C (New) | Cassane diterpene | 32.1 µM (K1) |  | *Caesalpinia sappan* (seed) | 121 |
| 25 | CaesalsappaninD (New) | Cassane diterpene | 28.4 µM (K1) |  | *Caesalpinia sappan* (seed) | 121 |
| 26 | Caesalsappanin E (New) | Cassane diterpene | 15.7 µM (K1) |  | *Caesalpinia sappan* (seed) | 121 |
| 27 | Caesalsappanin F (New) | Cassane diterpene | 19.2 µM (K1) |  | *Caesalpinia sappan* (seed) | 121 |
| 28 | Caesalsappanin G (New) | Cassane diterpene | 0.78 µM (K1) |  | *Caesalpinia sappan* (seed) | 121 |
| 29 | Caesalsappanin H (New) | Cassane diterpene | 0.52 µM (K1) |  | *Caesalpinia sappan* (seed) | 121 |
| 30 | Caesalsappanin I (New) | Cassane diterpene | 2.5 µM (K1) |  | *Caesalpinia sappan* (seed) | 121 |
| 31 | Caesalsappanin J (New) | Cassane diterpene | 29.7 µM (K1) |  | *Caesalpinia sappan* (seed) | 121 |
| 32 | Caesalsappanin K (New) | Cassane diterpene | 41.2 µM (K1) |  | *Caesalpinia sappan* (seed) | 121 |
| 33 | Caesalsappanin L (New) | Cassane diterpene | 38.9 µM (K1) |  | *Caesalpinia sappan* (seed) | 121 |
| 34 | (S)-10-hydroxycannabinol (New) | Cannabinoid | 3.4 µg/ml (D6)  10.42 µM  2.3 µg/ml (W2)  7.05 µM |  | *Cannabis sativa* (leaf and bud) | 122 |
| 35 | 3α-O-(β-D-Glucopyranosyl)desoxy  podophyllotoxin (New) | Lignan | 12.6 µM (Dd2) |  | *Cleistanthus boivinianus* (leaf) | 123 |
| 36 | (2S)-1,2-di-O-[(9Z)-octadeca-9-  enoyl]-3-O-β-D-galactopyranosyl glycerol |  | 34 µg/ml (NF54)  40.51 µM |  | *Conyza sumatrensis* (leaf) | 124 |
| 37 | (2S)-1,2-di-O-[(9Z,12Z,15Z)-octa  deca-9,12,15-trienoyl]-3-O-(6-sulpho-α-D) quinovopyranosyl glycerol |  | 17.9 µg/ml (NF54)  21.53 µM |  | *Conyza sumatrensis* (leaf) | 124 |
| 38 | 1-O-β-D-glucopyranosyl-(2S,3R,8E)  -2-[(2′R)-2-hydroxy-palmitoylamino  ]-8-octadecene-1,3-diol |  | 18 µg/ml (NF54)  22.43 µM |  | *Conyza sumatrensis* (leaf) | 124 |
| 39 | 3-O-β-D-glucopyranosyl-3,4-dihydroxybenzoic acid | Phenolic glycoside | 25 µg/ml (NF54)  79.04 µM |  | *Conyza sumatrensis* (leaf) | 124 |
| 40 | Gardenin C | Flavonoid | 10.1 µg/ml (3D7)  25 µM |  | *Crassocephalum bauchiense* (whole plant) | 125 |
| 41 | Cryptorigidifoliol A (New) | 2-pyrone | 9.2 µM (Dd2) |  | *Cryptocarya rigidifolia* (root wood) | 126 |
| 42 | Cryptorigidifoliol B (New) | 2-pyrone | 5.8 µM (Dd2) |  | *Cryptocarya rigidifolia* (root wood) | 126 |
| 43 | Cryptorigidifoliol C (New) | 2-pyrone | 5.5 µM (Dd2) |  | *Cryptocarya rigidifolia* (root wood) | 126 |
| 44 | Cryptorigidifoliol D (New) | 2-pyrone | 7.4 µM (Dd2) |  | *Cryptocarya rigidifolia* (root wood) | 126 |
| 45 | Cryptorigidifoliol E (New) | 2-pyrone | 9.0 µM (Dd2) |  | *Cryptocarya rigidifolia* (root wood) | 126 |
| 46 | Cryptorigidifoliol F (New) | 2-pyrone | 4.0 µM (Dd2) |  | *Cryptocarya rigidifolia* (root wood) | 126 |
| 47 | Cryptorigidifoliol G (New) | 2-pyrone | 6.0 µM (Dd2) |  | *Cryptocarya rigidifolia* (root wood) | 126 |
| 48 | Cryptorigidifoliol H (New) | 2-pyrone | >10 µM (Dd2) |  | *Cryptocarya rigidifolia* (root wood) | 126 |
| 49 | Cryptorigidifoliol I (New) | 2-pyrone | >10 µM (Dd2) |  | *Cryptocarya rigidifolia* (root wood) | 126 |
| 50 | Cryptorigidifoliol J (New) | 2-pyrone | >10 µM (Dd2) |  | *Cryptocarya rigidifolia* (root wood) | 126 |
| 51 | Cryptorigidifoliol K (New) | 2-pyrone | >10 µM (Dd2) |  | *Cryptocarya rigidifolia* (root wood) | 126 |
| 52 | Naghibione (New) | Sesquiterpene | 68.1% suppression of P. berghei parasite |  | *Dorema hyrcanum* (root) | 127 |
| 53 | Prototiamin A (New) | Triterpene | 0.67 µM (NF54) |  | *Entandrophragma congoense* (bark) | 128 |
| 54 | Prototiamin B (New) | Triterpene | 1.3 µM (NF54) |  | *Entandrophragma congoense* (bark) | 128 |
| 55 | Prototiamin C (New) | Triterpene | 0.44 µM (NF54) |  | *Entandrophragma congoense* (bark) | 128 |
| 56 | Prototiamin D (New) | Triterpene | 2.0 µM (NF54) |  | *Entandrophragma congoense* (bark) | 128 |
| 57 | Prototiamin E (New) | Triterpene | 0.87 µM (NF54) |  | *Entandrophragma congoense* (bark) | 128 |
| 58 | Prototiamin F (New) | Triterpene | 1.4 µM (NF54) |  | *Entandrophragma congoense* (bark) | 128 |
| 59 | Apotirucallane-type triterpene | Triterpene | 1.3 µM (NF54) |  | *Entandrophragma congoense* (bark) | 128 |
| 60 | Prototiamin G (New) | Triterpene | 1.3 µM (NF54) |  | *Entandrophragma congoense* (bark) | 128 |
| 61 | seco-Tiaminic acid A (New) | Triterpene | 19.3 µM (NF54) |  | *Entandrophragma congoense* (bark) | 128 |
| 62 | Congoensin A (New) | Triterpene | 5.5 µM (NF54) |  | *Entandrophragma congoense* (bark) | 129 |
| 63 | Congoensin B (New) | Triterpene | 6.1 µM (NF54) |  | *Entandrophragma congoense* (bark) | 129 |
| 64 | Gladoral A | Triterpene | 2.4 µM (NF54) |  | *Entandrophragma congoense* (bark) | 129 |
| 65 | Bipendensin | Phenolic acetal | 24.5 µM (NF54) |  | *Entandrophragma congoense* (bark) | 129 |
| 66 | GB-1a (1) 4’,II-4’,I-5,II-5,I-7,II-7-hexahydroxy-I-3,II-8-biflavanone | Biflavonoid | 0.65 µM (FCR3) |  | *Garcinia kola* (nuts) | 130 |
| 67 | GB-1 (2) II-3,I-4’,II-4’,I-5,II-5,I-7,II-7-heptahydroxy-I-3,II- 8-biflavanone | Biflavonoid | 0.16 µM (FCR3)  52.4% inhibition of P. berghei parasite at 100mg/kg |  | *Garcinia kola* (nuts) | 130 |
| 68 | GB-2 (3) 2 or II-3,3’,I-4’,II-4’,I-5,II-5,I-7, II-7-octahydroxy-I-3,II-8-biflavanone | Biflavonoid | 0.21 µM (FCR3) |  | *Garcinia kola* (nuts) | 130 |
| 69 | 5,7-dihydroxy-3,3',4',6-Tetramethoxyflavone | Flavonoid | 21.3% inhibition at 10 µM (FcB1).  Synthetic analogue IC50 (5.8 µM). |  | *Gardenia oudiepe* (bud exudate) | 131 |
| 70 | (+)-altholactone | Styryllactone | 2.8 µg/ml (K1)  12.05 µM |  | *Goniothalamus elegants* (bark) | 132 |
| 71 | (+)-goniopypyrone | Styryllactone | 4.46 µg/ml (K1)  17.82 µM |  | *Goniothalamus elegants* (bark) | 132 |
| 72 | (+)-goniodiol | Styryllactone | 3.28 µg/ml (K1)  14.00 µM |  | *Goniothalamus elegants* (bark) | 132 |
| 73 | (+)-goniothalamin oxide | Styryllactone | 2.28 µg/ml (K1)  10.54 µM |  | *Goniothalamus elegants* (bark) | 132 |
| 74 | (+)-goniothalamin | Styryllactone | 2.65 µg/ml (K1)  13.23 µM |  | *Goniothalamus elegants* (bark) | 132 |
| 75 | Velutinam | Aristolactam alkaloid | 5.89 µg/ml (K1)  19.94 µM |  | *Goniothalamus elegants* (bark) | 132 |
| 76 | Myricetin | Flavonoid | 1.82 µg/ml (D6)  5.71 µM  1.51 µg/ml (W2)  4.74 µM |  | *Limonium caspium* (aerial parts) | 133 |
| 77 | 4-O-geranylisoliquiritigenin (New) | Flavonoid | 3.67 µM (3D7)  6.97 µM (Dd2) |  | *Millettia*  *Usaramensis* (root bark) | 134 |
| 78 | Tephrosin | Retinoid | 13.27 µM (3D7)  12.05 µM (Dd2) |  | *Millettia*  *Usaramensis* (root bark) | 134 |
| 79 | Balsaminoside B | Triterpene | 36.2% suppression of P. berghei parasite at 50mg/kg |  | *Momordica balsamina* (aerial part) | 135 |
| 80 | Karavilagenin C | Triterpene | 33.1% suppression of P. berghei parasite at 50mg/kg |  | *Momordica balsamina* (aerial part) | 135 |
| 81 | Neoboutomacroin (New) | Diterpenoid | 19.3 µM (D6)  14.2 µM (W2) |  | *Neoboutonia macrocalyx* (stem bark) | 136 |
| 82 | 3-*O*-Acetyloleuritolic acid | triterpene | 30.9 µM (D6)  9.0 µM (W2) |  | *Neoboutonia macrocalyx* (stem bark) | 136 |
| 83 | Simplexin | Diterpenoid | 64.2 µM (D6)  57.9 µM (W2) |  | *Neoboutonia macrocalyx* (stem bark) | 136 |
| 84 | Montanin | Diterpenoid | 22.7 µM (D6)  10.2 µM (W2) |  | *Neoboutonia macrocalyx* (stem bark) | 136 |
| 85 | (*S*)-virolongin B | Neolignan | 3.3 µM (Dd2) |  | *Ocotea cymosa* (stem) | 137 |
| 86 | Ococymosin (New) | Neolignan | 0.45 µM (Dd2) |  | *Ocotea cymosa* (stem) | 137 |
| 87 | Demethoxysibyllenone (New) | Neolignan | 14.6 µM (Dd2) |  | *Ocotea cymosa* (stem) | 137 |
| 88 | Demethylsibyllenone (New) | Neolignan | 42 µM (Dd2) |  | *Ocotea cymosa* (stem) | 137 |
| 89 | (7R,8R,1’R,3’R)-Δ8’-3,4,5-Trimethoxy-3’,4’-methylenedioxy-1’,2’,3’,6’-tetrahydro-6’-oxo-7.1’-8.3’-neolignan (New) | Neolignan | 7.7 µM (Dd2) |  | *Ocotea cymosa* (stem) | 137 |
| 90 | Ocobullenone | Neolignan | 4.1 µM (Dd2) |  | *Ocotea cymosa* (stem) | 137 |
| 91 | Bisclerodane imide | Clerodane diterpene | 4.53 µM (3D7) |  | *Polyalthia longifolia* (stem bark) | 138 |
| 92 | cleroda-3-ene, pyrrole-15,16-dione (New) | Clerodane diterpene | 4.76 µM (3D7) |  | *Polyalthia longifolia* (stem bark) | 138 |
| 93 | cleroda-3-ene pyrrolidine-15,16-dione (New) | Clerodane diterpene | 112.14 µM (3D7) |  | *Polyalthia longifolia* (stem bark) | 138 |
| 94 | cleroda-3,13(14)-dien- 15,16-diamide (New) | Clerodane diterpene | 67.12 µM (3D7) |  | *Polyalthia longifolia* (stem bark) | 138 |
| 95 | cleroda- 3-ene-15,16-diamide (New) | Clerodane diterpene | 10.17 µM (3D7) |  | *Polyalthia longifolia* (stem bark) | 138 |
| 96 | 16-Hydroxycleroda-3,13-dien-16,15-olide | Clerodane diterpene | 16.76 µM (K1) |  | *Polyalthia longifolia* (stem bark) | 139 |
| 97 | 16-Oxocleroda-3,13(14)E-dien-15-oic acid | Clerodane diterpene | 9.59 µM (K1) |  | *Polyalthia longifolia* (stem bark) | 139 |
| 98 | 3,16-Dihydroxycleroda-4(18),13  (14)Z-dien-15,16-olide | Clerodane diterpene | 18.41 µM (K1) |  | *Polyalthia longifolia* (stem bark) | 139 |
| 99 | Stigmasterol | Sterol | 153.79 µM (K1) |  | *Polyalthia longifolia* (stem bark) | 139 |
| 100 | Darienine | Azafluorenone alkaloid | 81.37 µM (K1) |  | *Polyalthia longifolia* (stem bark) | 139 |
| 101 | L-Stepholidine | Proto[berberine](https://en.wikipedia.org/wiki/Berberine) alkaloid | 319.05 µM (K1) |  | *Polyalthia longifolia* (stem bark) | 139 |
| 102 | Diacetylpiptocarphol | Sesquiterpenoid | 7.8 µM (FcM29) |  | *Pseudelephantopus spiralis* (aerial part) | 140 |
| 103 | Piptocarphin A | Sesquiterpenoid | 6.9 µM (FcM29) |  | *Pseudelephantopus spiralis* (aerial part) | 140 |
| 104 | Piptocarphin D | Sesquiterpenoid | 50 µM (FcM29) |  | *Pseudelephantopus spiralis* (aerial part) | 140 |
| 105 | (1S*,4R*,8S*,10R*)-1,4-epoxy-13-ethoxy-1,8,10- trihydroxygermacra-5E,7(11)-dien-6,12-olide | Sesquiterpenoid | 54.9 µM (FcM29) |  | *Pseudelephantopus spiralis* (aerial part) | 140 |
| 106 | 13-Demethoxy-11(S*),12(R*)-dihydroprotostemonine | Pyrrolidine alkaloid | 17.7 µg/ml (TM4)  45.47 µM  16.8 µg/ml (K1)  43.16 µM |  | *Stemona javanica* (root) | 141 |
| 107 | Protostemonine | Pyrrolidine alkaloid | 16.8 µg/ml (TM4)  40.23 µM  14.1 µg/ml (K1)  33.77 µM |  | *Stemona javanica* (root) | 141 |
| 108 | Isoprotostemonine | Pyrrolidine alkaloid | 16.0 µg/ml (TM4)  38.32 µM  11.9 µg/ml (K1)  28.5>99.94 µM |  | *Stemona javanica* (root) | 141 |
| 109 | Javastemonine A (New) | Pyrrolidine alkaloid | >38.9 µg/ml (TM4)  >99.94 µM  >38.9 µg/ml (K1)  >99.94 µM |  | *Stemona javanica* (root) | 141 |
| 110 | Javastemonine B (New) | Pyrrolidine alkaloid | >41.7 µg/ml (TM4)  >99.94 µM  >41.7 µg/ml (K1)  >99.94 µM |  | *Stemona javanica* (root) | 141 |
| 111 | Azadirone | Limonoid | 23.4 µM (D6)  29.6 µM (W2) |  | *Turraea robusta* (stem bark) and *Turraea nilotica* (root bark) | 142 |
| 112 | 12α-Acetoxy-7-deacetylazadirone | Limonoid | 31.0 µM (D6)  30.2 µM (W2) |  | *Turraea robusta* (stem bark) *and Turraea nilotica* (root bark) | 142 |
| 113 | Mzikonone | Limonoid | 36.6 µM (D6)  40.5 µM (W2) |  | *Turraea robusta* (stem bark) *and Turraea nilotica* (root bark) | 142 |
| 114 | 11-epi-Toonacilin | seco limonoid | 17.1 µM (D6)  14.4 µM (W2) |  | *Turraea robusta (ste, bark)* | 142 |
| 115 | Azadironolide | Triterpene | 2.4 µM (D6)  1.1 µM (W2) |  | *Turraea nilotica (root bark)* | 142 |
| 116 | Niloticin | Protolimonoid | 48.2 µM (D6)  77.0 µM (W2) |  | *Turraea nilotica* (root bark) | 142 |
| 117 | Hispidol B | Protolimonoid | 36.8 µM (D6)  37.2 µM (W2) |  | *Turraea nilotica* (root bark) | 142 |
| 118 | Piscidinol A | Protolimonoid | 37.6 µM (D6)  36.3 µM (W2) |  | *Turraea nilotica* (root bark) | 142 |
| 119 | Squalene | Triterpene | 1.7 µM (FcM29) |  | *Uapaca paludosa* (trunk bark) | 143 |
| 120 | Samvisterin (New) | Triterpene | 10.4 µM (FcM29) |  | *Uapaca paludosa* (trunk bark) | 143 |
| 121 | Lupeol | Triterpene | 70.4 µM (FcM29) |  | *Uapaca paludosa* (trunk bark) | 143 |
| 122 | β-sitosterol | Sterol | 31.8 µM (FcM29) |  | *Uapaca paludosa* (trunk bark) | 143 |
| 123 | Stigmasterol | Sterol | >120 µM (FcM29) |  | *Uapaca paludosa* (trunk bark) | 143 |
| 124 | Betulin | Triterpene | 6.8 µM (FcM29) |  | *Uapaca paludosa* (trunk bark) | 143 |
| 125 | Betulinic acid | Triterpene | 3.7 µM (FcM29) |  | *Uapaca paludosa* (trunk bark) | 143 |
| 126 | 4,5-dicaffeoyl quinic acid methyl ester | Phenolic ester | 3.29 µg/ml (D6)  6.2 µM  4.53 µg/ml (W2)  8.54 µM |  | *Vangueria edulis* (leaf) | 144 |
| 127 | Chevalone C | Meroterpenoid | 25 µg/ml (K1)  54.78 µM | Fungus | *Xylaria cf. cubensis* PK108 | 145 |
| 128 | Helvolic acid | Triterpene | 6.25 µg/ml (K1)  10.98µM | Fungus | *Xylaria cf. cubensis* PK108 | 145 |
| 129 | Penicilleremophilane A (New) | Sesqiterpene | 3.45 µM (K1) | Fungus | *Penicillium copticola* PSU-RSPG138 | 146 |
| 130 | (3R,4R,5R,6R,7R)-sporogen AO-1 | Sesqiterpene | 1.53 µM (K1) | Fungus | *Penicillium copticola* PSU-RSPG138 | 146 |
| 131 | (3R,4R,5R,6R,7R)-phomenone | Sesqiterpene | 5.41 µM (K1) | Fungus | *Penicillium copticola* PSU-RSPG138 | 146 |
| 132 | (3R,4R,5R,6R,7R)-3-acetyl-13-deoxyphomenone | Sesqiterpene | 4.55 µM (K1) | Fungus | *Penicillium copticola* PSU-RSPG138 | 146 |
| 133 | (3R,4R,- 5R,7S)-petasol | Sesqiterpene | 39.32 µM (K1) | Fungus | *Penicillium copticola* PSU-RSPG138 | 146 |
| 134 | 3α,13-dihydroxyeremophila-6,9,11-triene-8-one | Sesqiterpene | 15.71 µM (K1) | Fungus | *Penicillium copticola* PSU-RSPG138 | 146 |
| 135 | Entonalactam A (New) | Isoindolinone alkaloid | 36% inhibition at 50 µM (3D7) | Fungus | *Entonaema sp.* | 147 |
| 136 | Entonalactam B (New) | Isoindolinone alkaloid | 18% inhibition at 50 µM (3D7) | Fungus | *Entonaema sp.* | 147 |
| 137 | Entonalactam C (New) | Isoindolinone alkaloid | 26% inhibition at 50 µM (3D7) | Fungus | *Entonaema sp.* | 147 |
| 138 | 3-methoxy-5-methylbenzene-1,2-diol | Phenolic | 32% inhibition at 50 µM (3D7) | Fungus | *Entonaema sp.* | 147 |
| 139 | Daldinal B | Benzophenone | 39% inhibition at 50 µM (3D7) | Fungus | *Entonaema sp.* | 147 |
| 140 | ergosta-4,6,8(14),22-tetraen-3-one | Sterol | 66% inhibition at 50 µM (3D7) | Fungus | *Entonaema sp.* | 147 |
| 141 | Bastimolide A (New) | Macrolide | 80 nM (TM90-C2A)  90 nM (TM90-C2B)  140 nM (W2)  270 nM (TM91-C235) | Cyanobacterium | *Okeania hirsuta* | 148 |
| 142 | (−)-(1S,5S,8R)-2-Isocyanoclovene (New) | Isocyano sesquiterpene | 0.30 µM (3D7)  0.36 µM (Dd2) | Nudibranch | *Phyllidia ocellata* | 149 |
| 143 | (−)-(1S,5S,8R)-2-Isocyanoclovane (New) | Isocyano sesquiterpene | 0.29 µM (3D7)  0.83 µM (Dd2) | Nudibranch | *Phyllidia ocellata* | 149 |
| 144 | (+)-(1S*,4R*,5S*,10S*)-4,5-Epi-10-isocyanoisodauc-6-ene (New) | Isocyano sesquiterpene | 0.26 µM (3D7)  0.87 µM (Dd2) | Nudibranch | *Phyllidia ocellata* | 149 |
| 145 | Salinipostin A (New) | Bicyclic phosphotriester | 50 nM (W2) | Actinobacteria | *Salinispora sp.* | 150 |
| 146 | Salinipostin B (New) | Bicyclic phosphotriester | 0.139 µM (W2) | Actinobacteria | *Salinispora sp.* | 150 |
| 147 | Salinipostin C (New) | Bicyclic phosphotriester | 0.415 µM (W2) | Actinobacteria | *Salinispora sp.* | 150 |
| 148 | Salinipostin D (New) | Bicyclic phosphotriester | 0.082 µM (W2) | Actinobacteria | *Salinispora sp.* | 150 |
| 149 | Salinipostin E (New) | Bicyclic phosphotriester | 3.22 µM (W2) | Actinobacteria | *Salinispora sp.* | 150 |
| 150 | Salinipostin F (New) | Bicyclic phosphotriester | 0.266 µM (W2) | Actinobacteria | *Salinispora sp.* | 150 |
| 151 | Salinipostin G (New) | Bicyclic phosphotriester | 1.52 µM (W2) | Actinobacteria | *Salinispora sp.* | 150 |
| 152 | Salinipostin H (New) | Bicyclic phosphotriester | 8.7 µM (W2) | Actinobacteria | *Salinispora sp.* | 150 |
| 153 | Salinipostin I (New) | Bicyclic phosphotriester | 0.126 µM (W2) | Actinobacteria | *Salinispora sp.* | 150 |
| 154 | Salinipostin J (New) | Bicyclic phosphotriester | 49.6 µM (W2) | Actinobacteria | *Salinispora sp.* | 150 |
| 155 | Salinipostin K (New) | Bicyclic phosphotriester | 32.0 µM (W2) | Actinobacteria | *Salinispora sp.* | 150 |
| 156 | Monamphilectine B (New) | Diterpene | 44.5 nM (3D7) | Marine sponge | *Svenzea flava* | 151 |
| 157 | Monamphilectine C (New) | Diterpene | 43.3 nM (3D) | Marine sponge | *Svenzea flava* | 151 |
| 158 | Netamine O (New) | Guanidine alkaloid | 16.99 µM | Marine sponge | *Biemna laboutei* | 152 |
| 159 | Netamine P (New) | Guanidine alkaloid | 32.62 µM | Marine sponge | *Biemna laboutei* | 152 |
| 160 | Netamine Q (New) | Guanidine alkaloid | 8.37 µM | Marine sponge | *Biemna laboutei* | 152 |
| 161 | Piperafizine A | Peptide | 6.57 µM (Dd2) | Actinobacteria | *Streptomyces sp.* MMS-0085-C-XT | 153 |
| 162 | Xestostreptin (New) | Peptide | 50.5 µM (Dd2) | Actinobacteria | *Streptomyces sp.* XM6014 | 153 |
| 163 | Territrem B |  | 2.83 µg/ml (K1)  5.37 µM | Fungus | *Aspergillus terreus* | 154 |
| 164 | Actinoramide A (New) | Peptide | 200 nM | Acinobacteria | *Streptomyces bangulaensis* | 155 |
| 165 | α-Mangostin | Xanthone | 0.2 µM (FCR3)  36.10 µM (3D7)  80% suppression of parasite in P. berghei infected mice at 100mg/kg | Plant | *Garcinia mangostana* (husk) | 156 |
| 166 | δ-mangostin (New) | Xanthone | 121.2 µM (FCR3)  12.40 µM (3D7) | Plant | *Garcinia mangostana* (husk) | 156 |

2014

| 1 | Pellitorine (1) | Alkylamide | 14.6 µM (NF54) | Plant | *Achillea ptarmica* (flowering part) | 157 |
| --- | --- | --- | --- | --- | --- | --- |
| 2 | 8,9-*Z*-Dehydropellitorine | Alkylamide | 29.3 µM (NF54) | Plant | *Achillea ptarmica* (flowering part) | 157 |
| 3 | (E,E)-2,4- Dehydropellitorine | Alkylamide | 25.5 µM (NF54) | Plant | *Achillea ptarmica* (flowering part) | 157 |
| 4 | (E,E)-2,4-undecadien-8,10-diynoic acid pideridide (4) + (E,E)-2,4-undecadien-8,10-diynoic acid phenethylamide (5) (mixture ratio 3:1) | Alkylamide | 6.89 µg/ml (NF54) | Plant | *Achillea ptarmica* (flowering part) | 157 |
| 5 | Anacycline | Alkylamide | 26.7 µM (NF54) | Plant | *Achillea ptarmica* (flowering part) | 157 |
| 6 | Ammaniol | Tetrahydrofuran derivative | 22.6 µg/ml (NF54)  88.22 µM | Plant | *Ammannia multiflora* (whole plant) | 158 |
| 7 | 4-hydroxy-α-tetralone | Naphthoquinone | 31.5 µg/ml (NF54)  193.02 µM | Plant | *Ammannia multiflora and A. baccifera (whole plant)* | 158 |
| 8 | Tetralone-4-O-β-D-glucopyranoside | Naphthoquinone glycoside | 36.1 µg/ml (NF54)  111.01 µM | Plant | *Ammannia multiflora and A. baccifera* (whole plant) | 158 |
| 9 | Aphadilactone A (New) | Diterpene lactone | 190 nM (Dd2) |  | *Aphanamixis*  *Grandifolia* (leaf) | 159 |
| 10 | Aphadilactone B (New) | Diterpene lactone | 1350 nM (Dd2) |  | *Aphanamixis*  *Grandifolia* (leaf) | 159 |
| 11 | Aphadilactone C (New) | Diterpene lactone | 170 nM (Dd2) |  | *Aphanamixis*  *Grandifolia* (leaf) | 159 |
| 12 | Aphadilactone D (New) | Diterpene lactone | 120 nM (Dd2) |  | *Aphanamixis*  *Grandifolia* (leaf) | 159 |
| 13 | Aspidoscarpine | Indole alkaloid | 5.4 µg/ml (W2)  14.04 µM |  | *Aspidosperma olivaceum* (leaf) | 160 |
| 14 | Uleine | Indole alkaloid | 7.0 µg/ml (W2)  26.27 µM |  | *Aspidosperma olivaceum* (leaf) | 160 |
| 15 | Apparicine | Indole alkaloid | 3.0 µg/ml (W2)  11.34 µM |  | *Aspidosperma olivaceum* (leaf) | 160 |
| 16 | N-methyl-tetrahydroolivacine | Indole alkaloid | 5.7 µg/ml (W2)  21.56 µM |  | *Aspidosperma olivaceum* (leaf) | 160 |
| 17 | Ellipticine | Indole alkaloid | 0.81 µM (K1)  0.35 µM (3D7) |  | *Aspidosperma vargasii* (bark) | 161 |
| 18 | 2-methyl-1,2,3,4-tetrahydro  Ellipticine | Indole alkaloid | 4.2 µM (K1)  13 µM (3D7) |  | *Aspidosperma vargasii* (bark) | 161 |
| 19 | Bergenin | Phenolic glycoside | 2.41 µg/ml (D10)  7.34 µM |  | *Bergenia ligulata* (root) | 162 |
| 20 | 11-*O*-galloylbergenin | Phenolic glycoside | 2.34 µg/ml (D10)  4.87 µM |  | *Bergenia ligulata* (root) | 162 |
| 21 | *O*-tigloylcyclovirobuxeine-B | Cycloartane alkaloid | 0.91 µM (NF54) |  | *Buxus sempervirens* (aerial part) | 163 |
| 22 | Caesalminine A (New) | Cassane diterpene alkaloid | 0.42 µM (K1) |  | *Caesalpinia minax* (seed) | 164 |
| 23 | Caesalminine B (New) | Cassane diterpene alkaloid | 0.79 µM (K1) |  | *Caesalpinia minax (seed)* | 164 |
| 24 | 6α-acetoxyazadiradione | Limonoid | 15.4 µM (K1) |  | *Carapa guianensis* (seed) | 165 |
| 25 | Andirobin | Limonoid | 15.3 µM (K1) |  | *Carapa guianensis* (seed) | 165 |
| 26 | 6α-acetoxygedunin | Limonoid | 7.0 µM (K1)  65.7% inhibition of P. berghei parasite at 100mg/kg |  | *Carapa guianensis* (seed) | 165 |
| 27 | 7-deacetyl-7-oxogedunin | Limonoid | 20.7 µM (K1)  40.3% inhibition of P. berghei parasite at 100mg/kg |  | *Carapa guianensis* (seed) | 165 |
| 28 | Manghaslin | Flavonoid | >13.2 µM |  | *Carica papaya* (leaf) | 166 |
| 29 | Clitorin | Flavonoid | >13.5 µM |  | *Carica papaya* (leaf) | 166 |
| 30 | Rutin | Flavonoid | >16.4 µM |  | *Carica papaya* (leaf) | 166 |
| 31 | Nicotiflorin | Flavonoid | >16.8 µM |  | *Carica papaya* (leaf) | 166 |
| 32 | Carpamic acid | Piperidine alkaloid | >194.4 µM |  | *Carica papaya* (leaf) | 166 |
| 33 | Methyl carpamate | Piperidine alkaloid | >77.1 µM |  | *Carica papaya* (leaf) | 166 |
| 34 | Carpaine | Piperidine alkaloid | 0.2 µM  11.9% inhibition of P. berghei parasite at 5mg/kg |  | *Carica papaya* (leaf) | 166 |
| 35 | 6-(8-Methoxy-8-oxooctyl)-2-methylpiperidin-3-yl 8-(5-hydroxy- 6-methylpiperidin-2-yl)octanoate | Piperidine alkaloid | 1.85 µM |  | *Carica papaya* (leaf) | 166 |
| 36 | 13,26-Dimethyl-2,15-dioxa-12,25-diazatricyclo[22.2.2.211,14] triacontane-3,16-dione | Piperidine alkaloid | 1.0 µM |  | *Carica papaya* (leaf) | 166 |
| 37 | Quercetin-4’-methyl ether | Flavonoid | 81.48% suppression of P. berghei parasite at 2.5mg/kg |  | *Chromolaena odorata* (leaf) | 167 |
| 38 | (+)-Sebiferine | Morphinandienone | 22.46 µM (K1) |  | *Dehaasia longipedicellata* (bark) | 168 |
| 39 | (−)-Milonine | Morphinandienone | 0.097 µM (K1) |  | *Dehaasia longipedicellata* (bark) | 168 |
| 40 | (−)-Boldine | Aporphine | 2.602 µM (K1) |  | *Dehaasia longipedicellata* (bark) | 168 |
| 41 | (−)-Norboldine | Aporphine | 9.284 µM (K1) |  | *Dehaasia longipedicellata* (bark) | 168 |
| 42 | (−)-Reticuline | Benzylisoquinoline | <30.4 µM (K1) |  | *Dehaasia longipedicellata* (bark) | 168 |
| 43 | (−)-O-O-dimethylgrisabine | Benzylisoquinoline | 0.031 µM (K1) |  | *Dehaasia longipedicellata* (bark) | 168 |
| 44 | Drypetenone D (New) | Phenathrenone | 0.96 µM (NF54)  Not active in vivo |  | *Drypetes gerrardii* (stem) | 169 |
| 45 | Drypetenone E (New) | Phenathrenone | 2.04 µM (NF54) |  | *Drypetes gerrardii* (stem) | 169 |
| 46 | Phaseollidin | Pterocarpan | 1.66 µg/ml  5.12 µM |  | *Erythrina crista-galli* (bark) | 170 |
| 47 | Sandwicensin | Pterocarpan | 1.83 µg/ml  5.41 µM |  | *Erythrina crista-galli* (bark) | 170 |
| 48 | Lonchocarpol A | Flavonoid | 1.69 µg/ml  4.13 µM |  | *Erythrina crista-galli* (bark) | 170 |
| 49 | Guttiferone E | Benzophenone | 7.90 µM (D6)  7.47 µM (W2) |  | *Garcinia xanthochymus* (fruit) | 171 |
| 50 | Isoxanthochymol | Benzophenone | 6.97 µM (D6)  7.90 µM (W2) |  | *Garcinia xanthochymus* (fruit) | 171 |
| 51 | Guttiferone H | Benzophenone | 5.31 µM (D6)  5.31 µM (W2) |  | *Garcinia xanthochymus* (fruit) | 171 |
| 52 | α-Mangostin | Xanthone | 11.40 µM (D6)  10.20 µM (W2) |  | *Garcinia mangostana* (fruit) | 171 |
| 53 | β-Mangostin | Xanthone | 7.42 µM (D6)  4.21 µM (W2) |  | *Garcinia mangostana* (fruit) | 171 |
| 54 | 3-Isomangostin | Xanthone | 7.88 µM (D6)  6.15 µM (W2) |  | *Garcinia mangostana* (fruit) | 171 |
| 55 | Myristicyclin A (New) | Procyanidin | 54 µM |  | *Horsfieldia spicata* (whole plant) | 172 |
| 56 | Myristicyclin B (New) | Procyanidin | 7.9 µM |  | *Horsfieldia spicata* (whole plant) | 172 |
| 57 | 1-(2,6-Dihydroxyphenyl)-1-decanone | Aromatic aldehyde | 9.2 µM |  | *Horsfieldia spicata* (whole plant) | 172 |
| 58 | Chrysophanol | Anthraquinone | 58 µM (Dd2) |  | *Kniphofia ensifolia* (whole plant) | 173 |
| 59 | aloe-emodin | Anthraquinone | 55 µM (Dd2) |  | *Kniphofia ensifolia* (whole plant) | 173 |
| 60 | Kniphofione A (New) | Anthraquinone | 26 µM (Dd2) |  | *Kniphofia ensifolia* (whole plant) | 173 |
| 61 | Kniphofione B (New) | Anthraquinone | 9.0 µM (Dd2) |  | *Kniphofia ensifolia* (whole plant) | 173 |
| 62 | Knipholone | Anthraquinone | 1.1 µM (Dd2) |  | *Kniphofia ensifolia* (whole plant) | 173 |
| 63 | 10-(chrysophanol-7’-yl)-10-  hydroxychrysophanol-9-anthrone | Anthraquinone | 0.4 µM (Dd2) |  | *Kniphofia ensifolia* (whole plant) | 173 |
| 64 | Chryslandicin | Anthraquinone | 0.2 µM (Dd2) |  | *Kniphofia ensifolia* (whole plant) | 173 |
| 65 | Asphodeline | Anthraquinone | 10 µM (Dd2) |  | *Kniphofia ensifolia* (whole plant) | 173 |
| 66 | Microcarpin | Anthraquinone | 10 µM (Dd2) |  | *Kniphofia ensifolia* (whole plant) | 173 |
| 67 | 1-(5,7-Dihydroxy-2,2-dimethylchroman-6-yl)-3-(2,2-dimethylchroman-6-yl)propan-1-one (New) | Chalcone | 14.4 µM (3D7)  11.7 µM (Dd2)  6.7 µM (W2) |  | *Metrodorea stipularis* (stem) | 174 |
| 68 | 1-(5,7-Dihydroxy-2,2 -dimethylchroman-6-yl)-3-(1,1,4a-trimethyl-2,3,4,4a, 9a-hexahydro-1H-xanthen-7-yl)propan-1-one (New) | Chalcone | 17.7 µM (3D7)  12.4 µM (Dd2)  8.5 µM (W2) |  | *Metrodorea stipularis* (stem) | 174 |
| 69 | Myristic acid | Fatty acid | 10.5 µg/ml (3D7)  45.97 µM  82.6% suppression of P. berghei parasite at 100mg/kg |  | *Murraya koenigii* (leaf) | 175 |
| 70 | β-caryophyllene | Sesquiterpene | 8.25 µg/ml (3D7)  40.36 µM  88.2% suppression of P.berghei parasite at 100mg/kg |  | *Murraya koenigii* (leaf) | 175 |
| 71 | Neomacrolactone (New) | Cycloartane triterpene | 1.1 µg/ml (FcB1)  2.08 µM |  | *Neoboutonia macrocalyx* (leaf) | 176 |
| 72 | Acetoxyneomacrolactone (New) | Cycloartane triterpene | 1.4 µg/ml (FcB1)  2.46 µM |  | *Neoboutonia macrocalyx* (leaf) | 176 |
| 73 | Hydroxyneomacolactone (New) | Cycloartane triterpene | 0.8 µg/ml (FcB1)  1.47 µM |  | *Neoboutonia macrocalyx* (leaf) | 176 |
| 74 | α-Acetoxy-6-hydroxyneomacro  lactone (New) | Cycloartane triterpene | 1.6 µg/ml (FcB1)  2.73 µM |  | *Neoboutonia macrocalyx* (leaf) | 176 |
| 75 | Epoxyneomacrolactone (New) | Cycloartane triterpene | 5.1 µg/ml (FcB1)  9.43 µM |  | *Neoboutonia macrocalyx* (leaf) | 176 |
| 76 | α-Acetoxy-6,7-epoxyneomacro  lactone (New) | Cycloartane triterpene | 6.4 µg/ml (FcB1)  10.99 µM |  | *Neoboutonia macrocalyx* (leaf) | 176 |
| 77 | Methylen-neomacrolactone (New) | Cycloartane triterpene | 1.0 µg/ml (FcB1)  1.90 µM |  | *Neoboutonia macrocalyx* (leaf) | 176 |
| 78 | Neomacroin (New) | Cycloartane triterpene | 1.7 µg/ml (FcB1)  3.21 µM |  | *Neoboutonia macrocalyx* (leaf) | 176 |
| 79 | Neomacrotriol (New) | Cycloartane triterpene | >10 µg/ml (FcB1)  >21.16 µM |  | *Neoboutonia macrocalyx* (leaf) | 176 |
| 80 | Neonthrene(New) | phenanthrene | 9.8 µg/ml (FcB1)  36.28 µM |  | *Neoboutonia macrocalyx* (leaf) | 176 |
| 81 | 22-de-O-acetyl-26-deoxyneobouto  mellerone | Cycloartane triterpene | 1.1 µg/ml (FcB1)  2.15 µM |  | *Neoboutonia macrocalyx* (leaf) | 176 |
| 82 | Mellerin B | Diterpene | 9.6 µg/ml (FcB1)  19.04 µM |  | *Neoboutonia macrocalyx* (leaf) | 176 |
| 83 | 6-hydroxystigmast-4-en-3-one | Sterol | >10 µg/ml (FcB1)  >23.34 µM |  | *Neoboutonia macrocalyx* (leaf) | 176 |
| 84 | 5-*O*-methyl-5-epiisogoyazensolide (New) | Sesquiterpene lactone | 6.2 µM (Dd2) |  | *Piptocoma antillana* (leaf and twig) | 177 |
| 85 | 15-*O*-methylgoyazensolide(New) | Sesquiterpene lactone | 2.2 µM (Dd2) |  | *Piptocoma antillana* (leaf and twig) | 177 |
| 86 | 1-oxo-3,10-epoxy-8-(2-methylacryloxy)-15-acetoxygermacra-2,4,11(13)-trien-6(12)-olide | Sesquiterpene lactone | 8.0 µM (Dd2) |  | *Piptocoma antillana* (leaf and twig) | 177 |
| 87 | 5-epiisogoyazensolide | Sesquiterpene lactone | 9.0 µM (Dd2) |  | *Piptocoma antillana* (leaf and twig) | 177 |
| 88 | 5,6-didehydro-7-hydroxy-taxodone | Abietane diterpene | 9.2 µM (K1) |  | *Plectranthus barbatus* (aerial parts) | 178 |
| 89 | Taxodione | Abietane diterpene | 8.5 µM (K1) |  | *Plectranthus barbatus* (aerial parts) | 178 |
| 90 | 20-deoxocarnosol | Abietane diterpene | 11.1 µM (K1) |  | *Plectranthus barbatus* (aerial parts) | 178 |
| 91 | 6α,11,12,-trihydroxy-7β,20-epoxy-8,11,13-abietatriene | Abietane diterpene | 31.6 µM (K1) |  | *Plectranthus barbatus* (aerial parts) | 178 |
| 92 | Plumbagin | Naphthoquinone | 580 nM (3D)  370 nM (K1)  41% suppression of P. berghei parasite at 25mg/kg |  |  | 179 |
| 93 | Ferruginol | Abietane diterpene | 2.47 µM (3D7)  1.33 µM (K1)  Synthetically modified derivative showed improved activity and selectivity |  | *Podocarpus ferruginea* | 180 |
| 94 | N-acetyl-8a-polyveolinone (New) | Indolosesquiterpene alkaloid | 7.6 µM (NF54) |  | *Polyalthia oliveri* (stem bark) | 181 |
| 95 | N-acetyl-polyveoline (New) | Indolosesquiterpene alkaloid | 29.1 µM (NF54) |  | *Polyalthia oliveri* (stem bark) | 181 |
| 96 | (–)-cassine | Piperidine alkaloid | 1.82 µM (3D7) |  | *Senna spectabilis* (leaf) | 182 |
| 97 | (–)-spectaline | Piperidine alkaloid | 2.76 µM (3D7) |  | *Senna spectabilis* (leaf) | 182 |
| 98 | Strychnobaillonine (New) | Indole alkaloid | 1.1 µM (3D7) |  | *Strychnos icaja* (root) | 183 |
| 99 | Strychnohexamine | Indole alkaloid | 0.6 µM (3D7) |  | *Strychnos icaja* (root) | 183 |
| 100 | Quercetin-3-methyl ether | Flavonoid | 4.37 µg/ml (D6)  13.81 µM |  | *Tagetes minuta* (leaf) | 184 |
| 101 | Nitidine | Benzophenanthridine alkaloid | 0.077 µg/ml (3D7)  0.22 µM  0.027 µg/ml (F32)  0.077 µM |  | *Zanthoxylum chalybeum* (root bark) | 185 |
| 102 | Methyl canadine | Isoquinoline alkaloid | 2.01 µg/ml (3D7)  5.67 µM |  | *Zanthoxylum chalybeum* (root bark) | 185 |
| 103 | Chelerythrine | Benzophenanthridine alkaloid | 1.35 µg/ml (3D7)  3.87 µM |  | *Zanthoxylum chalybeum* (root bark) | 185 |
| 104 | Tembetarin | Benzylisoquinoline alkaloid | 2.01 µg/ml (3D7)  4.26 µM |  | *Zanthoxylum chalybeum* (root bark) | 185 |
| 105 |  |  | 3.66 µg/ml (K1) |  | *Toddalia asiatica* (stem bark) | 186 |
| 106 | Turranoic acid (New) | Multiflorane triterpene | 5.2 µM (FCM29) |  | *Turraea sp.* (leaf) | 187 |
| 107 | Turraenine (New) | Multiflorane triterpene | 16.6 µM (FCM29) |  | *Turraea sp.* (leaf) | 187 |
| 108 | Triptocallic acid B | Multiflorane triterpene | 16.4 µM (FCM29) |  | *Turraea sp.* (leaf) | 187 |
| 109 | Normelicopidine | Acridone alkaloid | 18.9 µg/ml (Dd2)  63.19 µM |  | *Zanthoxylum simullans* (root bark) | 188 |
| 110 | Normelicopine | Acridone alkaloid | 25.9 µg/ml (Dd2)  86.54 µM  31.2 µg/ml (3D7)  104.25 µM |  | *Zanthoxylum simullans* (root bark) | 188 |
| 111 | Melicopine | Acridone alkaloid | 33.7 µg/ml (Dd2)  107.56 µM  29.7 µg/ml (3D7)  94.79 µM |  | *Zanthoxylum simullans* (root bark) | 188 |
| 112 | Melicopidine | Acridone alkaloid | 21.6 µg/ml (Dd2)  68.94 µM  25.5 µg/ml (3D7)  81.39 µM |  | *Zanthoxylum simullans* (root bark) | 188 |
| 113 | Melicopicine | Acridone alkaloid | 41.2 µg/ml (Dd2)  125.09 µM  37.7 µg/ml (3D7)  114.46 µM |  | *Zanthoxylum simullans* (root bark) | 188 |
| 114 | Netamine K (New) | Pyrimidine alkaloid | 2.4 µM | Marine sponge | *Biemna Laboutei* | 189 |
| 115 | Mirabilin A | Pyrimidine alkaloid | 20.7 µM | Marine sponge | *Biemna laboutei* | 189 |
| 116 | Diacarperoxide H (New) | Norditerpene cyclic peroxide | 12.9 µM (W2) | Marine sponge | *Diacarnus megaspinorhabdosa* | 190 |
| 117 | Diacarperoxide I (New) | Norditerpene cyclic peroxide | 4.8 µM (W2)  7.9 µM (D6) | Marine sponge | *Diacarnus megaspinorhabdosa* | 190 |
| 118 | Diacarperoxide J (New) | Norditerpene cyclic peroxide | 1.8 µM (W2)  1.6 µM (D6) | Marine sponge | *Diacarnus megaspinorhabdosa* | 190 |
| 119 | Ganoboninketal A (New) | Norlanostane-triterpene | 4.0 µM (3D7) | Mushroom | *Ganoderma boninense* | 191 |
| 120 | Ganoboninketal B (New) | Norlanostane-triterpene | 7.9 µM (3D7) | Mushroom | *Ganoderma boninense* | 191 |
| 121 | Ganoboninketal C (New) | Norlanostane-triterpene | 1.7 µM (3D7) | Mushroom | *Ganoderma boninense* | 191 |
| 122 | Ganodermalactone F (New) | Triterpene Lactone | 10.0 µM (K1) | Fungus | *Ganoderma* sp. KM01 | 192 |
| 123 | Schisanlactone B | Triterpene Lactone | 6.0 µM (K1) | Fungus | *Ganoderma* sp. KM01 | 192 |
| 124 | Colossolactone E | Triterpene Lactone | 10.0 µM (K1) | Fungus | *Ganoderma* sp. KM01 | 192 |
| 125 | Calvasterol A | Sterol | 3.4 µg/ml (K1)  8.05 µM | Fungus | *Gymnoascus reessii* | 193 |
| 126 | Calvasterol B | Sterol | 3.4 µg/ml (K1)  7.72 µM | Fungus | *Gymnoascus reessii* | 193 |
| 127 | ergosta-4-6,8(14),22-tetraene-3-one | Sterol | 4.5 µg/ml (K1)  11.47 µM | Fungus | *Gymnoascus reessii* | 193 |
| 128 | (22E,24R)-ergosta-4,7,22-triene-3-one | Sterol | 3.3 µg/ml (K1)  8.36 µM | Fungus | *Gymnoascus reessii* | 193 |
| 129 | 24(R)-5α,8α-epidioxyergosta-6-22-diene-3β-ol | Sterol | 4.1 µg/ml (K1)  9.56 µM | Fungus | *Gymnoascus reessii* | 193 |
| 130 | (1-O-[(E)-2’,2’,4-trimethyl-3’-oxohexadec-4’-enoyl]-sn-glycero-3-O-4’’-(N,N,N-trimethyl)-L-homoserine (MDN-0104) (New) | Betaine lipid | 7.0 µM (3D7) | Fungus | *Heterospora*  *Chenopodii* | 194 |
| 131 | Aszonapyrone A | Meroterpenoid | 1.34 µg/ml (K1)  2.93 µM | Fungus | *Neosartorya tatenoi* KKU-2NK23 | 195 |
| 132 | Pilobolusone A (New) | Depsidone | 5.35 µM (K1) | Fungus | *Pilobolus heterosporus* | 196 |
| 133 | Pilobolusone D (New) | Depsidone | 17.95 µM (K1) | Fungus | *Pilobolus heterosporus* | 196 |
| 134 | 11H-dibenzo[b,e][1, 4]dioxepin-4-  carboxaldehyde,3,8-dihydroxy-9-methyl-1,6-bis[(1E)-1-methyl-1-propen-1-yl]-11-oxo | Depsidone | 3.67 µM (K1) | Fungus | *Pilobolus heterosporus* | 196 |
| 135 | 11H-dibenzo[b,e][1,4]dioxepin-11-one,3,8-dihydroxy-4-(methoxy  methyl)-9-methyl-1,6-bis[(1E)-1-methyl-1-propen-1-yl | Depsidone | 23.56 µM (K1) | Fungus | *Pilobolus heterosporus* | 196 |
| 136 | polyketide endoperoxide 1 | Cyclic peroxide | 3.89 µM (D10)  2.91 µM (W2) | Marine sponge | *Plakortis simplex* | 197 |
| 137 | polyketide endoperoxide 2 (New) | Cyclic peroxide | 4.05 µM (D10)  2.70 µM (W2) | Marine sponge | *Plakortis simplex* | 197 |
| 138 | polyketide endoperoxide 3 | Cyclic peroxide | 1.77 µM (D10)  1.56 µM (W2) | Marine sponge | *Plakortis simplex* | 197 |
| 139 | polyketide endoperoxide 5 (New) | Cyclic peroxide | 6.18 µM (D10)  4.98 µM (W2) | Marine sponge | *Plakortis simplex* | 197 |
| 140 | polyketide endoperoxide 6 (New) | Cyclic peroxide | 11.4 µM (W2) | Marine sponge | *Plakortis simplex* | 197 |
| 141 | polyketide endoperoxide 7 (New) | Cyclic peroxide | 5.12 µM (D10)  4.10 µM (W2) | Marine sponge | *Plakortis simplex* | 197 |
| 142 | Preussiafuran A (New) | Dibenzofuran | 8.76 µM (NF54) | Fungus | *Preussia sp.* | 198 |
| 143 | Preussiafuran B (New) | Dibenzofuran | 15.0 µM (NF54) | Fungus | *Preussia sp.* | 198 |
| 144 | Cissetin |  | 10.3 µM (NF54) | Fungus | *Preussia sp.* | 198 |
| 145 | Asterric acid |  | 8.67 µM (NF54) | Fungus | *Preussia sp.* | 198 |
| 146 | Mollemycin A (New) | Glycol-hexadepsipeptide-polyketide | 7 nM (3D7)  9 nM (Dd2) | actinobacteria | *Streptomyces sp.* (CMBM0244) | 199 |

2013

| 1 | Abruquinone H (New) | Isoflavan hydroquinone | 8.0 µM (K1) | Plant | *Abrus precatorius ssp. Africanus* (whole plant) | 200 |
| --- | --- | --- | --- | --- | --- | --- |
| 2 | Abruquinone I (New) | Isoflavan quinone | 20.4 µM (K1) | Plant | *Abrus precatorius ssp. Africanus* (whole plant) | 200 |
| 3 | Abruquinone B | Isoflavan quinone | 4.1 µM (K1) | Plant | *Abrus precatorius ssp. Africanus* (whole plant) | 200 |
| 4 | 7,8,3’,5’-tetrameth  oxyisoflavan-1’,4’-quinone | Isoflavan quinone | 8.9 µM (K1) | Plant | *Abrus precatorius ssp. Africanus* (whole plant) | 200 |
| 5 | Mbandakamine A (New) | Naphthylisoquinoline alkaloid | 0.13 µM (NF54)  (Dicetate salt 0.043 µM NF54) |  | *Ancistrocladus Sp.* (leaf) | 201 |
| 6 | Mbandakamine B (New) | Naphthylisoquinoline alkaloid | (diacetate salt 0.14 µM NF54) |  | *Ancistrocladus Sp.* (leaf) | 201 |
| 7 | Ancistectorine A1 (New) | Naphthylisoquinoline alkaloid | 0.57 µM (K1) |  | *Ancistrocladus tectorius* (twig) | 202 |
| 8 | N-Methylancistect  orine A1 (New) | Naphthylisoquinoline alkaloid | 0.08 µM (K1) |  | *Ancistrocladus tectorius* (twig) | 202 |
| 9 | Ancistectorine A2 (New) | Naphthylisoquinoline alkaloid | 0.07 µM (K1) |  | *Ancistrocladus tectorius* (twig) | 202 |
| 10 | 5-epi-Ancistectorine A2 (New) | Naphthylisoquinoline alkaloid | 0.03 µM (K1) |  | *Ancistrocladus tectorius* (twig) | 202 |
| 11 | Ancistectorine A3 (New) | Naphthylisoquinoline alkaloid | 0.68 µM (K1) |  | *Ancistrocladus tectorius* (twig) | 202 |
| 12 | Ancistectorine B1 (New) | Naphthylisoquinoline alkaloid | 4.20 µM (K1) |  | *Ancistrocladus tectorius* (twig) | 202 |
| 13 | Ancistectorine C1 (New) | Naphthylisoquinoline alkaloid | 2.80 µM (K1) |  | *Ancistrocladus tectorius* (twig) | 202 |
| 14 | 3,4,5,6-tetradehydro  -β-yohimbine | Indole alkaloid | 39.9 µM (K1) |  | *Aspidosperma ulei* (leaf) | 203 |
| 15 | 20-*epi*-dasycarpid  One | Indole alkaloid | 16.7 µM (K1) |  | *Aspidosperma ulei* (root bart) | 203 |
| 16 | 20(*E*)-*nor*-subincana  dine E | Indole alkaloid | 54.3 µM (K1) |  | *Aspidosperma ulei* (stem bark) | 203 |
| 17 | Cryptobeilic acid A (New) | Beilschmiedic acid derivative | 17.7 µM (NF54) |  | *Beilschmiedia cryptocaryoides* (bark) | 204 |
| 18 | Cryptobeilic acid B (New) | Beilschmiedic acid derivative | 5.35 µM (NF54) |  | *Beilschmiedia cryptocaryoides* (bark) | 204 |
| 19 | Cryptobeilic acid C (New) | Beilschmiedic acid derivative | 14 µM (NF54) |  | *Beilschmiedia cryptocaryoides* (bark) | 204 |
| 20 | Cryptobeilic acid D (New) | Beilschmiedic acid derivative | 10.8 µM (NF54) |  | *Beilschmiedia cryptocaryoides* (bark) | 204 |
| 21 | Tsangibeilin B | Endiandric acid derivative | 8.2 µM (NF54) |  | *Beilschmiedia cryptocaryoides* (bark) | 204 |
| 22 | Voulkensin C (New) | Cassane diterpene | 11.63 µM (D6)  16.53 µM (W2) |  | *Caesalpinia volkensii* (stem bark) | 205 |
| 23 | Voulkensin D (New) | Cassane diterpene | 17.26 µM (D6)  22.79 µM (W2) |  | *Caesalpinia volkensii* (stem bark) | 205 |
| 24 | Voulkensin E (New) | Cassane diterpene | 18.69 µM (D6)  15.57 µM (W2) |  | *Caesalpinia volkensii* (stem bark) | 205 |
| 25 | 3-β-Glu-3-xyl-stigmasterol (New) | Strerol | 4.44 µM (D6)  2.74 µM (W2) |  | *Caesalpinia volkensii* (stem bark) | 205 |
| 26 | Oleanolic acid | Triterpene | 23.81 µM (D6)  26.24 µM (W2) |  | *Caesalpinia volkensii* (stem bark) | 205 |
| 27 | 3-β-Acetylolean-12-en-28-methyl ester | Triterpene | 18.69 µM (D6)  24.44 µM (W2) |  | *Caesalpinia volkensii* (stem bark) | 205 |
| 28 | Voucapan-5-ol | Cassane diterpene | >50 µM (D6)  >50 µM (W2) |  | *Caesalpinia volkensii* (stem bark) | 205 |
| 29 | Caesaldekarin C | Cassane diterpene | 34.44 µM (D6)  30.69 µM (W2) |  | *Caesalpinia volkensii* (stem bark) | 205 |
| 30 | Deoxycaesaldekarin C | Cassane diterpene | 25.67 µM (D6)  30.33 µM (W2) |  | *Caesalpinia volkensii* (stem bark) | 205 |
| 31 | Cajachalcone | Flavonoid | 7.4 µM (K1) |  | *Cajanus cajan* (leaf) | 206 |
| 32 | Garjasmine | Iridoid | 38.5 µg/ml (K1)  171.71 µM |  | *Canthium Multiflorum* (aerial part) | 207 |
| 33 | Gardenine | Alkaloid | 7.17 µg/ml (K1)  32.12 µM |  | *Canthium Multiflorum* (aerial part) | 207 |
| 34 | Deacetyl asperulos  idic acid | Iridoid | >50 µg/ml (K1)  >128.09 µM |  | *Canthium Multiflorum* (aerial part) | 207 |
| 35 | Griffinoid C (New) | Flavonoid | 15.74 µM (K1) |  | *Combretum griffithii* (stem) | 208 |
| 36 | Griffinoid D (New) | Flavonoid | 13.04 µM (K1) |  | *Combretum griffithii* (stem) | 208 |
| 37 | Griffithane E (New) | Flavonoid | 9.66 µM (K1) |  | *Combretum griffithii* (stem) | 208 |
| 38 | 1-(4-hydroxy-3,5-dimethoxyphenyl)-3-  (4-hydroxy-3-metho  xyphenyl)propane | Diaryl propanone | 14.45 µM (K1) |  | *Combretum griffithii* (stem) | 208 |
| 39 | Watsonianone A (New) | β-triketone | 8.8 µM (Dd2)  5.3 µM (3D7) |  | *Corymbia watsoniana* (flower) | 209 |
| 40 | Watsonianone B (New) | β-triketone | 0.44 µM (Dd2)  0.29 µM (3D7) |  | *Corymbia watsoniana* (flower) | 209 |
| 41 | Watsonianone C (New) | β-triketone | 1.18 µM (Dd2)  1.07 µM (3D7) |  | *Corymbia watsoniana* (flower) | 209 |
| 42 | (+)-*N*-methylisococlaurine | Benzylisoquinoline alkaloid | 5.4 µM (K1) |  | *Cryptocarya nigra* (bark) | 210 |
| 43 | Atherosperminine | Phenanthrene  alkaloids | 5.8 µM (K1) |  | *Cryptocarya nigra* (bark) | 210 |
| 44 | 2-hydroxyatherosper  minine | Phenanthrene  alkaloids | 0.75 µM (K1) |  | *Cryptocarya nigra* (bark) | 210 |
| 45 | Quercitrin | Flavonoid glycoside | 5.96 µg/ml (3D7)  13.29 µM  2.26 µg/ml (Dd2)  5.04 µM |  | *Dacryodes edulis* (stem bark) | 211 |
| 46 | Afzelin | Flavonoid glycoside | 4.59 µg/ml (3D7)  10.61 µM  19.34 µg/ml (Dd2)  44.72 µM |  | *Dacryodes edulis* (stem bark) | 211 |
| 47 | Quercetin | Flavonoid | 6.07 µg/ml (3D7)  20.08 µM  5.91 µg/ml (Dd2)  19.55 µM |  | *Dacryodes edulis* (stem bark) | 211 |
| 48 | Methyl-3,4,5-trihyd  roxybenzoate (Methyl gallate) | Phenolic | 0.37 µg/ml (3D7)  2.00 µM  0.55 µg/ml (Dd2)  2.98 µM |  | *Dacryodes edulis* (stem bark) | 211 |
| 49 | Datiscoside | Cucurbitacin triterpene glycoside | 13.5 µM (D10) |  | *Datisca glomerata* (aerial part) | 212 |
| 50 | Datiscoside I (New) | Cucurbitacin triterpene glycoside | 16.0 µM (D10) |  | *Datisca glomerata* (aerial part) | 212 |
| 51 | Datiscoside B | Cucurbitacin triterpene glycoside | 14.5 µM (D10) |  | *Datisca glomerata* (aerial part) | 212 |
| 52 | Datiscoside J (New) | Cucurbitacin triterpene glycoside | 16.3 µM (D10) |  | *Datisca glomerata* (aerial part) | 212 |
| 53 | Datiscoside K (New) | Cucurbitacin triterpene glycoside | 7.7 µM (D10) |  | *Datisca glomerata* (aerial part) | 212 |
| 54 | Datiscoside L (New) | Cucurbitacin triterpene glycoside | 12.7 µM (D10) |  | *Datisca glomerata* (aerial part) | 212 |
| 55 | Datiscoside M (New) | Cucurbitacin triterpene glycoside | 33.3 µM (D10) |  | *Datisca glomerata* (aerial part) | 212 |
| 56 | Datiscoside N (New) | Cucurbitacin triterpene glycoside | 8.8 µM (D10) |  | *Datisca glomerata* (aerial part) | 212 |
| 57 | Datiscoside O (New) | Cucurbitacin triterpene glycoside | 27.8 µM (D10) |  | *Datisca glomerata* (aerial part) | 212 |
| 58 | Parvifloral B (New) | Trinorcadalene naphthalene | 11.45 µM (K1) |  | *Decaschistia parviflora* (root) | 213 |
| 59 | Parvifloral F (New) | Trinorcadalene naphthalene | 6.85 µM (K1) |  | *Decaschistia parviflora* (root) | 213 |
| 60 | 1-β-(p-methoxycin  namoyl)-polygodial | Drimane sesquiterpene | 4.87 µM (FcR3) |  | *Drimys brasilensis* (stem bark) | 214 |
| 61 | 1-β-(p-cumaroyl  oxyl)-polygodial | Drimane sesquiterpene | 1.01 µM (FcR3) |  | *Drimys brasilensis* (stem bark) | 214 |
| 62 | Chamaejasmin | Flavonoid | 2.32 µg/ml  3.96 µM |  | *Enkleia siamensis* (root) | 215 |
| 63 | Euojaposphingoside A (New) | Glycosylsphingolipid | 44.3 µM (D10) |  | *Euonymus japonicas* (stem bark) | 216 |
| 64 | Euojaposphingoside B (New) | Glycosylsphingolipid | 38.3 µM (D10) |  | *Euonymus japonicas* (stem bark) | 216 |
| 65 | 1-O-[β-D-glucopyran  osyl]-(2S,3R,9E)-3-hydroxymethyl-2-N-[(2R)-hydroxynon  acosanoyl)-trideca  sphinga-9-ene | Glycosylsphingolipid | 52.3 µM (D10) |  | *Euonymus japonicas* (stem bark) | 216 |
| 66 | 1-O-[β-D-glucopyran  osyl]-(2S,3R,9E,12E)-2-N-[(2R)-hydroxy  tetracosanoyl]octa  decasphinga-9,12–diene | Glycosylsphingolipid | 34.3 µM (D10) |  | *Euonymus japonicas* (stem bark) | 216 |
| 67 | 1-O-[β-D-glucopyran  osyl]-(2S,3R,5R,9E)-2-N-[tridecanoyl]non  acosasphinga-9-ene | Glycosylsphingolipid | 21.3 µM (D10) |  | *Euonymus japonicas* (stem bark) | 216 |
| 68 | β-carboline-1-propio  nic acid | β-carboline alkaloid | 76.73 nM (TD7) |  | *Eurycoma longifolia* (root) | 217 |
| 69 | Eurycomanone | Quassinoid | 2.04 nM (TD7) |  | *Eurycoma longifolia* (root) | 217 |
| 70 | 18-dehydro-6α-hydroxyeurycomalactone (New) | Quassinoid | 119.12 nM (TD7) |  | *Eurycoma longifolia* (root) | 217 |
| 71 | Eurycomanol | Quassinoid | 171.39 nM (TD7) |  | *Eurycoma longifolia* (root) | 217 |
| 72 | 2-(2-benzoyl-β-D-glucopyranosyloxy)-7-(1α,2α,6α-trihydr  oxy-3-oxocyclohex-  4-enoyl)-5-hydroxy  benzyl alcohol (New) | Phenolic glycoside | 4.4 µM (3D7) |  | *Flacourtia indica* (leaf and twig) | 218 |
| 73 | Poliothrysoside | Phenolic glycoside | 8.1 µM (3D7) |  | *Flacourtia indica* (leaf and twig) | 218 |
| 74 | Catechin-[5,6-e]-4β-(3,4-dihydroxy  phenyl)dihydro-2(3H)-pyranone | Flavonoid | 1.1 µM (3D7) |  | *Flacourtia indica* (leaf and twig) | 218 |
| 75 | 2-(6-benzoyl-β-D-glucopyranosyloxy)-7-(1α,2α,6α-trihydroxy-3-oxocyc  lohex-4-enoyl)-5-hydroxybenzyl alcohol | Phenolic glycoside | 3.6 µM (3D7) |  | *Flacourtia indica* (leaf and twig) | 218 |
| 76 | Chrysoeriol-7-O-b-D-glucopyranoside | Flavonoid glycoside | 4.4 µM (3D7) |  | *Flacourtia indica* (leaf and twig) | 218 |
| 77 | Mururin A | Flavonoid | 1.2 µM (3D7)  1.3 µM (K1) |  | *Flacourtia indica* (leaf and twig) | 218 |
| 78 | Goniothaline A (New) | Pyridocoumarin alkaloid | >50 µM (3D7) |  | *Goniothalamus australis* (aerial part) | 219 |
| 79 | Goniothaline B (New) | Pyridocoumarin alkaloid | >50 µM (3D7) |  | *Goniothalamus australis* (aerial part) | 219 |
| 80 | Aristolactam AII | Aristolactam alkaloid | 28 µM (3D7) |  | *Goniothalamus australis* (aerial part) | 219 |
| 81 | Enterocarpam II | Aristolactam alkaloid | >50 µM (3D7) |  | *Goniothalamus australis* (aerial part) | 219 |
| 82 | Caldensine | Aristolactam alkaloid | 25 µM (3D7) |  | *Goniothalamus australis* (aerial part) | 219 |
| 83 | Sauristolactam | Aristolactam alkaloid | 9 µM (3D7) |  | *Goniothalamus australis* (aerial part) | 219 |
| 84 | (-)-anonaine | aporphine alkaloid | 7 µM (3D7) |  | *Goniothalamus australis* (aerial part) | 219 |
| 85 | Asimilobine | aporphine alkaloid | 22 µM (3D7) |  | *Goniothalamus australis* (aerial part) | 219 |
| 86 | Altholactone | Styryllactone | >50 µM (3D7) |  | *Goniothalamus australis* (aerial part) | 219 |
| 87 | (+)-goniofufurone | Styryllactone | >50 µM (3D7) |  | *Goniothalamus australis* (aerial part) | 219 |
| 88 | Conessine | Steroidal alkaloid | 1.3 µg/ml (K1)  3.64 µM  88.95% suppression of parasite in P. berghei infected mice at 10mg/kg |  | *Holarrhena antidysenterica* (bark) | 220 |
| 89 | Jatrophone | Diterpene | <8.09 µM (3D7)  <4.05 µM (K1)  Synthetic analogue showed better therapeutic window. |  | *Jatropha isabelli* | 221 |
| 90 | Grandifolione | Limonoid | 1.39 µM (K1) |  | *Khaya anthotheca* (seed) | 222 |
| 91 | 7-deacetylkhivorin | Limonoid | 2.52 µM (K1) |  | *Khaya anthotheca* (seed) | 222 |
| 92 | Knipholone cyclooxanthrone (New) | Phenyl anthrone | 6.13 µg/ml (W2)  14.66 µM  3.96 µg/ml (D6)  9.47 µM |  | *Kniphofia foliosa* (root) | 223 |
| 93 | 10-methoxy-10,7’-(chrysophanol anth  rone)-chrysophanol (New) | Anthraquinone | 1.17 µg/ml (W2)  2.24 µM  4.07 µg/ml (D6)  7.79 µM |  | *Kniphofia foliosa* (root) | 223 |
| 94 | Dianellin | Naphthalene glycoside | 3.28 µg/ml (W2)  6.25 µM  5.47 µg/ml (D6)  10.43 µM |  | *Kniphofia foliosa* (root) | 223 |
| 95 | Lippialactone (New) | α-Pyrone | 9.1 µg/ml (D10)  24.72 µM |  | *Lippia javanica* (aerial part) | 224 |
| 96 | (+)-5,6-dehydro  lycorine (New) | Phenanthridine alkaloid | 2.3 µM (D6)  1.9 µM (W2) |  | *Lycoris radiate* (bulbs) | 225 |
| 97 | (+)-3α,6β-diacetyl-bulbispermine (New) | Phenanthridine alkaloid | 18.9 µM (D6)  20.1 µM (W2) |  | *Lycoris radiate* (bulbs) | 225 |
| 98 | (+)-3α-hydroxy-6β-acetylbulbispermine (New) | Phenanthridine alkaloid | 17.9 µM (D6)  19.3 µM (W2) |  | *Lycoris radiate* (bulbs) | 225 |
| 99 | (+)-3α-methoxy-6β  -acetylbulbispermine | Alkaloid | 21.3 µM (D6)  23.4 µM (W2) |  | *Lycoris radiate* (bulbs) | 225 |
| 100 | Mallotojaponin B (New) | Phenolic | 0.75 µM (Dd2) |  | *Mallotus oppositifolius* (leaf and inflorescence) | 226 |
| 101 | Mallotojaponin C (New) | Phenolic | 0.14 µM (Dd2) |  | *Mallotus oppositifolius* (leaf and inflorescence) | 226 |
| 102 | Simplicifolianine (New) | Protoberberine (benzylisoquinoline ) alkaloid | 0.78 µg/ml (TM4)  2.05 µM  1.29 µg/ml (K1CB1)  3.39 µM |  | *Meconopsis simplicifolia* (aerial part) | 227 |
| 103 | Protopine | Benzylisoquinolin alkaloid | 1.45 µg/ml (TM4)  4.10 µM  1.38 µg/ml (K1CB1)  3.90 µM |  | *Meconopsis simplicifolia* (aerial part) | 227 |
| 104 | Ursolic acid | Triterpene | 6.8 µg/ml (D10)  14.88 µM  Semisynthetic derivative showed 94.01% P. berghei parasite suppression in mice |  | *Mimusops caffra* (leaf) | 228 |
| 105 | Cupacinoside | Diterpene glycoside | 4.0 µM (Dd2) |  | *Molinaea retusa* (root) | 229 |
| 106 | 6-de-*O*-acetyl  Cupacinoside | Diterpene glycoside | 6.4 µM (Dd2) |  | *Molinaea retusa* (root) | 229 |
| 107 | 25-(acetyloxy)-2-(β-D-glucopyranosyloxy  )-3,16-dihydroxy-9-methyl-19-norlano  sta-5,23-dien-22-one | Triterpene glycoside | 8.3 µM (3D7) |  | *Picrorhiza scrophulariiflora* (rhizome) | 230 |
| 108 | 4-Nerolydilcathecol | Sesquiterpene | 1.8 µg/ml (D6)  5.72 µM |  | *Piper umbellate* (aerial part) | 231 |
| 109 | Taraxasterol acetate | Triterpene | 4.7 µg/ml (NF54)  10.02 µM  60.13% suppression of P. berghei parasite in mice at 100mg/kg |  | *Pluchea lanceolata* (aerial part) | 232 |
| 110 | Stachydrine | Pyrollidine alkaloid | 6.26 µg/ml  43.72 |  | *Ritchiea capparoides var. longipedicellata* (leaf) | 233 |
| 111 | Nepodin | Naphthalene | 0.74 µg/ml (3D7)  3.42 µM  0.79 µg/ml (S20)  3.65 µM  97.1% suppression of P. berghei parasite in mice at 10mg/kg |  | *Rumex crispus* (whole plant) | 234 |
| 112 | Salaterpene A (New) | Sesquiterpene | 2.02 µM (W2) |  | *Salacia longipes var. camerunensis* (seed) | 235 |
| 113 | Salaterpene B (New) | Sesquiterpene | 1.81 µM (W2) |  | *Salacia longipes var. camerunensis* (seed) | 235 |
| 114 | Salaterpene C (New) | Sesquiterpene | 2.63 µM (W2) |  | *Salacia longipes var. camerunensis* (seed) | 235 |
| 115 | Salaterpene D(New) | Sesquiterpene | 2.38 µM (W2) |  | *Salacia longipes var. camerunensis* (seed) | 235 |
| 116 | 2β-acetoxy-1α,6β,9β  -tribenzoyloxy-4β-hydroxy-dihydro-bagarofuran | Sesquiterpene | 1.71 µM (W2) |  | *Salacia longipes var. camerunensis* (seed) | 235 |
| 117 | 12-Deoxy-salvipisone | Abietane diterpene | 8.8 µM (K1) |  | *Salvia sahendica* (root) | 236 |
| 118 | Sahandinone | Abietane diterpene | 5.1 µM (K1) |  | *Salvia sahendica* (root) | 236 |
| 119 | 12-Deoxy-6,7-dehydroroyleanone | Abietane diterpene | 17.8 µM (K1) |  |  | 236 |
| 120 | Δ^9^-Ferruginol | Abietane diterpene | 0.9 µM (K1) |  | *Salvia sahendica* (root) | 236 |
| 121 | Ferruginol | Abietane diterpene | 0.9 µM (K1) |  | *Salvia sahendica* (root) | 236 |
| 122 | 7α-acetoxyroylean  One | Abietane diterpene | 1.3 µM (K1) |  | *Salvia sahendica* (root) | 236 |
| 123 | Sahandol (New) | Abietane diterpene | 4.7 µM (K1) |  | *Salvia sahendica* (root) | 236 |
| 124 | Sahandone | Abietane diterpene | 17.2 µM (K1) |  | *Salvia sahendica* (root) | 236 |
| 125 | Stephanine | Isoquinoline (aporphine) alkaloid | 4.2 µM (W2) |  | *Stephania rotunda* (tuber) | 237 |
| 126 | Vireakine (New) | Isoquinoline (aporphine) alkaloid | 8.8 µM (W2) |  | *Stephania rotunda* (tuber) | 237 |
| 127 | Tetrahydropalmatine | Isoquinoline alkaloid | 30.9 µM (W2) |  | *Stephania rotunda* (tuber) | 237 |
| 128 | Xylopinine | Isoquinoline alkaloid | 52.3 µM (W2) |  | *Stephania rotunda* (tuber) | 237 |
| 129 | Roemerine | Isoquinoline (aporphine) alkaloid | 17.9 µM (W2) |  | *Stephania rotunda* (tuber) | 237 |
| 130 | Cepharanthine | Isoquinoline alkaloid | 1.2 µM (W2) |  | *Stephania rotunda* (tuber) | 237 |
| 131 | Palmatine | Isoquinoline alkaloid | 3.0 µM (W2) |  | *Stephania rotunda* (tuber) | 237 |
| 132 | Pseudopalmatine | Isoquinoline alkaloid | 2.8 µM (W2) |  | *Stephania rotunda* (tuber) | 237 |
| 133 | Fimbricalyx B (New) | Phenanthrone | 0.019 µM (K1) |  | *Strophioblachia f imbricalyx* (root) | 238 |
| 134 | Fimbricalyxanhydride A | Phenanthrone | 3.9 µM (K1) |  | *Strophioblachia f imbricalyx* (root) | 238 |
| 135 | Fimbricalyx A | Phenanthrone | 2.9 µM (K1) |  | *Strophioblachia f imbricalyx* (root) | 238 |
| 136 | Strychnochrysine | Bisindole alkaloid | 8.5 µM (3D7)  10.3 µM (W2) |  | *Strychnos nux-vomica*  (stem bark suber powder) | 239 |
| 137 | Demethoxyguiaflavine (New) | Bisindole alkaloid | 13.6 µM (3D7)  12.7 µM (W2) |  | *Strychnos nux-vomica*  (stem bark suber powder) | 239 |
| 138 | Stuhlmoavin (New) | Naphthoquinone | 24 µM (Dd2) |  | *Stuhlmannia moavi* (leaf) | 240 |
| 139 | Bonducellin | Homoisoflavonoid | 26 µM (Dd2) |  | *Stuhlmannia moavi* (root) | 240 |
| 140 | 3,4,5’-trihydroxy-  3’-methoxy-trans-stilbene | Stilbene | 27 µM (Dd2) |  | *Stuhlmannia moavi* (root) | 240 |
| 141 | Amplexine | Seco iridoid | 7.1 µg/ml (W2)  35.45 µM |  | *Tachia grandiflora* (leaf) | 241 |
| 142 | 23-galloylarjunic acid | Triterpene | 4.5 µg/ml (D6)  6.85 µM  2.8 µg/ml (W2)  4.26 µM |  | *Terminalia brownii* (stem bark) | 242 |
| 143 | 4-*O*-(3″,4″-di-*O*-galloyl-α-L-rhamno  pyranosyl)ellagic acid | Phenolic glycoside | 4.7 µg/ml (D6)  6.2 µM  4.7 µg/ml (W2)  6.2 µM |  | *Terminalia brownii* (stem bark) | 242 |
| 144 | Vernopicrin | Sesquiterpene lactone | 2.33 µM (Dd2)  1.77 µM (Hb3) |  | *Vernonia guineensis* (leaf) | 243 |
| 145 | Vernomelitensin | Sesquiterpene lactone | 1.64 µM (Dd2)  1.36 µM (Hb3) |  | *Vernonia guineensis* (leaf) | 243 |
| 146 | Pentaisovalerylsucrose | Saccharide ester | 1.96 µM (Dd2)  2.13 µM (Hb3) |  | *Vernonia guineensis* (root) | 243 |
| 147 | Dicerandrol D (New) | Polyketide | 600 nM (3D7) | Endophytic fungus | *Diaporthe sp.* (CY-5188) | 244 |
| 148 | Orthidine F | Polyamine diamide | 0.89 µM (K1) | Ascidian | *Aplidium orthium* | 245 |
| 149 | Axidjiferosides A + B + C (New) | Glycosphingolipid | 0.53 µM (FcB1) | Marine sponge | *Axinyssa djiferi* | 246 |
| 150 | Eleganolone | Diterpene | 7.9 µM (K1) | Algae | *Bifurcaria bifurcata* | 247 |
| 151 | Bromophycolide A | Macrocyclic meroditerpene | 47% suppression of P. yoelii parasite in infected mice at 10mg/kg | Algae | *Callophycus serratus* | 248 |
| 152 | Apicidin F (New) | Cyclic tetrapeptides | 0.67 µM (NF54) | Fungus | *Fusarium fujikuroi* | 249 |
| 153 | Kabiramide L (New) | Trisoxazole macrolide | 2.6 µM (K1) | Marine sponge | *Pachastrissa nux* | 250 |
| 154 | Kabiramide I | Trisoxazole macrolide | 4.5 µM (K1) | Marine sponge | *Pachastrissa nux* | 250 |
| 155 | Thiaplakortone A (New) | Thiazine alkaloid | 51 nM (3D7)  6.6 nM (Dd2) | Marine sponge | *Plakortis lita* | 251 |
| 156 | Thiaplakortone B (New) | Thiazine alkaloid | 650 nM (3D7)  92 nM (Dd2) | Marine sponge | *Plakortis lita* | 251 |
| 157 | Thiaplakortone C (New) | Thiazine alkaloid | 309 nM (3D7)  171 nM (Dd2) | Marine sponge | *Plakortis lita* | 251 |
| 158 | Thiaplakortone D (New) | Thiazine alkaloid | 279 nM (3D7)  159 nM (Dd2) | Marine sponge | *Plakortis lita* | 251 |
| 159 | Saccharosporone A (New) | Aromatic  polyketides | 4.1 µM (K1) | Actinomycete | *Saccharopolyspora sp.* (BCC 21906) | 252 |
| 160 | Saccharosporone B (New) | Aromatic  polyketides | 3.9 µM (K1) | Actinomycete | *Saccharopolyspora sp.* (BCC 21906) | 252 |
| 161 | (+)-rubiginone B2 | Aromatic  polyketides | 6.0 µM (K1) | Actinomycete | *Saccharopolyspora sp.* (BCC 21906) | 252 |
| 162 | Tetrangulol methyl ether | Aromatic  polyketides | 4.6 µM (K1) | Actinomycete | *Saccharopolyspora sp.* (BCC 21906) | 252 |
| 163 | Farneside A (New) | Sesquiterpenoid Nucleoside | 69.3 µM (3D7) | Actinobacteria | *Streptomyces sp.*(CNT-372) | 253 |

2012

| 1 | 15-acetoxy-8β-[(2-methylbutyryloxy)]-14-oxo-4,5-cis-acanthospermolide | Sesquiterpene lactone | 2.9 µM (3D7) | Plant | *Acanthospermum hispidum* (aerial part) | 254 |
| --- | --- | --- | --- | --- | --- | --- |
| 2 | 9α-acetoxy-15-hydroxy-8β-(2-methylbutyryloxy)-14-oxo-4,5-trans-acanthospermolide | Sesquiterpene lactone | 2.23 µM (3D7) | Plant | *Acanthospermum hispidum* (aerial part) | 254 |
| 3 | 3',4',7-trihydroxyflavone | Flavonoid | 0.078 µg/ml  0.28 µM | Plant | *Albizia zygia* (bark) | 255 |
| 4 | Jozimine A2 (New) | Naphthylisoquinoline | 1.4 nM (NF54) | Plant | *Ancistrocladus sp.* (root bark) | 256 |
| 5 | Voulkensin B | Cassane diterpene | 46.13 µg/ml (D6)  127.26 µM  34.43 µg/ml (W2)  94.98 µM | Plant | *Caesalpinia volkensii* (root bark) | 257 |
| 6 | Deoxycaesaldekarin C | Cassane diterpene | 25.67 µg/ml (D6)  77.73 µM  30.33 µg/ml (W2)  91.85 µM | Plant | *Caesalpinia volkensii* (root bark) | 257 |
| 7 | Caesaldekarin C | Cassane diterpene | 34.44 µg/ml (D6)  99.40 µM  30.69 µg/ml (W2)  88.58 µM | Plant | *Caesalpinia volkensii* (root bark) | 257 |
| 8 | 5-hydroxy vinhaticoic acid | Cassane diterpene | 46.14 µg/ml (D6)  138.79 µM  47.54 µg/ml (W2)  143.00 µM | Plant | *Caesalpinia volkensii* (root bark) | 257 |
| 9 | Cassiarin G (New) | Isoquinoline alkaloid | >50 µM (3D7) |  | *Cassia siamea* (leaf) | 258 |
| 10 | Cassiarin H (New) | Isoquinoline alkaloid | >50 µM (3D7) |  | *Cassia siamea* (leaf) | 258 |
| 11 | Cassiarin J (New) | Isoquinoline alkaloid | 0.3 µM (3D7) |  | *Cassia siamea* (leaf) | 258 |
| 12 | Cassiarin K (New) | Isoquinoline alkaloid | 1.4 µM (3D7) |  | *Cassia siamea* (leaf) | 258 |
| 13 | ergosta-4,6,8,22-tetraene-3-one | Sterol | 61.0 µM (D10) |  | *Cornus florida* (bark) | 259 |
| 14 | 3-epideoxyflindissol (New) | Tirucallane triterpene | 128.0 µM (D10) |  | *Cornus florida* (bark) | 259 |
| 15 | 3β-O-cis-coumaroylbetulinic acid | Lupane triterpene | 10.4 µM (D10) |  | *Cornus florida* (bark) | 259 |
| 16 | 3β-O-trans-coumaroylbetulinic acid | Lupane triterpene | 15.3 µM (D10) |  | *Cornus florida* (bark) |  |
| 17 | Dubiamine (New) | Isoquinoline alkaloid | >20 µg/ml (TM4)  >66.85 µM  >20 µg/ml (K1CB1)  >66.85 µM |  | *Corydalis dubia* (whole plant) | 260 |
| 18 | Scoulerine | Isoquinoline alkaloid | 1.78 µg/ml (TM4)  5.43 µM  1.04 µg/ml (K1CB1)  3.17 µM |  | *Corydalis dubia* (whole plant) | 260 |
| 19 | Capnoidine | Isoquinoline alkaloid | >20 µg/ml (TM4)  >54.44 µM  >20 µg/ml (K1CB1)  >54.44 |  | *Corydalis dubia* (whole plant) | 260 |
| 20 | Bicuculline | Isoquinoline alkaloid | >10 µg/ml (TM4)  >27.22 µM  >10 µg/ml (K1CB1)  >27.22 µM |  | *Corydalis dubia* (whole plant) | 260 |
| 21 | Protopine | Isoquinoline alkaloid | 1.45 µg/ml (TM4)  4.10 µM  1.38 µg/ml (K1CB1)  3.90 µM |  | *Corydalis crispa* (whole plant) | 261 |
| 22 | 13-oxoprotopine | Isoquinoline alkaloid | >4.6 µg/ml (TM4)  >12.52 µM  >4.6 µg/ml (K1CB1)  >12.52 µM |  | *Corydalis crispa* (whole plant) | 261 |
| 23 | Stylopine | Isoquinoline alkaloid | >4.0 µg/ml (TM4)  >12.37 µM  >4.0 µg/ml (K1CB1)  >12.37 µM |  | *Corydalis crispa* (whole plant) | 261 |
| 24 | Coreximine | Isoquinoline alkaloid | 5.56 µg/ml (TM4)  16.98 µM  6.87 µg/ml (K1CB1)  20.98 µM |  | *Corydalis crispa* (whole plant) | 261 |
| 25 | Ochrobirine | Isoquinoline alkaloid | >9.2 µg/ml (TM4)  >24.90 µM  >9.2 µg/ml (K1CB1)  >24.90 µM |  | *Corydalis crispa* (whole plant) | 261 |
| 26 | Bicuculline | Isoquinoline alkaloid | >9.2 µg/ml (TM4)  >25.04 µM  >9.2 µg/ml (K1CB1)  >25.04 µM |  | *Corydalis crispa* (whole plant) | 261 |
| 27 | Urospermal A-15-O-acetate | Sesquiterpene lactone | 0.92 µM (3D)  0.77 µM (W2) |  | *Dicoma tomentosa* (whole plant) | 262 |
| 28 | Sanandajin (New) | Sesquiterpene coumarin | 2.6 µM (K1) |  | *Ferula pseudalliacea* (root) | 263 |
| 29 | Kamolonol acetate (New) | Sesquiterpene coumarin | 16.1 µM (K1) |  | *Ferula pseudalliacea* (root) | 263 |
| 30 | Methyl galbanate | Sesquiterpene coumarin | 7.1 µM (K1) |  | *Ferula pseudalliacea* (root) | 263 |
| 31 | Geissolosimine | Indole alkaloid | 0.96 µM (D10) |  | *Geissospermum vellosii* (stem bark) | 264 |
| 32 | Geissospermine | Indole alkaloid | 5.02 µM (D10) |  | *Geissospermum vellosii* (stem bark) | 264 |
| 33 | Geissoschizoline | Indole alkaloid | 13.96 µM (D10) |  | *Geissospermum vellosii* (stem bark) | 264 |
| 34 | Geissoschizone (New) | Indole alkaloid | 10.29 µM (D10) |  | *Geissospermum vellosii* (stem bark) | 264 |
| 35 | Vellosiminol | Indole alkaloid | 157.0 µM (D10) |  | *Geissospermum vellosii* (stem bark) | 264 |
| 36 | Decarboxyportentol acetate (New) | Spiro heterocycle | 16 µM (3D7) |  | *Laumoniera bruceadelpha* (bark) | 265 |
| 37 | 3,4-dehydrothespirone (New) | Spiro heterocycle | 0.027 µM (3D7) |  | *Laumoniera bruceadelpha* (bark) | 265 |
| 38 | Nymphaeol B | Flavonoid | 4.02 µM (3D7) |  | *Macaranga triloba* (inflorescence) | 266 |
| 39 | Nymphaeol C | Flavonoid | 2.04 µM (3D7) |  | *Macaranga triloba* (inflorescence) | 266 |
| 40 | 6-farnesyl-3′,4′,5,7-tetrahydroxyflavanone | Flavonoid | 0.06 µM (3D7) |  | *Macaranga triloba* (inflorescence) | 266 |
| 41 | 3’-Oxotabernaelegantine B (New) | Indole alkaloid | 10.4 µM (FcB1) |  | *Muntafara sessilifolia* (stem bark) | 267 |
| 42 | 3’-Oxotabernaelegantine A (New) | Indole alkaloid | 4.4 µM (FcB1) |  | *Muntafara sessilifolia* (stem bark) | 267 |
| 43 | 3’(R/S)-Hydroxytabernaelegantine A (New) | Indole alkaloid | 6.2 µM (FcB1) |  | *Muntafara sessilifolia* (stem bark) | 267 |
| 44 | 3’(S)-Hydroxytabernaelegantine C (New) | Indole alkaloid | 6.1 µM (FcB1) |  | *Muntafara sessilifolia* (stem bark) | 267 |
| 45 | 19,20α-Dihydroeleganine A (New) | Indole alkaloid | 137.8 µM (FcB1) |  | *Muntafara sessilifolia* (stem bark) | 267 |
| 46 | 3-oxocoronaridine -7-hydroxy  indolenine | Indole alkaloid | 118.2 µM (FcB1) |  | *Muntafara sessilifolia* (stem bark) | 267 |
| 47 | 3-oxocoronaridine | Indole alkaloid | 10.8 µM (FcB1) |  | *Muntafara sessilifolia* (stem bark) | 267 |
| 48 | 3(R/S)-hydroxycoronaridine | Indole alkaloid | 5.9 µM (FcB1) |  | *Muntafara sessilifolia* (stem bark) | 267 |
| 49 | Coronaridine | Indole alkaloid | 6.21 µM (FcB1) |  | *Muntafara sessilifolia* (stem bark) | 267 |
| 50 | Tabernaemontanine | Indole alkaloid | 12.0 µM (FcB1) |  | *Muntafara sessilifolia* (stem bark) | 267 |
| 51 | Tabernaelegantine B | Indole alkaloid | 2.7 µM (FcB1) |  | *Muntafara sessilifolia* (stem bark) | 267 |
| 52 | Tabernaelegantine D | Indole alkaloid | 1.2 µM (FcB1) |  | *Muntafara sessilifolia* (stem bark) | 267 |
| 53 | Tabernaelegantine A | Indole alkaloid | 13.3 µM (FcB1) |  | *Muntafara sessilifolia* (stem bark) | 267 |
| 54 | Chamaejasmin | Flavonoid | 14.01 µM (D10) |  | *Ormocarpum trichocarpum* (aerial part) | 268 |
| 55 | Diphysin | Flavonoid | 16.90 µM (D10) |  | *Ormocarpum trichocarpum* (aerial part) | 268 |
| 56 | 3’’-epidiphysin (New) | Flavonoid | 94.32 µM (D10) |  | *Ormocarpum trichocarpum* (aerial part) | 268 |
| 57 | 7,7’’-di-O-methylchamaejasmin (4) + 7,7’’-di-O-methylisochamaejasmin (New) | Flavonoid | 4.03 µM (D10) |  | *Ormocarpum trichocarpum* (aerial part) | 268 |
| 58 | 16β-hydroxylupane-1,20(29)-dien-3-one | Lupane tritrpene | 28.3 µM (K1) |  | *Parinari excelsa* (stem bark) | 269 |
| 59 | Oleanolic acid | Oleanane triterpene | 69.9 µM (K1) |  | *Parinari excelsa* (stem bark) | 269 |
| 60 | 3β-hydroxy-olean-5,12-dien-28-oic acid | Oleanane triterpene | 77.9 µM (K1) |  | *Parinari excelsa* (stem bark) | 269 |
| 61 | 3-*O*-β-d-glucopyranosyl-stigmasta-5,11(12)-diene | Sterol glycoside | 145.0 µM (K1) |  | *Parinari excelsa (stem bark)* | 269 |
| 62 | Chlorogenic acid | Phenolic ester | 74.1 µM (K1) |  | *Parinari excelsa (stem bark)* | 269 |
| 63 | 1-(7-allyl-5-(ethoxymethoxy)-8-methoxy-4,6-bis(methoxy  methoxy)naphthalen-1-yl)ethanone (New) | Naphthoquinone | 90.5 µM (K1) |  | *Parinari excelsa (stem bark)* | 269 |
| 64 | Jacaranone | Benzoquinone | 7.82 µg/ml (K1)  42.92 µM |  | *Pentacalia desiderabilis* (leaf) | 270 |
| 65 | Geraniin | Ellagitannin | 11.74 µM (3D7) |  | *Phyllanthus muellerianus* | 271 |
| 66 | Ellagic acid | Phenolic | 2.88 µM (3D7) |  | *Anogeissus leiocarpus* | 271 |
| 67 | Gallic acid | Phenolic acid | 71.53 µM (3D7) |  | *Anogeissus leiocarpus* | 271 |
| 68 | Gentisic acid | Phenolic acid | 4.76 µM (3D7) |  | *Anogeissus leiocarpus* | 271 |
| 69 | Simalikalactone D | Quassinoid | 10 nM (FcB1) |  | *Quassia amara* | 272 |
| 70 | Tomentosone A (New) | Polycyclic phloroglucinol | 1.0 µM (3D7)  1.49 µM (Dd2) |  | *Rhodomyrtus tomentosa* (leaf) | 273 |
| 71 | Tomentosone B (New) | Polycyclic phloroglucinol | 75 (3D7) and 45 (Dd2)% Inhibition at 40 µM |  | *Rhodomyrtus tomentosa* (leaf) | 273 |
| 72 | 2,3,6-trihydroxy benzoic acid | Phenolic acid | 16.47 µM (W2)  53.66% suppression of parasite in P. berghei infected mice at 50mg/kg |  | *Sorindeia juglandifolia* (fruit) | 274 |
| 73 | 2,3,6-trihydroxy methyl benzoate | Phenolic ester | 13.04 µM (W2) |  | *Sorindeia juglandifolia* (fruit) | 274 |
| 74 | Spiranthenone A (New) | Acylphloroglucinol | 23.4 µg/ml (FcB1)  45.67 µM |  | *Spiranthera*  *Odoratissima* (leaf) |  |
| 75 | Spiranthenone B (New) | Acylphloroglucinol | 15.5 µg/ml (FcB1)  32.13 µM |  | *Spiranthera*  *Odoratissima* (leaf) | 275 |
| 76 | 6α-acetoxy,1β-hydroxyeudesm-4(15)-ene (New) | Sesquiterpene | 13.9 µg/ml (FcB1)  49.60 µM |  | *Spiranthera*  *Odoratissima* (leaf) | 275 |
| 77 | Sesamine | Lignan | 9.1 µg/ml (FcB1)  25.68 µM |  | *Spiranthera*  *Odoratissima* (leaf) | 275 |
| 78 | 3-hydroxylongicaudatine Y (New) | Indole alkaloid | 6.22 µM (3D7)  21.84 µM (W2) |  | *Strychnos malacoclados* (stem bark) | 276 |
| 79 | Bisnordihydrotoxiferine | Indole alkaloid | 3.29 µM (3D7) |  | *Strychnos malacoclados* (stem bark) | 276 |
| 80 | Divarine | Indole alkaloid | 1.37 µM (3D7)  0.50 µM (W2) |  | *Strychnos malacoclados* (stem bark) | 276 |
| 81 | Longicaudatine | Indole alkaloid | 0.68 µM (3D7)  0.57 µM (W2) |  | *Strychnos malacoclados* (stem bark) | 276 |
| 82 | Longicaudatine Y | Indole alkaloid | 1.51 µM (3D7)  0.72 µM (W2) |  | *Strychnos malacoclados* (stem bark) | 276 |
| 83 | Longicaudatine F | Indole alkaloid | 1.19 µM (3D7)  1.04 µM (W2) |  | *Strychnos malacoclados* (stem bark) | 276 |
| 84 | Gedunin | Limonoid | MIC 10 µg/ml (3D7)  20.72 µM |  | *Xylocarpus granatum* (fruit) | 277 |
| 85 | Xyloccensin-I | Limonoid | MIC 10 µg/ml (3D7)  15.46 µM |  | *Xylocarpus granatum (fruit)* | 277 |
| 86 | Kororamide A (New) | Indole alkaloid | 72 and 50% inhibition at 20 µM | Bryozoan | *Amathia tortuosa* | 278 |
| 87 | Convolutamine F | Alkaloid | 80% inhibition at 40 µM | Bryozoan | *Amathia tortuosa* | 278 |
| 88 | 19-Hydroxypsammaplysin E (New) | Spirooxepinisooxazole alkaloid | 6.4 µM (3D7) | Marine sponge | *Aplysinella strongylata* | 279 |
| 89 | Bromophycoic acid A (New) | Diterpene-benzoate | 30.7 µM (3D7) | Red algae | *Callophycus sp.* | 280 |
| 90 | Bromophycoic acid B (New) | Diterpene-benzoate | 41.3 µM (3D7) | Red algae | *Callophycus sp.* | 280 |
| 91 | Bromophycoic acid C (New) | Diterpene-benzoate | 8.7 µM (3D7) | Red algae | *Callophycus sp.* | 280 |
| 92 | Bromophycoic acid D (New) | Diterpene-benzoate | 27.0 µM (3D7) | Red algae | *Callophycus sp.* | 280 |
| 93 | Bromophycoic acid E (New) | Diterpene-benzoate | >100 µM (3D7) | Red algae | *Callophycus sp.* | 280 |
| 94 | Albopunctatone (New) | Anthrone-anthraquinone | 5.3 µM (3D7)  4.4 µM (Dd2) | Ascidian | *Didemnum albopunctatum* | 281 |
| 95 | (E)-2-(hept-1-enyl)-3 (hydroxymethyl)-5-(3-methyl  but-2-enyl)benzene-1,4-diol (New) | Phenolic | 1. µg/ml (D6)   6.08 µM  2.8 µg/ml (W2)  8.52 µM | Fungus | *Eurotium repens* | 282 |
| 96 | Flavoglaucin | Phenolic | 3.0 µg/ml (D6)  9.85 µM  2.7 µg/ml (W2)  8.86 µM | Fungus | *Eurotium repens* | 282 |
| 97 | Tetrahydroauroglaucin | Phenolic | 2.8 µg/ml (D6)  9.25 µM  2.3 µg/ml (W2)  7.60 µM | Fungus | *Eurotium repens* | 282 |
| 98 | Auroglaucin | Phenolic | 1.8 µg/ml (D6)  6.03 µM  1.1 µg/ml (W2)  3.68 µM | Fungus | *Eurotium repens* | 282 |
| 99 | 2-(2’,3-epoxy-1’,3’-heptadienyl)-6-hydroxy-5-(3-methyl-2-butenyl)benzaldehyde | Benzofuran | 1. µg/ml (D6)   6.70 µM  2.8 µg/ml (W2)  9.38 µM | Fungus | *Eurotium repens* | 282 |
| 100 | Lasionectrin (New) | Naphthopyrone | 11.0 µM (3D7) | Fungus | *Lasionectria sp.* | 283 |
| 101 | Ingamine A | Ingamine alkaloid | 90 ng/ml (D6)  0.20 µM  72 ng/ml (W2)  0.16 µM | Marine sponge | *Petrosid* Ng5 Sp5 | 284 |
| 102 | 22(S)-Hydroxyingamine A (New) | Ingamine (piperidine) alkaloid | 220 ng/ml (D6)  0.47 µM  140 ng/ml (W2)  0.301 µM | Marine sponge | *Petrosid* Ng5 Sp5 | 284 |
| 103 | Dihydroingenamine D (New) | Ingamine alkaloid | 78 ng/ml (D6)  0.183 µM  57 ng/ml (W2)  0.134 µM | Marine sponge | *Petrosid* Ng5 Sp5 | 284 |
| 104 | (2Z,6R,8R,9E)[3-ethyl-5-(2-ethyl-hex-3-enyl)-6-methyl-5H-furan-2-ylidene]-acetic acid methyl ester | Furanylidenic ester | 2.0 µg/ml (D6)  6.8 µM  2.0 µg/ml (W2)  6.8 µM | Marine sponge | *Plakortis simplex.* | 285 |
| 105 | 6-deoxy-8-O-methylrabelomycin | Anthraquinone | 18.5 µM (P. berghei liver stage) | Fungus | *Pseudonocardia sp.* EC080529-01 | 286 |
| 106 | 8-O-Methyltetrangulol (X-14881 E) | Anthraquinone | 3.0 µM (P. berghei liver stage) | Fungus | *Pseudonocardia sp.* EC080529-01 | 286 |
| 107 | (+)-cercosporin | Perylenequinone | 1.08 µM (D6)  1.62 µM (W2) | Ascomycetes fungi | *Septoria pistaciarum* | 287 |
| 108 | (+)-14-O-acetylcercosporin | Perylenequinone | 2.78 µM (D6)  3.12 µM (W2) | Ascomycetes fungi | *Septoria pistaciarum* | 287 |
| 109 | (+)-di-O-acetylcercosporin | Perylenequinone | 2.75 µM (D6)  1.94 µM (W2) | Ascomycetes fungi | *Septoria pistaciarum* | 287 |
| 110 | Urdamycinone E (New) | Anthraquinone | 0.053 µg/ml (K1)  0.099 µM | Actinobacteria | *Streptomyces sp.* BCC45596 | 288 |
| 111 | Urdamycinone G (New) | Anthraquinone | 0.142 µg/ml (K1)  0.27 µM | Actinobacteria | *Streptomyces sp.* BCC45596 | 288 |
| 112 | Dehydroxyaquayamycin (New) | Anthraquinone | 2.93 µg/ml (K1)  6.74 µM | Actinobacteria | *Streptomyces sp.* BCC45596 | 288 |
| 113 | Urdamycin E | Anthraquinone | 0.17 µg/ml (K1)  0.19 µM | Actinobacteria | *Streptomyces sp.* BCC45596 | 288 |
| 114 | 8-isocyanato-15-formamido  amphilect-11(20)-ene(New) | Amphilectane diterpene | 8.85 µM (K1) | Sponge | *Stylissa cf. massa* | 289 |
| 115 | 8-isothiocyanato-15-formamido  amphilect-11(20)-ene (New) | Amphilectane diterpene | 8.07 µM (K1) | Sponge | *Stylissa cf. massa* | 289 |
| 116 | 8-isocyano-15-formamidoamphilect-11(20)-ene | Amphilectane diterpene | 0.52 µM (K1) | Sponge | *Stylissa cf. massa* | 289 |
| 117 | Araplysillin I | Isooxazoline alkaloid | 4.5 µM (FcB1)  4.6 µM (3D7) | Sponge | *Suberea ianthelliformis* | 290 |
| 118 | Araplysillin N20-formamide (New) | Isooxazoline alkaloid | 3.6 µM (FcB1)  7.0 µM (3D7) | Sponge | *Suberea ianthelliformis* | 290 |
| 119 | Araplysillin N20-hydroxyformamide (New) | Isooxazoline alkaloid | 5.0 µM (FcB1)  4.1 µM (3D7) | Sponge | *Suberea ianthelliformis* | 290 |
| 120 | Araplysillin II | Isooxazoline alkaloid | 34.2 µM (FcB1) | Sponge | *Suberea ianthelliformis* | 290 |
| 121 | Araplysillin IV (New) | Isooxazoline alkaloid | 27.6 µM (FcB1) | Sponge | *Suberea ianthelliformis* | 290 |
| 122 | Araplysillin V (New) | Isooxazoline alkaloid | 50.5 µM (FcB1) | Sponge | *Suberea ianthelliformis* | 290 |
| 123 | Araplysillin VI (New) | Bromotyrosine derivative | 37.4 µM (FcB1) | Sponge | *Suberea ianthelliformis* | 290 |
| 124 | Aerophobin I | Isooxazoline alkaloid | 59.0 µM (FcB1) | Sponge | *Suberea ianthelliformis* | 290 |
| 125 | Aerophobin II | Isooxazoline alkaloid | 24.9 µM (FcB1)  19.9 µM (3D7) | Sponge | *Suberea ianthelliformis* | 290 |
| 126 | Purealidin Q | Isooxazoline alkaloid | 3.6 µM (FcB1) | Sponge | *Suberea ianthelliformis* | 290 |
| 127 | Aerothionin | Isooxazoline alkaloid | 3.4 µM (FcB1)  4.2 µM (3D7) | Sponge | *Suberea ianthelliformis* | 290 |
| 128 | Homoaerothionin | Isooxazoline alkaloid | 2.8 µM (FcB1)  4.0 µM (3D7) | Sponge | *Suberea ianthelliformis* | 290 |
| 129 | Aplysinone D | Isooxazoline alkaloid | 1.0 µM (FcB1)  3.1 µM (3D7) | Sponge | *Suberea ianthelliformis* | 290 |
| 130 | 11,19-Dideoxyfistularin 3 | Isooxazoline alkaloid | 2.1 µM (FcB1)  0.9 µM (3D7) | Sponge | *Suberea ianthelliformis* | 290 |
| 131 | 11-Hydroxyfistularin 3 | Isooxazoline alkaloid | 2.1 µM (FcB1)  2.6 µM (3D7) | Sponge | *Suberea ianthelliformis* | 290 |
| 132 | Torrubiellin A (New) | Anthraquinone | 3.10 µM (K1) | Fungus | *Torrubiella sp.* BCC 28517 | 291 |
| 133 | Torrubiellin B (New) | Anthraquinone | 0.33 µM (K1) | Fungus | *Torrubiella sp.* BCC 28517 | 291 |
| 134 | Tsitsikammamine C (New) | Pyrroloiminoquinone alkaloid | 13 nM (3D7)  18 nM (Dd2) | Marine sponge | *Zyzzya sp* | 292 |
| 135 | Makaluvamine J | Pyrroloiminoquinone alkaloid | 25 nM (3D7)  22 nM (Dd2) | Marine sponge | *Zyzzya sp* | 292 |
| 136 | Makaluvamine G | Pyrroloiminoquinone alkaloid | 36 nM (3D7)  39 nM (Dd2)  48% suppression of parasite in P. berghei infected mice | Marine sponge | *Zyzzya sp* | 292 |
| 137 | Makaluvamine L | Pyrroloiminoquinone alkaloid | 40 nM (3D7)  21 nM (Dd2) | Marine sponge | *Zyzzya sp* | 292 |
| 138 | Makaluvamine K | Pyrroloiminoquinone alkaloid | 396 nM (3D7)  300 nM (Dd2) | Marine sponge | *Zyzzya sp* | 292 |
| 139 | Damirone A | Pyrroloiminoquinone alkaloid | 1.88 µM (3D7)  0.36 µM (Dd2) | Marine sponge | *Zyzzya sp* | 292 |
| 140 | Damirone B | Pyrroloiminoquinone alkaloid | 12.25 µM (3D7)  3.8 µM (Dd2) | Marine sponge | *Zyzzya sp* | 292 |

2011

| 1 | Amorphaquinone | Isoflavanquinone | 3.0 µg/ml (D6)  8.66 µM  3.7 µg/ml (W2)  10.69 µM | Plant | *Abrus schimperi* (leaf) | 293 |
| --- | --- | --- | --- | --- | --- | --- |
| 2 | Pendulone | Isoflavanquinone | 1.7 µg/ml (D6)  4.9 µM  2.2 µg/ml (W2)  6.35 µM | Plant | *Abrus schimperi* (leaf) | 293 |
| 3 | Rutin | Flavonoid glycoside | 68.5 µg/ml (D10)  112.19 µM  76.4 µg/ml (W2)  125.13 µM | Plant | *Achillea millefolium* (aerial part) | 294 |
| 4 | Luteolin 7-*O*-glucoside | Flavonoid glycoside | 26.2 µg/ml (D10)  58.43 µM  26.8 µg/ml (W2)  59.77 µM | Plant | *Achillea millefolium* (aerial part) | 294 |
| 5 | Apigenin 4′-*O*-glucoside | Flavonoid glycoside | 71.4 µg/ml (D10)  165.13 µM  58.7 µg/ml (W2)  135.76 µM | Plant | *Achillea millefolium* (aerial part) | 294 |
| 6 | Apigenin 7-*O*-glucoside | Flavonoid glycoside | 10.1 µg/ml (D10)  23.35 µM  6.1 µg/ml (W2)  14.10 µM | Plant | *Achillea millefolium* (aerial part) | 294 |
| 7 | Ethyl eichlerianoate | Dammarane  triterpenoid | >10 µg/ml (K1)  >19.88 µM | Plant | *Aglaia erythrosperma* (fruit) | 295 |
| 8 | Aglinin A | Dammarane  triterpenoid | >10 µg/ml (K1)  >20.37 µM | Plant | *Aglaia erythrosperma* (fruit) | 295 |
| 9 | 5,6-desmethylenedioxy-5-methoxy-aglalactone | Benzolactone | >10 µg/ml (K1)  >33.29 µM | Plant | *Aglaia erythrosperma* (leaf) | 295 |
| 10 | 4’-demethoxy-3’,4’-methylenedioxy  Methylrocaglate | Benzofuran (flavagline) | 7.3 µg/ml (K1)  14.41 µM | Plant | *Aglaia erythrosperm* (seed) | 295 |
| 11 | Alisol A | Protostane triterpenoid | 13.8 µM (K1) | Plant | *Alisma plantago-aquatica* (root) | 296 |
| 12 | Alisol B 23-acetate | Protostane triterpenoid | 12.8 µM (K1) | Plant | *Alisma plantago-aquatica* (root) | 296 |
| 13 | Alisol B 11-monoacetate | Protostane triterpenoid | 5.41 µM (K1) | Plant | *Alisma plantago-aquatica* (root) | 296 |
| 14 | Alisol G | Protostane triterpenoid | 6.98 µM (K1) | Plant | *Alisma plantago-aquatica* (root) | 296 |
| 15 | Marmesinin | Coumarin | 5.3 µM (D10) |  | *Angelica gigas* (root) | 297 |
| 16 | Nodakenin | Coumarin | >100 µM (D10) |  | *Angelica gigas* (root) | 297 |
| 17 | Skimmin | Coumarin | >100 µM (D10) |  | *Angelica gigas* (root) | 297 |
| 18 | Apiosylskimmin | Coumarin | 42.7 µM (D10) |  | *Angelica gigas* (root) | 297 |
| 19 | Magnolioside | Coumarin | 8.2 µM (D10) |  | *Angelica gigas* (root) | 297 |
| 20 | Eudesmin | Lignan | >25 µg/ml (FcB1)  >64.69 µM |  | *Artemisia gorgonum* (aerial part) | 298 |
| 21 | Magnolin | Lignan | 22.7 µg/ml (FcB1)  54.50 µM |  | *Artemisia gorgonum* (aerial part) | 298 |
| 22 | Epimagnolin A | Lignan | 5.7 µg/ml (FcB1)  13.68 µM |  | *Artemisia gorgonum* (aerial part) | 298 |
| 23 | Aschantin | Lignan | 5.7 µg/ml (FcB1)  14.23 µM |  | *Artemisia gorgonum* (aerial part) | 298 |
| 24 | Kobusin | Lignan | 7.67 µg/ml (FcB1)  20.70 µM |  | *Artemisia gorgonum* (aerial part) | 298 |
| 25 | Sesamin | Lignan | 3.37 µg/ml (FcB1)  9.51 µM |  | *Artemisia gorgonum* (aerial part) | 298 |
| 26 | Artemetin | Flavonoid | 3.50 µg/ml (FcB1)  9.01 µM |  | *Artemisia gorgonum* (aerial part) | 298 |
| 27 | Athrolide A (New) | Sesquiterpene lactone | 34.3 µM (HB3)  32.7 µM (Dd2) |  | *Athroisma proteiforme* (aerial part) | 299 |
| 28 | Athrolide C (New) | Sesquiterpene lactone | 6.6 µM (HB3)  5.5 µM (Dd2) |  | *Athroisma proteiforme* (aerial part) | 299 |
| 29 | Athrolide D (New) | Sesquiterpene lactone | 7.2 µM (HB3)  4.2 µM (Dd2) |  | *Athroisma proteiforme* (aerial part) | 299 |
| 30 | Athrolide E (New) | Sesquiterpene lactone | 16.0 µM (HB3)  11.7 µM (Dd2) |  | *Athroisma proteiforme* (aerial part) | 299 |
| 31 | Cananginone E (New) | Acetogenin | 24.4 µM (K1) |  | *Cananga latifolia* (stem bark) | 300 |
| 32 | Cassiarin F (New) | Alkaloid | 3.3 µM (3D7) |  | *Cassia siamea* (flower) | 301 |
| 33 | Rutarin | Coumarin | 88.0 µg/ml (FcB1)  207.35 µM |  | *Citropsis articulata* (root bark) | 302 |
| 34 | Demethylsuberosin | Coumarin | 16.7 µg/ml (FcB1)  72.52 µM |  | *Citropsis articulata* (root bark) | 302 |
| 35 | 5-hydroxynoracronycine | Coumarin | 0.9 µg/ml (FcB1)  2.78 µM |  | *Citropsis articulata* (root bark) | 302 |
| 36 | 1,5-dihydroxy-2,3-dimethoxy-10-methyl-9-acridone | Acridone alkaloid | 3.0 µg/ml (FcB1)  9.95 µM |  | *Citropsis articulata* (root bark) | 302 |
| 37 | 7*α*-obacunyl acetate | Limonoid | 9.3 µg/ml (FcB1)  19.19 µM |  | *Citropsis articulata* (root bark) | 302 |
| 38 | 4-hydroxycembra-2*E*,7*E*,11*Z*-trien-  20,10-olide (New) | Diterpene | 20.8 µg/ml (D10)  65.31 µM |  | *Croton gratissimus* (leaf) | 303 |
| 39 | 7-acetoxy-1,4-dihydroxycembra-2*E*,8(19),11*Z*-trien-20,10-olide (New) | Diterpene | 13.5 µg/ml (D10)  34.39 µM |  | *Croton gratissimus* (leaf) | 303 |
| 40 | Cupacinoside (1) (New) | Diterpene glycosides | 1.3 µM (K1) |  | *Cupania cinerea* (bark) | 304 |
| 41 | 60-de-O-acetylcupacinoside (New) | Diterpene glycosides | 2.1 µM (K1) |  | *Cupania cinerea* (bark) | 304 |
| 42 | Cupacinoxepin (New) | Triterpene lactone | 8.7 µM (K1) |  | *Cupania cinerea* (bark) | 304 |
| 43 | Lupenone | Triterpene | 4.7 µM (K1) |  | *Cupania cinerea* (bark) | 304 |
| 44 | Betulone | Triterpene | 3.0 µM (K1) |  | *Cupania cinerea* (bark) | 304 |
| 45 | Taraxerol | Triterpene | 8.5 µM (K1) |  | *Cupania cinerea* (bark) | 304 |
| 46 | Dehydrobrachylaenolide | Sesquiterpene lactone | 1.86 µM (D10)  4.09 µM (K1) |  | *Dicoma anomala*  *subsp. Gerrardii* (root) | 305 |
| 47 | (2’R)-2’,3’-epoxy-N-methylatanine (New) | Quinolone alkaloid | 74% inhibition at 80 µM (Dd2) |  | *Drummondita calida* (stem) | 306 |
| 48 | 2-oxo-8-tigloyloxyguaia-1(10),3-diene-6,12-olide-14-carboxylic acid (New) | Sesquiterpene lactone | >13 µM |  | *Eupatorium perfoliatum* (aerial part) | 307 |
| 49 | 14-hydroxy-2-oxo-14(8-tigloyloxyguaia-1(10),2-diene-6,12,2,14-diolid-4-yl)-8-tigloyloxyguaia-1(10),3-diene-6,12-olide (New) | Sesquiterpene lactone | 2.0 µM |  | *Eupatorium perfoliatum* (aerial part) | 307 |
| 50 | 3α,14-dihydroxy-8β-tigloyloxy-6βH,  7αH,11αH-germacra-1(10)Z,4Z-dien-6,12-olide (New) | Sesquiterpene lactone | >13 µM |  | *Eupatorium perfoliatum* (aerial part) | 307 |
| 51 | Eupafolin | Flavonoid | 10.2 µM |  | *Eupatorium perfoliatum* (aerial part) | 307 |
| 52 | Robustaside E (New) | Phenolic glycoside | 55.5 µM (D6)  77.4 µM (3D7)  55.5 µM (Dd2)  29.4 µM (K1) |  | *GreVillea* “Poorinda Queen” (leaf and twi*g)* | 308 |
| 53 | Robustaside F (New) | Phenolic glycoside | 14.7 µM (D6) |  | *GreVillea* “Poorinda Queen” (leaf and twi*g)* | 308 |
| 54 | Robustaside G (New) | Phenolic glycoside | 4.7 µM (D6)  5.5 µM (3D7)  4.4 µM (Dd2)  3.9 µM (K1)  21% suppression of parasite in P. berghei infected mice |  | *GreVillea* “Poorinda Queen” (leaf and twi*g)* | 308 |
| 55 | Robustaside D | Phenolic glycoside | 5.1 µM (D6)  5.5 µM (3D7)  3.0 µM (Dd2)  3.0 µM (K1) |  | *GreVillea* “Poorinda Queen” (leaf and twi*g)* | 308 |
| 56 | Betulinic acid | Triterpene | 4.5 µM (W2mef)  5.6 µM (SHF4) |  | *Hypericum lanceolatum* (stem bark) | 309 |
| 57 | 2,2’,5,6’-Tetrahydroxybenzophenone | Benzophenone | 55.12 µM (W2mef)  13.41 µM (SHF4) |  | *Hypericum lanceolatum* (stem bark) | 309 |
| 58 | 5-Hydroxy-3-methoxyxanthone | Xanthone | 3.26 µM (W2mef)  1.43 µM (SHF4) |  | *Hypericum lanceolatum* (stem bark) | 309 |
| 59 | 3-Hydroxy-5-methoxyxanthone | Xanthone | 33.81 µM (W2mef)  34.09 µM (SHF4) |  | *Hypericum lanceolatum* (stem bark) | 309 |
| 60 | Atranorin | Phenolic (depside) | 4.41 µM (W2)  2.81 µM (CAM10)  2.78 µM (SHF4) |  | *Kigelia Africana* (stem bark) | 310 |
| 61 | 2β,3β,19α-trihydroxy-urs-12-en-28-oic acid | triterpene | 1.60 µM (W2)  2.17 µM (CAM10)  8.02 µM (SHF4) |  | *Kigelia Africana* (stem bark) | 310 |
| 62 | Specicoside | Iridoid glycoside | 1.54 µM (W2)  2.34 µM (CAM10)  2.7 µM (SHF4) |  | *Kigelia Africana* (stem bark) | 310 |
| 63 | *p*-hydroxycinnamoic acid | Phenolic acid | 53.84 µM (W2)  7.13 µM (CAM10)  6.71 µM (SHF4) |  | *Kigelia Africana* (stem bark) | 310 |
| 64 | Asimilobine | Isoquinoline (aporphine) alkaloid | 4.5 µM (D10)  21.7 µM (Dd2) |  | *Liriodendron tulipifera* (bark) | 311 |
| 65 | Norushinsunine | Isoquinoline alkaloid | 105.2 µM (D10)  109.8 µM (Dd2) |  | *Liriodendron tulipifera* (bark) | 311 |
| 66 | Norglaucine | Isoquinoline alkaloid | 64.4 µM (D10)  94.3 µM (Dd2) |  | *Liriodendron tulipifera* (bark) | 311 |
| 67 | Liriodenine | Isoquinoline alkaloid | 14.9 µM (D10)  28.7 µM (Dd2) |  | *Liriodendron tulipifera* (bark) | 311 |
| 68 | Anonaine | Isoquinoline alkaloid | 4.5 µM (D10)  19.6 µM (Dd2) |  | *Liriodendron tulipifera* (bark) | 311 |
| 69 | Oxoglaucine | Isoquinoline alkaloid | 25.9 µM (D10)  59.2 µM (Dd2) |  | *Liriodendron tulipifera* (bark) | 311 |
| 70 | Peroxyferolide | Sesquiterpene lactone | 18.3 µM (D10)  12.7 µM (Dd2) |  | *Liriodendron tulipifera* (leaf) | 311 |
| 71 | Lipiferolide | Sesquiterpene lactone | 5.9 µM (D10)  7.5 µM (Dd2) |  | *Liriodendron tulipifera* (leaf) | 311 |
| 72 | Karavilagenin C | Triterpenoid | 10.4 µM (3D7)  11.2 µM (Dd2)  Synthetic derivative showed improved activity |  | *Momordica*  *Balsamina* (aerial part) | 312 |
| 73 | Cucurbalsaminol C (New) | Triterpenoid | 52.7 µM (3D7)  67.6 µM (Dd2) |  | *Momordica*  *Balsamina* (aerial part) | 312 |
| 74 | Balsaminol F (New) | Triterpenoid | 18.0 µM (3D7)  20.0 µM (Dd2)  Synthetic derivative showed improved activity |  | *Momordica*  *Balsamina* (aerial part) | 313 |
| 75 | Balsaminoside B (New) | Triterpenoid | 2.9 µM (3D7)  6.3 µM (Dd2) |  | *Momordica*  *Balsamina* (aerial part) | 313 |
| 76 | Balsaminoside C | Triterpenoid | 3.4 µM (3D7)  7.2 µM (Dd2) |  | *Momordica*  *Balsamina* (aerial part) | 313 |
| 77 | Kuguaglycoside A | Triterpenoid | 3.9 µM (3D7)  2.5 µM (Dd2) |  | *Momordica*  *Balsamina* (aerial part) | 313 |
| 78 | Pentalongin | Naphthoquinone | 0.27 µg/ml (W2)  1.27 µM  0.23 µg/ml (D6)  1.08 µM |  | *Pentas longiflora* (root) | 314 |
| 79 | Psychorubrin | Naphthoquinone | 0.91 µg/ml (W2)  3.95 µM  0.82 µg/ml (D6)  3.56 µM |  | *Pentas longiflora* (root) | 314 |
| 80 | Mollugin | Naphthoquinone | 10.22 µg/ml (W2)  35.94 µM  7.56 µg/ml (D6)  26.59 µM |  | *Pentas longiflora* (root) | 314 |
| 81 | Tectoquinone | Anthraquinone | 10.78 µg/ml (W2)  48.51 µM  6.74 µg/ml (D6)  30.33 µM |  | *Pentas lanceolata* (root) | 314 |
| 82 | Rubiadin | Anthraquinone | 8.36 µg/ml (W2)  32.88 µM  5.47 µg/ml (D6)  21.51 µM |  | *Pentas lanceolata* (root) | 314 |
| 83 | Rubiadin-1-methyl ether | Anthraquinone | 18.91 µg/ml (W2)  70.49 µM  12.08 µg/ml (D6)  45.03 µM |  | *Pentas lanceolata* (root) | 314 |
| 84 | Nordamnacanthal | Anthraquinone | 9.33 µg/ml (W2)  34.78 µM  9.29 µg/ml (D6)  34.63 µM |  | *Pentas lanceolata* (root) | 314 |
| 85 | Damnacanthal | Anthraquinone | 10.88 µg/ml (W2)  38.57 µM  7.67 µg/ml (D6)  27.19 µM |  | *Pentas lanceolata* (root) | 314 |
| 86 | Lucidin-ω-methyl ether | Anthraquinone | 13.19 µg/ml (W2)  46.40 µM  12.08 µg/ml (D6)  42.49 µM |  | *Pentas lanceolata* (root) | 314 |
| 87 | Damnacanthol | Anthraquinone | 31.42 µg/ml (W2)  110.53 µM  16.07 µg/ml (D6)  56.53 µM |  | *Pentas lanceolata* (root) | 314 |
| 88 | 5,6-Dihydroxydamnacanthol(New) | Anthraquinone | 19.33 µg/ml (W2)  61.15 µM  15.02 µg/ml (D6)  47.52 µM |  | *Pentas lanceolata* (root) | 314 |
| 89 | Physalin B | Steroid | 2.8 µM (W2) |  | *Physalis angulata* | 315 |
| 90 | Physalin D | Steroid | 55 µM (W2)  65% suppression of parasite in P. berghei infected mice at 50mg/kg |  | *Physalis angulate* | 315 |
| 91 | Physalin F | Steroid | 2.2 µM (W2) |  | *Physalis angulata* | 315 |
| 92 | Physalin G | Steroid | 6.7 µM (W2) |  | *Physalis angulata* | 315 |
| 93 | Dehydrojuliprosopine (New) | Indolizidine alkaloid | 560 ng/ml (D6)  0.89 µM  600 ng/ml (W2)  0.96 µM |  | *Prosopis glandulosa var. glandulosa* (leaf) | 316 |
| 94 | Juliprosine | Indolizidine alkaloid | 170 ng/ml (D6)  0.27 µM  150 ng/ml (W2)  0.23 µM |  | *Prosopis glandulosa var. glandulosa* (leaf) | 316 |
| 95 | Salvadione C (New) | Triterpene | 1.43 µM (K1) |  | *Salvia hydrangea* (aerial part) | 317 |
| 96 | Perovskone B (New) | Triterpene | 0.18 µM (K1) |  | *Salvia hydrangea* (aerial part) | 317 |
| 97 | Miltirone | Tanshinone diterpene | 5.0 µM (K1) |  | *Salvia miltiorrhiza* (root) | 318 |
| 98 | Tanshinone IIa | Tanshinone diterpene | 4.0 µM (K1) |  | *Salvia miltiorrhiza* (root) | 318 |
| 99 | 1,2 dihydrotanshinquinone | Tanshinone diterpene | 5.3 µM (K1) |  | *Salvia miltiorrhiza* (root) | 318 |
| 100 | Methylenetanshinquinone | Tanshinone diterpene | 6.3 µM (K1) |  | *Salvia miltiorrhiza* (root) | 318 |
| 101 | 1-oxomiltirone | Tanshinone diterpene | 30 µM (K1) |  | *Salvia miltiorrhiza* (root) | 318 |
| 102 | 11-hydroxymiltiodiol | Tanshinone diterpene | 30 µM (K1) |  | *Salvia miltiorrhiza* (root) | 318 |
| 103 | Tanshinone I | Tanshinone diterpene | 7.2 µM (K1) |  | *Salvia miltiorrhiza* (root) | 318 |
| 104 | Methyltanshinonate | Tanshinone diterpene | 4.1 µM (K1) |  | *Salvia miltiorrhiza* (root) | 318 |
| 105 | Cryptotanshinone | Tanshinone diterpene | 16.1 µM (K1) |  | *Salvia miltiorrhiza* (root) | 318 |
| 106 | (z)-Non-2-en-6,8-diynoic acid isobutylamide | N-alkylamide | 54.03 µg/ml (D10)  266.15 µM |  | *Spilanthes acmella* (flower) | 319 |
| 107 | Spilanthol | N-alkylamide | 26.43 µg/ml (D10)  119.59 µM |  | *Spilanthes acmella* (flower) | 319 |
| 108 | (2E)-N-isobutylundeca-2-ene-8, 10-diynamide | N-alkylamide | 29.34 µg/ml (D10)  127.01 µM |  | *Spilanthes acmella* (flower) | 319 |
| 109 | Spilanthic acid 2-methylbutylamide | N-alkylamide | 33.73 µg/ml (D10)  143.53 µM |  | *Spilanthes acmella* (flower) | 319 |
| 110 | 5,8,4’-trihydroxy-3,7,3’-trimethoxy  Flavone | Flavonoid | 7.5 µM (D6)  6.4 µM (W2) |  | *Zanthoxylum monophyllum* (leaf) | 320 |
| 111 | Mauritine M (New) | Cyclopeptide alkaloid | 3.7 µM (K1) |  | *Ziziphus mauritiana* (root) | 321 |
| 112 | Nummularine H | Cyclopeptide alkaloid | 4.2 µM (K1) |  | *Ziziphus mauritiana* (root) | 321 |
| 113 | Nummularine B | Cyclopeptide alkaloid | 10.3 µM (K1) |  | *Ziziphus mauritiana* (root) | 321 |
| 114 | Hemsine A | Cyclopeptide alkaloid | 7.3 µM (K1) |  | *Ziziphus mauritiana* (root) | 321 |
| 115 | Amathamide H (New) | Pyrrolidine alkaloid | 10.2 µM (Dd2)  28.0 µM (3D7) | Bryozoan | *Amathia wilsoni* | 322 |
| 116 | Amathamide C | Pyrrolidine alkaloid | 8.0 µM (Dd2)  14.9 µM (3D7) | Bryozoan | *Amathia wilsoni* | 322 |
| 117 | Longirostrerone A (New) | Azaphilone | 0.63 µM (K1) | Fungus | Chaetomium longirostre | 323 |
| 118 | Longirostrerone B (New) | Azaphilone | 3.73 µM (K1) | Fungus | Chaetomium longirostre | 323 |
| 119 | Longirostrerone C (New) | Azaphilone | 0.62 µM (K1) | Fungus | Chaetomium longirostre | 323 |
| 120 | Pestalopyrone | 2-pyrone | 37 µM (Dd2) | Endophytic fungus | *Delitzchia winteri* | 324 |
| 121 | Haliclonacyclamine A | Piperidine alkaloid | 0.33 µg/ml (3D7)  0.70 µM  0.052 µg/ml (FcB1)  0.11 µM  45% suppression of P. vinckei parasite in infected mice at 10mg/kg | Sponge | *Haliclona sp.* | 325 |
| 122 | Hypocrellol D (New) | Lanostane triterpene | 8.0 µM (K1) | Fungus | *Hypocrella sp.* BCC 14524 | 326 |
| 123 | 3β,7β,15α,22-Tetrahydroxyhopane (New) | Hopane triterpene | 12.0 µM (K1) | Fungus | *Hypocrella sp.* BCC 14524 | 326 |
| 124 | 3β-Acetoxy-7β,15α,22-trihydroxyhopane(New) | Hopane triterpene | 5.2 µM (K1) | Fungus | *Hypocrella sp.* BCC 14524 | 326 |
| 125 | Lagunamide C (New) | Cyclodepsipeptide | 0.29 µM (NF54) | Cyanobacterium | *Lyngbya majuscula* | 327 |
| 126 | Marinacarboline A (New) | β-carboline  alkaloid | 36.03 µM (3D7)  1.92 µM (Dd2) | Actinobacteria | *Marinactinospora thermotolerans* SCSIO 00652 | 328 |
| 127 | Marinacarboline B (New) | β-carboline  alkaloid | 16.65 µM (3D7)  15.59 µM (Dd2) | Actinobacteria | *Marinactinospora thermotolerans* SCSIO 00652 | 328 |
| 128 | Marinacarboline C (New) | β-carboline  alkaloid | 3.09 µM (3D7)  3.38 µM (Dd2) | Actinobacteria | *Marinactinospora thermotolerans* SCSIO 00652 | 328 |
| 129 | Marinacarboline D (New) | β-carboline  alkaloid | 5.39 µM (3D7)  3.59 µM (Dd2) | Actinobacteria | *Marinactinospora thermotolerans* SCSIO 00652 | 328 |
| 130 | 13-*N*-demethyl-methylpendolmycin (New) | Indolactam alkaloid | 20.75 µM (3D7)  18.67 µM (Dd2) | Actinobacteria | *Marinactinospora thermotolerans* SCSIO 00652 | 328 |
| 131 | Methylpendolmycin-14-*O*-*α*-glucoside (New) | Indolactam alkaloid | 10.43 µM (3D7)  5.03 µM (Dd2) | Actinobacteria | *Marinactinospora thermotolerans* SCSIO 00652 | 328 |
| 132 | Cercosporin | Perylenequinone | 1.03 µM (W2) | Endophytic fungus | *Mycosphaerella sp.* F2140 | 329 |
| 133 | Kabiramide J (New) | Trisoxazole macrolide | 0.31 µM (K1) | Sponge | *Pachastrissa nux* | 330 |
| 134 | Kabiramide K (New) | Trisoxazole macrolide | 0.39 µM (K1) | Sponge | *Pachastrissa nux* | 330 |
| 135 | Kabiramide B | Trisoxazole macrolide | 1.67 µM (K1) | Sponge | *Pachastrissa nux* | 330 |
| 136 | Kabiramide C | Trisoxazole macrolide | 4.79 µM (K1) | Sponge | *Pachastrissa nux* | 330 |
| 137 | Kabiramide D | Trisoxazole macrolide | 1.87 µM (K1) | Sponge | *Pachastrissa nux* | 330 |
| 138 | Puberulic acid | Tropolone | 10.0 ng/ml (K1)  50.47 nM  10.0 ng/ml (FCR3)  50.47 nM  69% suppression of parasite in P. berghei infected mice at 2mg/kg | Fungus | *Penicillium sp.* FKI-4410 | 331 |
| 139 | Viticolin A (New) | Tropolone | 9.92 µg/ml (K1)  54.4 µM | Fungus | *Penicillium*  *sp.* FKI-4410 | 331 |
| 140 | Viticolin B (New) | Tropolone | 0.84 µg/ml (K1)  3.95 µM  0.54 µg/ml (FCR3)  2.54 µM | Fungus | *Penicillium*  *sp.* FKI-4410 | 331 |
| 141 | Stipitatic acid | Tropolone | 7.07 µg/ml (K1)  31.2 µM | Fungus | *Penicillium sp.* FKI-4410 | 331 |
| 142 | Phomoarcherin B (New) | Sesquiterpene | 0.79 µg/ml (K1)  2.59 µM | Endophytic Fungus | *Phomopsis archeri* | 332 |
| 143 | Psammaplysin H (New) | Bromotyrosine alkaloid | 0.41 µM (3D7) | Marine sponge | *Pseudoceratina sp.* (G319257) | 333 |
| 144 | Psammaplysin G | Bromotyrosine alkaloid | 5.22 µM (3D7) | Marine sponge | *Pseudoceratina sp.* (G319257) | 333 |
| 145 | Psammaplysin F | Bromotyrosine alkaloid | 1.92 µM (3D7) | Marine sponge | *Pseudoceratina sp.* (G319257) | 333 |
| 146 | Opacaline A (New) | β-carboline alkaloid | 2.5 µM (K1) | Ascidian | *Pseudodistoma opacum* | 334 |
| 147 | Opacaline B (New) | β-carboline alkaloid | 4.5 µM (K1) | Ascidian | *Pseudodistoma opacum* | 334 |
| 148 | Sterostrein A (New) | Sesquiterpene | 2.3 µg/ml (K1)  5.32 µM | Basidiomycete m ushroom | *Stereum ostrea BCC 22955* | 335 |
| 149 | Purealidin B | Bromotyrosine alkaloid | 23.2% inhibition at 5µM (NF54) | Marine sponge | *Verongula rigida* | 336 |

2010

| 1 | Eupalestin | Flavonoid | 10.99 µM (K1) | Plant | *Ageratum conyzoides* (aerial part) | 337 |
| --- | --- | --- | --- | --- | --- | --- |
| 2 | 5,6,7,5’-tetramethoxy-3’,4’-methylene  dioxyflavone | Flavonoid | 11.04 µM (K1) | Plant | *Ageratum conyzoides* (aerial part) | 337 |
| 3 | 5,6,7,3’,4’,5’-Hexamethoxyflavone | Flavonoid | 7.43 µM (K1) | Plant | *Ageratum conyzoides* (aerial part) | 337 |
| 4 | Ageconyflavone C | Flavonoid | 9.25 µM (K1) | Plant | *Ageratum conyzoides* (aerial part) | 337 |
| 5 | Shuangancistrotectorine A (New) | Naphthylisoquinoline alkaloid | 0.052 µM (K1) | Plant | *Ancistrocladus tectorius* (twigs) | 338 |
| 6 | Shuangancistrotectorine B (New) | Naphthylisoquinoline alkaloid | 0.076 µM (K1) | Plant | *Ancistrocladus tectorius* (twigs) | 338 |
| 7 | Shuangancistrotectorine C (New) | Naphthylisoquinoline alkaloid | 0.234 µM (K1) | Plant | *Ancistrocladus tectorius* (twigs) | 338 |
| 8 | Shuangancistrotectorine D (New) | Naphthylisoquinoline alkaloid | 0.085 µM (K1) | Plant | *Ancistrocladus tectorius* (twigs) | 338 |
| 9 | Shuangancistrotectorine E (New) | Naphthylisoquinoline alkaloid | 1.05 µM (K1) | Plant | *Ancistrocladus tectorius* (twigs) | 338 |
| 10 | (+)-4′-Decanoyl-*cis*-khellactone | Pyranocoumarin | 1.5 µM (D10) | Plant | *Agelica purpuraefolia (rhizome)* | 339 |
| 11 | (+)-3′-Decanoyl-*cis*-khellactone | Pyranocoumarin | 2.4 µM (D10) | Plant | *Agelica purpuraefolia (rhizome)* | 339 |
| 12 | Styracifolin A (New) | Flavonoid | 5.7 µM (FcB1) | Plant | *Artocarpus styracifolius* (stem bark) | 340 |
| 13 | Styracifolin B (New) | Flavonoid | 1.12 µM (FcB1) | Plant | *Artocarpus styracifolius* (stem bark) | 340 |
| 14 | Artoheterophyllin B | Flavonoid | 2.8 µM (FcB1) | Plant | *Artocarpus styracifolius* (stem bark) | 340 |
| 15 | Artoheterophyllin C | Flavonoid | 13.7 µM (FcB1) | Plant | *Artocarpus styracifolius* (stem bark) | 340 |
| 16 | Artonin A | Flavonoid | 4.9 µM (FcB1) | Plant | *Artocarpus styracifolius* (stem bark) | 340 |
| 17 | Artonin B | Flavonoid | 1.56 µM (FcB1) | Plant | *Artocarpus styracifolius* (stem bark) | 340 |
| 18 | Artonin F | Flavonoid | 2.2 µM (FcB1) | Plant | *Artocarpus styracifolius* (stem bark) | 340 |
| 19 | Heterophyllin | Flavonoid | 1.2 µM (FcB1) | Plant | *Artocarpus styracifolius* (stem bark) | 340 |
| 20 | Kaurenic acid | Diterpene | 18.0 µM (D6)  17.6 µM (W2) |  | *Aspilia pruliseta* (aerial part) | 341 |
| 21 | 15-(2Z)-[(2-methyl-1-oxo-2-butenyl)  oxy]-4α,15β-kaur-16-en-18-oic acid | Diterpene | 14.3 µM (D6)  18.4 µM (W2) |  | *Aspilia pruliseta* (aerial part) | 341 |
| 22 | 16,17-epoxy-15-[(2-methyl-1-oxo-2-  butenyl)oxy]-4α,15β(Z)-kauran-18-oic acid | Diterpene | 23.4 µM (D6)  17.5 µM (W2) |  | *Aspilia pruliseta* (aerial part) | 341 |
| 23 | ent-15β-senecioyloxy-16,17-epoxy-kauran-18-oic acid (New) | Diterpene | 23.4 µM (D6) |  | *Aspilia pruliseta* (aerial part) | 341 |
| 24 | 1β,6α-dihydroxy-4(15)-eudesmene | Sesquiterpene | 42.1 µM (D10) |  | *Carpesium divaricatum* (whole plant) | 342 |
| 25 | β-dictyopterol | Sesquiterpene | 13.1 µM (D10) |  | *Carpesium divaricatum* (whole plant) | 342 |
| 26 | 2-isopropenyl-6-acetyl-8-methoxy-1,3-benzodioxin-4-one |  | 2.3 µM (D10) |  | *Carpesium divaricatum* (whole plant) | 342 |
| 27 | 5-acetyl-6-hydroxy-2αisopropenyl-3β-methoxy-2,3H-benzofuran | Benzofuran | 34.1 µM (D10) |  | *Carpesium divaricatum* (whole plant) | 342 |
| 28 | 3β-acetoxy6-acetyl-5-hydroxy-2α-isopropenyl-2,3H-benzofuran | Benzofuran | 23.1 µM (D10) |  |  | 342 |
| 29 | Tormentic acid | Triterpene | 58% suppression of parasite in P. berghei infected mice at 15mg/kg |  | *Cecropia pachystachya* (root) | 343 |
| 30 | O-methylmukonal | Carbazole alkaloid | MIC 6.74 µg/ml (K1)  29.94 µM |  | *Clausena excavate* (stem) | 344 |
| 31 | Mukonal | Carbazole alkaloid | 3.27 µg/ml (K1)  15.49 µM |  | *Clausena harmandiana* (root) | 345 |
| 32 | 7-Methoxymukonal | Carbazole alkaloid | 2.94 µg/ml (K1)  12.07 µM |  | *Clausena harmandiana (root)* | 345 |
| 33 | Taxodione | Abietane diterpenoid | 1.2 µg/ml (D6)  3.81 µM  1.2 µg/ml (W2)  3.81 µM |  | *Clerodendrum eriophyllum* (root) | 346 |
| 34 | 6-hydroxysalvinolone | Abietane diterpenoid | 1.8 µg/ml (D6)  5.44 µM  2.5 µg/ml (W2)  7.56 µM |  | *Clerodendrum eriophyllum* (root) | 346 |
| 35 | 6,11,12,16-tetrahydroxy-5,8,11,13-abietatetra-en-7-one | Abietane diterpenoid | 3.0 µg/ml (D6)  8.66 µM  4.8 µg/ml (W2)  13.85 µM |  | *Clerodendrum eriophyllum* (root) | 346 |
| 36 | Protopine | Isoquinoline alkaloid | 4.25 µM (TM4)  4.29 µM (K1) |  | *Corydalis calliantha* (whole plant) | 347 |
| 37 | Cheilanthifoline | Isoquinoline alkaloid | 2.78 µM (TM4)  3.76 µM (K1) |  | *Corydalis calliantha* (whole plant) | 347 |
| 38 | Norbergenin | Phenolic glycoside | 12.7µg/ml (K1)  40.41 µM |  | *Diospyros sanza-minika* (Stem bark) | 348 |
| 39 | 4-*O*-galloylnorbergenin | Phenolic glycoside | 3.9 µg/ml (K1)  8.36 µM |  | *Diospyros sanza-minika* (Stem bark) | 348 |
| 40 | 11-*O*-*p*-hydroxybenzoylnorbergenin | Phenolic glycoside | 4.9 µg/ml (K1)  11.28 µM |  | *Diospyros sanza-minika*  (Stem bark) | 348 |
| 41 | 4-*O*-(3′-methylgalloyl)norbergenin | Phenolic glycoside | 0.6 µg/ml (K1)  1.24 µM |  | *Diospyros sanza-minika*  (Stem bark) | 348 |
| 42 | 4-*O*-syringoylnorbergenin | Phenolic glycoside | 18.0 µg/ml (K1)  36.40 µM |  | *Diospyros sanza-minika*  (Stem bark) | 348 |
| 43 | 7-*O*-α-d-glucopyranosyl-3,4′-dihydroxy-3′-(4-hydroxy-3-methylbutyl)-5,6-dimethoxyflavone (New) | Flavonoid glycoside | 12.3 µM (D6)  12.9 µM (W2) |  | *Duranta repens* (whole plant) | 349 |
| 44 | 7-*O*-α-d-glucopyranosyl(6′′′-*p*-hydroxy  cinnamoyl)-3,4′-dihydroxy-3′-(4-hydroxy-3-methylbutyl)-5,6-dimethoxyflavone (New) | Flavonoid glycoside | 9.7 µM (D6)  9.2 µM (W2) |  | *Duranta repens* (whole plant) | 349 |
| 45 | 3,7,4′-trihydroxy-3′-(4-hydroxy-3-methylbutyl)-5,6-dimethoxyflavone | Flavonoid | 5.2 µM (D6)  5.9 µM (W2) |  | *Duranta repens* (whole plant) | 349 |
| 46 | 3,7-dihydroxy-3′-(4-hydroxy-3-methylbutyl)-5,6,4′-trimethoxyflavone | Flavonoid | 6.4 µM (D6)  7.0 µM (W2) |  | *Duranta repens* (whole plant) | 349 |
| 47 | 5,7-dihydroxy-3′-(2-hydroxy-3-methyl-3-butenyl)-3,6,4′-trimethoxyflavone | Flavonoid | 6.9 µM (D6)  6.7 µM (W2) |  | *Duranta repens* (whole plant) | 349 |
| 48 | 3,7-dihydroxy-3′-(2-hydroxy-3-methyl-3-buten-yl)-5,6,4′-trimethoxyflavone | Flavonoid | 8.6 µM (D6)  8.3 µM (W2) |  | *Duranta repens* (whole plant) | 349 |
| 49 | 7-*O*-α-d-glucopyranosyl-3,5-dihydroxy-3′-(4′′-acetoxy-3′′-methylbutyl)-6,4′-dimethoxyflavone | Flavonoid glycoside | 13.5 µM (D6)  13.1 µM (W2) |  | *Duranta repens* (whole plant) | 349 |
| 50 | Pyrocatechol | Phenolic | 27.0 µM (W2) |  | *Flacourtia indica* (aerial part) | 350 |
| 51 | Homaloside D | Phenolic glycoside | 20.0 µM (W2) |  | *Flacourtia indica* (aerial part) | 350 |
| 52 | Poliothrysoside | Phenolic glycoside | 7.0 µM (W2) |  | *Flacourtia indica* (aerial part) | 350 |
| 53 | Isoborreverine | Indole alkaloid | 0.24 µM (3D7)  0.16 µM (FCR3)  0.47 µM (HB3)  0.33 µM (K1) |  | *Flindersia amboinensis* | 351 |
| 54 | Dimethylisoborreverine | Indole alkaloid | 0.22 µM (3D7)  0.02 µM (FCR3)  0.81 µM (HB3)  0.06 µM (K1) |  | *Flindersia amboinensis* | 351 |
| 55 | Flinderole A | Indole alkaloid | 0.75 µM (3D7)  0.92 µM (FCR3)  1.16 µM (HB3)  1.61 µM (K1) |  | *Flindersia amboinensis* | 351 |
| 56 | Flinderole B | Indole alkaloid | 0.21 µM (3D7)  0.11 µM (FCR3)  0.64 µM (HB3)  0.08 µM (K1) |  | *Flindersia amboinensis* | 351 |
| 57 | Flinderole C | Indole alkaloid | 1.10 µM (3D7)  0.36 µM (FCR3)  1.17 µM (HB3)  0.33 µM (K1) |  | *Flindersia amboinensis* | 351 |
| 58 | Liriodenine | Isoquinoline alkaloid | 18.79 µM (3D7)  25.85 µM (FCR3)  12.31 µM (HB3)  17.65 µM (K1) |  | *Stephania zippeliana* | 351 |
| 59 | Xylopine | Isoquinoline alkaloid | 1.85 µM (3D7)  3.50 µM (FCR3)  1.06 µM (HB3)  3.12 µM (K1) |  | *Stephania zippeliana* | 351 |
| 60 | Voacamine | Indole alkaloid | 2.13 µM (3D7)  1.64 µM (FCR3)  1.84 µM (HB3)  1.72 µM (K1) |  | *Voacanga papuana* | 351 |
| 61 | Gomphostenin (New) | Clerodane diterpene | 38.2 µg/ml (MRC-02, 114.99 µM)  37.7 µg/ml (113.48 µM)  80.6% suppression of parasite in P. berghei infected mice at 200mg/kg |  | *Gomphostemma niveum* (leaf) | 352,353 |
| 62 | Gomphostenin A (New) | Clerodane diterpene | 3.4 µg/ml (MRC-02, 9.08 µM)  3.2 µg/ml (8.55 µM)  92.65% suppression of parasite in P. berghei infected mice at 200mg/kg |  | *Gomphostemma niveum* (leaf) | 352,353 |
| 63 | Oxyonoseriolide (New) | Lindenane  Sesquiterpene | 107.7 µM (FcB1) |  | *Hedyosmum angustifolium* (bark) | 354 |
| 64 | Onoseriolide | Lindenane  Sesquiterpene | 12.5 µM (FcB1) |  | *Hedyosmum angustifolium (bark)* | 354 |
| 65 | Otogirin | Phloroglucinol derivative | >50 µM (D10) |  | *Hypericum erectum* (whole plant) | 355 |
| 66 | Otogirone | Phloroglucinol derivative | 5.6 µM (D10) |  | *Hypericum erectum (whole plant)* | 355 |
| 67 | Erectquione A | Phloroglucinol derivative | 11.2 µM (D10) |  | *Hypericum erectum (whole plant)* | 355 |
| 68 | Erectquione B | Phloroglucinol derivative | 7.2 µM (D10) |  | *Hypericum erectum (whole plant)* | 355 |
| 69 | Erectquione C | Phloroglucinol derivative | 13.4 µM (D10) |  | *Hypericum erectum (whole plant)* | 355 |
| 70 | Jacaglabroside A (New) | Phenylethanoid | 1.02 µg/ml (K1)  1.42 µM |  | *Jacaranda glabra* (leaf) | 356 |
| 71 | Jacaglabroside B (New) | Phenylethanoid | 0.56 µg/ml (K1)  0.93 µM |  | *Jacaranda glabra* (leaf) | 356 |
| 72 | Jacaglabroside C(New) | Phenylethanoid | 0.56 µg/ml (K1)  0.76 µM |  | *Jacaranda glabra* (leaf) | 356 |
| 73 | Jacaglabroside D (New) | Phenylethanoid | 0.55 µg/ml (K1)  0.75 µM |  | *Jacaranda glabra* (leaf) | 356 |
| 74 | 2- acetylnaphtho[2,3-b]furan-4,9-dione | Naphthoquinone | 0.11 µg/ml (K1)  0.46 µM  0.16 µg/ml (W2)  0.67 µM |  | *Markhamia tomentosa* (stem bark) | 357 |
| 75 | 2-acetyl-6-methoxynaphtho[2,3-b]furan-4,9-dione | Naphthoquinone | 0.44 µg/ml (K1)  1.6 µM  0.93 µg/ml (W2)  3.46 µM |  | *Markhamia tomentosa* (stem bark) | 357 |
| 76 | Pomolic acid | Triterpene | 3.47 µg/ml (K1)  7.34 µM |  | *Markhamia tomentosa* (stem bark) | 357 |
| 77 | 3-acetylpomolic acid | Triterpene | 2.10 µg/ml (K1)  4.08 µM |  | *Markhamia tomentosa* (stem bark) | 357 |
| 78 | Balsaminol C (New) | Cucurbitane triterpenoid | 19.6 µM (3D7)  22.4 µM (Dd2) |  | *Momordica balsamina* (aerial part) | 358 |
| 79 | Balsaminol D (New) | Cucurbitane triterpenoid | 25.9 µM (3D7)  45.6 µM (Dd2) |  | *Momordica balsamina* (aerial part) | 358 |
| 80 | Balsaminol E (New) | Cucurbitane triterpenoid | 20.4 µM (3D7)  19.6 µM (Dd2) |  | *Momordica balsamina* (aerial part) | 358 |
| 81 | Balsaminagenin A | Cucurbitane triterpenoid | 19.1 µM (3D7)  27.3 µM (Dd2) |  | *Momordica balsamina* (aerial part) | 358 |
| 82 | Balsaminagenin B | Cucurbitane triterpenoid | 18.7 µM (3D7)  19.2 µM (Dd2) |  | *Momordica balsamina* (aerial part) | 358 |
| 83 | Balsaminapentaol A | Cucurbitane triterpenoid | 14.6 µM (3D7)  33.0 µM (Dd2) |  | *Momordica balsamina* (aerial part) | 358 |
| 84 | Balsaminol A | Cucurbitane triterpenoid | 17.1 µM (3D7)  23.5 µM (Dd2) |  | *Momordica balsamina* (aerial part) | 358 |
| 85 | Balsaminol B | Cucurbitane triterpenoid | 15.2 µM (3D7)  17.9 µM (Dd2) |  | *Momordica balsamina* (aerial part) | 358 |
| 86 | Balsaminoside A | Cucurbitane triterpenoid | 4.6 µM (3D7)  4.0 µM (Dd2) |  | *Momordica balsamina* (aerial part) | 358 |
| 87 | Cucurbalsaminol A | Cucurbitane triterpenoid | 13.2 µM (3D7)  17.6 µM (Dd2) |  | *Momordica balsamina* (aerial part) | 358 |
| 88 | Cucurbalsaminol B | Cucurbitane triterpenoid | 17.7 µM (3D7)  28.3 µM (Dd2) |  | *Momordica balsamina* (aerial part) | 358 |
| 89 | Karavilagenin E | Cucurbitane triterpenoid | 7.4 µM (3D7)  8.2 µM (Dd2) |  | *Momordica balsamina* (aerial part) | 358 |
| 90 | Cucurbita-5,23(*E*)-diene-3β,7β,25-triol | Cucurbitane triterpenoid | 30.6 µM (3D7)  50.1 µM (Dd2) |  | *Momordica balsamina* (aerial part) | 358 |
| 91 | 5-carboxystrictosidine | Indole alkaloid | 41.2 µM |  | *Nauclea pobeguinii* (stem bark) | 359 |
| 92 | 19-O-methylangustoline | Indole alkaloid | 26.5 µM |  | *Nauclea pobeguinii* (stem bark) | 359 |
| 93 | Liquiritigeninyl-(I-3,II-3)-naringenin (New) | Flavonoid | 32.9 µM (K1) |  | *Ormocarpum kirkii* (root) | 360 |
| 94 | 5,5’’-Di-O-methyldiphysin (New) | Flavonoid | 21.1 µM (K1) |  | *Ormocarpum kirkii* (root) | 360 |
| 95 | 4’’-Hydroxydiphysolone (New) | Flavonoid | 23.7 µM (K1) |  | *Ormocarpum kirkii* (root) | 360 |
| 96 | (+)-Chamaejasmin | Flavonoid | 15.0 µM (K1) |  | *Ormocarpum kirkii* (root) | 360 |
| 97 | Isochamaejasmin | Flavonoid | 7.3 µM (K1) |  | *Ormocarpum kirkii* (root) | 360 |
| 98 | (I-3,II-3)-Biliquiritigenin | Flavonoid | 32.3 µM (K1) |  | *Ormocarpum kirkii* (root) | 360 |
| 99 | Diphysin | Flavonoid | 39.5 µM (K1) |  | *Ormocarpum kirkii* (root) | 360 |
| 100 | Pentadexanthone (New) | Xanthone | 3.0 µM (W2) |  | *Pentadesma butyracea* (fruit) | 361 |
| 101 | Cratoxylone | Xanthone | 2.89 µM (W2) |  | *Pentadesma butyracea* (fruit) | 361 |
| 102 | α-mangostin | Xanthone | 2.77 µM (W2) |  | *Pentadesma butyracea* (fruit) | 361 |
| 103 | Garcinone E | Xanthone | 0.41 µM (W2) |  | *Pentadesma butyracea* (fruit) | 361 |
| 104 | Kaousine (New) | Piperidinone alkaloid | 82.0 µM (W2) |  | *Piper capense* (aerial part) | 362 |
| 105 | Z-antiepilepsirine | Piperidine alkaloid | 27.0 µM (W2) |  | *Piper capense* (aerial part) | 362 |
| 106 | Apigenine dimethylether | Flavonoid | 42.0 µM (W2) |  | *Piper capense* (aerial part) | 362 |
| 107 | Quassin | Quassinoid | 0.15 µM(MRC-pf-20) |  | *Quassia amara* (bark) | 363 |
| 108 | Neo-quassin | Quassinoid | 0.10 µM (MRC-pf-303) |  | *Quassia amara* (bark) | 363 |
| 109 | 1,2-Dimethoxy-6-methyl-9,10-anthraquinone (New) | Anthraquinone | 1.10 µM (3D7) |  | *Rennellia elliptica* (root) | 364 |
| 110 | Nordamnacanthal | Anthraquinone | 72.46 µM (3D7) |  | *Rennellia elliptica* (root) | 364 |
| 111 | 2-formyl-3-hydroxy-9,10-anthraquinone | Anthraquinone | 0.63 µM (3D7) |  | *Rennellia elliptica* (root) | 364 |
| 112 | Damnacanthal | Anthraquinone | 51.28 µM (3D7) |  | *Rennellia elliptica* (root) | 364 |
| 113 | Lucidin-*ω*-methyl ether | Anthraquinone | 2.10 µM (3D7) |  | *Rennellia elliptica* (root) | 364 |
| 114 | 3-hydroxy-2-methyl-9,10-anthraquinone | Anthraquinone | 0.34 µM (3D7) |  | *Rennellia elliptica* (root) | 364 |
| 115 | 1,5,6-Trihydroxy-3-methoxy-7-geranylxanthone (New) | Xanthone | 10.5 µM (FcB1) |  | *Rheedia acuminata* (bark) | 365 |
| 116 | 2-(1’,1’-Dimethylprop-2’-enyl)-1,4,5-Trihydroxyxanthone | Xanthone | 15.1 µM (FcB1) |  | *Rheedia acuminata* (bark) | 365 |
| 117 | Pyrojacareubine | Xanthone | 11.4 µM (FcB1) |  | *Rheedia acuminata* (bark) | 365 |
| 118 | Isogarcinol | Phloroglucinol derivative | 3.5 µM (FcB1) |  | *Rheedia acuminata* (bark) | 365 |
| 119 | 7-epi-Isogarcinol | Phloroglucinol derivative | 3.2 µM (FcB1) |  | *Rheedia acuminata* (bark) | 365 |
| 120 | Betulin | Triterpene | 3.2 µg/ml (D10)  7.22 µM |  | *Schefflera umbellifera* (leaf) | 366 |
| 121 | 3α-(1-methylitaconyl)-6β-senecioyloxytropane (New) | Tropane alkaloid | 22.8 µM (K1) |  | *Schizanthus tricolor* (aerial part) | 367 |
| 122 | 3α-(1-methylmesaconyl)-6β-senecioyloxytropane | Tropane alkaloid | 63.5 µM (K1) |  | *Schizanthus tricolor* (aerial part) | 367 |
| 123 | 3α-(1-methylmesaconyl)-6β-angeloyloxytropane | Tropane alkaloid | 24.8 µM (K1) |  | *Schizanthus tricolor* (aerial part) | 367 |
| 124 | 3α-(1-methylmesaconyl)-6β-tigloyloxytropane | Tropane alkaloid | 36.0 µM (K1) |  | *Schizanthus tricolor* (aerial part) | 367 |
| 125 | Solanine | Steroidal glycoalkaloid | 41.3% suppression of parasite in P. yoelii infected mice at 7.5mg/kg |  | *Solanum tuberosum* (aerial part) | 368 |
| 126 | Solasonine | Steroidal glycoalkaloid | 57.47% suppression of parasite in P. yoelii infected mice at 7.5mg/kg |  | *Solanum*  *nigrum* (fruit) | 368 |
| 127 | Solamargine | Steroidal glycoalkaloid | 64.89% suppression of parasite in P. yoelii infected mice at 7.5mg/kg |  | *Solanum*  *nigrum* (fruit) | 368 |
| 128 | Tomatine | Steroidal glycoalkaloid | 65.25% suppression of parasite in P. yoelii infected mice at 7.5mg/kg |  | *Lycopersicon esculentum* (fruit) | 368 |
| 129 | Chaconine | Steroidal glycoalkaloid | 71.38% suppression of parasite in P. yoelii infected mice at 7.5mg/kg |  | *Solanum tuberosum* (aerial part) | 368 |
| 130 | 2-norcepharanthine | Isoquinoline alkaloid | 0.3 µM (W2) |  | *Stephania rotunda* (tuber) | 369 |
| 131 | Cepharanoline | Isoquinoline alkaloid | 0.2 µM (W2) |  | *Stephania rotunda* (tuber) | 369 |
| 132 | Fangchinoline | Isoquinoline alkaloid | 0.3 µM (W2) |  | *Stephania rotunda* (tuber) | 369 |
| 133 | 7-epi-Isogarcinol | Benzophenone | 3.2 µM (FcB1) |  | *Symphonia globulifera* (root bark) | 370 |
| 134 | 14-Deoxy-7-epi-isogarcinol (New) | Benzophenone | 2.5 µM (FcB1) |  | *Symphonia globulifera* (root bark) | 370 |
| 135 | Symphonone A (New) | Benzophenone | 2.8 µM (FcB1) |  | *Symphonia globulifera* (root bark) | 370 |
| 136 | Symphonone B (New) | Benzophenone | 3.3 µM (FcB1) |  | *Symphonia globulifera* (root bark) | 370 |
| 137 | Symphonone C (New) | Benzophenone | 2.6 µM (FcB1) |  | *Symphonia globulifera* (root bark) | 370 |
| 138 | 7-epi-Coccinone B (New) | Benzophenone | 3.3 µM (FcB1) |  | *Symphonia globulifera* (root bark) | 370 |
| 139 | Symphonone D (New) | Benzophenone | 2.1 µM (FcB1) |  | *Symphonia globulifera* (root bark) | 370 |
| 140 | Symphonone E (New) | Benzophenone | 2.7 µM (FcB1) |  | *Symphonia globulifera* (root bark) | 370 |
| 141 | 7-epi-Garcinol | Benzophenone | 10.1 µM (FcB1) |  | *Symphonia globulifera* (root bark) | 370 |
| 142 | Symphonone F (New) | Benzophenone | 3.2 µM (FcB1) |  | *Symphonia globulifera* (root bark) | 370 |
| 143 | Symphonone G (New) | Benzophenone | 2.1 µM (FcB1) |  | *Symphonia globulifera* (root bark) | 370 |
| 144 | Symphonone H (New) | Benzophenone | 3.0 µM (FcB1) |  | *Symphonia globulifera* (root bark) | 370 |
| 145 | Symphonone I (New) | Benzophenone | 6.7 µM (FcB1) |  | *Symphonia globulifera* (root bark) | 370 |
| 146 | 2-hydroxymethyl-non-3-ynoic acid 2-[2,2']-bithiophenyl-5-ethyl ester (New) | Thiophene derivative | 10.0 µg/ml (MRC-pf-2) 26.55 µM  20.0 µg/ml (MRC-pf-56) 53.11 µM |  | *Tagetes erecta* (root) | 371 |
| 147 | (+)-Preocoteine | Isoquinoline alkaloid | 1.3 µM (FcB1) |  | *Thalictrum flavum* (root) | 372 |
| 148 | (+)-O-Methylcassythine | Isoquinoline alkaloid | 8.6 µM (FcB1) |  | *Thalictrum flavum* (root) | 372 |
| 149 | (-)-Armepavine | Isoquinoline alkaloid | 11.4 µM (FcB1) |  | *Thalictrum flavum* (root) | 372 |
| 150 | (-)-Northalidasine | Isoquinoline alkaloid | 5.2 µM (FcB1) |  | *Thalictrum flavum* (root) | 372 |
| 151 | (-)-Northalrugosidine | Isoquinoline alkaloid | 4.3 µM (FcB1) |  | *Thalictrum flavum* (root) | 372 |
| 152 | (-)-Thalfoetidine | Isoquinoline alkaloid | 3.3 µM (FcB1) |  | *Thalictrum flavum* (root) | 372 |
| 153 | (-)-Northalfoetidine (New) | Isoquinoline alkaloid | 4.5 µM (FcB1) |  | *Thalictrum flavum* (root) | 372 |
| 154 | (-)-Thaligosidine | Isoquinoline alkaloid | 2.0 µM (FcB1) |  | *Thalictrum flavum* (root) | 372 |
| 155 | (+)-Thalicberine | Isoquinoline alkaloid | 4.2 µM (FcB1) |  | *Thalictrum flavum* (root) | 372 |
| 156 | Thaliglucinone | Isoquinoline alkaloid | 6.7 µM (FcB1) |  | *Thalictrum flavum* (root) | 372 |
| 157 | Berberine | Isoquinoline alkaloid | 0.9 µM (FcB1) |  | *Thalictrum flavum* (root) | 372 |
| 158 | Pseudoberberine | Isoquinoline alkaloid | 1.3 µM (FcB1) |  | *Thalictrum flavum* (root) | 372 |
| 159 | Muzigadiolide | Sesquiterpene | 7.2 µM (3D7)  7.3 µM (K1) |  | *Warburgia ugandensis* (stem bark) | 373 |
| 160 | 11α-hydroxymuzigadiolide | Sesquiterpene | 6.4 µM (3D7) |  | *Warburgia ugandensis* (stem bark) | 373 |
| 161 | Muzigadial | Sesquiterpene | 20.2 µM (K1) |  | *Warburgia ugandensis* (stem bark) | 373 |
| 162 | 6α,9α-dihydroxy-4(13),7-coloratadiene-  11,12-dial | Sesquiterpene | 32.9 µM (3D7)  11.0 µM (K1) |  | *Warburgia ugandensis* (stem bark) | 373 |
| 163 | Cinnamolide | Sesquiterpene | 43.8 µM (3D7)  23.7 µM (K1) |  | *Warburgia ugandensis* (stem bark) | 373 |
| 164 | cinnamolide-3β-acetate | Sesquiterpene | 34.5 µM (3D7) |  | *Warburgia ugandensis* (stem bark) | 373 |
| 165 | Mukaadial | Sesquiterpene | 6.4 µM (3D7)  7.9 µM (K1) |  | *Warburgia ugandensis* (stem bark) | 373 |
| 166 | Ugandensidial | Sesquiterpene | 10.6 µM (K1) |  | *Warburgia ugandensis* (stem bark) | 373 |
| 167 | 7α-hydroxy-8-drimen-11,12-olide | Sesquiterpene | 86.0 µM (3D7)  50.8 µM (K1) |  | *Warburgia ugandensis* (stem bark) | 373 |
| 168 | 7β-hydroxy-4(13),7-coloratadiene-11,12-olide | Sesquiterpene | 70.4 µM (3D7) |  | *Warburgia ugandensis* (stem bark) | 373 |
| 169 | (+)-7-Bromotrypargine (New) | β-carboline alkaloid | 3.5 µM (3D7)  5.4 µM (Dd2) | Marine sponge | *Ancorina sp.* | 374 |
| 170 | Aplidiopsamine A (New) | Pyrrolo-quinoline alkaloidn | 1.47 µM (3D7)  1.65 µM (Dd2) | Ascidian | *Aplidiopsis confluata* | 375 |
| 171 | 17(21)-hopene-6α,12β-diol (New) | Hopane triterpene | 15 µM (K1) | Fungus | *Aschersonia paraphysata* BCC 11964 | 376 |
| 172 | 17(21)-hopen-12-β-ol (New) | Hopane triterpene | >23 µM (K1) | Fungus | *Aschersonia paraphysata* BCC 11964 | 376 |
| 173 | Zeorinin | Hopane triterpene | >23 µM (K1) | Fungus | *Aschersonia paraphysata* BCC 11964 | 376 |
| 174 | Aschernaphthopyrone A | Naphthopyrone | 7.3 µM (K1) | Fungus | *Aschersonia paraphysata* BCC 11964 | 376 |
| 175 | Aschernaphthopyrone B | Naphthopyrone | >19 µM (K1) | Fungus | *Aschersonia paraphysata* BCC 11964 | 376 |
| 176 | Butyrolactone V | Butenolide | 7.9 µg/ml (K1)  17.94 µM | Fungus | *Aspergillus terreus* BCC 4651 | 377 |
| 177 | Hymenidin | Bromopyrrole alkaloid | 12.54 µg/ml (K1)  40.43 µM | Marine sponge | *Agelas sp.* | 378 |
| 178 | Dispacamide B | Bromopyrrole alkaloid | 1.34 µg/ml (K1)  4.10 µM | Marine sponge | *Agelas sp.* | 378 |
| 179 | Stevensine | Bromopyrrole alkaloid | 4.88 µg/ml (K1)  12.60 µM | Marine sponge | *Axinella verrucosa* | 378 |
| 180 | Spongiacidin B | Bromopyrrole alkaloid | 1.09 µg/ml (K1)  3.36 µM | Marine sponge | *Axinella verrucosa* | 378 |
| 181 | Longamide B | Bromopyrrole alkaloid | 7.46 µg/ml (K1)  21.19 µM | Marine sponge | *Agelas dispar* | 378 |
| 182 | Sceptrin | Bromopyrrole alkaloid | 11.08 µg/ml (K1)  17.86 µM | Marine sponge | *Agelas sp.* | 378 |
| 183 | Dibromopalau’amine | Bromopyrrole alkaloid | 1.48 µg/ml (K1)  2.65 µM | Marine sponge | *Axinella verrucosa* | 378 |
| 184 | Agelongine | Bromopyrrole alkaloid | 11.18 µg/ml (K1)  32.96 µM | Marine sponge | *Agelas longissima* | 378 |
| 185 | Bromophycolide R (New) | Macrolide | 1.7 µM | Red algea | *Callophycus serratus* | 379 |
| 186 | Bromophycolide S (New) | Macrolide | 0.9 µM | Red algea | *Callophycus serratus* | 379 |
| 187 | Bromophycolide T (New) | Macrolide | 8.4 µM | Red algea | *Callophycus serratus* | 379 |
| 188 | Bromophycolide U (New) | Macrolide | 2.1 µM | Red algea | *Callophycus serratus* | 379 |
| 189 | Callophycolide A (New) | Macrolide | 5.2 µM | Red algae | *Callophycus serratus* | 380 |
| 190 | Bromophycolide A | Macrolide | 0.7 µM | Red algae | *Callophycus serratus* | 380 |
| 191 | Debromophycolide A | Macrolide | >100.0 µM | Red algae | *Callophycus serratus* | 380 |
| 192 | β-Tocopherylhydroquinone | Hydroquinone | 190.0 µM | Algae | *Amphiroa crassa* | 380 |
| 193 | δ-Tocopherylhydroquinone | Hydroquinone | 220.0 µM | Algae | *Amphiroa crassa* | 380 |
| 194 | 9-Methoxystrobilurin A | β-methoxyacrylate | 0.39 µg/ml (K1)  1.35 µM | Fungus | *Favolaschia tonkinensis* BCC 18689 | 381 |
| 195 | 9-Methoxystrobilurin B (New) | β-methoxyacrylate | 0.30 µg/ml (K1)  0.85 µM | Fungus | *Favolaschia tonkinensis* BCC 18689 | 381 |
| 196 | 9-Methoxystrobilurin G (New) | β-methoxyacrylate | 0.03 µg/ml (K1)  0.06 µM | Fungus | *Favolaschia tonkinensis* BCC 18689 | 381 |
| 197 | Oudemansin A | β-methoxyacrylate | 3.0 µg/ml (K1)  10.33 µM | Fungus | *Favolaschia tonkinensis* BCC 18689 | 381 |
| 198 | Oudemansin B | β-methoxyacrylate | 0.4 µg/ml (K1)  1.12 µM | Fungus | *Favolaschia tonkinensis* BCC 18689 | 381 |
| 199 | 9α-hydroxyhalorosellinia A | Anthraquinone | 25.0 µM (K1) | Fungus | *Fusarium* spp. PSU-F14 | 382 |
| 200 | Bostrycin | Anthraquinone | 9.8 µM (K1) | Fungus | *Fusarium* spp. PSU-F14 | 382 |
| 201 | Nigrosporin B | Anthraquinone | 13.0 µM (K1) | Fungus | *Fusarium* spp. PSU-F14 | 382 |
| 202 | Javanicin | Naphthoquinone | 12.0 µM (K1) | Fungus | *Fusarium* sp.  PSU-F135 | 382 |
| 203 | Anhydrofusarubin | Naphthoquinone | 14.0 µM (K1) | Fungus | *Fusarium* sp.  PSU-F135 | 382 |
| 204 | Ganoderic acid TR | Lanostane triterpene | 20.0 µM | Mushroom | *Ganoderma lucidum* | 383 |
| 205 | Ganoderic Acid TR 1 (New) | Lanostane triterpene | 18.0 µM | Mushroom | *Ganoderma lucidum* | 383 |
| 206 | Ganoderic aldehyde TR (New) | Lanostane triterpene | 6.0 µM | Mushroom | *Ganoderma lucidum* | 383 |
| 207 | Ganoderic acid S | Lanostane triterpene | 11.0 µM | Mushroom | *Ganoderma lucidum* | 383 |
| 208 | Ganodermanondiol | Lanostane triterpene | 13.0 µM | Mushroom | *Ganoderma lucidum* | 383 |
| 209 | 23-Hydroxyganoderic Acid S (New) | Lanostane triterpene | 11.0 µM | Mushroom | *Ganoderma lucidum* | 383 |
| 210 | Psammaplysin G (New) | Bromotyrosine alkaloid | 98% inhibition at 40 µM (Dd2) | Sponge | *Hyattella*  sp. | 384 |
| 211 | Psammaplysin F | Bromotyrosine alkaloid | 1.4 µM (Dd2)  0.87 µM (3D7) | Sponge | *Hyattella*  sp. | 384 |
| 212 | Discorhabdin A | Pyrroloiminoquinone | 0.053 µM (D6)  0.053 µM (W2) | Sponge | *Latrunculia sp.* | 385 |
| 213 | Discorhabdin C | Pyrroloiminoquinone | 2.8 µM (D6)  2.0 µM (W2) | Sponge | *Latrunculia sp.* | 385 |
| 214 | Dihydrodiscorhabdin C | Pyrroloiminoquinone | 0.17 µM (D6)  0.13 µM (W2) | Sponge | *Latrunculia sp.* | 385 |
| 215 | Malyngolide dimer (New) | δ-Lactone | 19.0 µM (W2) | Cyanobacterium | *Lyngbya majuscula* | 386 |
| 216 | Lagunamide A (New) | Cyclodepsipeptide | 0.19 µM (NF54) | Cyanobacterium | *Lyngbya majuscula* | 387 |
| 217 | Lagunamide B (New) | Cyclodepsipeptide | 0.91 µM (NF54) | Cyanobacterium | *Lyngbya majuscula* | 387 |
| 218 | Paecilomycin A (New) | Resorcylic acid lactone | 0.78 µM (3D7) | Fungus | *Paecilomyces* sp. SC0924 | 388 |
| 219 | Paecilomycin B (New) | Resorcylic acid lactone | 3.8 µM(3D7) | Fungus | *Paecilomyces* sp. SC0924 | 388 |
| 220 | Paecilomycin E (New) | Resorcylic acid lactone | 20.0 nM(3D7)  8.8 µM(Dd2) | Fungus | *Paecilomyces* sp. SC0924 | 388 |
| 221 | Paecilomycin F (New) | Resorcylic acid lactone | 1.1 µM(3D7)  1.7 µM(Dd2) | Fungus | *Paecilomyces* sp. SC0924 | 388 |
| 222 | Aigilomycin B | Resorcylic acid lactone | 0.65 µM(3D7)  13.5 µM(Dd2) | Fungus | *Paecilomyces* sp. SC0924 | 388 |
| 223 | Aigilomycin G | Resorcylic acid lactone | 3.1 µM(3D7)  10.5 µM (Dd2) | Fungus | *Paecilomyces* sp. SC0924 | 388 |
| 224 | Aigilomycin F | Resorcylic acid lactone | 10.9 nM(3D7) | Fungus | *Paecilomyces* sp. SC0924 | 388 |
| 225 | Pandaroside E (New) | Steroidal glycoside | 13.8 µM (K1) | Marine sponge | *Pandaros acanthifolium* | 389 |
| 226 | Pandaroside F (New) | Steroidal glycoside | 5.7 µM (K1) | Marine sponge | *Pandaros acanthifolium* | 389 |
| 227 | Pandaroside G (New) | Steroidal glycoside | 2.5 µM (K1) | Marine sponge | *Pandaros acanthifolium* | 389 |
| 228 | Pandaroside H (New) | Steroidal glycoside | 22.8 µM (K1) | Marine sponge | *Pandaros acanthifolium* | 389 |
| 229 | Pandaroside I(New) | Steroidal glycoside | >25.0 µM (K1) | Marine sponge | *Pandaros acanthifolium* | 389 |
| 230 | Pandaroside J (New) | Steroidal glycoside | 24.3 µM (K1) | Marine sponge | *Pandaros acanthifolium* | 389 |
| 231 | Pandaroside E methyl ester (New) | Steroid | 5.9 µM (K1) | Marine sponge | *Pandaros acanthifolium* | 389 |
| 232 | Pandaroside F methyl ester (New) | Steroid | 9.9 µM (K1) | Marine sponge | *Pandaros acanthifolium* | 389 |
| 233 | Pandaroside G methyl ester (New) | Steroid | 0.39 µM (K1) | Marine sponge | *Pandaros acanthifolium* | 389 |
| 234 | Pandaroside H methyl ester(New) | Steroid | 10.2 µM (K1) | Marine sponge | *Pandaros acanthifolium* | 389 |
| 235 | Pandaroside I methyl ester (New) | Steroid | 13.0 µM (K1) | Marine sponge | *Pandaros acanthifolium* | 389 |
| 236 | Pandaroside J methyl ester (New) | Steroid | 12.4 µM (K1) | Marine sponge | *Pandaros acanthifolium* | 389 |
| 237 | Pandaroside A | Steroidal glycoside | 17.6 µM (K1) | Marine sponge | *Pandaros acanthifolium* | 389 |
| 238 | Pandaroside C | Steroidal glycoside | >25.0 µM (K1) | Marine sponge | *Pandaros acanthifolium* | 389 |
| 239 | Pandaroside D | Steroidal glycoside | 13.5 µM (K1) | Marine sponge | *Pandaros acanthifolium* | 389 |
| 240 | Pestalactam A (New) | Lactam | 41.3% inhibition at 25 µM (Dd2)  16.2% inhibition at 25 µM (3D7) | Endophytic fungus | *Pestalotiopsis* sp. | 390 |
| 241 | Pestalactam B (New) | Lactam | 36.3% inhibition at 25 µM (Dd2)  20.7% inhibition at 25 µM (3D7) | Endophytic fungus | *Pestalotiopsis* sp. | 390 |
| 242 | brefeldin A | Lactone | 1.12 µg/ml (K1)  3.99 µM | Fungus | *Penicillium sp.* BCC 17468 | 391 |
| 243 | Manadoperoxide A (New) | Cyclic peroxide | 6.88 µM (D10)  3.74 µM (W2) | Sponge | *Plakortis* cfr. *simplex* | 392 |
| 244 | Manadoperoxide B (New) | Cyclic peroxide | 6.76 µM (D10)  3.69 µM (W2) | Sponge | *Plakortis* cfr. *simplex* | 392 |
| 245 | Manadoperoxide C (New) | Cyclic peroxide | 4.54 µM (D10)  2.33 µM (W2) | Sponge | *Plakortis* cfr. *simplex* | 392 |
| 246 | Manadoperoxide D (New) | Cyclic peroxide | 10.38 µM (D10)  7.93 µM (W2) | Sponge | *Plakortis* cfr. *simplex* | 392 |
| 247 | Plakortin | Cyclic peroxide | 0.87 µM (D10)  0.39 µM (W2) | Sponge | *Plakortis* cfr. *simplex* | 392 |
| 248 | Epiplakinic acid F methyl ester (New) | Cyclic peroxide | 4.0 µM (W2) | Sponge | *Plakortis halichondrioides* | 393 |
| 249 | Plakortolide J (New) | Cyclic peroxide | >10.0 µM (W2) | Sponge | *Plakortis halichondrioides* | 393 |
| 250 | Epiplakinidioic acid (New) | Cyclic peroxide | 0.3 µM (W2) | Sponge | *Plakortis halichondrioides* | 393 |
| 251 | Epiplakinic acid F | Cyclic peroxide | 3.0 µM (W2) | Sponge | *Plakortis halichondrioides* | 393 |
| 252 | Pyridone alkaloid (New) | Pyridone alkaloid | 0.33 µg/ml (D6)  0.88 µM  0.20 µg/ml (W2)  0.53 µM | Fungus | *Septoria pistaciarum* | 394 |
| 253 | Torrubiellone A (New) | Pyridone alkaloid | 8.1 µM (K1) | Fungus | *Torrubiella sp.* BCC 2165 | 395 |
| 254 | Halenaquinone | Quinone polyketide | >30.0 µM (FcB1)  >30.0 µM (3D7) | Marine sponge | *Xestospongia* sp. | 396 |
| 255 | 3-Ketoadociaquinone A | Quinone polyketide | 1.08 µM (FcB1)  1.67 µM (3D7) | Marine sponge | *Xestospongia* sp. | 396 |
| 256 | 3-Ketoadociaquinone B | Quinone polyketide | 3.89 µM (FcB1)  4.12 µM (3D7) | Marine sponge | *Xestospongia* sp. | 396 |
| 257 | Tetrahydrohalenaquinone A | Quinone polyketide | >29.0 µM (FcB1)  >29.0 µM (3D7) | Marine sponge | *Xestospongia* sp. | 396 |
| 258 | Tetrahydrohalenaquinone B | Quinone polyketide | >29.0 µM (FcB1)  >29.0 µM (3D7) | Marine sponge | *Xestospongia* sp. | 396 |
| 259 | Halenaquinol sulfate | Quinone polyketide | >24.0 µM (FcB1)  >24.0 µM (3D7) | Marine sponge | *Xestospongia* sp. | 396 |
| 260 | Xestosaprol C methylacetal (New) | Quinone polyketide | >21.0 µM (FcB1)  >21.0 µM (3D7) | Marine sponge | *Xestospongia* sp. | 396 |
| 261 | Orhalquinone (New) | Quinone polyketide | 9.22 µM (FcB1)  10.94 µM (3D7) | Marine sponge | *Xestospongia* sp. | 396 |
| 262 | 7α,10α-Dihydroxy-1β-methoxyeremophil-11(13)-en-12,8β-olide (New) | Eremophilane sesquiterpenoids | 8.1 µM (K1) | Fungus | *Xylaria* sp. BCC 21097 | 397 |
| 263 | 1α,10α-Epoxy-7α-hydroxyeremophil-11(13)-en-12,8β-olide (New) | Eremophilane sesquiterpenoids | 13.0 µM (K1) | Fungus | *Xylaria* sp. BCC 21097 | 397 |

References

1. D. Abdissa, G. Geleta, K. Bacha and N. Abdissa, *PLoS One*, 2017, 12, e0173882.

2. N. Cho, Y. Du, A. L. Valenciano, M. L. Fernández-Murga, M. Goetz, J. Clement, M. B. Cassera and D. G. Kingston, *Bioorg. Med. Chem. Lett.*, 2018, 28, 40-42.

3. J. B. Althaus, C. Malyszek, M. Kaiser, R. Brun and T. J. Schmidt, *Molecules*, 2017, 22, 796.

4. D. T. Tshitenge, D. Feineis, V. Mudogo, M. Kaiser, R. Brun and G. Bringmann, *Sci. Rep.*, 2017, 7, 5767.

5. J. Li, R. Seupel, T. Bruhn, D. Feineis, M. Kaiser, R. Brun, V. Mudogo, S. Awale and G. Bringmann, *J. Nat. Prod.*, 2017, 80, 2807-2817.

6. J. Li, R. Seupel, D. Feineis, V. Mudogo, M. Kaiser, R. Brun, D. Brünnert, M. Chatterjee, E.-J. Seo and T. Efferth, *J. Nat. Prod.*, 2017, 80, 443-458.

7. G. Bringmann, R. Seupel, D. Feineis, M. Xu, G. Zhang, M. Kaiser, R. Brun, E.-J. Seo and T. Efferth, *Fitoterapia*, 2017, 121, 76-85.

8. S. P. Senadeera, S. Duffy, V. M. Avery and A. R. Carroll, *Bioorg. Med. Chem. Lett.*, 2017, 27, 2602-2607.

9. S. Cheenpracha, P. Boapun, T. Limtharakul, S. Laphookhieo and S. G. Pyne, *Nat. Prod. Res.*, 2017, 1-7.

10. M. D. Pereira, T. da Silva, A. C. C. Aguiar, G. Oliva, R. V. Guido, J. K. Yokoyama-Yasunaka, S. R. Uliana and L. M. Lopes, *Planta Med.*, 2017, 83, 912-920.

11. H. L. Greve, M. Kaiser, R. Brun and T. J. Schmidt, *Planta Med.*, 2017, 83, 1214-1226.

12. Y. Endo, T. Kasahara, K. Harada, M. Kubo, T. Etoh, M. Ishibashi, A. Ishiyama, M. Iwatsuki, K. Otoguro and S. O̅mura, *J. Nat. Prod.*, 2017, 80, 3120-3127.

13. P. Chumkaew, J. Pechwang and T. Srisawat, *J. Nat. Med.*, 2017, 71, 570-573.

14. R. S. O. Nondo, M. J. Moshi, P. Erasto, P. J. Masimba, F. Machumi, A. W. Kidukuli, M. Heydenreich and D. Zofou, *BMC Complementary Altern. Med.*, 2017, 17, 167.

15. N.-L. Zhu, Z.-H. Sun, M.-G. Hu, T.-Y. Wu, J.-Q. Yuan, H.-F. Wu, Y. Tian, P.-F. Li, J.-S. Yang and G.-X. Ma, *Molecules*, 2017, 22, 1751.

16. J. Yu, B. Zhou, S. Dalal, Q. Liu, M. B. Cassera and J. Yue, *Chin. J. Chem.*, 2018, 36, 124-128.

17. W. Sangsopha, K. Kanokmedhakul, R. Lekphrom and S. Kanokmedhakul, *Nat. Prod. Res.*, 2017, 1-4.

18. C. C. Presley, Y. Du, S. Dalal, E. F. Merino, J. H. Butler, S. Rakotonandrasana, V. E. Rasamison, M. B. Cassera and D. G. Kingston, *Bioorg. Med. Chem.*, 2017, 25, 4203-4211.

19. E. Tuenter, K. Segers, K. B. Kang, J. Viaene, S. H. Sung, P. Cos, L. Maes, Y. V. Heyden and L. Pieters, *Molecules*, 2017, 22, 224.

20. Y. Melaku, T. Worku, Y. Tadesse, Y. Mekonnen, J. Schmidt, N. Arnold and E. Dagne, *Curr. Bioact. Compd.*, 2017, 13, 268-273.

21. H. Bitew, W. Mammo, A. Hymete and M. Y. Yeshak, *Molecules*, 2017, 22, 1965.

22. K. Bezu, D. Bisrat and K. Asres, *Pharmacogn. J*, 2015, 7, 305-310.

23. G. M. Happi, F. M. Talontsi, H. Laatsch, S. Zühlke, B. T. Ngadjui, M. Spiteller and S. F. Kouam, *Fitoterapia*, 2018, 124, 17-22.

24. R. Kumar, S. Duffy, V. M. Avery and R. A. Davis, *Bioorg. Med. Chem. Lett.*, 2017, 27, 4091-4095.

25. S. V. Singh, A. Manhas, S. P. Singh, S. Mishra, N. Tiwari, P. Kumar, K. Shanker, K. Srivastava, K. V. Sashidhara and A. Pal, *Phytomedicine*, 2017, 30, 1-9.

26. L. P. Robertson, S. Duffy, Y. Wang, D. Wang, V. M. Avery and A. R. Carroll, *J. Nat. Prod.*, 2017.

27. S. V. Singh, A. Manhas, Y. Kumar, S. Mishra, K. Shanker, F. Khan, K. Srivastava and A. Pal, *Biomed. Pharmacother.*, 2017, 89, 761-771.

28. A. E. Fox Ramos, C. Alcover, L. Evanno, A. Maciuk, M. Litaudon, C. Duplais, G. Bernadat, J.-F. o. Gallard, J.-C. Jullian and E. Mouray, *J. Nat. Prod.*, 2017, 80, 1007-1014.

29. P. T. Tshibangu, P. M. Kapepula, M. K. Kapinga, A. T. Mukuta, D. T. Kalenda, A. T. Tchinda, A. A. Mouithys-Mickalad, O. Jansen, E. Cieckiewicz and M. Tits, *J. Ethnopharmacol.*, 2017, 196, 261-266.

30. A. Amin, E. Tuenter, K. Foubert, J. Iqbal, P. Cos, L. Maes, V. Exarchou, S. Apers and L. Pieters, *Front. Pharmacol.*, 2017, 8, 232.

31. C. C. Presley, A. L. Valenciano, M. L. Fernández-Murga, Y. Du, N. Shanaiah, M. B. Cassera, M. Goetz, J. A. Clement and D. G. Kingston, *J. Nat. Prod.*, 2017.

32. D. K. Singh, H. S. Cheema, A. Saxena, S. Singh, M. P. Darokar, D. U. Bawankule, K. Shanker and S. Luqman, *Phytomedicine*, 2017, 36, 262-272.

33. A. V. Gadetskaya, S. M. Mohamed, A. H. Tarawneh, N. M. Mohamed, G. Ma, B. N. Ponomarev, G. E. Zhusupova, C. L. Cantrell, S. J. Cutler and S. A. Ross, *Med. Chem. Res.*, 2017, 26, 2743-2750.

34. E. Mehrotra, J. Vishwakarma, A. C. Tripathi, P. K. Sonar and S. K. Saraf, *Nat. Prod. Res.*, 2017, 1-5.

35. A. Latif, Y. Du, S. R. Dalal, E. F. Merino, M. B. Cassera, M. Goetz and D. G. Kingston, *Chem. Biodiversity*, 2017.

36. Y. Du, A. K. Abedi, A. L. Valenciano, M. L. Fernández‐Murga, M. B. Cassera, V. E. Rasamison, W. L. Applequist, J. S. Miller and D. G. Kingston, *Chem. Biodiversity*, 2017, 14.

37. O. Jansen, A. T. Tchinda, J. Loua, V. Esters, E. Cieckiewicz, A. Ledoux, P. D. Toukam, L. Angenot, M. Tits and A. M. Balde, *J. Ethnopharmacol.*, 2017, 203, 20-26.

38. T. Zininga, L. Ramatsui, P. B. Makhado, S. Makumire, I. Achilinou, H. Hoppe, H. Dirr and A. Shonhai, *Molecules*, 2017, 22, 2139.

39. G. Komlaga, G. Genta-Jouve, S. Cojean, R. A. Dickson, M. L. Mensah, P. M. Loiseau, P. Champy and M. A. Beniddir, *Tetrahedron Lett.*, 2017, 58, 3754-3756.

40. K. M. d. Araújo-Vilges, S. V. d. Oliveira, S. C. P. Couto, H. H. Fokoue, G. A. S. Romero, M. J. Kato, L. A. S. Romeiro, J. R. S. A. Leite and S. A. S. Kuckelhaus, *Pharm. Biol.*, 2017, 55, 1601-1607.

41. E. O. N’Nang Obiang, G. g. Genta-Jouve, J.-F. o. Gallard, B. Kumulungui, E. Mouray, P. Grellier, L. Evanno, E. Poupon, P. Champy and M. A. Beniddir, *Org. Lett.*, 2017, 19, 6180-6183.

42. A. Ledoux, A. St-Gelais, E. Cieckiewicz, O. Jansen, A. Bordignon, B. Illien, N. Di Giovanni, A. Marvilliers, F. Hoareau and H. Pendeville, *J. Nat. Prod.*, 2017, 80, 1750-1757.

43. L. S. Sidjui, Y. O. Nganso, R. M. Toghueo, B. N. Wakeu, J. T. Dameue, P. Mkounga, A. Adhikari, M. Lateef, G. N. Folefoc and M. S. Ali, *Z. Naturforsch. C Bio. Sci.*, 2018, 73, 153-160.

44. R. Rajendran, B. S. Narashimman, V. Trivedi and R. Chaturvedi, *J. Biosci. Bioeng.*, 2017, 124, 99-107.

45. P. M. Le, V. Srivastava, T. T. Nguyen, B. Pradines, M. Madamet, J. Mosnier, T. T. Trinh and H. Lee, *Phytother. Res.*, 2017, 31, 1357-1368.

46. Y. Atilaw, S. Duffy, M. Heydenreich, L. Muiva-Mutisya, V. M. Avery, M. Erdélyi and A. Yenesew, *Molecules*, 2017, 22, 318.

47. Y. Atilaw, L. Muiva-Mutisya, A. Ndakala, H. M. Akala, R. Yeda, Y. J. Wu, P. Coghi, V. K. Wong, M. Erdélyi and A. Yenesew, *Molecules*, 2017, 22, 1514.

48. L. M. Muiva-Mutisya, Y. Atilaw, M. Heydenreich, A. Koch, H. M. Akala, A. C. Cheruiyot, M. L. Brown, B. Irungu, F. A. Okalebo and S. Derese, *Nat. Prod. Res.*, 2017, 1-8.

49. Y. Du, K. C. Pearce, Y. Dai, P. Krai, S. Dalal, M. B. Cassera, M. Goetz, T. D. Crawford and D. G. Kingston, *J. Nat. Prod.*, 2017, 80, 1639-1647.

50. Y. Chen, J. Zhao, Y. Qiu, H. Yuan, S. I. Khan, N. Hussain, M. I. Choudhary, F. Zeng, D.-A. Guo and I. A. Khan, *Fitoterapia*, 2017, 119, 64-68.

51. C. Auranwiwat, P. Wongsomboon, T. Thaima, R. Rattanajak, S. Kamchonwongpaisan, A. C. Willis, W. Lie, S. G. Pyne and T. Limtharakul, *Fitoterapia*, 2017, 120, 103-107.

52. A.-W. Salae, O. Chairerk, P. Sukkoet, T. Chairat, U. Prawat, P. Tuntiwachwuttikul, P. Chalermglin and S. Ruchirawat, *Phytochemistry*, 2017, 135, 135-143.

53. A. Bordignon, M. Frédérich, A. Ledoux, P.-E. Campos, P. Clerc, T. Hermann, J. Quetin-Leclercq and E. Cieckiewicz, *Nat. Prod. Res.*, 2017, 1-4.

54. L. K. Omosa and E. K. Okemwa, *Pharmacogn. Commun.*, 2017, 7, 41.

55. E. K. Aratikatla, T. R. Valkute, S. K. Puri, K. Srivastava and A. K. Bhattacharya, *Eur. J. Med. Chem.*, 2017, 138, 1089-1105.

56. M. Isaka, S. Palasarn, S. Sommai, S. Veeranondha, K. Srichomthong, P. Kongsaeree and S. Prabpai, *Tetrahedron*, 2017, 73, 1561-1567.

57. V. Smyrniotopoulos, C. Merten, M. Kaiser and D. Tasdemir, *Mar. Drugs*, 2017, 15, 245.

58. X.-Q. Zhang, C. Spadafora, L. M. Pineda, M. G. Ng, J.-H. Sun, W. Wang, C.-Y. Wang, Y.-C. Gu and C.-L. Shao, *Sci. Rep.*, 2017, 7, 11822.

59. J. Otaka, D. Hashizume, Y. Masumoto, A. Muranaka, M. Uchiyama, H. Koshino, Y. Futamura and H. Osada, *Org. Lett.*, 2017, 19, 4030-4033.

60. M. C. Ferreira, C. L. Cantrell, D. E. Wedge, V. N. Gonçalves, M. R. Jacob, S. Khan, C. A. Rosa and L. H. Rosa, *Mem. Inst. Oswaldo Cruz*, 2017, 112, 692-697.

61. S. R. Ibrahim, H. M. Abdallah, E. S. Elkhayat, N. M. Al Musayeib, H. Z. Asfour, M. F. Zayed and G. A. Mohamed, *J. Asian Nat. Prod. Res.*, 2018, 20, 75-85.

62. N. Bunbamrung, C. Intaraudom, A. Dramae, N. Boonyuen, S. Veeranondha, P. Rachtawee and P. Pittayakhajonwut, *Phytochem. Lett.*, 2017, 20, 274-281.

63. G. Chianese, J. Silber, P. Luciano, C. Merten, D. Erpenbeck, B. l. Topaloglu, M. Kaiser and D. Tasdemir, *J. Nat. Prod.*, 2017, 80, 2566-2571.

64. P.-E. Campos, J.-L. Wolfender, E. F. Queiroz, L. Marcourt, A. Al-Mourabit, M. Frederich, A. Bordignon, N. De Voogd, B. Illien and A. Gauvin-Bialecki, *J. Nat. Prod.*, 2017, 80, 1404-1410.

65. J. Kornsakulkarn, W. Choowong, P. Rachtawee, N. Boonyuen, S. Kongthong, M. Isaka and C. Thongpanchang, *Phytochem. Lett.*, 2018, 24, 46-50.

66. C.-L. Shao, X.-F. Mou, F. Cao, C. Spadafora, E. Glukhov, L. Gerwick, C.-Y. Wang and W. H. Gerwick, *J. Nat. Prod.*, 2018.

67. A. M. White, K. Dao, D. Vrubliauskas, Z. A. Könst, G. K. Pierens, A. Mándi, K. T. Andrews, T. S. Skinner-Adams, M. E. Clarke and P. T. Narbutas, *J. Org. Chem.*, 2017, 82, 13313-13323.

68. T. Nogawa, N. Kato, T. Shimizu, A. Okano, Y. Futamura, S. Takahashi and H. Osada, *J. Antibiot.*, 2018, 71, 123.

69. K. Supong, P. Sripreechasak, S. Tanasupawat, K. Danwisetkanjana, P. Rachtawee and P. Pittayakhajonwut, *Appl. Microbiol. Biotechnol.*, 2017, 101, 533-543.

70. J.-P. Jang, T. Nogawa, Y. Futamura, T. Shimizu, D. Hashizume, S. Takahashi, J.-H. Jang, J. S. Ahn and H. Osada, *J. Nat. Prod.*, 2017, 80, 134-140.

71. N. M. Zin, M. S. Baba, A. H. Zainal-Abidin, J. Latip, N. W. Mazlan and R. Edrada-Ebel, *Drug Des., Dev. Ther.*, 2017, 11, 351.

72. G. M. LaMonte, J. Almaliti, B. Bibo-Verdugo, L. Keller, B. Y. Zou, J. Yang, Y. Antonova-Koch, P. Orjuela-Sanchez, C. A. Boyle and E. Vigil, *J. Med. Chem.*, 2017, 60, 6721-6732.

73. M. Issac, M. Aknin, A. Gauvin-Bialecki, N. De Voogd, A. Ledoux, M. Frederich, Y. Kashman and S. Carmeli, *J. Nat. Prod.*, 2017, 80, 1110-1116.

74. B. K. Lombe, T. Bruhn, D. Feineis, V. Mudogo, R. Brun and G. Bringmann, *Org. Lett.*, 2017, 19, 6740-6743.

75. S. Meesala, P. Gurung, K. Karmodiya, P. Subrayan and M. G. Watve, *J. Asian Nat. Prod. Res.*, 2017, 1-8.

76. A. E. Wright, K. B. Killday, D. Chakrabarti, E. A. Guzmán, D. Harmody, P. J. McCarthy, T. Pitts, S. A. Pomponi, J. K. Reed and B. F. Roberts, *Mar. Drugs*, 2017, 15, 16.

77. I. Atay, H. Kirmizibekmez, M. Kaiser, G. Akaydin, E. Yesilada and D. Tasdemir, *Pharm. Biol.*, 2016, 54, 1808-1814.

78. M. S. Nogueira, F. B. Da Costa, R. Brun, M. Kaiser and T. J. Schmidt, *Molecules*, 2016, 21, 1237.

79. T. Teka, D. Bisrat, M. Y. Yeshak and K. Asres, *Molecules*, 2016, 21, 1415.

80. H. Zhang, J. Liu, L.-S. Gan, S. Dalal, M. B. Cassera and J.-M. Yue, *Org. Biomol. Chem.*, 2016, 14, 957-962.

81. P. Chumkaew and T. Srisawat, *J. Asian Nat. Prod. Res.*, 2017, 19, 247-253.

82. J. C. Chukwujekwu, K. R. Rengasamy, C. A. de Kock, P. J. Smith, L. P. Slavětínská and J. van Staden, *J. Enzyme Inhib. Med. Chem.*, 2016, 31, 63-66.

83. S. Cai, A. L. Risinger, S. Nair, J. Peng, T. J. Anderson, L. Du, D. R. Powell, S. L. Mooberry and R. H. Cichewicz, *J. Nat. Prod.*, 2015, 79, 490-498.

84. B. Zhou, Y. Wu, S. Dalal, E. F. Merino, Q.-F. Liu, C.-H. Xu, T. Yuan, J. Ding, D. G. Kingston and M. B. Cassera, *J. Nat. Prod.*, 2016, 80, 96-107.

85. S. S. Nyandoro, J. J. Munissi, A. Gruhonjic, S. Duffy, F. Pan, R. Puttreddy, J. P. Holleran, P. A. Fitzpatrick, J. Pelletier and V. M. Avery, *J. Nat. Prod.*, 2016, 80, 114-125.

86. M. M. Adia, S. N. Emami, R. Byamukama, I. Faye and A.-K. Borg-Karlson, *J. Ethnopharmacol.*, 2016, 186, 14-19.

87. C. C. Presley, P. Krai, S. Dalal, Q. Su, M. Cassera, M. Goetz and D. G. Kingston, *Bioorg. Med. Chem.*, 2016, 24, 5418-5422.

88. J. Chukwujekwu, C. de Kock, P. Smith, F. Van Heerden and J. Van Staden, *S. Afr. J. Bot.*, 2016, 106, 101-103.

89. B. Zhou, Y. Wu, S. Dalal, M. B. Cassera and J.-M. Yue, *J. Nat. Prod.*, 2016, 79, 1952-1961.

90. M. Kubo, W. Yatsuzuka, S. Matsushima, K. Harada, Y. Inoue, H. Miyamoto, M. Matsumoto and Y. Fukuyama, *Chem. Pharm. Bull.*, 2016, 64, 957-960.

91. H. Ke, J. M. Morrisey, S. Qu, O. Chantarasriwong, M. W. Mather, E. A. Theodorakis and A. B. Vaidya, *Antimicrob. Agents Chemother.*, 2017, 61, e01220-01216.

92. H. L. Mai, P. Grellier, E. Prost, P. Lemoine, C. Poullain, V. Dumontet, B. Deguin, T. B. H. Vo, S. Michel and R. Grougnet, *Phytochemistry*, 2016, 122, 193-202.

93. D. M. Muganza, B. Fruth, J. L. Nzunzu, E. Tuenter, K. Foubert, P. Cos, L. Maes, R. C. Kanyanga, V. Exarchou and S. Apers, *J. Ethnopharmacol.*, 2016, 193, 510-516.

94. Q. Su, S. Dalal, M. Goetz, M. B. Cassera and D. G. Kingston, *Nat. Prod. Commun.*, 2016, 11, 719.

95. A. E. Mostafa, A. Atef, A.-E. I. Mohammad, S. J. Cutler and S. A. Ross, *Phytochem. Lett.*, 2016, 17, 213-218.

96. P. Lomchid, P. Nasomjai, S. Kanokmedhakul, J. Boonmak, S. Youngme and K. Kanokmedhakul, *Planta Med.*, 2017, 83, 334-340.

97. K. Foubert, T. Gorella, A. Faizal, P. Cos, L. Maes, S. Apers, D. Geelen and L. Pieters, *Planta Med.*, 2016, 82, 1568-1575.

98. A. L. Eaton, S. Dalal, M. B. Cassera, S. Zhao and D. G. Kingston, *J. Nat. Prod.*, 2016, 79, 1679-1683.

99. H. Khan, H. Amin, A. Ullah, S. Saba, J. Rafique, K. Khan, N. Ahmad and S. L. Badshah, *Oxid. Med. Cell. Longevity*, 2016, 2016.

100. V. E. Rasamison, P. J. Brodie, E. F. Merino, M. B. Cassera, M. A. Ratsimbason, S. Rakotonandrasana, A. Rakotondrafara, E. Rafidinarivo, D. G. Kingston and H. L. Rakotondraibe, *Nat. Prod. Bioprospect.*, 2016, 6, 261-265.

101. T. Promchai, A. Jaidee, S. Cheenpracha, K. Trisuwan, R. Rattanajak, S. Kamchonwongpaisan, S. Laphookhieo, S. G. Pyne and T. Ritthiwigrom, *J. Nat. Prod.*, 2016, 79, 978-983.

102. P. Vásquez-Ocmín, M. Haddad, A. Gadea, V. Jullian, D. Castillo, L. Paloque, J. P. Cerapio, G. Bourdy and M. Sauvain, *Nat. Prod. Res.*, 2017, 31, 138-142.

103. H. Xiao, R. Rao Ravu, B. L. Tekwani, W. Li, W.-B. Liu, M. R. Jacob, S. I. Khan, X. Cai, C.-Y. Peng and I. A. Khan, *Nat. Prod. Res.*, 2017, 31, 2053-2057.

104. E. Houël, F. Nardella, V. Jullian, A. Valentin, C. Vonthron-Sénécheau, P. Villa, A. Obrecht, M. Kaiser, E. Bourreau and G. Odonne, *J. Ethnopharmacol.*, 2016, 187, 241-248.

105. P. Mollinedo, J. L. Vila, H. Arando, M. Sauvain, E. Deharo and J. A. Bravo, *Nat. Prod. Res.*, 2016, 30, 2594-2597.

106. Q. Su, S. Dalal, M. Goetz, M. B. Cassera and D. G. Kingston, *Bioorg. Med. Chem.*, 2016, 24, 2544-2548.

107. C. D. Goodman, I. Austarheim, V. Mollard, B. Mikolo, K. E. Malterud, G. I. McFadden and H. Wangensteen, *Malar. J.*, 2016, 15, 481.

108. E. Tuenter, R. Ahmad, K. Foubert, A. Amin, M. Orfanoudaki, P. Cos, L. Maes, S. Apers, L. Pieters and V. Exarchou, *J. Nat. Prod.*, 2016, 79, 2865-2872.

109. E. Tuenter, V. Exarchou, A. Baldé, P. Cos, L. Maes, S. Apers and L. Pieters, *J. Nat. Prod.*, 2016, 79, 1746-1751.

110. F. Yang, R.-P. Wang, B. Xu, H.-B. Yu, G.-Y. Ma, G.-F. Wang, S.-W. Dai, W. Zhang, W.-H. Jiao and S.-J. Song, *Bioorg. Med. Chem. Lett.*, 2016, 26, 2084-2087.

111. O.-a. Rajachan, K. Kanokmedhakul, W. Sanmanoch, S. Boonlue, S. Hannongbua, P. Saparpakorn and S. Kanokmedhakul, *Phytochemistry*, 2016, 132, 68-75.

112. S. Son, S.-K. Ko, J. W. Kim, J. K. Lee, M. Jang, I.-J. Ryoo, G. J. Hwang, M. C. Kwon, K.-S. Shin and Y. Futamura, *Phytochemistry*, 2016, 122, 154-164.

113. K. Supong, C. Thawai, W. Choowong, C. Kittiwongwattana, D. Thanaboripat, C. Laosinwattana, P. Koohakan, N. Parinthawong and P. Pittayakhajonwut, *Res. Microbiol.*, 2016, 167, 290-298.

114. A. G. B. Azebaze, J. E. M. Teinkela, E. L. Nguemfo, A. Valentin, A. B. Dongmo and J. C. Vardamides, *Afr. Health Sci.*, 2015, 15, 835-840.

115. L. R. T. Yamthe, P. V. T. Fokou, C. D. J. Mbouna, R. Keumoe, B. L. Ndjakou, P. T. Djouonzo, A. N. Mfopa, J. Legac, N. Tsabang and J. Gut, *Medicines*, 2015, 2, 55-66.

116. P. D. Douanla, T. K. Tabopda, A. T. Tchinda, E. Cieckiewicz, M. Frédérich, F. F. Boyom, N. Tsabang, S. Yeboah, A. E. Nkengfack and M. H. K. Tchuendem, *Phytochemistry*, 2015, 117, 521-526.

117. Q. Su, P. Krai, M. Goetz, M. B. Cassera and D. G. Kingston, *Planta Med.*, 2015, 81, 1128.

118. M. F. Dolabela, M. M. Póvoa, G. C. Brandão, F. D. Rocha, L. F. Soares, R. C. Paula and A. B. Oliveira, *Malar. J.*, 2015, 14, 498.

119. L. Pan, U. M. Acuña, H. Chai, H.-Y. Park, T. N. Ninh, B. Van Thanh, E. F. Merino, M. B. Cassera, L. H. Rakotondraibe and E. J. C. De Blanco, *Planta Med.*, 2015, 81, 1133.

120. O. O. Ogunlana and O. Ogunlana, *J. Chem. Pharm. Res.*, 2015, 7, 931-937.

121. G. Ma, H. Wu, D. Chen, N. Zhu, Y. Zhu, Z. Sun, P. Li, J. Yang, J. Yuan and X. Xu, *J. Nat. Prod.*, 2015, 78, 2364-2371.

122. S. A. Ahmed, S. A. Ross, D. Slade, M. M. Radwan, I. A. Khan and M. A. ElSohly, *Phytochemistry*, 2015, 117, 194-199.

123. Y. Liu, K. Young, L. H. Rakotondraibe, P. J. Brodie, J. D. Wiley, M. B. Cassera, M. W. Callmander, R. Rakotondrajaona, E. Rakotobe and V. E. Rasamison, *J. Nat. Prod.*, 2015, 78, 1543-1547.

124. P. K. Boniface, S. Verma, A. Shukla, H. S. Cheema, S. K. Srivastava, F. Khan, M. P. Darokar and A. Pal, *Parasitol. Int.*, 2015, 64, 118-123.

125. A. T. Tchinda, S. R. Mouokeu, R. A. Ngono, M. R. Ebelle, A. L. Mokale, D. K. Nono and M. Frédérich, *Nat. Prod. Res.*, 2015, 29, 1990-1994.

126. Y. Liu, L. H. Rakotondraibe, P. J. Brodie, J. D. Wiley, M. B. Cassera, J. S. Miller, F. Ratovoson, E. Rakotobe, V. E. Rasamison and D. G. Kingston, *J. Nat. Prod.*, 2015, 78, 1330-1338.

127. F. Naghibi, S. Ghafari, S. Esmaeili and K. Jenett-Siems, *Iran. J. Pharm. Res.*, 2015, 14, 961.

128. G. M. Happi, S. F. Kouam, F. M. Talontsi, M. Lamshöft, S. Zühlke, J. O. Bauer, C. Strohmann and M. Spiteller, *J. Nat. Prod.*, 2015, 78, 604-614.

129. G. M. Happi, S. F. Kouam, F. M. Talontsi, S. Zühlke, M. Lamshöft and M. Spiteller, *Fitoterapia*, 2015, 102, 35-40.

130. B. Konziase, *J. Ethnopharmacol.*, 2015, 172, 214-218.

131. L. H. Mai, G. G. Chabot, P. Grellier, L. Quentin, V. Dumontet, C. Poulain, L. S. Espindola, S. Michel, H. T. Vo and B. Deguin, *Eur. J. Med. Chem.*, 2015, 93, 93-100.

132. N. Suchaichit, K. Kanokmedhakul, N. Panthama, K. Poopasit, P. Moosophon and S. Kanokmedhakul, *Fitoterapia*, 2015, 103, 206-212.

133. A. V. Gadetskaya, A. H. Tarawneh, G. E. Zhusupova, N. G. Gemejiyeva, C. L. Cantrell, S. J. Cutler and S. A. Ross, *Fitoterapia*, 2015, 104, 80-85.

134. T. Deyou, I. Gumula, F. Pang, A. Gruhonjic, M. Mumo, J. Holleran, S. Duffy, P. A. Fitzpatrick, M. Heydenreich and G. r. Landberg, *J. Nat. Prod.*, 2015, 78, 2932-2939.

135. L. F. R. e Silva, C. Ramalhete, K. L. Nogueira, S. Mulhovo, M.-J. U. Ferreira and A. M. Pohlit, *Eur. J. Med. Chem.*, 2015, 102, 398-402.

136. J. Namukobe, B. T. Kiremire, R. Byamukama, J. M. Kasenene, H. M. Akala, E. Kamau and V. Dumontet, *J. Ethnopharmacol.*, 2015, 162, 317-322.

137. L. H. Rakotondraibe, P. R. Graupner, Q. Xiong, M. Olson, J. D. Wiley, P. Krai, P. J. Brodie, M. W. Callmander, E. Rakotobe and F. Ratovoson, *J. Nat. Prod.*, 2015, 78, 431-440.

138. K. Annan, E. Ekuadzi, C. Asare, K. Sarpong, D. Pistorius, L. Oberer, B. A. Gyan and M. Ofori, *Phytochem. Lett.*, 2015, 11, 28-31.

139. S. Y. Gbedema, M. T. Bayor, K. Annan and C. W. Wright, *J. Ethnopharmacol.*, 2015, 169, 176-182.

140. C. Girardi, N. Fabre, L. Paloque, A. P. Ramadani, F. Benoit-Vical, G. González-Aspajo, M. Haddad, E. Rengifo and V. Jullian, *J. Ethnopharmacol.*, 2015, 170, 167-174.

141. R. A. Ramli, P. Pudjiastuti, T. S. Tjahjandaric, W. Lie, R. Rattanajak, S. Kamchonwongpaisan and S. G. Pyne, *Phytochem. Lett.*, 2015, 11, 157-162.

142. B. N. Irungu, N. Adipo, J. A. Orwa, F. Kimani, M. Heydenreich, J. O. Midiwo, P. M. Björemark, M. Håkansson, A. Yenesew and M. Erdélyi, *J. Ethnopharmacol.*, 2015, 174, 419-425.

143. J. Banzouzi, P. N. Soh, S. Ramos, P. Toto, A. Cavé, J. Hemez and F. Benoit-Vical, *J. Ethnopharmacol.*, 2015, 173, 100-104.

144. S. M. Mohamed, K. M. Elokely, E. Y. Bachkeet, S. A. Bayoumi, V. Carnevale, M. L. Klein, S. J. Cutler and S. A. Ross, *Nat. Prod. Commun.*, 2015, 10, 1897.

145. S. Sawadsitang, W. Mongkolthanaruk, N. Suwannasai and S. Sodngam, *Nat. Prod. Res.*, 2015, 29, 2033-2036.

146. C. Daengrot, V. Rukachaisirikul, C. Tansakul, T. Thongpanchang, S. Phongpaichit, K. Bowornwiriyapan and J. Sakayaroj, *J. Nat. Prod.*, 2015, 78, 615-622.

147. V. Choomuenwai, K. D. Beattie, P. C. Healy, K. T. Andrews, N. Fechner and R. A. Davis, *Phytochemistry*, 2015, 117, 10-16.

148. C.-L. Shao, R. G. Linington, M. J. Balunas, A. Centeno, P. Boudreau, C. Zhang, N. Engene, C. Spadafora, T. S. Mutka and D. E. Kyle, *J. Org. Chem.*, 2015, 80, 7849-7855.

149. A. M. White, G. K. Pierens, T. Skinner-Adams, K. T. Andrews, P. V. Bernhardt, E. H. Krenske, E. Mollo and M. J. Garson, *J. Nat. Prod.*, 2015, 78, 1422-1427.

150. C. J. Schulze, G. Navarro, D. Ebert, J. DeRisi and R. G. Linington, *J. Org. Chem.*, 2015, 80, 1312-1320.

151. E. Avilés, J. Prudhomme, K. G. Le Roch and A. D. Rodríguez, *Tetrahedron*, 2015, 71, 487-494.

152. E. Gros, M. T. Martin, J. Sorres, C. Moriou, J. Vacelet, M. Frederich, M. Aknin, Y. Kashman, A. Gauvin‐Bialecki and A. Al‐Mourabit, *Chem. Biodiversity*, 2015, 12, 1725-1733.

153. L. H. Rakotondraibe, R. Rasolomampianina, H.-Y. Park, J. Li, C. Slebodnik, P. J. Brodie, L. C. Blasiak, R. Hill, K. TenDyke and Y. Shen, *Bioorg. Med. Chem. Lett.*, 2015, 25, 5646-5649.

154. B. Chaiyosang, K. Kanokmedhakul, J. Boonmak, S. Youngme, V. Kukongviriyapan, K. Soytong and S. Kanokmedhakul, *Nat. Prod. Res.*, 2016, 30, 1017-1024.

155. K. C.-C. Cheng, S. Cao, A. Raveh, R. MacArthur, P. Dranchak, G. Chlipala, M. T. Okoneski, R. Guha, R. T. Eastman and J. Yuan, *J. Nat. Prod.*, 2015, 78, 2411-2422.

156. Y. Upegui, S. M. Robledo, J. F. Gil Romero, W. Quiñones, R. Archbold, F. Torres, G. Escobar, B. Nariño and F. Echeverri, *Phytother. Res.*, 2015, 29, 1195-1201.

157. J. B. Althaus, M. Kaiser, R. Brun and T. J. Schmidt, *Molecules*, 2014, 19, 6428-6438.

158. H. C. Upadhyay, B. S. Sisodia, J. Agrawal, A. Pal, M. P. Darokar and S. K. Srivastava, *Med. Chem. Res.*, 2014, 23, 870-876.

159. J. Liu, X.-F. He, G.-H. Wang, E. F. Merino, S.-P. Yang, R.-X. Zhu, L.-S. Gan, H. Zhang, M. B. Cassera and H.-Y. Wang, *J. Org. Chem.*, 2013, 79, 599-607.

160. T. P. Chierrito, A. C. Aguiar, I. M. de Andrade, I. P. Ceravolo, R. A. Gonçalves, A. J. de Oliveira and A. U. Krettli, *Malar. J.*, 2014, 13, 142.

161. A. Montoia, L. F. R. e Silva, Z. E. Torres, D. S. Costa, M. C. Henrique, E. S. Lima, M. C. Vasconcellos, R. C. Souza, M. R. Costa and A. Grafov, *Bioorg. Med. Chem. Lett.*, 2014, 24, 2631-2634.

162. G. Uddin, A. Sadat and B. S. Siddiqui, *Trop Biomed*, 2014, 31, 143-148.

163. J. B. Althaus, G. Jerz, P. Winterhalter, M. Kaiser, R. Brun and T. J. Schmidt, *Molecules*, 2014, 19, 6184-6201.

164. G. Ma, Z. Sun, Z. Sun, J. Yuan, H. Wei, J. Yang, H. Wu and X. Xu, *Fitoterapia*, 2014, 95, 234-239.

165. T. B. Pereira, L. F. R. e Silva, R. C. Amorim, M. R. Melo, R. C. Z. de Souza, M. N. Eberlin, E. S. Lima, M. C. Vasconcellos and A. M. Pohlit, *Malar. J.*, 2014, 13, 317.

166. T. Julianti, M. De Mieri, S. Zimmermann, S. N. Ebrahimi, M. Kaiser, M. Neuburger, M. Raith, R. Brun and M. Hamburger, *J. Ethnopharmacol.*, 2014, 155, 426-434.

167. I. Ezenyi, O. Salawu, R. Kulkarni and M. Emeje, *Parasitol. Res.*, 2014, 113, 4415-4422.

168. K. H. Leong, *Planta Med.*, 2014, 80, 599-603.

169. Y. Hata, M. De Mieri, S. N. Ebrahimi, T. Mokoka, G. Fouche, M. Kaiser, R. Brun, O. Potterat and M. Hamburger, *Phytochem. Lett.*, 2014, 10, cxxxiii-cxl.

170. T. S. Tjahjandarie, P. Pudjiastuti, R. D. Saputri and M. Tanjung, *J. Chem. Pharm. Res*, 2014, 6, 786-790.

171. J. T. Lyles, A. Negrin, S. I. Khan, K. He and E. J. Kennelly, *Planta Med.*, 2014, 80, 676-681.

172. Z. Lu, R. M. Van Wagoner, C. D. Pond, A. R. Pole, J. B. Jensen, D. A. Blankenship, B. T. Grimberg, R. Kiapranis, T. K. Matainaho and L. R. Barrows, *Org. Lett.*, 2013, 16, 346-349.

173. Y. Dai, L. Harinantenaina, J. D. Bowman, I. O. Da Fonseca, P. J. Brodie, M. Goetz, M. B. Cassera and D. G. Kingston, *Bioorg. Med. Chem.*, 2014, 22, 269-276.

174. M. C. d. M. Burger, J. B. Fernandes, M. F. d. G. a. F. da Silva, A. Escalante, J. Prudhomme, K. G. Le Roch, M. A. Izidoro and P. C. Vieira, *J. Nat. Prod.*, 2014, 77, 2418-2422.

175. C. Kamaraj, A. A. Rahuman, S. M. Roopan, A. Bagavan, G. Elango, A. A. Zahir, G. Rajakumar, C. Jayaseelan, T. Santhoshkumar and S. Marimuthu, *Parasitol. Res.*, 2014, 113, 1657-1672.

176. J. Namukobe, B. T. Kiremire, R. Byamukama, J. M. Kasenene, V. Dumontet, F. Guéritte, S. Krief, I. Florent and J. D. Kabasa, *Phytochemistry*, 2014, 102, 189-196.

177. Y. Liu, L. H. Rakotondraibe, P. J. Brodie, J. D. Wiley, M. B. Cassera, M. Goetz and D. G. Kingston, *Nat. Prod. Commun.*, 2014, 9, 1403.

178. R. A. Mothana, M. S. Al-Said, N. M. Al-Musayeib, A. A. E. Gamal, S. M. Al-Massarani, A. J. Al-Rehaily, M. Abdulkader and L. Maes, *Int. J. Mol. Sci.*, 2014, 15, 8360-8371.

179. W. Sumsakul, T. Plengsuriyakarn, W. Chaijaroenkul, V. Viyanant, J. Karbwang and K. Na-Bangchang, *BMC Complementary Altern. Med.*, 2014, 14, 15.

180. M. A. González, J. Clark, M. Connelly and F. Rivas, *Bioorg. Med. Chem. Lett.*, 2014, 24, 5234-5237.

181. S. F. Kouam, A. W. Ngouonpe, M. Lamshöft, F. M. Talontsi, J. O. Bauer, C. Strohmann, B. T. Ngadjui, H. Laatsch and M. Spiteller, *Phytochemistry*, 2014, 105, 52-59.

182. M. Pivatto, L. R. Baccini, A. Sharma, M. Nakabashi, A. Danuello, C. Viegas Júnior, C. R. Garcia and V. S. Bolzani, *J. Braz. Chem. Soc.*, 2014, 25, 1900-1906.

183. A. T. Tchinda, O. Jansen, J.-N. Nyemb, M. Tits, G. Dive, L. Angenot and M. Frédérich, *J. Nat. Prod.*, 2014, 77, 1078-1082.

184. N. M. Al-Musayeib, G. A. Mohamed, S. R. Ibrahim and S. A. Ross, *Molecules*, 2014, 19, 2819-2828.

185. R. Muganga, L. Angenot, M. Tits and M. Frédérich, *Planta Med.*, 2014, 80, 482-489.

186. R. Phatchana and C. Yenjai, *Planta Med.*, 2014, 80, 719-722.

187. V. E. Rasamison, L. H. Rakotondraibe, C. Slebodnick, P. J. Brodie, M. Ratsimbason, K. TenDyke, Y. Shen, L. M. Randrianjanaka and D. G. Kingston, *Org. Lett.*, 2014, 16, 2626-2629.

188. C. Wang, J. Wan, Z. Mei and X. Yang, *Pharmacogn. Mag.*, 2014, 10, 73.

189. E. Gros, A. Al-Mourabit, M.-T. r. s. Martin, J. Sorres, J. Vacelet, M. Frederich, M. Aknin, Y. Kashman and A. Gauvin-Bialecki, *J. Nat. Prod.*, 2014, 77, 818-823.

190. F. Yang, Y. Zou, R.-P. Wang, M. T. Hamann, H.-J. Zhang, W.-H. Jiao, B.-N. Han, S.-J. Song and H.-W. Lin, *Mar. Drugs*, 2014, 12, 4399-4416.

191. K. Ma, J. Ren, J. Han, L. Bao, L. Li, Y. Yao, C. Sun, B. Zhou and H. Liu, *J. Nat. Prod.*, 2014, 77, 1847-1852.

192. W. Lakornwong, K. Kanokmedhakul, S. Kanokmedhakul, P. Kongsaeree, S. Prabpai, P. Sibounnavong and K. Soytong, *J. Nat. Prod.*, 2014, 77, 1545-1553.

193. S. Kitchawalit, K. Kanokmedhakul, S. Kanokmedhakul and K. Soytong, *Nat. Prod. Res.*, 2014, 28, 1045-1051.

194. J. s. Martín, G. Crespo, V. González-Menéndez, G. Pérez-Moreno, P. Sánchez-Carrasco, I. Pérez-Victoria, L. M. Ruiz-Pérez, D. González-Pacanowska, F. Vicente and O. Genilloud, *J. Nat. Prod.*, 2014, 77, 2118-2123.

195. T. Yim, K. Kanokmedhakul, S. Kanokmedhakul, W. Sanmanoch and S. Boonlue, *Nat. Prod. Res.*, 2014, 28, 1847-1852.

196. O.-a. Rajachan, S. Kanokmedhakul, K. Kanokmedhakul and K. Soytong, *Planta Med.*, 2014, 80, 1635-1640.

197. G. Chianese, M. Persico, F. Yang, H.-W. Lin, Y.-W. Guo, N. Basilico, S. Parapini, D. Taramelli, O. Taglialatela-Scafati and C. Fattorusso, *Bioorg. Med. Chem.*, 2014, 22, 4572-4580.

198. F. M. Talontsi, M. Lamshöft, C. Douanla-Meli, S. F. Kouam and M. Spiteller, *Fitoterapia*, 2014, 93, 233-238.

199. R. Raju, Z. G. Khalil, A. M. Piggott, A. Blumenthal, D. L. Gardiner, T. S. Skinner-Adams and R. J. Capon, *Org. Lett.*, 2014, 16, 1716-1719.

200. Y. Hata, M. Raith, S. N. Ebrahimi, S. Zimmermann, T. Mokoka, D. Naidoo, G. Fouche, V. Maharaj, M. Kaiser and R. Brun, *Planta Med.*, 2013, 79, 492-498.

201. G. Bringmann, B. K. Lombe, C. Steinert, K. N. Ioset, R. Brun, F. Turini, G. n. Heubl and V. Mudogo, *Org. Lett.*, 2013, 15, 2590-2593.

202. G. Bringmann, G. Zhang, T. Ölschläger, A. Stich, J. Wu, M. Chatterjee and R. Brun, *Phytochemistry*, 2013, 91, 220-228.

203. Z. E. dos Santos Torres, E. R. Silveira, L. F. Rocha e Silva, E. S. Lima, M. C. de Vasconcellos, D. E. de Andrade Uchoa and A. M. Pohlit, *Molecules*, 2013, 18, 6281-6297.

204. F. M. Talontsi, M. Lamshöft, J. O. Bauer, A. A. Razakarivony, B. Andriamihaja, C. Strohmann and M. Spiteller, *J. Nat. Prod.*, 2013, 76, 97-102.

205. C. O. Ochieng, L. A. Manguro, P. O. Owuor and H. Akala, *Bioorg. Med. Chem. Lett.*, 2013, 23, 3088-3095.

206. E. Ajaiyeoba, O. Ogbole, O. Abiodun, J. Ashidi, P. Houghton and C. W. Wright, *J. Parasitol. Res.*, 2013, 2013.

207. S. F. Kouam, A. W. Ngouonpe, A. Bullach, M. Lamshöft, G. M. Kuigoua and M. Spiteller, *Fitoterapia*, 2013, 91, 199-204.

208. P. Moosophon, S. Kanokmedhakul, K. Kanokmedhakul, M. Buayairaksa, J. Noichan and K. Poopasit, *J. Nat. Prod.*, 2013, 76, 1298-1302.

209. A. R. Carroll, V. M. Avery, S. Duffy, P. I. Forster and G. P. Guymer, *Org. Biomol. Chem.*, 2013, 11, 453-458.

210. A. A. Nasrullah, A. Zahari, J. Mohamad and K. Awang, *Molecules*, 2013, 18, 8009-8017.

211. D. Zofou, E. L. Tematio, F. Ntie-Kang, M. Tene, M. N. Ngemenya, P. Tane and V. P. Titanji, *PLoS One*, 2013, 8, e79544.

212. R. Graziose, M. H. Grace, T. Rathinasabapathy, P. Rojas-Silva, C. Dekock, A. Poulev, M. A. Lila, P. Smith and I. Raskin, *Phytochemistry*, 2013, 87, 78-85.

213. N. Wongsa, S. Kanokmedhakul, K. Kanokmedhakul, P. Kongsaeree, S. Prabpai and S. G. Pyne, *Phytochemistry*, 2013, 95, 368-374.

214. V. D. Claudino, K. C. d. Silva, V. Cechinel Filho, R. A. Yunes, F. D. Monache, A. Giménez, E. Salamanca, D. Gutierrez-Yapu and A. Malheiros, *Mem. Inst. Oswaldo Cruz*, 2013, 108, 140-144.

215. O.-a. Rajachan, S. Kanokmedhakul, P. Nasomjai and K. Kanokmedhakul, *Nat. Prod. Res.*, 2014, 28, 268-270.

216. M. A. Tantry, A. Idris and I. A. Khan, *Fitoterapia*, 2013, 89, 58-67.

217. H. Yusuf, M. Mustofa, R. A. Susidarti, P. B. S. Asih and S. Suryawati, *Int. J. Res. Pharm. Biomed. Sci.*, 2013, 4, 728-734.

218. K. V. Sashidhara, S. P. Singh, S. V. Singh, R. K. Srivastava, K. Srivastava, J. Saxena and S. K. Puri, *Eur. J. Med. Chem.*, 2013, 60, 497-502.

219. C. Levrier, M. Balastrier, K. D. Beattie, A. R. Carroll, F. Martin, V. Choomuenwai and R. A. Davis, *Phytochemistry*, 2013, 86, 121-126.

220. V. K. Dua, G. Verma, B. Singh, A. Rajan, U. Bagai, D. D. Agarwal, N. Gupta, S. Kumar and A. Rastogi, *Malar. J.*, 2013, 12, 194.

221. V. Hadi, M. Hotard, T. Ling, Y. G. Salinas, G. Palacios, M. Connelly and F. Rivas, *Eur. J. Med. Chem.*, 2013, 65, 376-380.

222. C. Obbo, B. Makanga, D. Mulholland, P. Coombes and R. Brun, *J. Ethnopharmacol.*, 2013, 147, 220-223.

223. N. Abdissa, M. Induli, H. M. Akala, M. Heydenreich, J. O. Midiwo, A. Ndakala and A. Yenesew, *Phytochem. Lett.*, 2013, 6, 241-245.

224. M. T. Ludere, T. Van Ree and R. Vleggaar, *Fitoterapia*, 2013, 86, 188-192.

225. B. Hao, S.-F. Shen and Q.-J. Zhao, *Molecules*, 2013, 18, 2458-2468.

226. L. Harinantenaina, J. D. Bowman, P. J. Brodie, C. Slebodnick, M. W. Callmander, E. Rakotobe, R. Randrianaivo, V. E. Rasamison, A. Gorka and P. D. Roepe, *J. Nat. Prod.*, 2013, 76, 388-393.

227. P. Wangchuk, P. A. Keller, S. G. Pyne, W. Lie, A. C. Willis, R. Rattanajak and S. Kamchonwongpaisan, *J. Ethnopharmacol.*, 2013, 150, 953-959.

228. M. B. Simelane, A. Shonhai, F. O. Shode, P. Smith, M. Singh and A. R. Opoku, *Molecules*, 2013, 18, 12313-12323.

229. A. L. Eaton, L. Harinantenaina, P. J. Brodie, M. B. Cassera, J. D. Bowman, M. W. Callmander, R. Randrianaivo, R. Rakotondrajaona, E. Rakotobe and V. E. Rasamison, *Nat. Prod. Commun.*, 2013, 8, 1201.

230. H. Wang, W. Zhao, V. Choomuenwai, K. T. Andrews, R. J. Quinn and Y. Feng, *Bioorg. Med. Chem. Lett.*, 2013, 23, 5915-5918.

231. B. S. Bagatela, A. P. Lopes, F. L. A. Fonseca, M. A. Andreo, D. N. Nanayakkara, J. K. Bastos and F. F. Perazzo, *Nat. Prod. Res.*, 2013, 27, 2202-2209.

232. S. Mohanty, P. Srivastava, A. K. Maurya, H. S. Cheema, K. Shanker, S. Dhawan, M. P. Darokar and D. U. Bawankule, *J. Ethnopharmacol.*, 2013, 149, 797-802.

233. B. Taiwo, E. Akinkunmi and N. Omisore, *Afr. J. Tradit., Complementary Altern. Med.*, 2013, 10, 528-531.

234. K. H. Lee and K.-H. Rhee, *Arch. Pharmacal Res.*, 2013, 36, 430-435.

235. B. M. Mba’ning, B. N. Lenta, D. T. Noungoué, C. Antheaume, Y. F. Fongang, S. A. Ngouela, F. F. Boyom, P. J. Rosenthal, E. Tsamo and N. Sewald, *Phytochemistry*, 2013, 96, 347-352.

236. S. N. Ebrahimi, S. Zimmermann, J. Zaugg, M. Smiesko, R. Brun and M. Hamburger, *Planta Med.*, 2013, 29, 150-156.

237. B. Baghdikian, V. Mahiou-Leddet, S. Bory, S.-S. Bun, A. Dumetre, F. Mabrouki, S. Hutter, N. Azas and E. Ollivier, *J. Ethnopharmacol.*, 2013, 145, 381-385.

238. P. Seephonkai, S. G. Pyne, A. C. Willis and W. Lie, *J. Nat. Prod.*, 2013, 76, 1358-1364.

239. M.-C. Jonville, G. Dive, L. Angenot, J. Bero, M. Tits, E. Ollivier and M. Frédérich, *Phytochemistry*, 2013, 87, 157-163.

240. Y. Liu, L. Harinantenaina, P. J. Brodie, J. D. Bowman, M. B. Cassera, C. Slebodnick, M. W. Callmander, R. Randrianaivo, E. Rakotobe and V. E. Rasamison, *Bioorg. Med. Chem.*, 2013, 21, 7591-7594.

241. E. S. Lima, M. C. d. Vasconcellos, E. S. P. Aranha, D. S. Costa, E. V. Mustafa, S. K. R. d. Morais, M. d. G. C. Alecrim, S. M. Nunomura, L. Struwe and V. F. d. Andrade-Neto, *Mem. Inst. Oswaldo Cruz*, 2013, 108, 501-507.

242. F. Machumi, J. O. Midiwo, M. R. Jacob, S. I. Khan, B. L. Tekwani, J. Zhang, L. A. Walker and I. Muhammad, *Nat. Prod. Commun.*, 2013, 8, 761.

243. N. J. Toyang, M. A. Krause, R. M. Fairhurst, P. Tane, J. Bryant and R. Verpoorte, *J. Ethnopharmacol.*, 2013, 147, 618-621.

244. L. Calcul, C. Waterman, W. S. Ma, M. D. Lebar, C. Harter, T. Mutka, L. Morton, P. Maignan, A. V. Olphen and D. E. Kyle, *Mar. Drugs*, 2013, 11, 5036-5050.

245. L. P. Liew, M. Kaiser and B. R. Copp, *Bioorg. Med. Chem. Lett.*, 2013, 23, 452-454.

246. F. Farokhi, P. Grellier, M. Clément, C. Roussakis, P. M. Loiseau, E. Genin-Seward, J.-M. Kornprobst, G. Barnathan and G. Wielgosz-Collin, *Mar. Drugs*, 2013, 11, 1304-1315.

247. J.-B. Gallé, B. Attioua, M. Kaiser, A.-M. Rusig, A. Lobstein and C. Vonthron-Sénécheau, *Mar. Drugs*, 2013, 11, 599-610.

248. M. E. Teasdale, J. Prudhomme, M. Torres, M. Braley, S. Cervantes, S. C. Bhatia, J. J. La Clair, K. Le Roch and J. Kubanek, *ACS Med. Chem. Lett.*, 2013, 4, 989-993.

249. K. W. Von Bargen, E.-M. Niehaus, K. Bergander, R. Brun, B. Tudzynski and H.-U. Humpf, *J. Nat. Prod.*, 2013, 76, 2136-2140.

250. T. Sirirak, L. Brecker and A. Plubrukarn, *Nat. Prod. Res.*, 2013, 27, 1213-1219.

251. R. A. Davis, S. Duffy, S. Fletcher, V. M. Avery and R. J. Quinn, *J. Org. Chem.*, 2013, 78, 9608-9613.

252. C. Boonlarppradab, C. Suriyachadkun, P. Rachtawee and W. Choowong, *J. Antibiot.*, 2013, 66, 305.

253. E. Zafrir Ilan, M. R. Torres, J. Prudhomme, K. Le Roch, P. R. Jensen and W. Fenical, *J. Nat. Prod.*, 2013, 76, 1815-1818.

254. H. Ganfon, J. Bero, A. T. Tchinda, F. Gbaguidi, J. Gbenou, M. Moudachirou, M. Frédérich and J. Quetin-Leclercq, *J. Ethnopharmacol.*, 2012, 141, 411-417.

255. M. A. Abdalla and H. Laatsch, *Afr. J. Tradit., Complementary Altern. Med.*, 2012, 9, 56-58.

256. G. Bringmann, G. Zhang, T. Büttner, G. Bauckmann, T. Kupfer, H. Braunschweig, R. Brun and V. Mudogo, *Chem. Eur. J.*, 2013, 19, 916-923.

257. C. O. Ochieng, P. O. Owuor, L. A. Mang'uro, H. Akala and I. O. Ishola, *Fitoterapia*, 2012, 83, 74-80.

258. J. Deguchi, T. Hirahara, Y. Hirasawa, W. Ekasari, A. Widyawaruyanti, O. Shirota, M. Shiro and H. Morita, *Chem. Pharm. Bull.*, 2012, 60, 219-222.

259. R. Graziose, P. Rojas-Silva, T. Rathinasabapathy, C. Dekock, M. H. Grace, A. Poulev, M. A. Lila, P. Smith and I. Raskin, *J. Ethnopharmacol.*, 2012, 142, 456-461.

260. P. Wangchuk, P. A. Keller, S. G. Pyne, A. C. Willis and S. Kamchonwongpaisan, *J. Ethnopharmacol.*, 2012, 143, 310-313.

261. P. Wangchuk, P. A. Keller, S. G. Pyne, T. Sastraruji, M. Taweechotipatr, R. Rattanajak, A. Tonsomboon and S. Kamchonwongpaisan, *Nat. Prod. Commun.*, 2012, 7, 575-580.

262. O. Jansen, M. Tits, L. Angenot, J.-P. Nicolas, P. De Mol, J.-B. Nikiema and M. Frédérich, *Malar. J.*, 2012, 11, 289.

263. D. Dastan, P. Salehi, A. R. Gohari, S. Zimmermann, M. Kaiser, M. Hamburger, H. R. Khavasi and S. N. Ebrahimi, *Phytochemistry*, 2012, 78, 170-178.

264. F. Mbeunkui, M. H. Grace, C. Lategan, P. J. Smith, I. Raskin and M. A. Lila, *J. Ethnopharmacol.*, 2012, 139, 471-477.

265. H. Morita, R. Mori, J. Deguchi, S. Oshimi, Y. Hirasawa, W. Ekasari, A. Widyawaruyanti and A. H. A. Hadi, *J. Nat. Med.*, 2012, 66, 571-575.

266. I. Zakaria, N. Ahmat, F. M. Jaafar and A. Widyawaruyanti, *Fitoterapia*, 2012, 83, 968-972.

267. M. Girardot, C. Deregnaucourt, A. Deville, L. Dubost, R. Joyeau, L. Allorge, P. Rasoanaivo and L. Mambu, *Phytochemistry*, 2012, 73, 65-73.

268. J. C. Chukwujekwu, C. A. De Kock, P. J. Smith, F. R. Van Heerden and J. Van Staden, *Planta Med.*, 2012, 78, 1857-1860.

269. B. Attioua, D. Yeo, L. Lagnika, R. Harisolo, C. Antheaume, B. Weniger, M. Kaiser, A. Lobstein and C. Vonthron-Sénécheau, *Pharm. Biol.*, 2012, 50, 801-806.

270. T. R. Morais, P. Romoff, O. A. Fávero, J. Q. Reimão, W. C. Lourenço, A. G. Tempone, A. D. Hristov, S. M. Di Santi, J. H. G. Lago and P. Sartorelli, *Parasitol. Res.*, 2012, 110, 95-101.

271. D. Ndjonka, B. Bergmann, C. Agyare, F. M. Zimbres, K. Lüersen, A. Hensel, C. Wrenger and E. Liebau, *Parasitol. Res.*, 2012, 111, 827-834.

272. S. Bertani, E. Houël, V. Jullian, G. Bourdy, A. Valentin, D. Stien and E. Deharo, *Exp. Parasitol.*, 2012, 130, 341-347.

273. A. Hiranrat, W. Mahabusarakam, A. R. Carroll, S. Duffy and V. M. Avery, *J. Org. Chem.*, 2011, 77, 680-683.

274. R. G. Kamkumo, A. M. Ngoutane, L. R. Tchokouaha, P. V. Fokou, E. A. Madiesse, J. Legac, J. J. Kezetas, B. N. Lenta, F. F. Boyom and T. Dimo, *Malar. J.*, 2012, 11, 382.

275. L. C. Albernaz, A. Deville, L. Dubost, J. E. de Paula, B. Bodo, P. Grellier, L. S. Espindola and L. Mambu, *Planta Med.*, 2012, 78, 459-464.

276. M. Frédérich, *Planta Med.*, 2012, 78, 377-382.

277. V. Lakshmi, S. Srivastava, S. K. Mishra, M. N. Srivastava, K. Srivastava and S. K. Puri, *Nat. Prod. Res.*, 2012, 26, 1012-1015.

278. A. R. Carroll, S. J. Wild, S. Duffy and V. M. Avery, *Tetrahedron Lett.*, 2012, 53, 2873-2875.

279. I. W. Mudianta, T. Skinner-Adams, K. T. Andrews, R. A. Davis, T. A. Hadi, P. Y. Hayes and M. J. Garson, *J. Nat. Prod.*, 2012, 75, 2132-2143.

280. M. E. Teasdale, T. L. Shearer, S. Engel, T. S. Alexander, C. R. Fairchild, J. Prudhomme, M. Torres, K. Le Roch, W. Aalbersberg and M. E. Hay, *J. Org. Chem.*, 2012, 77, 8000-8006.

281. A. R. Carroll, B. D. Nash, S. Duffy and V. M. Avery, *J. Nat. Prod.*, 2012, 75, 1206-1209.

282. J. Gao, M. M. Radwan, F. León, X. Wang, M. R. Jacob, B. L. Tekwani, S. I. Khan, S. Lupien, R. A. Hill and F. M. Dugan, *Med. Chem. Res.*, 2012, 21, 3080-3086.

283. N. El Aouad, G. Pérez-Moreno, P. Sánchez, J. Cantizani, F. J. Ortiz-López, J. s. Martín, V. González-Menéndez, L. M. Ruiz-Pérez, D. González-Pacanowska and F. Vicente, *J. Nat. Prod.*, 2012, 75, 1228-1230.

284. M. Ilias, M. A. Ibrahim, S. I. Khan, M. R. Jacob, B. L. Tekwani, L. A. Walker and V. Samoylenko, *Planta Med.*, 2012, 78, 1690-1697.

285. X.-F. Liu, Y. Shen, F. Yang, M. T. Hamann, W.-H. Jiao, H.-J. Zhang, W.-S. Chen and H.-W. Lin, *Tetrahedron*, 2012, 68, 4635-4640.

286. G. Carr, E. R. Derbyshire, E. Caldera, C. R. Currie and J. Clardy, *J. Nat. Prod.*, 2012, 75, 1806-1809.

287. M. Kumarihamy, S. I. Khan, M. Jacob, B. L. Tekwani, S. O. Duke, D. Ferreira and N. D. Nanayakkara, *J. Nat. Prod.*, 2012, 75, 883-889.

288. K. Supong, C. Thawai, K. Suwanborirux, W. Choowong, S. Supothina and P. Pittayakhajonwut, *Phytochem. Lett.*, 2012, 5, 651-656.

289. N. Chanthathamrongsiri, S. Yuenyongsawad, C. Wattanapiromsakul and A. Plubrukarn, *J. Nat. Prod.*, 2012, 75, 789-792.

290. L. Mani, V. Jullian, B. Mourkazel, A. Valentin, J. Dubois, T. Cresteil, E. Folcher, J. N. Hooper, D. Erpenbeck and W. Aalbersberg, *Chem. Biodiversity*, 2012, 9, 1436-1451.

291. M. Isaka, S. Palasarn, P. Tobwor, T. Boonruangprapa and K. Tasanathai, *J. Antibiot.*, 2012, 65, 571.

292. R. A. Davis, M. S. Buchanan, S. Duffy, V. M. Avery, S. A. Charman, W. N. Charman, K. L. White, D. M. Shackleford, M. D. Edstein and K. T. Andrews, *J. Med. Chem.*, 2012, 55, 5851-5858.

293. A. A. Rahman, V. Samoylenko, S. K. Jain, B. L. Tekwani, S. I. Khan, M. R. Jacob, J. O. Midiwo, J. P. Hester, L. A. Walker and I. Muhammad, *Nat. Prod. Commun.*, 2011, 6, 1645.

294. S. Vitalini, G. Beretta, M. Iriti, S. Orsenigo, N. Basilico, S. Dall'Acqua, M. Iorizzi and G. Fico, *Acta Biochim. Pol.*, 2011, 58, 203-219.

295. J. Phongmaykin, T. Kumamoto, T. Ishikawa, E. Saifah and R. Suttisri, *Nat. Prod. Res.*, 2011, 25, 1621-1628.

296. M. Adams, S. Gschwind, S. Zimmermann, M. Kaiser and M. Hamburger, *J. Ethnopharmacol.*, 2011, 135, 43-47.

297. H.-I. Moon, J.-H. Lee, Y.-C. Lee and K.-S. Kim, *Immunopharmacol. Immunotoxicol.*, 2011, 33, 663-666.

298. R. Ortet, S. Prado, E. L. Regalado, F. A. Valeriote, J. Mendiola and O. P. Thomas, *J. Ethnopharmacol.*, 2011, 138, 637-640.

299. E. Pan, A. P. Gorka, J. N. Alumasa, C. Slebodnick, L. Harinantenaina, P. J. Brodie, P. D. Roepe, R. Randrianaivo, C. Birkinshaw and D. G. Kingston, *J. Nat. Prod.*, 2011, 74, 2174-2180.

300. N. Wongsa, S. Kanokmedhakul and K. Kanokmedhakul, *Phytochemistry*, 2011, 72, 1859-1864.

301. J. Deguchi, T. Hirahara, S. Oshimi, Y. Hirasawa, W. Ekasari, O. Shirota, T. Honda and H. Morita, *Org. Lett.*, 2011, 13, 4344-4347.

302. D. Lacroix, S. Prado, D. Kamoga, J. Kasenene and B. Bodo, *J. Nat. Prod.*, 2011, 74, 2286-2289.

303. M. K. Langat, N. R. Crouch, P. J. Smith and D. A. Mulholland, *J. Nat. Prod.*, 2011, 74, 2349-2355.

304. M. S. Gachet, O. Kunert, M. Kaiser, R. Brun, M. Zehl, W. Keller, R. A. Munoz, R. Bauer and W. Schuehly, *J. Nat. Prod.*, 2011, 74, 559-566.

305. J. V. Becker, M. M. Van der Merwe, A. C. van Brummelen, P. Pillay, B. G. Crampton, E. M. Mmutlane, C. Parkinson, F. R. Van Heerden, N. R. Crouch and P. J. Smith, *Malar. J.*, 2011, 10, 295.

306. X. Yang, Y. Feng, S. Duffy, V. M. Avery, D. Camp, R. J. Quinn and R. A. Davis, *Planta Med.*, 2011, 77, 1644-1647.

307. M. Maas, A. Hensel, F. B. da Costa, R. Brun, M. Kaiser and T. J. Schmidt, *Phytochemistry*, 2011, 72, 635-644.

308. S. P. Ovenden, M. Cobbe, R. Kissell, G. W. Birrell, M. Chavchich and M. D. Edstein, *J. Nat. Prod.*, 2010, 74, 74-78.

309. D. Zofou, T. K. Kowa, H. K. Wabo, M. N. Ngemenya, P. Tane and V. P. Titanji, *Malar. J.*, 2011, 10, 167.

310. D. Zofou, A. B. O. Kengne, M. Tene, M. N. Ngemenya, P. Tane and V. P. Titanji, *Parasitol. Res.*, 2011, 108, 1383-1390.

311. R. Graziose, T. Rathinasabapathy, C. Lategan, A. Poulev, P. J. Smith, M. Grace, M. A. Lila and I. Raskin, *J. Ethnopharmacol.*, 2011, 133, 26-30.

312. C. Ramalhete, D. Lopes, J. Molnár, S. Mulhovo, V. E. Rosário and M.-J. U. Ferreira, *Bioorg. Med. Chem.*, 2011, 19, 330-338.

313. C. Ramalhete, F. P. da Cruz, D. Lopes, S. Mulhovo, V. E. Rosário, M. Prudêncio and M.-J. U. Ferreira, *Bioorg. Med. Chem.*, 2011, 19, 7474-7481.

314. M. Endale, J. P. Alao, H. M. Akala, N. K. Rono, F. L. Eyase, S. Derese, A. Ndakala, M. Mbugua, D. S. Walsh and P. Sunnerhagen, *Planta Med.*, 2012, 78, 31-35.

315. M. S. Sá, M. N. de Menezes, A. U. Krettli, I. M. Ribeiro, T. C. Tomassini, R. Ribeiro dos Santos, W. F. de Azevedo Jr and M. B. Soares, *J. Nat. Prod.*, 2011, 74, 2269-2272.

316. A. A. Rahman, V. Samoylenko, M. R. Jacob, R. Sahu, S. K. Jain, S. I. Khan, B. L. Tekwani and I. Muhammad, *Planta Med.*, 2011, 77, 1639.

317. M. Moridi Farimani, M. B. Bahadori, S. Taheri, S. N. Ebrahimi, S. Zimmermann, R. Brun, G. Amin and M. Hamburger, *J. Nat. Prod.*, 2011, 74, 2200-2205.

318. S. Ślusarczyk, S. Zimmermann, M. Kaiser, A. Matkowski, M. Hamburger and M. Adams, *Planta Med.*, 2011, 77, 1594-1596.

319. F. Mbeunkui, M. H. Grace, C. Lategan, P. J. Smith, I. Raskin and M. A. Lila, *J. Chromatogr. B: Anal. Technol. Biomed. Life Sci.*, 2011, 879, 1886-1892.

320. R. Rodríguez-Guzmán, L. C. J. Fulks, M. M. Radwan, C. L. Burandt and S. A. Ross, *Planta Med.*, 2011, 77, 1542-1544.

321. P. Panseeta, K. Lomchoey, S. Prabpai, P. Kongsaeree, A. Suksamrarn, S. Ruchirawat and S. Suksamrarn, *Phytochemistry*, 2011, 72, 909-915.

322. A. R. Carroll, S. Duffy, M. Sykes and V. M. Avery, *Org. Biomol. Chem.*, 2011, 9, 604-609.

323. N. Panthama, S. Kanokmedhakul, K. Kanokmedhakul and K. Soytong, *J. Nat. Prod.*, 2011, 74, 2395-2399.

324. S. Cao and J. Clardy, *Tetrahedron Lett.*, 2011, 52, 2206-2208.

325. L. Mani, S. Petek, A. Valentin, S. Chevalley, E. Folcher, W. Aalbersberg and C. Debitus, *Nat. Prod. Res.*, 2011, 25, 1923-1930.

326. M. Isaka, P. Chinthanom, M. Sappan, R. Chanthaket, J. J. Luangsa-ard, S. Prabpai and P. Kongsaeree, *J. Nat. Prod.*, 2011, 74, 2143-2150.

327. A. Tripathi, J. Puddick, M. R. Prinsep, M. Rottmann, K. P. Chan, D. Y.-K. Chen and L. T. Tan, *Phytochemistry*, 2011, 72, 2369-2375.

328. H. Huang, Y. Yao, Z. He, T. Yang, J. Ma, X. Tian, Y. Li, C. Huang, X. Chen and W. Li, *J. Nat. Prod.*, 2011, 74, 2122-2127.

329. E. Moreno, T. Varughese, C. Spadafora, A. E. Arnold, P. D. Coley, T. A. Kursar, W. H. Gerwick and L. Cubilla-Rios, *Nat. Prod. Commun.*, 2011, 6, 835.

330. T. Sirirak, S. Kittiwisut, C. Janma, S. Yuenyongsawad, K. Suwanborirux and A. Plubrukarn, *J. Nat. Prod.*, 2011, 74, 1288-1292.

331. M. Iwatsuki, S. Takada, M. Mori, A. Ishiyama, M. Namatame, A. Nishihara-Tsukashima, K. Nonaka, R. Masuma, K. Otoguro and K. Shiomi, *J. Antibiot.*, 2011, 64, 183.

332. C. Hemtasin, S. Kanokmedhakul, K. Kanokmedhakul, C. Hahnvajanawong, K. Soytong, S. Prabpai and P. Kongsaeree, *J. Nat. Prod.*, 2011, 74, 609-613.

333. M. Xu, K. T. Andrews, G. W. Birrell, T. L. Tran, D. Camp, R. A. Davis and R. J. Quinn, *Bioorg. Med. Chem. Lett.*, 2011, 21, 846-848.

334. S. T. Chan, A. N. Pearce, M. J. Page, M. Kaiser and B. R. Copp, *J. Nat. Prod.*, 2011, 74, 1972-1979.

335. M. Isaka, U. Srisanoh, W. Choowong and T. Boonpratuang, *Org. Lett.*, 2011, 13, 4886-4889.

336. E. Galeano, O. P. Thomas, S. Robledo, D. Munoz and A. Martinez, *Mar. Drugs*, 2011, 9, 1902-1913.

337. A. M. Nour, S. A. Khalid, M. Kaiser, R. Brun, E. A. Wai’l and T. J. Schmidt, *J. Ethnopharmacol.*, 2010, 129, 127-130.

338. M. Xu, T. Bruhn, B. Hertlein, R. Brun, A. Stich, J. Wu and G. Bringmann, *Chem. Eur. J.*, 2010, 16, 4206-4216.

339. I. M. Chung, B. K. Ghimire, E. Y. Kang and H. I. Moon, *Phytother. Res.*, 2010, 24, 469-471.

340. M. Bourjot, C. Apel, M.-T. Martin, P. Grellier, F. Guéritte and M. Litaudon, *Planta Med.*, 2010, 76, 1600-1604.

341. F. M. Sebisubi, O. Odyek, W. W. Anokbonggo, J. Ogwal-Okeng, E. J. Carcache-Blanco, C. Ma, J. Orjala and G. T. Tan, *Planta Med.*, 2010, 76, 1870.

342. I. M. Chung, S. H. Seo, E. Y. Kang, W. H. Park, S. D. Park and H. I. Moon, *Phytother. Res.*, 2010, 24, 451-453.

343. V. T. Uchôa, R. C. de Paula, L. G. Krettli, A. E. G. Santana and A. U. Krettli, *Drug Dev. Res.*, 2010, 71, 82-91.

344. T. Sripisut and S. Laphookhieo, *J. Asian Nat. Prod. Res.*, 2010, 12, 614-617.

345. T. Thongthoom, U. Songsiang, C. Phaosiri and C. Yenjai, *Arch. Pharmacal Res.*, 2010, 33, 675-680.

346. F. Machumi, V. Samoylenko, A. Yenesew, S. Derese, J. O. Midiwo, F. T. Wiggers, M. R. Jacob, B. L. Tekwani, S. I. Khan and L. A. Walker, *Nat. Prod. Commun.*, 2010, 5, 853.

347. P. Wangchuk, J. B. Bremner, R. Rattanajak and S. Kamchonwongpaisan, *Phytother. Res.*, 2010, 24, 481-485.

348. J. G. Tangmouo, R. Ho, A. Matheeussen, A. M. Lannang, J. Komguem, B. B. Messi, L. Maes and K. Hostettmann, *Phytother. Res.*, 2010, 24, 1676-1679.

349. F. Ijaz, N. Ahmad, I. Ahmad, A. ul Haq and F. Wang, *J. Enzyme Inhib. Med. Chem.*, 2010, 25, 773-778.

350. A. M. Kaou, V. Mahiou-Leddet, C. Canlet, L. Debrauwer, S. Hutter, M. Laget, R. Faure, N. Azas and E. Ollivier, *J. Ethnopharmacol.*, 2010, 130, 272-274.

351. L. S. Fernandez, M. L. Sykes, K. T. Andrews and V. M. Avery, *Int. J. Antimicrob. Agents*, 2010, 36, 275-279.

352. M. Sathe and M. Kaushik, *Bioorg. Med. Chem. Lett.*, 2010, 20, 1312-1314.

353. M. Sathe, R. Ghorpade, A. Srivastava and M. Kaushik, *J. Ethnopharmacol.*, 2010, 130, 171-174.

354. L. Acebey, V. Jullian, D. Sereno, S. Chevalley, Y. Estevez, C. Moulis, S. Beck, A. Valentin, A. Gimenez and M. Sauvain, *Planta Med.*, 2010, 76, 365-368.

355. H. I. Moon, *Phytother. Res.*, 2010, 24, 941-944.

356. M. S. Gachet, O. Kunert, M. Kaiser, R. Brun, R. A. Munoz, R. Bauer and W. Schühly, *J. Nat. Prod.*, 2010, 73, 553-556.

357. F. Tantangmo, B. Lenta, F. Boyom, S. Ngouela, M. Kaiser, E. Tsamo, B. Weniger, P. Rosenthal and C. Vonthron-Senecheau, *Ann. Trop. Med. Parasitol.*, 2010, 104, 391-398.

358. C. Ramalhete, D. Lopes, S. Mulhovo, J. Molnár, V. E. Rosário and M.-J. U. Ferreira, *Bioorg. Med. Chem.*, 2010, 18, 5254-5260.

359. K. Mesia, R. K. Cimanga, L. Dhooghe, P. Cos, S. Apers, J. Totté, G. L. Tona, L. Pieters, A. J. Vlietinck and L. Maes, *J. Ethnopharmacol.*, 2010, 131, 10-16.

360. L. Dhooghe, S. Maregesi, I. Mincheva, D. Ferreira, J. P. Marais, F. Lemière, A. Matheeussen, P. Cos, L. Maes and A. Vlietinck, *Phytochemistry*, 2010, 71, 785-791.

361. B. N. Lenta, L. M. Kamdem, S. Ngouela, F. Tantangmo, K. P. Devkota, F. F. Boyom, P. J. Rosenthal and E. Tsamo, *Planta Med.*, 2011, 77, 377-379.

362. A. M. Kaou, V. Mahiou-Leddet, C. Canlet, L. Debrauwer, S. Hutter, N. Azas and E. Ollivier, *Fitoterapia*, 2010, 81, 632-635.

363. K. Mishra, D. Chakraborty, A. Pal and N. Dey, *Exp. Parasitol.*, 2010, 124, 421-427.

364. C. P. Osman, N. H. Ismail, R. Ahmad, N. Ahmat, K. Awang and F. M. Jaafar, *Molecules*, 2010, 15, 7218-7226.

365. G. Marti, V. Eparvier, M. Litaudon, P. Grellier and F. Guéritte, *Molecules*, 2010, 15, 7106-7114.

366. X. Mthembu, F. Van Heerden and G. Fouché, *S. Afr. J. Bot.*, 2010, 76, 82-85.

367. S. Cretton, G. Glauser, M. Humam, D. Jeannerat, O. Munoz, L. Maes, P. Christen and K. Hostettmann, *J. Nat. Prod.*, 2010, 73, 844-847.

368. Y. Chen, S. Li, F. Sun, H. Han, X. Zhang, Y. Fan, G. Tai and Y. Zhou, *Pharm. Biol.*, 2010, 48, 1018-1024.

369. A. Chea, S.-S. Bun, N. Azas, M. Gasquet, S. Bory, E. Ollivier and R. Elias, *Nat. Prod. Res.*, 2010, 24, 1766-1770.

370. G. Marti, V. Eparvier, C. Moretti, S. Prado, P. Grellier, N. Hue, O. Thoison, B. Delpech, F. Guéritte and M. Litaudon, *Phytochemistry*, 2010, 71, 964-974.

371. P. Gupta and N. Vasudeva, *Pharm. Biol.*, 2010, 48, 1218-1223.

372. J. Ropivia, S. Derbré, C. Rouger, F. Pagniez, P. Le Pape and P. Richomme, *Molecules*, 2010, 15, 6476-6484.

373. A. A. Wube, F. Bucar, S. Gibbons, K. Asres, L. Rattray and S. L. Croft, *Phytother. Res.*, 2010, 24, 1468-1472.

374. R. A. Davis, S. Duffy, V. M. Avery, D. Camp, J. N. Hooper and R. J. Quinn, *Tetrahedron Lett.*, 2010, 51, 583-585.

375. A. R. Carroll, S. Duffy and V. M. Avery, *J. Org. Chem.*, 2010, 75, 8291-8294.

376. M. Isaka, A. Yangchum, P. Rachtawee, S. Komwijit and A. Lutthisungneon, *J. Nat. Prod.*, 2010, 73, 688-692.

377. R. Haritakun, P. Rachtawee, R. Chanthaket, N. Boonyuen and M. Isaka, *Chem. Pharm. Bull.*, 2010, 58, 1545-1548.

378. F. Scala, E. Fattorusso, M. Menna, O. Taglialatela-Scafati, M. Tierney, M. Kaiser and D. Tasdemir, *Mar. Drugs*, 2010, 8, 2162-2174.

379. A.-S. Lin, E. P. Stout, J. Prudhomme, K. L. Roch, C. R. Fairchild, S. G. Franzblau, W. Aalbersberg, M. E. Hay and J. Kubanek, *J. Nat. Prod.*, 2010, 73, 275-278.

380. E. P. Stout, J. Prudhomme, K. Le Roch, C. R. Fairchild, S. G. Franzblau, W. Aalbersberg, M. E. Hay and J. Kubanek, *Bioorg. Med. Chem. Lett.*, 2010, 20, 5662-5665.

381. J. Kornsakulkarn, C. Thongpanchang, R. Chainoy, W. Choowong, S. Nithithanasilp and T. Thongpanchang, *J. Nat. Prod.*, 2010, 73, 759-762.

382. K. Trisuwan, N. Khamthong, V. Rukachaisirikul, S. Phongpaichit, S. Preedanon and J. Sakayaroj, *J. Nat. Prod.*, 2010, 73, 1507-1511.

383. M. Adams, M. Christen, I. Plitzko, S. Zimmermann, R. Brun, M. Kaiser and M. Hamburger, *J. Nat. Prod.*, 2010, 73, 897-900.

384. X. Yang, R. A. Davis, M. S. Buchanan, S. Duffy, V. M. Avery, D. Camp and R. J. Quinn, *J. Nat. Prod.*, 2010, 73, 985-987.

385. M. Na, Y. Ding, B. Wang, B. L. Tekwani, R. F. Schinazi, S. Franzblau, M. Kelly, R. Stone, X.-C. Li and D. Ferreira, *J. Nat. Prod.*, 2009, 73, 383-387.

386. M. Gutiérrez, K. Tidgewell, T. L. Capson, N. Engene, A. Almanza, J. r. Schemies, M. Jung and W. H. Gerwick, *J. Nat. Prod.*, 2010, 73, 709-711.

387. A. Tripathi, J. Puddick, M. R. Prinsep, M. Rottmann and L. T. Tan, *J. Nat. Prod.*, 2010, 73, 1810-1814.

388. L. Xu, Z. He, J. Xue, X. Chen and X. Wei, *J. Nat. Prod.*, 2010, 73, 885-889.

389. E. L. Regalado, D. Tasdemir, M. Kaiser, N. Cachet, P. Amade and O. P. Thomas, *J. Nat. Prod.*, 2010, 73, 1404-1410.

390. R. A. Davis, A. R. Carroll, K. T. Andrews, G. M. Boyle, T. L. Tran, P. C. Healy, J. A. Kalaitzis and R. G. Shivas, *Org. Biomol. Chem.*, 2010, 8, 1785-1790.

391. P. Pittayakhajonwut, A. Dramae, C. Intaraudom, N. Boonyuen, S. Nithithanasilp, P. Rachtawee and P. Laksanacharoen, *Planta Med.*, 2011, 77, 74-76.

392. C. Fattorusso, M. Persico, B. Calcinai, C. Cerrano, S. Parapini, D. Taramelli, E. Novellino, A. Romano, F. Scala and E. Fattorusso, *J. Nat. Prod.*, 2010, 73, 1138-1145.

393. C. Jiménez-Romero, I. Ortiz, J. Vicente, B. Vera, A. D. Rodríguez, S. Nam and R. Jove, *J. Nat. Prod.*, 2010, 73, 1694-1700.

394. M. Kumarihamy, F. R. Fronczek, D. Ferreira, M. Jacob, S. I. Khan and N. D. Nanayakkara, *J. Nat. Prod.*, 2010, 73, 1250-1253.

395. M. Isaka, P. Chinthanom, S. Supothina, P. Tobwor and N. L. Hywel-Jones, *J. Nat. Prod.*, 2010, 73, 2057-2060.

396. A. Longeon, B. R. Copp, M. Roué, J. Dubois, A. Valentin, S. Petek, C. Debitus and M.-L. Bourguet-Kondracki, *Bioorg. Med. Chem.*, 2010, 18, 6006-6011.

397. M. Isaka, P. Chinthanom, T. Boonruangprapa, N. Rungjindamai and U. Pinruan, *J. Nat. Prod.*, 2010, 73, 683-687.
